# Chiral ion-pair organocatalyst promotes highly enantioselective 3-*exo* iodo-cycloetherification of allyl alcohols†
†Electronic supplementary information (ESI) available: Experimental procedures and characterization for all new compounds. CCDC 1023013 and 1028455. For ESI and crystallographic data in CIF or other electronic format see DOI: 10.1039/c5sc02485d


**DOI:** 10.1039/c5sc02485d

**Published:** 2015-08-27

**Authors:** Zhigao Shen, Xixian Pan, Yisheng Lai, Jiadong Hu, Xiaolong Wan, Xiaoge Li, Hui Zhang, Weiqing Xie

**Affiliations:** a Shaanxi Key Laboratory of Natural Products & Chemical Biology , College of Science , Northwest A&F University , 22 Xinong Road , Yangling 712100 , China . Email: xiewqsioc@aliyun.com; b State Key Laboratory of Natural Medicines , Jiangsu Key Laboratory of Drug Discovery for Metabolic Diseases , Center of Drug Discovery , China Pharmaceutical University , 24 Tongjiaxiang , Nanjing 210009 , China; c Hubei Collaborative Innovation Center for Rare Metal Chemistry , Hubei Normal University , China; d Shanghai Institute of Organic Chemistry , Chinese Academy of Sciences , China; e School of Science & Laboratory for Microstructures , Instrumental Analysis and Research Center , Shanghai University , China

## Abstract

By designing a novel chiral ion-pair organocatalyst composed of chiral phosphate and DABCO-derived quaternary ammonium, highly enantioselective 3-*exo* iodo-cycloetherification of allyl alcohols was achieved using NIS as a halogen source. Based on this reaction, one-pot asymmetric 3-*exo* iodo-cycloetherification/Wagner–Meerwein rearrangement of allyl alcohols en route to enantioenriched 2-iodomethyl-2-aryl cycloalkanones was subsequently developed. Due to the participation of adjacent iodine, the Wagner–Meerwein rearrangement of 2-iodomethyl-2-aryl epoxide proceeds with unusual retention of stereoconfiguration.

## Abstract 2

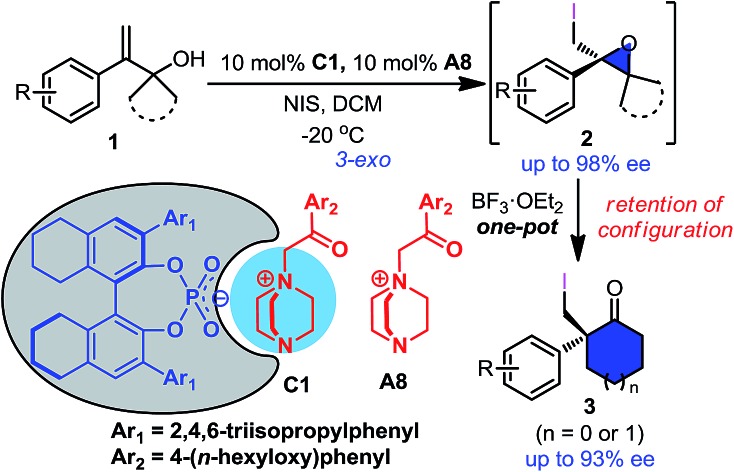
Enantioselective 3-*exo* iodo-cycloetherification of allyl alcohols was realized by employing a novel ion-pair organocatalyst.

Halogenative functionalization of olefins is one of the most important transformations in organic synthesis, as it not only provides a versatile handle for further derivatization, but also delivers highly diastereoselective ring closure when the nucleophile and alkene are tethered together.^1^ Even though applications of halogenation reactions in total synthesis are well documented,^2^ catalytic enantioselective halogenation remains a significant challenge due to the rapid interexchange of the halonium complex between olefins, which leads to rapid racemization of the enantiopure halonium intermediate.^3^ Therefore, limited success has been achieved, despite enormous efforts being devoted to asymmetric halogenation reactions.^4^ Very recently, there has been impressive progress in this field after the landmark reports of Borhan,5*a* Tang,5*b* Fujioka,5*c* Jacobsen,5*d* and Yeung5*e* in 2010, taking advantage of organocatalysts to effect asymmetric halo-lactonization.^5^ Organocatalyzed enantioselective halocyclization of olefinic amines, alcohols and other substrates subsequently emerged.^6^–^9^ However, asymmetric halocyclization reactions are currently limited to the formation of four- to six-membered rings.^5^–^9^ The generation of enantioenriched, more strained three-membered rings *via* catalytic asymmetric halocyclization remains elusive. In this regard, although 3-*exo* halo-cycloetherification of allyl alcohols has long been known,^10^ reactive halogenating agents or harsh reaction conditions are needed to effect the energetically disfavored 3-*exo* halocyclization, which impedes the development of an asymmetric version of this reaction.

With the advent and booming of organocatalysis,11a–c ion-pairing of organocatalysts has emerged as a powerful strategy for designing new efficient organocatalysts.11*d* By cooperatively activating reactive partners, ion-pair catalysts have catalyzed enantioselective reactions that are otherwise difficult to achieve using other organocatalysts. In addition, the ion-pairing strategy also enables catalyst screening *via* combinational approaches, which greatly accelerates the catalyst screening process. Inspired by Toste's recent work8b–f and our work on enantioselective halogenation reactions using chiral anionic phase transfer catalysts,^12^ we postulated that an ion-pair catalyst could facilitate the enantioselective halogenation reaction by cooperative and synergistic activation of both reactants (Fig. 1), which has been responsible for the success of previous catalysts.^5^–^9^ To this end, chiral phosphate was judiciously chosen as counter anion for its fine-tunable chiral pocket as well as its Brønsted basicity to allow interaction with the substrate.^8^ Furthermore, DABCO-derived quaternary ammonium could serve as an excellent candidate for the cation moiety, since its tertiary amine moiety could act as a Lewis base to stabilize the halonium complex, an approach which has been utilized for the synthesis of well-known Selectfluor^13^ and other halogenating reagents.8d,9c,10b

**Fig. 1:**
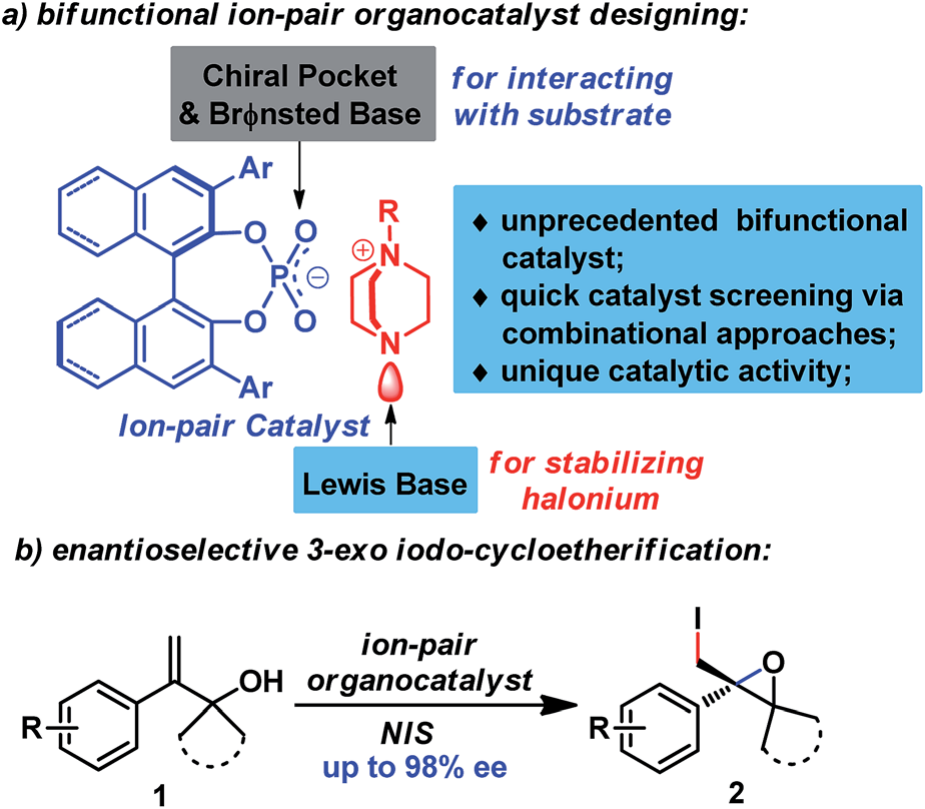
Ion-pair organocatalyst design for enantioselective 3-*exo* iodo-cycloetherification of allyl alcohols.

Herein, we would like to report the success of implementation of the ion-paring strategy, leading to the discovery of a novel ion-pair organocatalyst. This unprecedented organocatalyst enables the first enantioselective 3-*exo* iodo-cycloetherification of allyl alcohols using commercially available NIS as a halogen source. Additionally, this protocol provides direct access to enantiopure 2-iodomethyl epoxides,^14^ which have previously been tedious to prepare from allyl alcohols, requiring an asymmetric Sharpless epoxidation/hydroxyl transformation procedure.^15^

To validate our hypothesis, enantioselective 3-*exo*-iodocyclization of allyl alcohol **1a** was explored using an ion-pair organocatalyst generated *in situ* by combining silver phosphate with DABCO-derived quaternary ammonium salt for convenience of catalyst screening (Table 1). Initially, various ammonium salts were evaluated using 8*H-R*-TRIP-OAg **L1** as a chiral counter-anion source. After extensive screening, **A3** was determined to be a privileged scaffold, affording epoxide **2a** with 77% ee in moderate yield (entries 1–3 and ESI†). In contrast, ammonium salt **A2** derived from quinuclidine provided lower enantioselectivity, showing that the tertiary amine moiety of **A1** played a pivotal role in the reaction (entries 1 and 2). Further structural modification of ammonium salt **A3** revealed that **A8** was the optimal cation fragment for the ion-pair organocatalyst, furnishing epoxide **2a** with 92% ee (entries 3–9). As for the anion fragment, 8*H-R*-TRIP-OAg provided a better result than any other chiral silver phosphate evaluated (see ESI†). Importantly, both cationic and anionic fragments were indispensable for the reaction, as indicated by control experiments (entries 10–12). It should be pointed out that other organocatalysts (*e.g.* chiral phosphoric acid and quinine-derived catalysts) were also surveyed under identical reaction conditions but gave no desired cyclization product, with the starting material being fully recovered (Table S2, ESI†).

**Table 1:** Optimization of reaction conditions for enantioselective 3-*exo*-iodocyclization of allyl alcohol **1a**^*a*^

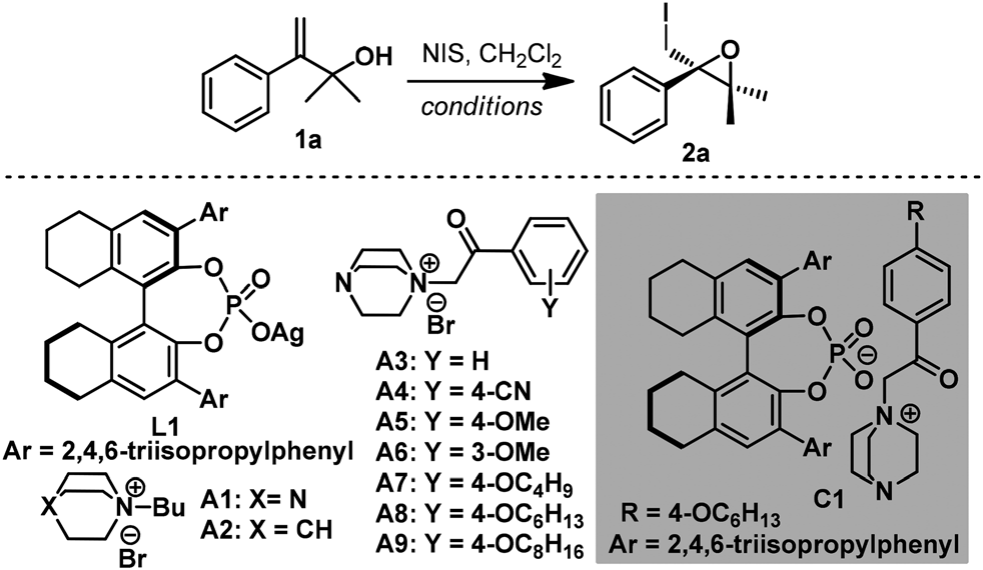						
Entry	Cat. (equiv.)	Additive (equiv.)	*T* (°C)	*t* (h)	Yield^*b*^ (%)	ee^*c*^ (%)
1	**L1** (0.1)	**A1** (0.12)	0	40	16	30
2	**L1** (0.1)	**A2** (0.12)	0	40	18	19
3	**L1** (0.1)	**A3** (0.12)	0	40	44	77
4	**L1** (0.1)	**A4** (0.12)	0	40	16	69
5	**L1** (0.1)	**A5** (0.12)	0	40	69	86
6	**L1** (0.1)	**A6** (0.12)	0	40	47	80
7	**L1** (0.1)	**A7** (0.12)	0	40	65	91
8	**L1** (0.1)	**A8** (0.12)	0	40	60	92
9	**L1** (0.1)	**A9** (0.12)	0	40	50	91
10	—	—	0	40	ND	—
11	**L1** (0.1)	—	0	40	ND	—
12	—	**A8** (0.12)	0	40	ND	—
13	**C1** (0.1)	—	0	40	42	83
14	**C1** (0.1)	**A8** (0.1)	0	40	82	92
15	**C1** (0.1)	S=PPh_3_ (0.1)	0	40	63	90
16^*d*^	**C1** (0.1)	**A8** (0.1)	0	40	62	69
17^*e*^	**C1** (0.1)	**A8** (0.1)	0	40	31	67
18	**C1** (0.1)	**A8** (0.1)	–20	107	99	94

^*a*^CH_2_Cl_2_ (1 mL) was added to a mixture of silver salt **L1** (0.01 mmol), ammonium salt **A** (0.012 mmol) and NIS (0.12 mmol), and the reaction mixture was cooled to 0 °C. Allyl alcohol **1a** (0.1 mmol) in 0.5 mL CH_2_Cl_2_ was then added dropwise, and the reaction was quenched at the indicated time.

^*b*^Isolated yield.

^*c*^Determined by HPLC using a Chiralpak AD column.

^*d*^CHCl_3_ as solvent.

^*e*^EtOAc as solvent. ND = not detected.

With the optimal anionic and cationic moiety of the catalyst identified, ion-pair organocatalyst **C1** was synthesized directly from 8*H-R*-TRIP and ammonium **A8** (see ESI†) and examined under otherwise identical reaction conditions. To our surprise, **2a** was obtained with only moderate enantioselectivity (83% ee, entry 13). As a slight excess of **A8** was used in the *in situ* procedure, we reasoned that **A8** might be an effective promoter for this reaction. Indeed, comparable enantioselectivity (92% ee, entry 14) was obtained by adding a catalytic amount of **A8** to the reaction. It is postulated that **A8** might act as a Lewis base to stabilize the iodonium intermediate8*d* and facilitate the transfer of iodine from NIS to the DABCO moiety of the ion-pair organocatalyst, leading to an acceleration of the reaction rate and increased enantioselectivity. Employing S=PPh_3_ (ref. 7c and e) as an additive also gave a comparable result, verifying the positive effect of a Lewis base as co-catalyst in this reaction (entry 15). With a suitable catalyst in hand, other reaction variations were subsequently evaluated. Other halogenating reagents such as NCS and NBS gave inferior results, leading to no reaction or a sharp drop in enantioselectivity (see ESI†). CH_2_Cl_2_ was determined to be the optimal solvent (entries 16, 17 and ESI†), and lowering the reaction temperature to –20 °C was beneficial for the reaction (entry 18).

After establishing the optimal reaction conditions, the substrate scope of this reaction was examined (Scheme 1). Both electron-withdrawing groups (**2aa–2af** and **2ce-2cf**) and electron-donating groups (**2ag-2ah** and **2ca–2ch**) on the phenyl moiety were tolerated, affording the corresponding epoxides with good to excellent enantioselectivities (87% to 99% ee). Gem-substituents were crucial for the reaction, as **2f** lacking gem-substituents was obtained in only 41% yield and 63% ee. Epoxides with cyclic gem-substituents were obtained with higher enantioselectivities (**2c-2ch** and ESI†) than those with acyclic gem-substituents (**2a** and **2b**). A 2-alkyl substituted allyl alcohol was also smoothly converted to epoxide **2g**, albeit with low enantioselectivity (37% ee). Furthermore, gram syntheses of epoxides **2a** and **2c–2e** were also smoothly realized by using 5 mol% **C1** without affecting enantioselectivities, and the catalyst loading could even be reduced to 1 mol% affording comparable results (Scheme 1 and ESI†). The absolute configuration of epoxide **2** was determined to be *R* based on X-ray crystallographic analysis of epoxide **2ac**,^16^ which was confirmed by vibrational circular dichroism (VCD) studies of epoxide **2c**.^17^

**Scheme 1:**
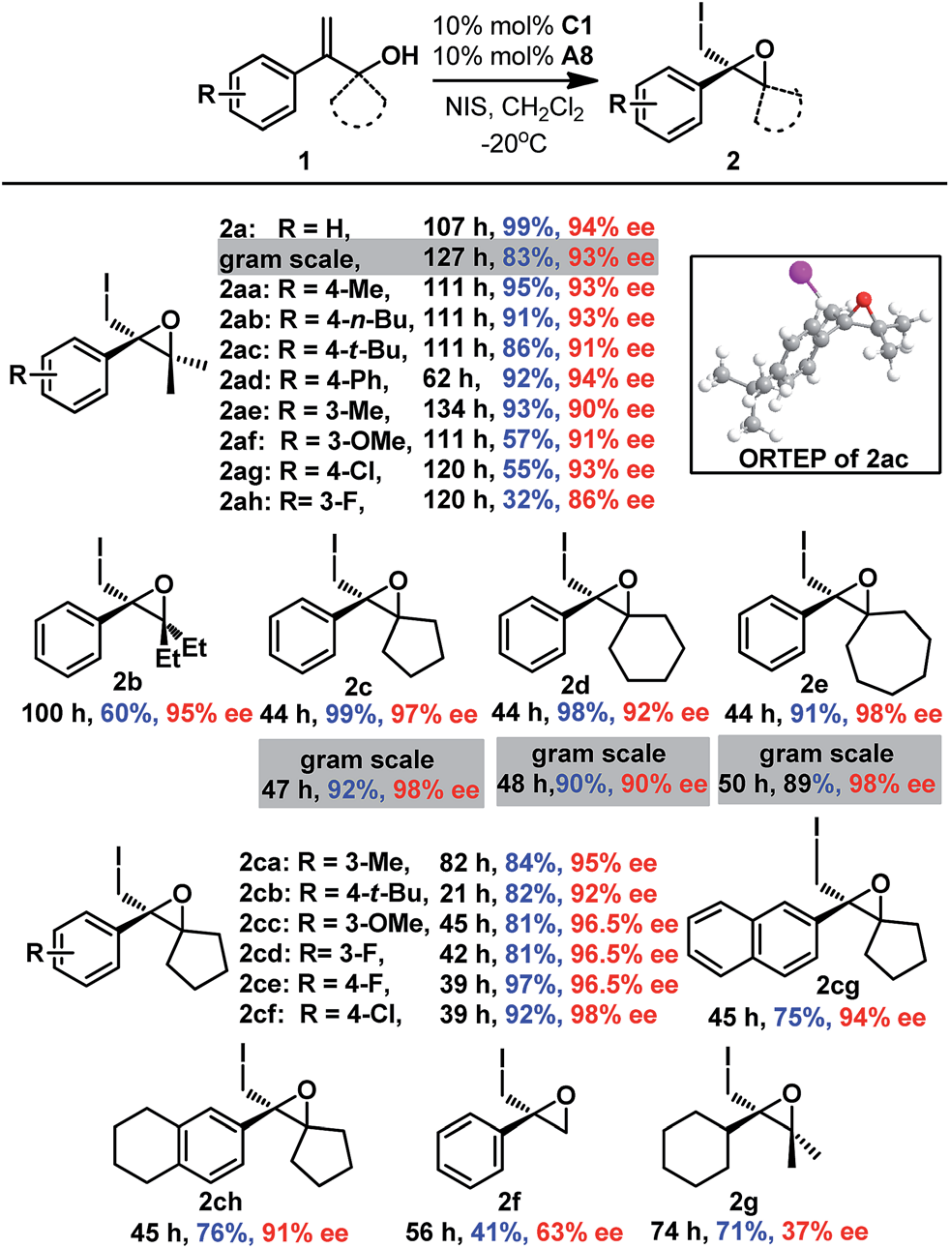
Substrate variation in the enantioselective 3-*exo* iodo-cycloetherification of allyl alcohols.

Next, Wagner–Meerwein rearrangement^18^ of epoxide **2c** was explored for the construction of 2-iodomethyl-2-aryl cyclohexanones with a chiral quaternary carbon center (Scheme 2). BF_3_·Et_2_O was determined to be the most efficient promoter (see ESI†), delivering cyclohexanone **3c** in good yield with partial loss of enantioselectivity (93% ee *vs.* 97% ee for epoxide **2c**). Surprisingly, the absolute configuration of **3c** was established to be *S* by X-ray crystallographic analysis of hydrazone **4** derived from **3c**,^16^ which indicated retention of stereoconfiguration in the Wagner–Meerwein rearrangement. This could be ascribed to the opening of the epoxide by the adjacent iodine to generate iodonium **TS2**, which then rearranged to ketone **3c** with double inversion of configuration. Furthermore, derivatizations of **3c** were also performed to demonstrate its synthetic utility. Substitution of the iodide with NaN_3_ provided azide ketone **5** smoothly, and the iodide could also be converted to an alcohol *via* formyloxylation/hydrolysis^19^ to give hydroxyl ketone **6** in satisfactory yield. It is noteworthy that no erosion of enantiopurity was detected in all these reactions.

**Scheme 2:**
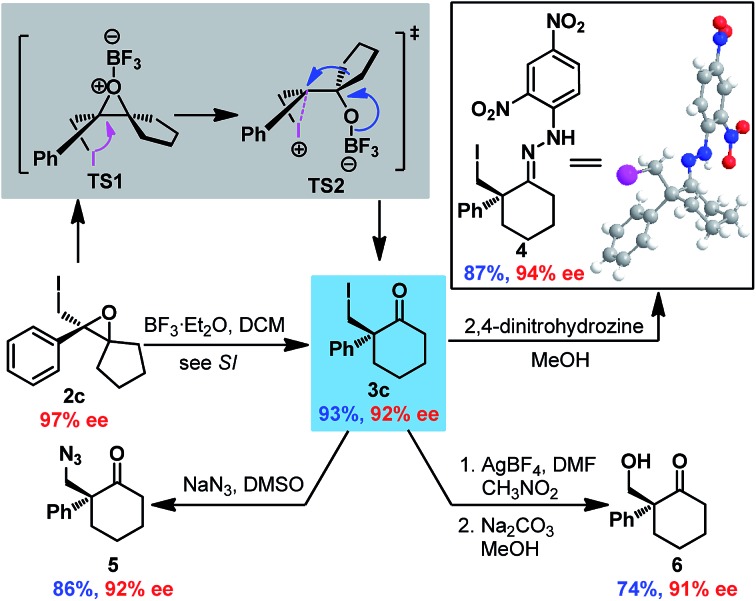
Transformations of spiro-epoxide **2c**.

To simplify the operation, one-pot asymmetric 3-*exo* iodo-cycloetherification/Wagner–Meerwein rearrangement was also developed (Scheme 3). Fortunately, when the iodo-cycloetherification reaction was completed, addition of BF_3_·OEt_2_ to the reaction mixture smoothly provided the desired cyclohexanone **3c** without reducing enantioselectivity, even on a 2.7 mmol scale (92% ee). Different substituents on the phenyl group were found to be compatible with the one-pot process, affording the corresponding cyclohexanones **3c–3f** in satisfactory enantiopurities. Furthermore, seven-membered cycloketone **3g** could also be obtained *via* this one-pot cascade reaction with 91% ee (comparable with that of the corresponding epoxide **2d**), providing a complementary route to previous protocols involving enantioselective halonium-induced semi-Pinacol rearrangement for the enantioselective construction of halogenated cycloheptanones.9a–e

**Scheme 3:**
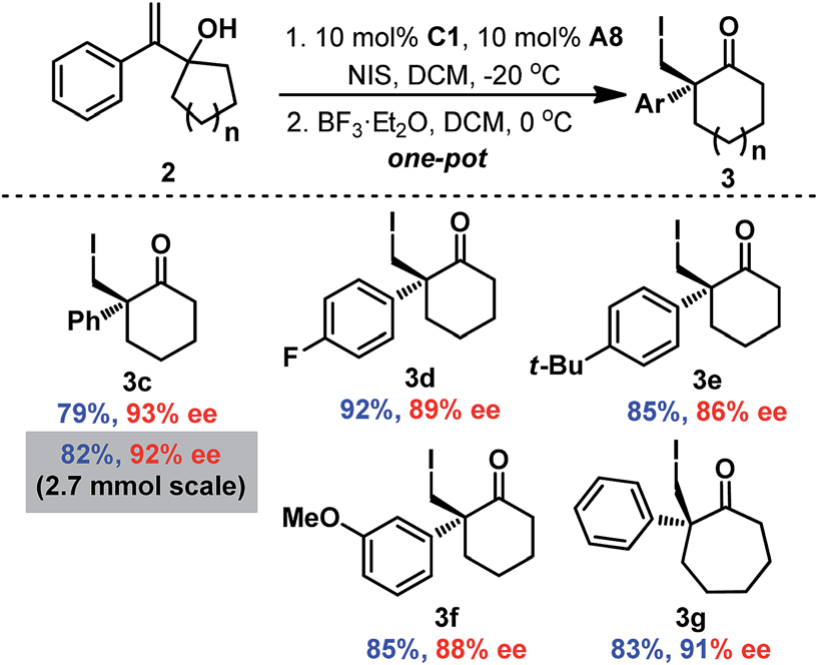
One-pot asymmetric 3-*exo* iodo-cycloetherification/Wagner–Meerwein rearrangement reaction.

## Conclusions

In conclusion, a novel ion-pair organocatalyst comprised of chiral phosphate and DABCO-derived quaternary ammonium was designed, which enabled the first asymmetric 3-*exo* iodo-cycloetherification of allyl alcohols using NIS as a halogenating reagent. By employing this novel catalyst, a variety of enantiopure 2-iodomethyl-2-aryl epoxides were successively prepared with good to excellent enantioselectivities, even on a gram scale. Subsequently, one-pot asymmetric 3-*exo* iodo-cycloetherification/Wagner–Meerwein rearrangement of 2-aryl-2-propen-3-ol was explored, which provided direct access to chiral 2-iodomethyl-2-aryl cycloalkanones with good enantioselectivities. Unusual retention of configuration owing to the assistance of the adjacent iodide was also observed in the Wagner–Meerwein rearrangement.

## Supplementary Material

Supplementary information

Crystal structure data
