# Supplementary material for: Chiral ion-pair organocatalyst promotes highly enantioselective 3-exo iodo-cycloetherification of allyl alcohols
Source: Chem Sci. 2015 Aug 27;6(12):6986–90. doi: 10.1039/c5sc02485d (PMC5947518; doi:10.1039/c5sc02485d)
Supplement: Supplementary file 1 [file SC-006-C5SC02485D-s001.pdf]

Electronic Supplementary Material (ESI) for Chemical Science.
This journal is © The Royal Society of Chemistry 2015

Supporting information for

Chiral Ion-Pair Organocatalyst Promotes Highly Enantioselective 3-*exo* Iodo-cycloetherification of Allyl Alcohols

Zhigao Shen,[a,b,†] Xixian Pan[c,†] Yisheng Lai,[b,†] Jiadong Hu,[b] Xiaolong Wan,[d] Xiaoge Li,[d] Hui Zhang,[e] and Weiqing Xie[a]

Table of contents

1. General information--S2
2. Reaction conditions optimization---S3
3. General procedure for preparation of substrates, ammonium salts and catalyst---S8
4. General procedure for asymmetric 3-*exo* iodo-cycloetherification----------------S18
5. Wagner-Meerwein Rearrangement of epoxide and one-pot procedure --S26
6. References--S33
7. Copies of spectra and HPLC reports---S34
8. VCD and IR experimental of **2c**---S176
9. X-ray crystallography of **2ac** and **4**--- S178

1. General information

Unless otherwise noted, reagents were obtained from commercial sources and used without further purification. Non-aqueous reaction were conducted under an inert atmosphere of nitrogen in flame-dried glassware. Anhydrous solvent were treated as follow: tetrahydrofuran and diethyl ether were distilled from sodium under nitrogen atmosphere, dimethylformamide were distilled over calcium hydride under reduced pressure, and dichloromethane and toluene was distilled distilled from calcium hydride under nitrogen atmosphere. Thin layer chromatography was conducted on Merck 60 F254 pre-coated silica gel plates. Column chromatography was carried out by normal silica gel (40-60 μm, 200-400 mesh, Silicycle P60). NMR data including ^{1}H NMR or ^{13}C NMR spectra were recorded on Agilent 500 and Agilent 400. ^{1}H NMR Chemical shifts were reported in ppm from the solvent resonance as the internal standard ($CDCl_3$: 7.26 ppm, D_2O: 4.79). ^{13}C NMR chemical shifts were reported in ppm relative to the solvent ($CDCl_3$:77 ppm). Infrared spectra were performed on a Nicolet 380FT-IR and are reported in terms of frequency of absorption (cm^{-1}). Low mass spectra were measured on a Shimadzu LCMS-2010EV mass spectrometer (ESI) and Agilent Technologies 5973N (EI). High resolution mass spectra were obtained from IonSpec 4.7 Tesla FTMS mass spectrometer (MALDI) , Bruker APEXIII 7.0 TESLA FTMS (ESI) and Waters Micromass GCT Premier (EI).

2. Reaction conditions optimization

Table S1. Evaluation of chiral silver phosphate and ammonium salts

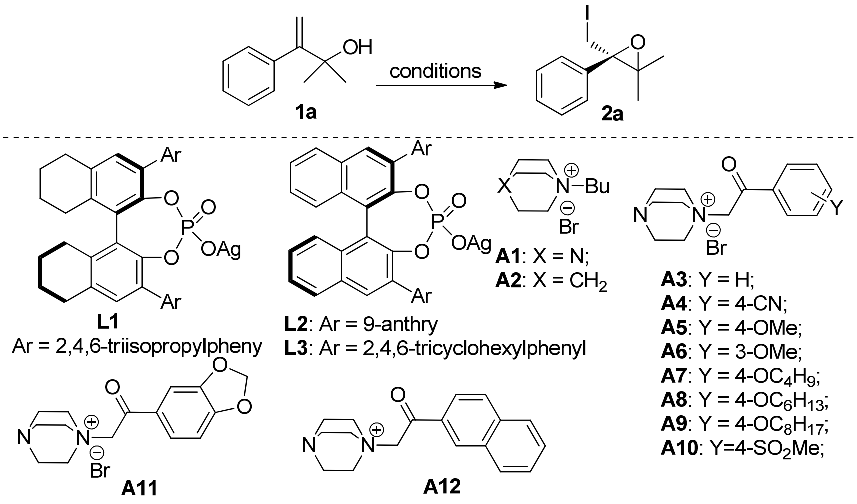

entry	solvent	silver salt (equiv)	additive (equiv)	yield[a] (%)	ee[b] (%)
1	DCM	**L1** (0.1)	**A1** (0.12)	16	30
2	DCM	**L1** (0.1)	**A2** (0.12)	18	19
3	DCM	**L1** (0.1)	**A3** (0.12)	44	77
4	DCM	**L1** (0.1)	**A4** (0.12)	16	69
5	DCM	**L1** (0.1)	**A5** (0.12)	69	86
6	DCM	**L1** (0.1)	**A6** (0.12)	47	80
7	DCM	**L1** (0.1)	**A7** (0.12)	65	91
8	DCM	**L1** (0.1)	**A8** (0.12)	60	92
9	DCM	**L1** (0.1)	**A9** (0.12)	50	91
10	DCM	**L1** (0.1)	**A10** (0.12)	21	43
11	DCM	**L1** (0.1)	**A11** (0.12)	61	88
12	DCM	**L1** (0.1)	**A12** (0.12)	65	81
13	DCM	**L2** (0.1)	**A8** (0.12)	30	40
14	DCM	**L3** (0.1)	**A8** (0.12)	62	67
15	DCM	--	--	trace	ND
16	DCM	**L1** (0.1)	--	trace	ND
17	DCM	--	**A8** (0.12)	trace	ND

Reaction conditions: To a mixture of silver salt **L1** (0.01 mmol), ammonium salt **A** (0.012 mmol) and NIS (0.12 mmol) was added DCM (1 mL) then the reaction mixture was cooled to 0 °C. Allyl alcohol **1a** (0.1 mmol) in 0.5 mL DCM was added dropwise and the reaction was quenched after 40 h. [a]Isolated yield. [b]Determined by HPLC using Chiralpak AD column.

Table S2. Survey of other organocatalysts.

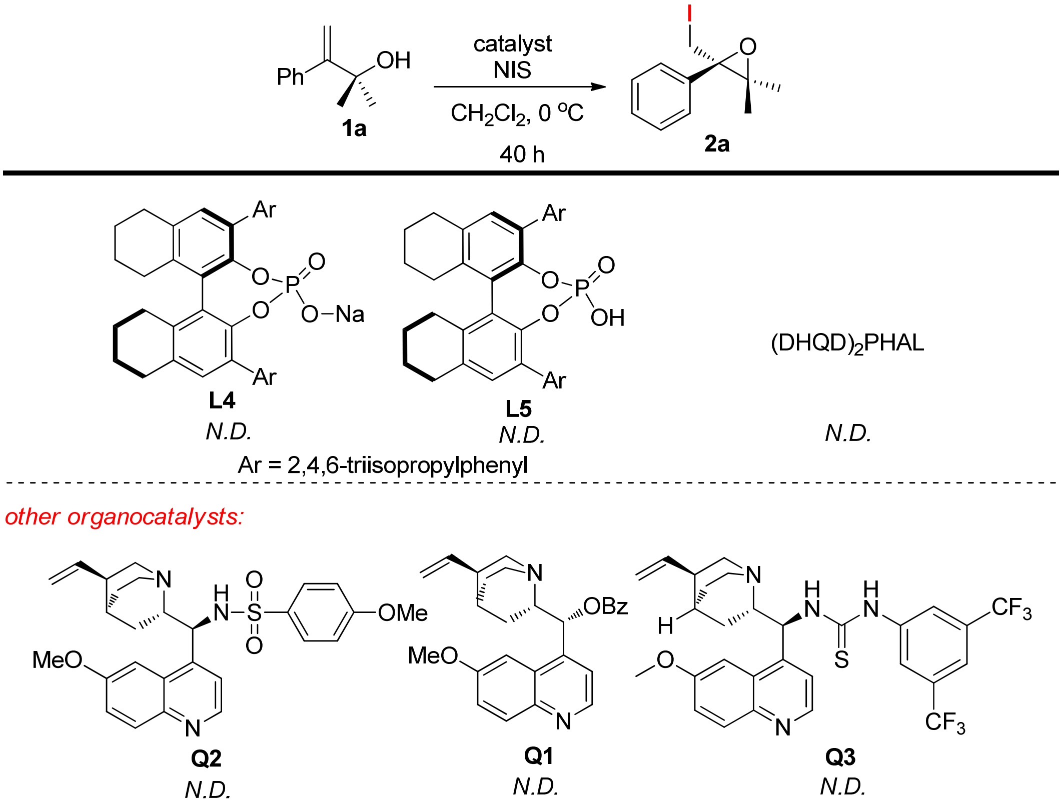

Reaction conditions: To a mixture of catalsyt **L1** (0.01 mmol), NIS (0.12 mmol) was added DCM (1 mL) then the reaction mixture was cooled to 0 °C. Allyl alcohol **1a** (0.1 mmol) in 0.5 mL DCM was added dropwise and the reaction was quenched after 40 h.

Table S3. Screening of halogen sources

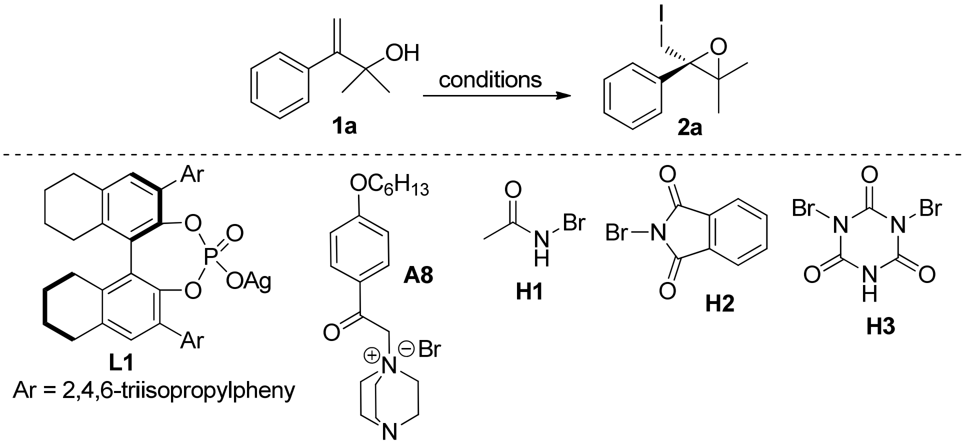

entry	solvent	silver salt (equiv)	additive (equiv)	halogenating reagent	yield[a] (%)	ee[b] (%)
1	DCM	**L1** (0.1)	**A8** (0.12)	NIS	65	91
2	DCM	**L1** (0.1)	**A8** (0.12)	I_2	NR	--
3	DCM	**L1** (0.1)	**A8** (0.12)	NCS	NR	--
4	DCM	**L1** (0.1)	**A8** (0.12)	NBS	15	79
5	DCM	**L1** (0.1)	**A8** (0.12)	**H1**	23	8
6	DCM	**L1** (0.1)	**A8** (0.12)	**H2**	46	31
7	DCM	**L1** (0.1)	**A8** (0.12)	**H3**	68	78

Reaction conditions: To a mixture of silver salt **L1** (0.01 mmol), ammonium salt **A8** (0.012 mmol) and halogenating reagent (0.12 mmol) was added DCM (1 mL) then the reaction mixture was cooled to 0 °C. Allyl alcohol **1a** (0.1 mmol) in 0.5 mL DCM was added dropwise and the reaction was quenched after 40 h. [a]Isolated yield. [b]Determined by HPLC using Chiralpak AD column.

Table S4. Effects of additive and temperature.

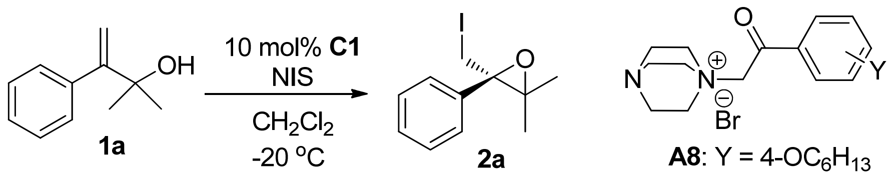

entry	catalyst (equiv)	additive (equiv)	T (°C)	t (h)	yield[a] (%)	ee[b] (%)
1	**C1** (0.1)	--	0	40	42	83
2	**C1** (0.1)	DABCO (0.1)	0	40	45	84
3	**C1** (0.1)	(-)-CSA (0.1)	0	40	38	80
4	**C1** (0.1)	Ph_3P=S (0.1)	0	40	63	90
5	**C1** (0.1)	**A8** (0.1)	0	40	82	92
6	**C1** (0.1)	**A8** (0.1)	-20	107	99	94
8	**C1** (0.1)	**A8** (0.1)	-40	121	83	94

Reaction conditions: To a mixture of silver salt **C1** (0.01 mmol), additive (0.012 mmol) and NIS (0.12 mmol) was added DCM (1 mL) then the reaction mixture was cooled to 0 °C. Allyl alcohol **1a** (0.1 mmol) in 0.5 mL DCM was added dropwise and the reaction was quenched at indicated time. [a]Isolated yield. [b]Determined by HPLC using Chiralpak AD column.

Table S5. Screening of solvents.

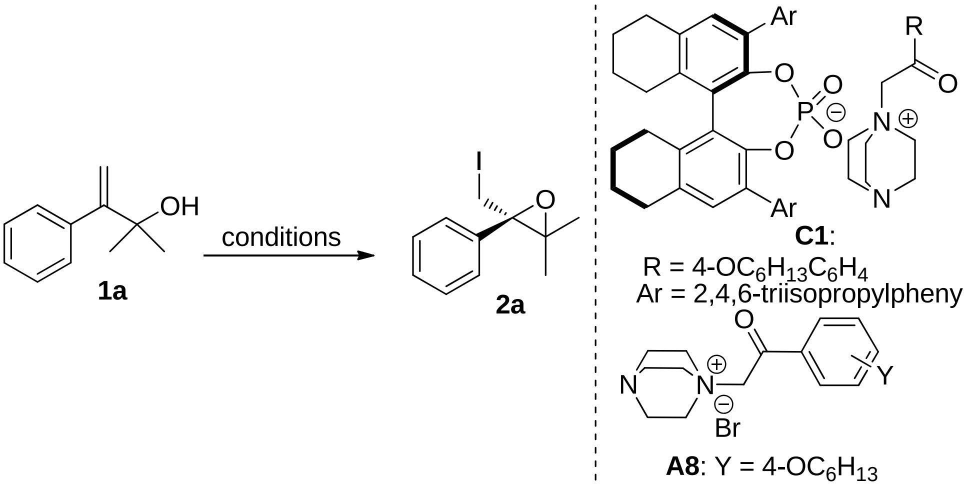

entry	solvent	catalyst (equiv)	additive (equiv)	yield[a] (%)	ee[b] (%)
1	CH_2Cl_2	**C1** (0.1)	**A8** (0.1)	82	92
2	$CHCl_3$	**C1** (0.1)	**A8** (0.1)	62	69
3	1,2-dichoroethane	**C1** (0.1)	**A8** (0.1)	76	19
4	Ethylene dibromide	**C1** (0.1)	**A8** (0.1)	49	55
5	CH_3CN	**C1** (0.1)	**A8** (0.1)	59	50
6	EtOAc	**C1** (0.1)	**A8** (0.1)	31	67
8	THF	**C1** (0.1)	**A8** (0.1)	73	85
9	Hexane	**C1** (0.1)	**A8** (0.1)	59	50

Reaction conditions: To a mixture of silver salt **L1** (0.01 mmol), additive (0.012 mmol) and NIS (0.12 mmol) was added Solvent (1 mL) then the reaction mixture was cooled to 0 °C. Allyl alcohol **1a** (0.1 mmol) in 0.5 mL DCM was added dropwise and the reaction was quenched after 40 h. [a]Isolated yield. [b]Determined by HPLC using Chiralpak AD column.

Proposed transition states for the observed stereoselectivity.

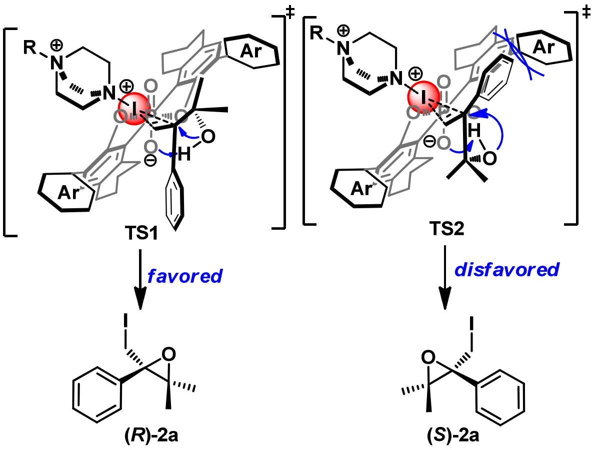

Figure S1. Rationale for the Observed Stereoselectivity.

To explain the stereoselectivity of this reaction, putative transition states are proposed based on previous working model for the chiral anionic phase-transfer catalyst (Figure S1).[1,2] That is, the iodine of NIS may be first transferred to DABCO moiety of the ion-pair organocatalyst, which may be accelerated by ammonium **A8**. Subsequently, alkene exchanges with one of DABCO-derived ammonium to produce cyclization precursor. In the transition state, chiral phosphate functionalizes as Brønsted base for deprotonation of hydroxyl of allyl alcohol and meanwhile is hold tightly with DABCO-derived ammonium by Columbic interaction. Through these cooperative interactions, substrate and NIS are simultaneously activated by the ion-pair catalyst, which leads to a well-defined transition state for the *3-exo* iodo-cycloetherification. As shown in Figure S1, iodonium complexing with *Re* face of alkene in **TS1** suffers less steric repulsion between substrate and phosphate than that in **TS2**, which would favorably produce the observed stereoisomer (*R*)-**2a**.

General procedure for preparation of substrates, ammonium salts and catalyst

3.1 General procedure for the synthesis of 2-Aryl-2-propen-1-ol

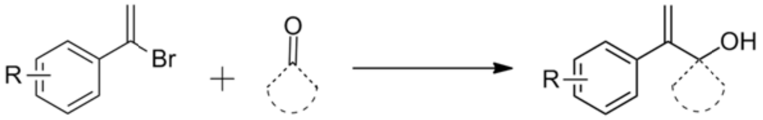

General procedure A[1a]

To a THF solution (20 ml) of alkene (4 mmol) was added *t*-BuLi (8 mmol) at -78 °C. After stirred at this temperature for 1.5 h, the lithium salt was quenched with ketone (4.8 mmol). The mixture stirred for a further 1h, and slowly warmed to rt. Water (10 ml) was then added, and the organic product extracted with Et_2O, and dried over anhydrous Na_2SO_4. The alcohol were purified by flash column chromatography (ethyl acetate/petroleum ether = 1:25, v/v).

General procedure B

A 50 mL two-neck round-bottomed flask equipped with an addition funnel and a condenser was charged with Mg turnings (264 mg, 11 mmol). Anhydrous THF (3 mL) and 1,2-dibromoethane (91 mg, 43 µL, 0.05 mmol) were added via syringe, then the mixture was activated by heat with a hairdryer during which time there were bubbles emerging from the surface of Mg turnings. After stirring at rt for 0.5 min, corresponding alkene (10 mmol) in anhydrous THF (10 mL) was then added dropwise over 0.5 h via the addition funnel, during which time a significant exotherm was observed. The reaction was then placed in an oil bath and heated at reflux for 2h. At which time, ketone (11 mmol) was added. After further stirring for 1.5 h at reflux, saturated NH_4Cl (aq.) (5 mL) was added. The organic layer was extracted with ethyl acetate and washed with brine, separated, dried over Na_2SO_4, filtered and then concentrated. The residue was purified by silica gel column chromatography (ethyl acetate/petroleum ether = 1:25, v/v) to afford the product.

General procedure C[1b]

Into a flame-dried 50 mL round-bottomed flask was added dry THF (15 mL) under a nitrogen atmosphere. After cooling to –78 ºC (acetone/dry-ice bath), *n*-BuLi (8.3 mL, 2.5 M in hexane, 20.5 mmol) and dry CH_3CN (550 µL, 10.5 mmol) were slowly added respectively. After stirring for 20 min, aryl epoxide (10 mmol) was then added. The reaction mixture was gradually warmed up to room temperature overnight and quenched with saturated $NaHCO_3$ (15 mL). After the phase separation, the aqueous layer was extracted with Et_2O. The combined organics were washed with brine, dried over Na_2SO_4, and concentrated under reduced pressure. The crude product was purified by column chromatography (ethyl acetate/petroleum ether = 1:25, v/v).

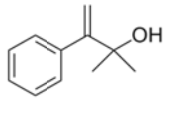

2-Methyl-3-phenyl-but-3-en-2-ol (1a)

1a, a known compound[1b], was prepared following the general procedure A by using acetone and α-bromostyrene as a colorless liquid (1.13 g, 61% yield on 11.4 mmol scale).

Analytical data for **1a:** ^{1}H NMR (500 MHz, $CDCl_3$) δ 7.33-7.27 (m, 5H), 5.46 (d, *J* = 1.0 Hz, 1H), 4.99

(d, J = 1.0 Hz, 1H), 2.12 (brs, 1H), 1.44 (s, 6H); ^{13}C NMR (126 MHz, $CDCl_3$) δ 157.1, 141.6, 128.9, 127.8, 127.0, 112.6, 73.0, 29.7; IR (film) 3388, 915, 703 cm^{-1}; HRMS(EI+) exact mass calcd. for $C_{11}H_{14}O$ requires m/z [M]$^+$: 162.1045. Found m/z 162.1047.

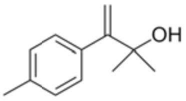

2-Methyl-3-p-tolyl-but-3-en-2-ol (1aa)

1aa, a known compound[1a], was prepared following the general procedure A by using acetone and 1-(1-bromovinyl)-4-methylbenzene as a yellow liquid (454 mg, 60% yield on 4.3 mmol scale).

Analytical data for **1aa:** ^{1}H NMR (500 MHz, $CDCl_3$) δ 7.20 (d, J = 10.0 Hz, 1H), 7.13 (d, J = 10.0 Hz, 2H), 5.41 (d, J = 1.0 Hz, 1H), 4.96 (d, J = 1.0 Hz, 1H), 2.36 (s, 3H), 1.66 (brs, 1H), 1.42 (s, 6H); ^{13}C NMR (126 MHz, $CDCl_3$) δ 157.0, 138.5, 136.7, 128.7, 128.5, 112.3, 73.1, 29.7, 21.1; IR (film) 3393, 2976, 910, 792 cm^{-1}; HRMS(EI+) exact mass calcd. for $C_{12}H_{16}O$ requires m/z 176.1201. Found m/z 176.1202.

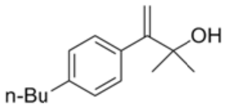

3-(4-Butyl-phenyl)-2-methyl-but-3-en-2-ol (1ab)

1ab was prepared following the general procedure A by using acetone and 1-(1-bromovinyl)-4-butylbenzene as a yellow liquid (417 mg, 49% yield on 3.9 mmol scale).

Analytical data for **1ab:** ^{1}H NMR (500 MHz, $CDCl_3$) δ 7.21 (d, J = 7.5 Hz, 2 H), 7.13 (d, J = 7.5 Hz, 2 H), 5.10 (d, J = 1.5 Hz, 1H), 4.96 (d, J = 1.5 Hz, 1H), 2.61 (t, J = 7.5 Hz, 2 H), 1.66 (brs, 1 H), 1. 65-1. 58 (m, 2 H), 1.41 (s, 6 H), 1. 40-1.34 (m, 2 H), 0.94 (t, J = 7.5 Hz, 3 H); ^{13}C NMR (126 MHz, $CDCl_3$) δ 157.0, 141.7, 138.6, 128.7, 127.8, 112.3, 73.1, 35.3, 33.6, 29.7, 22.4, 14.0; IR (film) 3392, 2929, 1177, 913, 839 cm^{-1}; HRMS(ESI+) exact mass calcd for $C_{15}H_{22}Na_1O_1$ requires m/z [M+Na]$^+$: 241.1563. Found m/z 241.1553.

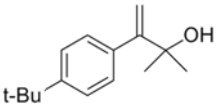

3-(4-tert-Butyl-phenyl)-2-methyl-but-3-en-2-ol (1ac)

1ac was prepared following the general procedure B by using acetone and 1-(1-bromovinyl)-4-(tert-butyl)benzene as a pale yellow solid (412 mg, 42% yield on 4.5mmol scale).

Analytical data for **1ac:** ^{1}H NMR (500 MHz, $CDCl_3$) δ 7.32 (d, J = 7.5 Hz, 2H), 7.23 (d, J = 7.5 Hz, 2H), 5.39 (d, J = 1.5 Hz, 1H), 4.97 (d, J = 1.5 Hz, 1H), 1.63 (brs, 1H), 1.42 (s, 6H), 1.33 (s, 9H); ^{13}C NMR (126 MHz, $CDCl_3$) δ 156.9, 149.9, 138.3, 128.5, 124.7, 112.3, 73.1, 34.5, 31.4, 29.7; IR (film) 3398, 1508, 913, 838 cm^{-1}; HRMS(ESI+) exact mass calcd for $C_{15}H_{22}Na_1O_1$ requires m/z [M+Na]$^+$: 241.1563. Found m/z 241.1557.

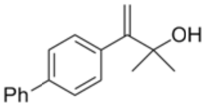

3-Biphenyl-4-yl-2-methyl-but-3-en-2-ol (1ad)

1ad was prepared following the general procedure B by using acetone and 4-(1-bromovinyl)-1,1'-biphenyl as a pale yellow solid (324 mg, 34% yield on 4.0 mmol scale).

Analytical data for **1ad:** ^{1}H NMR (500 MHz, $CDCl_3$) δ 7.62 (d, J = 8.0 Hz, 2H), 7.56 (d, J = 8.0 Hz,

2H), 7.46 (t, *J* = 7.5 Hz, 2H), 7.41 (d, *J* = 8.0 Hz, 2H), 7.36 (t, *J* = 7.5 Hz, 1H), 5.47 (s, 1H), 5.05 (s, 1H), 1.70 (brs, 1H), 1.47 (s, 6H); ^{13}C NMR (126 MHz, $CDCl_3$) δ 156.7, 140.8, 140.5, 139.9, 129.3, 128.79, 128.78, 127.3, 127.0, 126.5, 112.7, 73.1, 29.8; IR (film) 3394, 917, 851 cm^{-1}; HRMS(EI+) exact mass calcd for $C_{17}H_{18}O$ requires m/z [M]$^+$: 238.1358. Found m/z 238.1360.

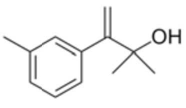

2-Methyl-3-m-tolyl-but-3-en-2-ol (1ae)

1ae was prepared following the general procedure B by using acetone and 1-(1-bromovinyl)-3-methylbenzene as a pale yellow liquid (221 mg, 37% yield on 3.4 mmol scale).

Analytical data for **1ae:** ^{1}H NMR (500 MHz, $CDCl_3$) δ 7.21 (t, *J* = 7.5 Hz, 1H), 7.12-7.10 (m, 3H), 5.42 (s, 1H), 4.96 (s, 1H), 2.37 (s, 3H), 1.76 (brs, 1H), 1.43 (s, 6H); ^{13}C NMR (126 MHz, $CDCl_3$) δ 157.2, 141.4, 137.3, 129.6, 127.8, 127.7, 125.9, 112.3, 73.0, 29.7, 21.5; IR (film) 3387, 911, 837 cm^{-1}; HRMS(EI+) exact mass calcd for $C_{12}H_{16}O$ requires m/z [M]$^+$:176.1201. Found m/z: 176.1202.

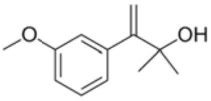

3-(3-Methoxy-phenyl)-2-methyl-but-3-en-2-ol (1af)

1af, a known compound[1a], was prepared following the general procedure A by using acetone and 1-(1-bromovinyl)-3-methoxybenzene as a pale yellow liquid (438 mg, 57% yield on 4.0 mmol scale).

Analytical data for **1af:** ^{1}H NMR (500 MHz, $CDCl_3$) δ 7.21-7.24 (t, *J* = 8.0 Hz, 1H), 6.83-6.89 (m, 3H), 5.42 (s, 1H) , 4.98 (s, 1H), 3.81 (s, 3H), 1.68 (brs, 1H), 1.42 (s, 6H); ^{13}C NMR (500 MHz, $CDCl_3$) δ 156.9, 156.0, 142.9, 128.8, 121.3, 114.8, 112.5, 112.3, 73.0, 55.2, 29.7; IR (film) 3402, 911, 832 cm^{-1}; HRMS (ESI+) exact mass calcd for $C_{12}H_{16}NaO_2$ requires m/z [M+Na]$^+$: 215.1043. Found m/z 215.1042.

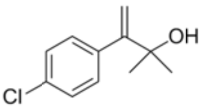

3-(4-Chloro-phenyl)-2-methyl-but-3-en-2-ol (1ag)

1ag, a known compound[1a], was prepared following the general procedure B by using acetone and 1-(1-bromovinyl)-4-chlorobenzene as a pale yellow liquid (227 mg, 36% yield on 3.2 mmol scale).

Analytical data for **1ag:** ^{1}H NMR (500 MHz, $CDCl_3$) δ 7.129-7.24 (m, 4H), 5.43 (d, *J* = 1.0 Hz, 1H), 4.97 (d, *J* = 1.0 Hz, 1H), 1.64 (brs, 1H), 1.40 (s, 6H); ^{13}C NMR (126 MHz, $CDCl_3$) δ 155.8, 139.9, 133.0, 130.2, 127.9, 113.2, 72.9, 29.6; IR (film) 3392, 2978, 1560, 920, 790 cm^{-1}; HRMS(EI+) exact mass calcd for $C_{11}H_{13}OCl$ requires m/z [M]$^+$: 196.0655. Found m/z 196.0653.

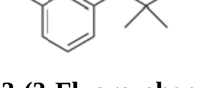

3-(3-Fluoro-phenyl)-2-methyl-but-3-en-2-ol (1ah)

1ah was prepared following the general procedure B by using acetone and 1-(1-bromovinyl)-3-fluorobenzene as a pale yellow liquid (173 mg, 26% yield on 3.7 mmol).

Analytical data for **1ah:** ^{1}H NMR (500 MHz, $CDCl_3$) δ^{1}H NMR (500 MHz, $CDCl_3$) δ 7.27 (q, *J* = 8.5 Hz, 1H), 7.11-7.04 (m, 2H), 6.98 (td, *J* = 8.5, 2.5 Hz, 1H), 5.44 (s, 1H), 5.00 (s, 1H), 1.65 (brs, 1H), 1.41 (s, 6H); ^{13}C NMR (126 MHz, $CDCl_3$) δ 162.1 (d, *J* = 247.0 Hz), 155.8, 143.7 (d, *J* = 8.8 Hz),

129.2 (d, J = 8.8 Hz), 124.5 (d, J = 2.5 Hz), 115.9 (d, J = 21.4 Hz), 113.9 (d, J = 21.4Hz), 113.2, 72.9, 29.6; ^{19}F NMR (376 MHz, $CDCl_3$) δ -113.84--113.90 (m); IR (film) 3396, 2961, 1223, 841 cm^{-1}; HRMS(EI+) exact mass calcd for $C_{11}H_{13}FO$ requires m/z $[M]^+$:180.0950. Found m/z: 180.0949.

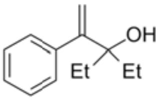

3-ethyl-2-phenylpent-1-en-3-ol (1b)

1b, a known compound[1c], was prepared following the general procedure A by using 3-pentanone and α-bromostyrene as a colorless liquid (504 mg, 53% yield on 5.0 mmol scale).

Analytical data for **1b:** ^{1}H NMR (500 MHz, $CDCl_3$) δ 7.32-7. 29 (m, 3H), 7.27-7.25 (m, 2H), 5.39 (d, J = 1.0 Hz, 1H), 5.14 (d, J = 1.0 Hz, 1H), 1.72-1.67 (m, 2H), 1.66-1.60 (m, 2H)), 1.50 (brs, 1H), 0.95 (t, J = 7.5 Hz, 6H); ^{13}C NMR (126 MHz, $CDCl_3$) δ 153.7, 141.8, 128.4, 127.8, 127.0, 115.4, 78.1, 31.9, 7.8; IR (film) 3407, 906, 832 cm^{-1}; HRMS(EI+) exact mass calcd for $C_{13}H_{18}O$ requires m/z $[M+Na]^+$: 190.1358. Found m/z 190.1354.

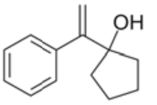

1-(1-Phenyl-vinyl)-cyclopentanol (1c)

1c, a known compound[1d], was prepared following the general procedure **B** by using cyclopentanone and α-bromostyrene as a colorless liquid (4.29 g. 76% yield on 30 mmol scale).

Analytical data for **1c:** ^{1}H NMR (500 MHz, $CDCl_3$) δ 7.41 (d, J = 5.0 Hz, 2H), 7.34-7.28 (m, 3H), 5.45 (s, 1H), 5.09 (s, 1H), 1.92-1.85 (m, 4H), 1.82-1.79 (m, 2H), 1.74-1.66 (m, 3H); ^{13}C NMR (126 MHz, $CDCl_3$) δ 154.9, 141.8, 128.5, 127.8, 127.1, 113.2, 84.1, 39.2, 23.3; IR (film) 3388, 915, 703 cm^{-1}; HRMS(ESI+) exact mass calcd for $C_{15}H_{20}NaO$ requires m/z $[M+Na]^+$: 239.1406. Found m/z 239.1398.

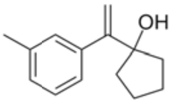

1-(1-(m-tolyl)vinyl)cyclopentanol (1ca)

1ca was prepared following the general procedure **B** by using cyclopentanone and 1-(1-bromovinyl)-3-methylbenzene as a yellow liquid (286 mg, 41% yield on 3.2 mmol scale).

Analytical data for **1ca:** ^{1}H NMR (500 MHz, $CDCl_3$) δ 7.22-7.17 (m, 3H), 7.10 (d, J = 10.0 Hz, 1H), 5.41 (d, J = 1.0 Hz, 1H), 5.05 (d, J = 1.0 Hz, 1H), 2.36 (s, 3H), 1.90-1.84 (m, 4H), 1.80-1.78 (m, 2H), 1.70-1.68 (m, 2H), 1.47 (brs, 1H); ^{13}C NMR (126 MHz, $CDCl_3$) δ 155.0, 141.6, 137.4, 129.2, 127.8, 127.7, 125.5, 113.0, 84.1, 39.2, 23.28, 23.27, 21.5; IR (film) 3420, 1601, 949, 911 cm^{-1}; HRMS(EI+) exact mass calcd for $C_{14}H_{18}O$ requires m/z $[M]^+$: 202.1358. Found m/z 202.1359.

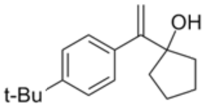

1-(1-(4-(tert-butyl)phenyl)vinyl)cyclopentanol (1cb)

1cb was prepared following the general procedure **B** by using cyclopentanone and 1-(1-bromovinyl)-4-(tert-butyl)benzene as a colorless liquid (708 mg, 63% yield on 4.6 mmol scale).

Analytical data for **1cb:** ^{1}H NMR (500 MHz, $CDCl_3$) δ 7.35 (d, J = 1.0 Hz, 4H), 5.42 (d, J = 1.0 Hz, 1H), 5.09 (s, J = 1.0 Hz, 1H), 1.93-1.87 (m, 4H), 1.84-1.81 (m, 2H), 1.72-1.70 (m, 2H), 1.36 (s, 9H); ^{13}C NMR (126 MHz, $CDCl_3$) δ 154.8, 149.9, 138.7, 128.2, 124.7, 112.9, 84.2, 39.3, 34.5, 31.4, 23.4; IR

(film) 3458, 999, 842 cm^{-1}; HRMS(EI+) exact mass calcd for $C_{17}H_{24}O$ requires m/z [M]$^+$: 244.1827. Found m/z 244.1830.

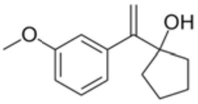

1-(1-(3-methoxyphenyl)vinyl)cyclopentanol (1cc)

1cc was prepared following the general procedure **B** by using cyclopentanone and 1-(1-bromovinyl)-3-methylbenzene as a yellow liquid (465 mg, 41% yield on 5.2 mmol scale).

Analytical data for **1cc:** ^{1}H NMR (500 MHz, $CDCl_3$) δ 7.24-7.21 (m, 1H), 6.97-6.94 (m, 2H), 6.83 (d, *J* = 10.0 Hz, 1H), 5.42 (s, 1H), 5.08 (s, 1H), 3.81 (s, 3H), 1.89-1.84 (m, 4H), 1.80-1.77 (m, 2H), 1.70-1.65 (m, 2H), 1.49 (brs, 1H); ^{13}C NMR (126 MHz, $CDCl_3$) δ 159.0, 154.7, 143.2, 128.8, 121.0, 114.4, 113.2, 112.4, 84.0, 55.2, 39.2, 23.3; IR (film) 3420, 1601, 949, 911 cm^{-1}; HRMS(EI+) exact mass calcd for $C_{14}H_{18}O_2$ requires m/z [M]$^+$: 218.1307. Found m/z 218.1309.

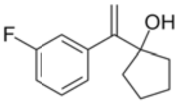

1-(1-(3-fluorophenyl)vinyl)cyclopentanol (1cd)

1cd was prepared following the general procedure **B** by using cyclopentanone and 1-(1-bromovinyl)-3-fluorobenzene as a yellow liquid (195 mg, 27% yield on 3.5 mmol scale).

Analytical data for **1cd:** ^{1}H NMR (500 MHz, $CDCl_3$) δ 7.28-7.23 (m, 1H), 7.17-7.12 (m, 2H), 6.70 (t, *J* = 12.5 Hz, 1H), 5.43 (s, 1H), 5.09 (s, 1H), 1.89-1.78 (m, 6H), 1.74-1.68 (m, 2H), 1.42 (brs, 1H); ^{13}C NMR (126 MHz, $CDCl_3$) δ 162.34 (d, *J* = 244.5 Hz), 153.7 (d, *J* = 1.9 Hz), 143.9 (d, *J* = 7.6 Hz), 129.2 (d, *J* = 8.5 Hz), 124.1 (d, *J* = 2.9 Hz), 115.5 (d, *J* = 20.9 Hz), 114.0 (d, *J* = 6.6 Hz), 113.8, 83.9, 39.1, 23.2; ^{19}F NMR (376MHz, $CDCl_3$) δ -113.78--113.85 (m); IR (film) 3447, 1508, 836 cm^{-1}; HRMS(EI+) exact mass calcd for $C_{13}H_{15}OF$ requires m/z [M]$^+$: 206.1107. Found m/z 206.1103.

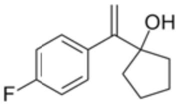

1-(1-(4-fluorophenyl)vinyl)cyclopentanol (1ce)

1ce was prepared following the general procedure **B** by using cyclopentanone and 1-(1-bromovinyl)-4-fluorobenzene as a yellow liquid (286 mg, 42% yield on 3.3 mmol scale).

Analytical data for **1ce:** ^{1}H NMR (500 MHz, $CDCl_3$) δ 7.39-7.35 (m, 2H), 7.01-6.96 (m, 2H), 5.41 (d, *J* = 1.0 Hz 1H), 5.06 (d, *J* = 1.0 Hz 1H), 1.90-1.74 (m, 6H), 1.71-1.67 (m, 2H), 1.50 (brs, 1H); ^{13}C NMR (126 MHz, $CDCl_3$) δ 162.1 (d, *J* = 244.7 Hz), 153.8, 137.6 (d, *J* = 3.8 Hz), 130.0 (d, *J* = 7.6 Hz), 114.6 (d, *J* = 9.2 Hz), 113.5, 84.1, 39.1, 23.2; ^{19}F NMR (376MHz, $CDCl_3$) δ -115.78--115.84; IR (film) 3397, 915, 841 cm^{-1}; HRMS(EI+) exact mass calcd for $C_{13}H_{15}OF$ requires m/z [M]$^+$: 206.1107. Found m/z 206.1104.

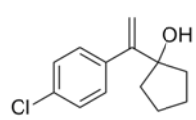

1-(1-(4-chlorophenyl)vinyl)cyclopentanol (1cf)

1cf was prepared following the general procedure **B** by using cyclopentanone and 1-(1-bromovinyl)-4-chlorobenzene as a yellow liquid (248 mg, 31% yield on 3.6 mmol scale).

Analytical data for **1cf:** ^{1}H NMR (500 MHz, $CDCl_3$) δ 7.34-7.32 (m, 2H), 7.27-7.25 (m, 2H), 5.41 (d, *J*

= 1.0 Hz, 1H), 5.06 (d, *J* = 1.0 Hz, 1H), 1.88-1.74 (m, 6H), 1.68-1.65 (m, 2H), 1.50 (brs, 1H); ^{13}C NMR (126 MHz, $CDCl_3$) δ 153.7, 140.2, 133.0, 129.8, 128.0, 113.8, 84.0, 39.1, 23.2; IR (film) 3398, 952, 861 cm^{-1}; HRMS(EI+) exact mass calcd for $C_{13}H_{15}OCl$ requires m/z $[M]^+$: 222.0811. Found m/z 222.0812.

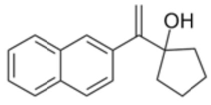

1-(1-(naphthalen-2-yl)vinyl)cyclopentanol (1cg)

1cg was prepared following the general procedure **B** by using cycloheptanone and 2-(1-bromovinyl) naphthalene as a yellow liquid (297 mg, 39% yield on 3.2 mmol scale).

Analytical data for **1cg:** ^1H NMR (500 MHz, $CDCl_3$) δ 7.86 (s, 1H), 7.83 (d, *J* = 7.5 Hz, 2H), 7.79 (d, *J* = 7.5 Hz, 1H), 7.56 (d, *J* = 10.0 Hz, 1H), 7.49-7.47 (m, 2H), 5.52 (s, 1H), 5.20 (s, 1H), 1.94-1.84 (m, 6H), 1.73-1.70 (m, 2H), 1.64 (brs, 1H); ^{13}C NMR (126 MHz, $CDCl_3$) δ 154.9, 139.3, 133.1, 132.5, 128.1, 127.5, 127.2, 127.07, 127.05, 126.06, 125.8, 113.7, 84.3, 39.3, 23.3; IR (film) 3435, 951, 899 cm^{-1}; HRMS (EI+) exact mass calcd for $C_{17}H_{18}O$ requires m/z $[M]^+$: 238.1358. Found m/z 238.1362.

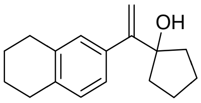

1-(1-(5,6,7,8-tetrahydronaphthalen-2-yl)vinyl)cyclopentanol (1ch)

1ch was prepared following the general procedure **B** by using cycloheptanone and 6-(1-bromovinyl)-1,2,3,4-tetrahydronaphthalene as a yellow liquid (387 mg, 47% yield on 3.4 mmol scale).

Analytical data for **1ch:** ^1H NMR (500 MHz, $CDCl_3$) δ 7.09 (d, *J* = 7.5 Hz, 2H), 7.07 (s, 1.0H)7.01 (d, *J* = 7.5 Hz, 1H), 5.38 (s, 1H), 5.04 (s, 1H), 2.76, (m, 4H), 1.91-1.84 (m, 4H), 1.81-1.78 (m, 6H), 1.71-1.66 (m, 2H), 1.62 (brs, 1H); ^{13}C NMR (126 MHz, $CDCl_3$) δ 154.9, 138.8, 136.5, 136.0, 129.1, 128.5, 125.6, 112.7, 84.1, 39.3, 29.5, 29.1, 23.3, 23.2; IR (film) 3425, 952, 879 cm^{-1}; HRMS (EI+) exact mass calcd for $C_{17}H_{22}O$ requires m/z $[M]^+$: 242.1671. Found m/z 242.1672.

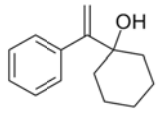

1-(1-Phenyl-vinyl)-cyclohexanol (1d)

1d, a known compound[1e], was prepared following the general procedure **B** by using cyclohexanone and α-bromostyrene as a pale yellow liquid (2.10 g, 52% yield on 20.0 mmol scale).

Analytical data for **1d:** ^1H NMR (500 MHz, $CDCl_3$) δ 7.31-7.27 (m, 5H), 5.43 (d, *J* = 5.0 Hz, 1H), 5.02 (d, *J* = 5.0 Hz, 1H), 1.69-1.56 (m, 8H), 1.53-1.50 (m, 2H), 1.44 (brs, 1H); ^{13}C NMR (126 MHz, $CDCl_3$) δ 156.7, 141.6, 129.0, 127.7, 126.9, 113.4, 73.6, 36.7, 25.5, 22.1; IR (film) 3388, 915, 703 cm^{-1}; HRMS(EI+) exact mass calcd for $C_{14}H_{18}O$ requires m/z $[M]^+$: 202.1358. Found m/z 202.1354.

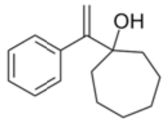

1-(1-Phenyl-vinyl)-cycloheptanol (1e)

1e was prepared following the general procedure **B** by using cycloheptanone and α-bromostyrene in 56% yield as a yellow liquid Yellow liquid (1.22 g, 56% yield on 1.0 mmol scale).

Analytical data for **1e:** ^1H NMR (500 MHz, $CDCl_3$) δ 7.32-7.29 (m, 5H), 5.40 (d, *J* = 1.0 Hz, 1H), 4.97 (d, *J* = 1.0 Hz, 1H), 1.99-1.94 (m, 2H), 1.99-1.94 (m, 2H), 1.79-1.74 (m, 2H), 1.67-1.61 (m, 4H),

1.56 (brs, 1H), 1.50-1.47 (m, 2H), 1.44-1.40 (m, 2H); ^{13}C NMR (126 MHz, $CDCl_3$) δ 157.6, 141.8, 129.1, 127.7, 126.9, 112.7, 77.5, 40.6, 29.2, 22.4; IR (film) 3421, 973, 702 cm^{-1}; HRMS(EI+) exact mass calcd for $C_{15}H_{20}O$ requires m/z $[M]^+$: 216.1514. Found m/z 216.1512.

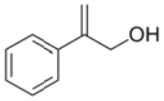

2-phenylprop-2-en-1-ol (1f)

1f was prepared following the general procedure **C** by using 2-phenyloxirane as a yellow liquid (684 mg, 51% yield on 10 mmol scale).

Analytical data for **1f:** ^{1}H NMR (500 MHz, $CDCl_3$) δ 7.45 (d, *J* = 8.0 Hz, 2H), 7.36 (t, *J* = 8.0 Hz, 2H), 7.31 (t, *J* = 8.0 Hz, 1H), 5.48 (s, 1H), 5.36 (s, 1H), 4.55 (s, 2H),1.79 (brs, 1H); ^{13}C NMR (126 MHz, $CDCl_3$) δ 147.3, 138.5, 128.5, 127.9, 126.1, 112.6, 65.0; IR (film) 3367, 961, 715 cm^{-1}; HRMS(EI+) exact mass calcd for $C_9H_{10}O$ requires m/z $[M]^+$: 134.0732 Found m/z 134.0729.

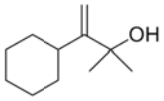

3-cyclohexyl-2-methylbut-3-en-2-ol (1g)

1g, a known compound[1a], was prepared following the general procedure A by using acetone and (1-bromovinyl)cyclohexane as a colorless liquid (309 mg, 46% yield on 4.0 mmol scale).

Analytical data for **1g:** ^{1}H NMR (500 MHz, $CDCl_3$) δ 5.11 (s, 1H), 4.80 (s, 1H), 2.01 (t, *J* = 11.5 Hz, 1H), 1.75-1.67(m, 5H), 1.55 (brs, 1H), 1.33 (s, 6H), 1.30-1.18 (m, 5H); ^{13}C NMR (126 MHz, $cdcl_3$) δ 162.2, 105.7, 73.8, 39.7, 35.5, 28.8, 27.1, 26.2; IR (film) 3401, 2874, 881 cm^{-1}; HRMS(EI+) exact mass calcd for $C_{11}H_{20}O$ requires m/z $[M]^+$: 148.1514. Found m/z 148.1510.

3.2 Synthesis of ammonium salt

General pocedure D

To a solution of DABCO (224 mg, 2.0 mmol) in 10 ml EA was added halogenated alkanes (2.0 mmol). The mixture was stirred at rt for 3 hours, filtrated then dried under reduced pressure getting the analytically pure quaternary ammonium salt.

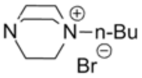

1-butyl-1,4-diazabicyclo[2.2.2]octan-1-ium bromide (A1)

Analytical data for: ^{1}H NMR (400 MHz, D_2O) δ 3.39 (t, *J* = 8.0 Hz, 6H), 3.27-3.23 (m, 2H), 3.18 (t, *J* = 8.0 Hz, 6H), 1.77-1.69 (m, 2H), 1.41-1.32 (m, 2H), 0.94 (t, *J* = 7.5 Hz, 3H); ^{13}C NMR (101 MHz, D_2O) δ 64.39, 64.36, 64.32, 51.98, 51.94, 51.91, 44.1, 23.1, 19.2, 12.7; HRMS (ESI+) exact mass calcd for $C_{10}H_{21}N_2$ requires m/z $[M-Br]^+$: 169.1699. Found m/z 169.1697

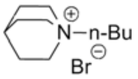

1-butylquinuclidin-1-ium bromide (A2)

Analytical data for **A2**: ^{1}H NMR (400 MHz, D_2O) δ 3.37 (t, *J* = 8.0 Hz, 6H), 3.11-3.07 (m, 2H), 2.18-2.15 (m, 1H), 2.10-1.92 (m, 6H), 1.72-1.66 (m, 2H), 1.36-1.31 (m, 2H), 0.92 (t, *J* = 8.0 Hz, 3H); ^{13}C NMR (101 MHz, D_2O) δ 64.08, 64.04, 64.01, 54.4, 23.37, 23.27, 19.2, 19.03, 18.98, 18.98, 12.7; HRMS (ESI+) exact mass calcd for $C_{11}H_{22}N$ requires m/z $[M-Br]^+$: 168.1747. Found m/z 168.1748.

Note: proton and carbon peak of NCH_2CO *of **A3-A12** are not found in the NMR spectra due to the rapid exchange with D_2O. Those two peaks could be detected by using $CDCl_3$ as solvent as exemplified by the spectra of **A8** in $CDCl_3$.*

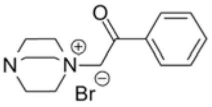

1-(2-oxo-2-phenylethyl)-1,4-diazabicyclo[2.2.2]octan-1-ium bromide (A3)

Analytical data for **A3**: ^{1}H NMR (500 MHz, D_2O) δ 7.99 (dd, *J* = 10.0, 1.0 Hz, 2H), 7.78 (dt, *J* = 10.0, 1.0 Hz, 1H), 7.62 (t, *J* = 10.0 Hz, 2H), 3.81 (t, *J* = 7.5 Hz, 6H), 3.32 (t, *J* = 7.5 Hz, 6H); ^{13}C NMR (126 MHz, D_2O) δ 191.7, 135.2, 134.1, 129.1, 127.9, 52.9, 44.1. HRMS (ESI+) exact mass calcd for $C_{14}H_{19}N_2O$ requires m/z [M-Br]$^+$: 231.1492. Found m/z 231.1490

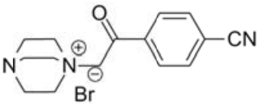

1-(2-(4-cyanphenyl)-2-oxo ethyl)-1,4-diazabicyclo[2.2.2]octan-1-ium bromide (A4)

Analytical data for **A4**: ^{1}H NMR (500 MHz, D_2O) δ 8.12 (dd, *J* = 7.5, 3.0 Hz, 2H), 7.98 (dd, *J* = 7.5, 3.0 Hz, 2H), 3.82 (t, *J* = 7.5 Hz, 6H), 3.33 (t, *J* = 7.5 Hz, 6H); ^{13}C NMR (126 MHz, D_2O) δ 190.5, 137.3, 133.1, 128.2, 118.3, 116.7, 52.9, 44.0; HRMS (ESI+) exact mass calcd for $C_{15}H_{18}N_3O$ requires m/z [M-Br]$^+$: 256.1444. Found m/z 256.1447.

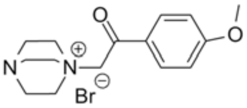

1-(2-(4-methoxyphenyl)-2-oxoethyl)-1,4-diazabicyclo[2.2.2]octan-1-ium bromide (A5)

Analytical data for **A5**: ^{1}H NMR (500 MHz, D_2O) δ 7.99 (d, *J* = 10.0 Hz, 2H), 7.13 (d, *J* = 10.0 Hz, 2H), 3.94 (s, 3H), 3.80 (t, *J* = 7.5 Hz, 6H), 3.31 (t, *J* = 7.5 Hz, 6H); ^{13}C NMR (101 MHz, D_2O) δ 189.8, 164.5, 130.5, 127.1, 114.3, 55.6, 52.8, 44.0; HRMS (ESI+) exact mass calcd for $C_{15}H_{20}N_2O_2$ requires m/z [M-Br]$^+$: 260.1525. Found m/z 260.1523.

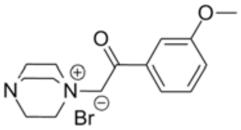

1-(2-(3-methoxyphenyl)-2-oxoethyl)-1,4-diazabicyclo[2.2.2]octan-1-ium bromide (A6)

Analytical data for **A6**: ^{1}H NMR (400 MHz, D_2O) δ 7.55 (d, *J* = 8.0, 1H), 7.50 (t, *J* = 8.0, 1H), 7.46 (s, 1H), 7.31 (dd, *J* = 8.0, 2.0 Hz, 1H), 3.86 (s, 3H), 3.77 (t, *J* = 8.0 Hz, 6H), 3.28 (t, *J* = 8.0 Hz, 6H); ^{13}C NMR (101 MHz, D_2O) δ 191.2, 159.2, 135.4, 130.3, 121.1, 120.8, 112.4, 55.5, 52.8, 44.0; HRMS (ESI+) exact mass calcd for $C_{15}H_{20}N_2O_2$ requires m/z [M-Br]$^+$: 260.1525. Found m/z 260.1524.

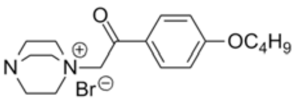

1-(2-(4-butoxyphenyl)-2-oxoethyl)-1,4-diazabicyclo[2.2.2]octan-1-ium bromide (A7)

Analytical data for **A7**: ^{1}H NMR (500 MHz, D_2O) δ 7.97 (d, *J* = 10.0 Hz, 2H), 7.11 (d, *J* = 10.0 Hz, 2H), 4.18 (t, *J* = 10.0 Hz, 2H), 3.79 (t, *J* = 7.5 Hz, 6H), 3.31 (t, *J* = 7.5 Hz, 6H), 1.82-1.76 (m, 2H), 1.50-1.45 (m, 2H), 0.96 (t, *J* = 7.5 Hz, 3H); ^{13}C NMR (126 MHz, D_2O) δ 189.9, 164.1, 130.6, 127.1, 114.9, 68.7, 52.9, 44.1, 30.3, 18.5, 13.0; HRMS (ESI+) exact mass calcd for $C_{18}H_{27}N_2O_2$ requires m/z

[M-Br]$^+$: 303.2067. Found m/z 303.2068.

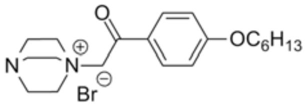

1-(2-(4-(hexyloxy)phenyl)-2-oxoethyl)-1,4-diazabicyclo[2.2.2]octan-1-ium bromide (A8)

Analytical data for **A8**: ^{1}H NMR (500 MHz, D_2O) δ 7.88 (d, *J* = 10.0 Hz, 2H), 6.84 (d, *J* = 10.0 Hz, 2H), 3.88 (m, 2H), 3.75 (m, 6H), 3.23 (m, 6H), 1.63 (m, 2H), 1.36-1.30 (m, 6H), 0.92 (t, *J* = 5.0 Hz, 3H); ^{13}C NMR (101 MHz, D_2O) δ 189.4, 163.8, 130.7, 127.0, 114.5, 68.4, 52.7, 44.0, 31.5, 28.7, 25.4, 22.4, 13.7; For comparison, NMR spectra were also measured by using $CDCl_3$ as solvent to find signals of *NCH_2CO*. H NMR (500 MHz, $CDCl_3$) δ 8.00 (d, *J* = 8.5 Hz, 3H), 6.84 (d, *J* = 8.5 Hz, 2H), *5.53 (s, 2H, NCH_2CO)*, 4.03 (s, 6H), 3.91 (t, *J* = 6.0 Hz, 2H), 3.22 (s, 6H), 1.80-1.67 (m, 2H), 1.46-1.36 (m, 2H), 1.37-1.26 (m, 4H), 0.93-0.85 (m, 3H).; ^{13}C NMR (101 MHz, $CDCl_3$) δ 189.3, 164.5, 131.0, 127.0, 114.7, 68.5, *65.3 (NCH_2CO)*, 52.8, 45.2, 31.5, 29.0, 25.6, 22.5, 14.00; HRMS (ESI+) exact mass calcd for $C_{20}H_{30}N_2O_2$ requires m/z [M-Br]$^+$: 330.2307. Found m/z 330.2307.

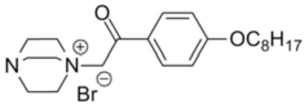

1-(2-(4-(octyloxy)phenyl)-2-oxoethyl)-1,4-diazabicyclo[2.2.2]octan-1-ium bromide (A9)

Analytical data for **A9**: ^{1}H NMR (500 MHz, D_2O) δ 7.87 (d, *J* = 7.5 Hz, 2H), 6.81 (d, *J* = 7.5 Hz, 2H), 3.84 (m, 2H), 3.74 (m, 6H), 3.20 (m, 6H), 1.61 (m, 2H), 1.41-1.23 (m, 10H), 0.95 (t, *J* = 7.5 Hz, 3H); ^{13}C NMR (101 MHz, D_2O) δ 189.3, 163.8, 130.8, 127.0, 114.4, 68.3, 52.7, 44.0, 31.9, 29.5, 29.3, 29.0, 25.9, 22.6, 13.9; HRMS (ESI+) exact mass calcd for $C_{22}H_{35}N_2O_2$ requires m/z [M-Br]$^+$: 359.2693. Found m/z 359.2695.

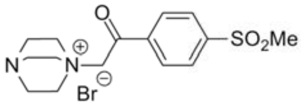

1-(2-(4-(methylsulfonyl)phenyl)-2-oxoethyl)-1,4-diazabicyclo[2.2.2]octan-1-ium bromide (A10)

Analytical data for **A10**: ^{1}H NMR (500 MHz, D_2O) δ 8.21 (d, *J* = 10.0 Hz, 2H), 8.13 (d, *J* = 10.0 Hz, 2H), 3.83 (t, *J* = 7.5 Hz, 6H), 3.33 (t, *J* = 7.5 Hz, 6H) and 3.33 (s, 3H); ^{13}C NMR (126 MHz, D_2O) δ 190.4, 143.7, 138.3, 128.9, 127.8, 52.9, 44.0, 42.9; HRMS (ESI+) exact mass calcd for $C_{15}H_{20}N_2O_3S$ requires m/z [M-Br]$^+$: 308.1195. Found m/z 308.1196.

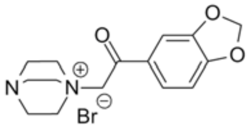

1-(2-(benzo[d][1,3]dioxol-5-yl)-2-oxoethyl)-1,4-diazabicyclo[2.2.2]octan-1-ium bromide (A11)

Analytical data for **A11**: ^{1}H NMR (500 MHz, D_2O) δ 7.63 (d, *J* = 10.0 Hz, 1H), 7.44 (s, 1H), 7.02 (d, *J* = 10.0 Hz, 1H), 6.13 (s, 2H), 3.78 (t, *J* = 7.5 Hz, 6H), 3.30 (t, *J* = 7.5 Hz, 6H); ^{13}C NMR (101 MHz, D_2O) δ 189.3, 153.2, 148.1, 128.6, 125.3, 108.3, 107.1, 102.4, 52.8, 44.0; HRMS (ESI+) exact mass calcd for $C_{15}H_{18}N_2O_3$ requires m/z [M-Br]$^+$: 274.1317. Found m/z 274.1319.

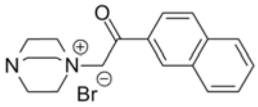

1-(2-(naphthalen-2-yl)-2-oxoethyl)-1,4-diazabicyclo[2.2.2]octan-1-ium bromide (A12)

Analytical data for **A12**: ^{1}H NMR (500 MHz, D_2O) δ 8.48 (s, 1H), 8.05 (d, *J* = 5.0 Hz, 1H), 7.98 (dd, *J* = 10.0, 5.0 Hz, 2H), 7.88 (dd, *J* = 10.0, 1.5 Hz, 1H), 7.74 (dd, *J* = 7.5, 1.5 Hz, 1H), 7.67 (t, *J* = 7.5 Hz, 1H), 3.80 (t, *J* = 7.5 Hz, 6H), 3.31 (t, *J* = 7.5 Hz, 6H); ^{13}C NMR (126 MHz, D_2O) δ 191.2, 135.9, 131.8, 130.5, 129.82, 129.77, 128.9, 127.8, 127.4, 122.5, 52.9, 44.1; HRMS (ESI+) exact mass calcd for $C_{18}H_{20}N_2O$ requires m/z [M-Br]$^+$: 280.1576. Found m/z 280.1577.

3.3 preparation of catalyst

3.3.1 Synthesis of silver phosphate[2]

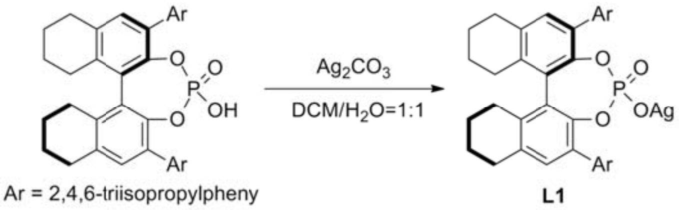

L1

To a solution of chiral phosphoric (140 mg, 0.25 mmol) in CH_2Cl_2 (1.5 mL) in the dark was added Ag_2CO_3 (34.5 mg, 0.125 mmol) followed by distilled water 1.5 mL. The resulting mixture was stirred for one hour. After this time, the mixture was diluted with water (3 mL) and CH_2Cl_2 (3mL). The aqueous layer extracted with CH_2Cl_2. The combined organic extracts were filtered through celite and concentrated to afford the product as a fluffy white solid (163 mg, 0.24 mmol, 95% yield).

3.3.2 Preparation of ion-pair catalyst

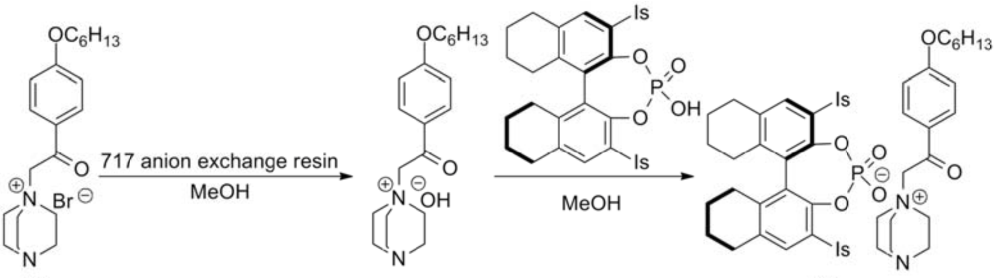

A8 C1

A solution of quaternary ammonium salt **A8** (1.0 mmol) and 707 anion exchange resin (4.0 g, 12.0 mmol, 3mmol/g) in 15 mL methanol was stirred at rt for 3 hours, then filtered. The filtrate was added 8H-TRIP phosphoric acid (1.0 mmol, 760 mg) and stirred for two hours at rt. Then concentrated to get the ionic pair catalyst **C1** quantitatively.

Analytical data for **C1**:^{1}H NMR (500 MHz, $CDCl_3$) δ 7.90 (d, *J* = 8.5 Hz, 2H), 6.97 (s, 2H), 6.93 (s, 2H), 6.84 (s, 2H), 6.64 (d, *J* = 8.5 Hz, 2H), 6.05 (d, *J* = 15.0 Hz, 1H), 4.42 (d, *J* = 15.0 Hz, 1H), 3.97 (d, *J* = 2.4 Hz, 2H), 3.51 (m, 3H), 3.40 (m, 3H), 2.95-2.58 (m, 20H), 2.28 (d, *J* = 16.6 Hz, 2H), 1.85-1.70 (m, 10H), 1.47-1.45 (m, 2H), 1.35-1.34 (m, 4H), 1.26-1.24 (m, 12H), 0.98 (m, 11H), 0.91 (m, 8H), 0.86 (d, *J* = 6.8 Hz, 6H); ^{13}C NMR (126 MHz, cdcl$_3$) δ 189.8, 164.5, 148.3, 147.1, 146.0, 135.9, 134.0, 132.2, 132.1, 131.2, 129.0, 128.1, 127.0, 120.9, 119.5, 114.4, 77.3, 68.3, 65.2, 52.0, 44.5, 33.9, 31.5, 30.7, 30.3, 29.2, 29.1, 27.8, 26.2, 25.7, 24.7, 24.3, 24.0, 23.5, 23.3, 23.1, 23.0, 22.6, 14.0; ^{31}P NMR (162 MHz, $CDCl_3$) δ 2.40; HRMS (ESI+) exact mass calcd for $C_{20}H_{31}N_2O_2$ requires m/z [M-phosphate]$^+$: 331.2380. Found m/z 331.2395. HRMS (ESI-) exact mass calcd for $C_{50}H_{64}O_4P_1$ requires m/z [M-ammonium]$^-$: 759.4548. Found m/z 759.4570.

4. General procedure for asymmetric 3-*exo* iodo-cycloetherification

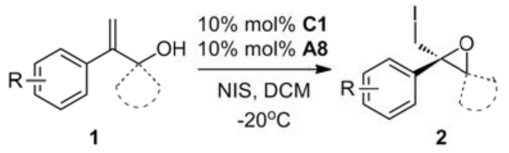

General procedure E

In a Schlenk tube, ion pair catalyst **C1** (0.01 mmol), ammonium salt **A8** (0.01 mmol), NIS (0.12 mmol) and CH_2Cl_2 (1 mL) was added, then the reaction mixture was cooled to -20 °C. Allyl alcohol **1** (0.1 mmol) in 0.5 mL $CH_2Cl_2$was added dropwise and the reaction was quenched by the addition of $Na_2S_2O_3$ aqueous solution at indicated time (monitored by TLC). The organic layer was separated, and the aqueous layer was extracted with ethyl acetate for three times. Then the organic layer combined washed with brine, dried over Na_2SO_4, filtered and then concentrated. The residue was purified by preparative TLC (ethyl acetate/petroleum ether = 1:30, v/v) to afford the product.

*Note: Racemic products were obtained by following general procedure E using rac-**C1** as catalyst.*

Characterization of product

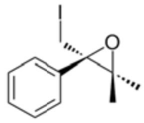

(*R*)-2-(iodomethyl)-3,3-dimethyl-2-phenyloxirane (2a)

Prepared according to the general procedure E with **1a** (16.2 mg, 0.1 mmol) over the course of 107 h at -20 °C as a yellow liquid (28.2 mg, 99% yield, 94% *ee*). The enantiomeric purity was determined by HPLC analysis (ChiralPak ID3 column, hexane/*i*-PrOH 98.5:1.5, 0.5 mL/min, t_{major} = 5.98 min, t_{minor}= 5.60 min). ^{1}H NMR (500 MHz, $CDCl_3$) δ 7.39-7.30 (m, 5H), 3.61-3.51 (m, 2H), 1.54 (s, 3H), 1.02 (s, 3H); ^{13}C NMR (126 MHz, $CDCl_3$) δ 138.6, 128.0, 127.7, 127.3, 68.2, 67.8, 22.8, 20.8, 11.6; IR (neat) 2961, 1447, 763, 700 cm^{-1}; HRMS (EI+) exact mass calcd for $C_{11}H_{13}O$: m/z 161.0966 ($[M-I]^+$), found: m/z 161.0964. $[\alpha]_D^{23.5}$ = -23.5 (*c* 0.55, $CHCl_3$).

Gram scale synthesis of 2a: At nitrogen atmosphere, CH_2Cl_2 (40 ml) was added to a mixture of ion pair catalyst **C1** (335 mg, 0.31 mmol), ammonium salt **A8** (126 mg, 0.31 mmol) and NIS (1.7g, 7.56 mmol) under -20 °C. After the mixture stirred for one minute, **1a** (1.0 g, 6.17 mmol) in CH_2Cl_2 (3.0ml) was added dropwise. After 127 h, the reaction was quenched by the addition of $Na_2S_2O_3$ aqueous solution. The organic layer was separated, and the aqueous layer was extracted with ethyl acetate for three times. Then the organic layer combined washed with brine, dried over Na_2SO_4, filtered and then concentrated. The residue was purified by column chromatography (ethyl acetate/petroleum ether = 1/100, v/v) to afford 1.47 g (83 %) yellow liquid. Enantiomeric excess was found to be 93% measured by chiral HPLC (ChiralPak AD-H column, hexane/*i*-PrOH 98.5:1.5 0.7 mL/min, t_{major} = 5.77 min, t_{minor} = 5.41 min).

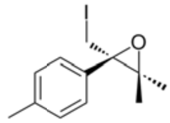

(*R*)-2-(iodomethyl)-3,3-dimethyl-2-(p-tolyl)oxirane (2aa)

Prepared according to the general procedure E with **1aa** (17.6 mg, 0.1 mmol) over the course of 111 h at -20 °C as a yellow liquid (27.6 mg, 95% yield, 93% *ee*). The enantiomeric purity was determined by HPLC analysis (ChiralPak ID3 column, hexane/*i*-PrOH 98.5:1.5, 0.7 mL/min, t_{major} = 3.63 min, t_{minor}=

3.44 min). ^{1}H NMR (500 MHz, $CDCl_3$) δ 7.27 (d, *J* = 10.0 Hz, 2H), 7.16 (d, *J* = 10.0 Hz, 2H), 3.59-3.55 (m, 2H), 2.35 (s, 3H), 1.52 (s, 3H), 1.02 (s, 3H); ^{13}C NMR (126 MHz, $CDCl_3$) δ 137.4, 135.5, 128.7, 127.1, 68.1, 67.8, 22.8, 21.2, 20.8, 12.0; IR (neat) 2922, 1515, 1142, 813, 554 cm^{-1}; HRMS (EI+) exact mass calcd for $C_{12}H_{15}O$: m/z 175.1123 ([M-I]$^+$), found: m/z 175.1120. $[\alpha]_D^{23.5}$ = -21.5 (*c* 0.44, $CHCl_3$).

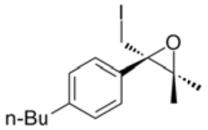

(*R*)-2-(4-butylphenyl)-2-(iodomethyl)-3,3-dimethyloxirane (2ab)

Prepared according to the general procedure E with **1ab** (21.8 mg, 0.1 mmol) over the course of 111 h at -20 °C as a yellow liquid (28.2 mg, 91% yield, 93% *ee*). The enantiomeric purity was determined by HPLC analysis (ChiralPak ADH column, hexane/iPrOH 98.5:1.5, 0.4 mL/min, t_{major} = 10.98 min, t_{minor}= 10.31 min). ^{1}HN NMR (500 MHz, $CDCl_3$) δ 7.28 (d, *J* = 10.0 Hz, 2H), 7.15 (d, *J* = 10.0 Hz, 2H), 3.59-3.55 (m, 2H), 2.61 (t, *J* = 7.5 Hz, 2H), 1.63-1.57 (m, 2H), 1.52 (s, 3H), 1.39-1.32 (m, 2H), 1.02 (s, 3H), 0.93 (t, *J* = 7.5 Hz, 3H); ^{13}C NMR (126 MHz, $CDCl_3$) δ 142.4, 135.7, 128.0, 127.1, 68.1, 67.8, 35.4, 33.5, 22.8, 22.4, 20.8, 14.0, 11.9; IR (neat) 2956, 1456, 1010, 831, 573 cm-1; HRMS (EI+) exact mass calcd for $C_{15}H_{21}O$: m/z 217.1592 ([M-I]$^+$), found: m/z 217.1594. $[\alpha]_D^{23.5}$ = -19.1 (*c* 1.06, $CHCl_3$).

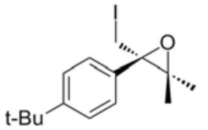

(*R*)-2-(4-(tert-butyl)phenyl)-2-(iodomethyl)-3,3-dimethyloxirane (2ac)

Prepared according to the general procedure E with **1ac** (21.8 mg, 0.1 mmol) over the course of 111 h at -20 °C as a white solid (28.2 mg, 86% yield, 91% *ee*). The enantiomeric purity was determined by HPLC analysis (Lux 5μ cellulose-3 column, hexane/*i*-PrOH 98:2, 0.5 mL/min, t_{major} = 7.68 min, t_{minor}= 8.25 min). ^{1}H NMR (500 MHz, $CDCl_3$) δ 7.35 (d, *J* = 8.5 Hz, 2H), 7.31 (d, *J* = 8.5 Hz, 2H), 3.58 (d, *J* = 10.0 Hz, 1H), 3.55 (d, *J* = 10.0 Hz, 1H), 1.53 (s, 3H), 1.32 (s, 9H), 1.02 (s, 3H); ^{13}C NMR (126 MHz, $CDCl_3$) δ 150.5, 135.3, 126.9, 124.9, 68.1, 67.7, 34.6, 31.4, 22.8, 20.8, 11.8; IR (neat) 2962, 1270, 1008, 830, 619 cm^{-1}; HRMS (EI+) exact mass calcd for $C_{11}H_{13}O$: m/z 217.1592 ([M-I]$^+$), found: m/z 217.1590. $[\alpha]_D^{23.5}$ = -13.6 (*c* 0.93, $CHCl_3$).

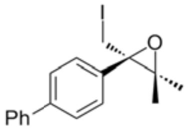

(*R*)-2-([1,1'-biphenyl]-4-yl)-2-(iodomethyl)-3,3-dimethyloxirane (2ad)

Prepared according to the general procedure E with **1ad** (23.8 mg, 0.1 mmol) over the course of 62 h at -20 °C as a yellow liquid (33.5 mg, 92% yield, 94% *ee*). The enantiomeric purity was determined by HPLC analysis (ChiralPak ID3 column, hexane/*i*-PrOH 98:2, 0.7 mL/min, t_{major} = 4.79 min, t_{minor}= 4.15 min). ^{1}H NMR (500 MHz, $CDCl_3$) δ 7.61 (d, *J* = 10.0 Hz, 2H), 7.59 (d, *J* = 10.0 Hz, 2H), 7.43-7.47 (m, 4H), 7.35 (t, *J* = 7.5 Hz, 1H), 3.63 (d, *J* = 10.0 Hz, 1H), 3.61 (d, *J* = 10.0 Hz, 1H),1.56 (s, 3H), 1.07 (s, 3H); ^{13}C NMR (126 MHz, $CDCl_3$) δ 140.6, 140.5, 137.6, 128.8, 127.7, 127.4, 127.1, 126.7, 68.3, 67.7, 22.9, 20.8, 11.5; IR (neat) 2922, 1487, 829, 755, 580 cm^{-1}; HRMS (EI+) exact mass calcd for $C_{17}H_{17}IO$:

m/z 364.0324 ([M]$^+$), found: m/z 364.0326. $[\alpha]_D^{23.5}$ = -9.3 (*c* 1.34, $CHCl_3$).

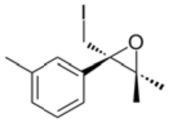

(*R*)-2-(iodomethyl)-3,3-dimethyl-2-(m-tolyl)oxirane (2ae)

Prepared according to the general procedure E with **1ae** (17.2 mg, 0.1 mmol) over the course of 134 h at -20 °C as a yellow liquid (27.3 mg, 93% yield, 90% *ee*). The enantiomeric purity was determined by HPLC analysis (Lux 5μ cellulose-4 column, hexane/*i*-PrOH 98:2, 0.7 mL/min, t_{major} = 5.89 min, t_{minor}= 5.51 min). ^{1}H NMR (500 MHz, $CDCl_3$) δ 7.23 (t, *J* = 5.0 Hz, 1H), 7.18 (s, 1H), 7.17 (d, *J* = 5.0 Hz, 1H), 7.11 (d, *J* = 5.0 Hz, 1H), 3.59 (d, *J* = 10.0 Hz, 1H), 3.56 (d, *J* = 10.0 Hz, 1H), 2.37 (s, 3H), 1.53 (s, 3H), 1.03 (s, 3H); ^{13}C NMR (126 MHz, $CDCl_3$) δ 138.4, 137.7, 128.5, 127.9, 127.8, 124.4, 68.2, 67.8, 22.8, 21.5, 20.8, 11.7; IR (neat) 2924, 1456, 1376, 785 cm^{-1}; HRMS (EI+) exact mass calcd for $C_{12}H_{15}O$: m/z 175.1123 ([M-I]$^+$), found: m/z 175.1119. $[\alpha]_D^{23.5}$ = -25.7 (*c* 0.84, $CHCl_3$).

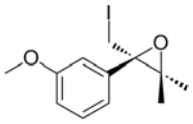

(*R*)-2-(iodomethyl)-2-(3-methoxyphenyl)-3,3-dimethyloxirane (2af)

Prepared according to the general procedure E with **1af** (16.2mg, 0.1 mmol) over the course of 120 h at -20 °C as a yellow liquid (18.7 mg, 57% yield, 91% *ee*). The enantiomeric purity was determined by HPLC analysis (ChiralPak OD-H column, hexane/*i*-PrOH 98:2, 0.4 mL/min, t_{major} = 7.75 min, t_{minor}= 9.12 min). ^{1}H NMR (500 MHz, $CDCl_3$) δ 7.26 (t, *J* = 10.0 Hz, 1H), 6.96 (d, *J* = 10.0 Hz, 1H), 6.93 (s, 1H), 6.84 (d, *J* = 5.0 Hz, 1H), 3.82 (s, 3H), 3.60-3.55 (m, 2H), 1.5 (s, 3H), 1.04 (s, 3H); ^{13}C NMR (126 MHz,) δ 159.3, 140.2, 129.1, 119.5, 113.2, 113.0, 68.3, 67.8, 55.3, 22.7, 20.8, 11.3; IR (neat) 2917, 1109, 813, 555 cm^{-1}; HRMS (EI+) exact mass calcd for $C_{12}H_{15}O_2$: m/z 191.1072 ([M-I]$^+$), found: m/z 191.1074. $[\alpha]_D^{23.5}$ = -30.8 (*c* 1.21, $CHCl_3$).

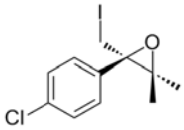

(*R*)-2-(4-chlorophenyl)-2-(iodomethyl)-3,3-dimethyloxirane (2ag)

Prepared according to the general procedure **E** with **1ag** (20.2 mg, 0.1 mmol) over the course of 120 h at -20 °C as a yellow liquid (18.1 mg, 55% yield, 93% *ee*). The enantiomeric purity was determined by HPLC analysis (ChiralPak AD-H column, hexane/*i*-PrOH 98.5:1.5, 0.4 mL/min, t_{major} = 12.17 min, t_{minor}= 11.49 min). ^{1}H NMR (500 MHz, $CDCl_3$) δ 7.33 (s, 4H), 3.54 (s, 2H), 1.53 (s, 3H), 1.01 (s, 3H); ^{13}C NMR (126 MHz, $CDCl_3$) 137.1, 133.6, 128.7, 128.3, 68.3, 67.4, 22.8, 20.7, 10.9; IR (neat) 2957, 1376, 1142, 814, 603 cm^{-1}; HRMS (EI+) exact mass calcd for $C_{11}H_{12}OCl$: m/z 195.0577 ([M-I]$^+$), found: m/z 195.0581. $[\alpha]_D^{23.5}$ = -19.2 (*c* 0.58, $CHCl_3$).

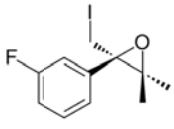

(*R*)-2-(3-fluorophenyl)-2-(iodomethyl)-3,3-dimethyloxirane (2ah)

Prepared according to the general procedure E with **1ah** (18.3 mg, 0.1 mmol) over the course of 107 h at -20 °C as a yellow liquid (10.1 mg, 32% yield, 86% *ee*). The enantiomeric purity was determined by

HPLC analysis (ChiralPak ID3 column, hexane/*i*-PrOH 98:2, 0.7 mL/min, t_{major} = 6.73 min, t_{minor} = 6.33 min). ^{1}H NMR (500 MHz, $CDCl_3$) δ 7.32 (dt, *J* = 10.0, 5.0 Hz, 1H), 7.17 (d, *J* = 10.0 Hz, 1H), 7.11 (d, *J* = 10.0 Hz, 1H), 7.01 (td, *J* = 10.0, 5.0 Hz, 1H), 3.57 (d, *J* = 10.0 Hz, 2H), 3.54 (d, *J* = 10.0 Hz, 2H), 1.53 (s, 3H), 1.03 (s, 3H); ^{13}C NMR (126 MHz, $CDCl_3$) δ 162.4 (d, *J* = 272.4 Hz), 141.2 (d, *J* = 5.0 Hz), 129.7 (d, *J* = 7.6 Hz), 122.8, 114.8 (d, *J* = 21.4 Hz), 114.5 (d, *J* = 22.7 Hz), 68.5, 67.4, 22.7, 20.7, 10.6; ^{19}F NMR (376 MHz, $CDCl_3$) δ -112.8; IR (neat) 2957, 1437, 763, 573 cm^{-1}; HRMS (EI+) exact mass calcd for $C_{11}H_{13}O$: m/z 161.0966 ($[M-I]^+$), found: m/z 161.0964. $[\alpha]_D^{23.5}$ = -23.9 (*c* 0.43, $CHCl_3$).

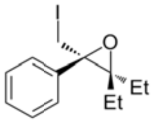

(*R*)-2,2-diethyl-3-(iodomethyl)-3-phenyloxirane (2b)

Prepared according to the general procedure E with **1b** (19.3 mg, 0.1 mmol) over the course of 115 h at -20 °C as a yellow liquid (19.0 mg, containing about 9% rearrangement product, 60% yield, 95% *ee*). The enantiomeric purity was determined by HPLC analysis (ChiralPak IC column, hexane/*i*-PrOH 98:2, 0.7 mL/min, t_{major} = 6.34 min, t_{minor} = 5.72 min). ^{1}H NMR (500 MHz, $CDCl_3$) δ 7.36-7.30 (m, 5H), 3.64-3.59 (m, 2H), 1.95-1.87 (m, 1H), 1.74-1.71 (m, 1H), 1.25-1.20 (m, 2H), 1.11 (t, *J* = 7.5 Hz, 3H), 0.82 (t, *J* = 7.5 Hz, 3H); ^{13}C NMR (126 MHz, $CDCl_3$) δ 138.8, 128.9, 127.9, 127.6, 75.8, 69.0, 24.8, 23.4, 12.2, 10.0, 8.8; IR (neat) 2956, 1447, 701, 577 cm^{-1}; HRMS (EI+) exact mass calcd for $C_{13}H_{17}O$: m/z 189.1279 ($[M-I]^+$), found: m/z 189.1278. $[\alpha]_D^{23.5}$ = -65.7 (*c* 0.47, $CHCl_3$).

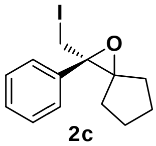

2c

gram scale (5 mol% **C1**)
47 h, 92%, 98% ee
3 mmol (1 mol% **C1**)
72 h, 87%, 97.6% ee

(*R*)-2-(iodomethyl)-2-phenyl-1-oxaspiro[2.4]heptanes (2c)

Prepared according to the general procedure E with **1c** (19.2 mg, 0.1 mmol) over the course of 44 h at -20 °C as a yellow liquid (32.1 mg, 99% yield, 97% *ee*). The enantiomeric purity was determined by HPLC analysis (ChiralPak OD-H column, hexane/*i*-PrOH 98:2, 0.7 mL/min, t_{major} = 7.18 min, t_{minor} = 7.81 min). ^{1}H NMR (500 MHz, $CDCl_3$) δ 7.36 (d, *J* = 5.0 Hz, 4H), 7.32-7.30 (m, 1H), 3.70 (d, *J* = 10.0 Hz, 1H), 3.45 (d, *J* = 10.0 Hz, 1H), 2.16-2.11 (m, 1H), 1.91-1.71 (m, 4H), 1.58-1.52 (m, 1H), 1.45-1.39 (m, 1H), 1.30-1.26 (m, 1H); ^{13}C NMR (126 MHz, $CDCl_3$) δ 128.1, 127.7, 126.7, 80.0, 66.3, 32.6, 30.7, 25.4, 25.1, 12.0; IR (neat) 2960, 1447, 762, 572 cm^{-1}; HRMS (EI+) exact mass calcd for $C_{13}H_{15}O$: m/z 187.1123 ($[M-I]^+$), found: m/z 187.1125. $[\alpha]_D^{23.5}$ = -38.5 (*c* 1.03, $CHCl_3$).

Gram scale synthesis of 2c: At nitrogen atmosphere, CH_2Cl_2 (30 mL) was added to a mixture of ion pair catalyst **C1** (290 mg, 0.27 mmol), ammonium salt **A8** (109 mg, 0.27 mmol) and NIS (1.46 g, 6.38 mmol) under -20 °C. After the mixture stirred for 5 min, **1c** (1.0 g) in CH_2Cl_2 (3.0 mL) was added dropwise. After 47 h, the reaction was quenched by the addition of $Na_2S_2O_3$ aqueous solution. The organic layer was separated, and the aqueous layer was extracted with ethyl acetate for three times. Then the organic layer combined, washed with brine, dried over Na_2SO_4, filtered and then concentrated. The residue was purified by column chromatography (ethyl acetate/petroleum ether = 1:100, v/v) to afford 1.54 g (92%) yellow liquid. Enantiomeric excess was found to be 98% measured by chiral HPLC (ChiralPak OD-H column, hexane/*i*-PrOH 98:2 0.7 mL/min, t_{major} = 6.56 min, t_{minor} = 7.04 min).

Synthesis of 2c using 1 mol% C1 on 3 mmol scale:

Following gram scale synthesis of **2c**, synthesis of **2c** on 3 mmol scale was carried out using 1 mol% **C1** (32 mg, 0.03 mmol) under -40 °C. After 72 h, most of **1c** was consumed and work-up of the reaction afforded **2c** (820 mg,

87%). Enantiomeric excess was found to be 97.6% measured by chiral HPLC (ChiralPak OD-H column, hexane/i-PrOH 98:2 0.7 mL/min, t_{major} = 7.16 min, t_{minor} = 7.79 min).

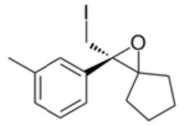

(*R*)-2-(iodomethyl)-2-(m-tolyl)-1-oxaspiro[2.4]heptane (2ca)

Prepared according to the general procedure E with **1ca** (20.8 mg, 0.1 mmol) over the course of 70 h at -20 °C as a yellow liquid (28.3 mg, 84 % yield, 95% *ee*). The enantiomeric purity was determined by HPLC analysis (Lux 5μ cellulose-4 column, hexane/*i*-PrOH 95:5, 0.7 mL/min, t_{major} = 6.32 min, t_{minor}= 5.86 min). ^{1}H NMR (500 MHz, $CDCl_3$) δ 7.24 (t, *J* = 7.5 Hz, 1H), 7.15(s, 1H), 7.14 (d, *J* = 7.5 Hz, 1H), 7.12 (d, *J* = 7.5 Hz, 1H), 3.69 (d, *J* = 10.0 Hz, 1H), 3.44 (d, *J* = 10.0 Hz, 1H), 2.37 (s, 3H), 2.15-2.10 (m, 1H), 1.90-1.84 (m, 1H), 1.80-1.72 (m, 3H), 1.60-1.51 (m, 1H), 1.46-1.40 (m, 1H), 1.31-1.26 (m, 1H); ^{13}C NMR (126 MHz, $CDCl_3$) δ 138.5, 137.7, 128.5, 127.9, 127.3, 123.8, 80.0, 66.3, 32.7, 30.7, 25.4, 25.1, 21.5, 12.3; IR (neat) 2961, 1447, 763, 700 cm-1; HRMS (EI+) exact mass calcd for $C_{14}H_{17}OI$: m/z 328.0324 ($[M]^+$), found: m/z 328.0322. $[\alpha]_D^{23.5}$ = -29.8 (*c* 1.13, $CHCl_3$)

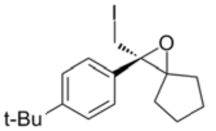

(*R*)-2-(4-(tert-butyl)phenyl)-2-(iodomethyl)-1-oxaspiro[2.4]heptane (2cb)

Prepared according to the general procedure E with **1cb** (24.5 mg, 0.1 mmol) over the course of 21 h at -20 °C as a yellow liquid (31.1 mg, 82% yield, 92% *ee*). The enantiomeric purity was determined by HPLC analysis (Lux 5μ cellulose-4 column, hexane/*i*-PrOH 98:2, 0.7 mL/min, t_{major} = 6.02 min, t_{minor}= 5.61 min). ^{1}H NMR (500 MHz, $CDCl_3$) δ 7.35 (d, *J* = 7.5 Hz, 2H), 7.27 (d, *J* = 7.5 Hz, 2H), 3.66 (d, *J* = 10.0 Hz, 1H), 3.46 (d, *J* = 10.0 Hz, 1H), 2.16-2.10 (m, 1H), 1.88-1.83 (m, 1H), 1.79-1.71 (m, 3H), 1.57-1.51 (m, 1H), 1.47-1.42 (m, 1H), 1.32 (s, 9H), 1.30-1.26 (m, 1H); ^{13}C NMR (126 MHz, $CDCl_3$) δ 150.5, 135.4, 126.4, 124.9, 79.9, 66.1, 34.6, 32.6, 31.4, 30.8, 25.4, 25.1, 12.3; IR (neat) 2961, 1447, 763, 700 cm^{-1}; HRMS (EI+) exact mass calcd for $C_{17}H_{23}O$: m/z 243.1749 ($[M-I]^+$), found: m/z 243.1748. $[\alpha]_D^{23.5}$ = -14.8 (*c* 1.24, $CHCl_3$).

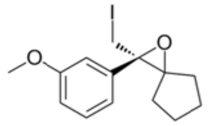

(*R*)-2-(iodomethyl)-2-(3-methoxyphenyl)-1-oxaspiro[2.4]heptane (2cc)

Prepared according to the general procedure E with **1cc** (22.0 mg, 0.1 mmol) over the course of 45 h at -20 °C as a yellow liquid (28.2 mg, 81% yield, 96.5% *ee*). The enantiomeric purity was determined by HPLC analysis (Lux 5μ cellulose-4 column, hexane/*i*-PrOH 98:2, 0.7 mL/min, t_{major} = 8.45 min, t_{minor}= 7.95 min). ^{1}H NMR (500 MHz, $CDCl_3$) δ 7.27 (t, *J* = 7.5 Hz, 1H), 6.93 (d, *J* = 7.5 Hz, 1H), 6.90 (s, 1H), 6.84 (dd, J_1 = 7.5, 2.5 Hz, 1H), 3.82 (s, 3H), 3.69 (d, *J* = 10.0 Hz, 1H), 3.43 (d, *J* = 10.0 Hz, 1H), 2.15-2.09 (m, 1H), 1.88-1.86 (m, 1H), 1.83-1.70 (m, 3H), 1.58-1.51 (m, 1H), 1.47-1.41 (m, 1H), 1.33-1.27 (m, 1H); ^{13}C NMR (126 MHz, $CDCl_3$) δ 159.4, 140.1, 129.2, 119.0, 113.2, 112.5, 80.1, 66.3, 55.3, 32.6, 30.8, 25.4, 25.1, 11.9; IR (neat) 2961, 1447, 763, 700 cm^{-1}; HRMS (EI+) exact mass calcd for $C_{14}H_{17}O_2I$: m/z 344.0273 ($[M]^+$), found: m/z 344.0269. $[\alpha]_D^{23.5}$ = -35.3 (*c* 1.09, $CHCl_3$).

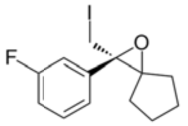

(*R*)-2-(3-fluorophenyl)-2-(iodomethyl)-1-oxaspiro[2.4]heptane (2cd)

Prepared according to the general procedure E with **1cd** (20.5 mg, 0.1 mmol) over the course of 42 h at -20 °C as a yellow liquid (26.7 mg, 81% yield, 96.5% *ee*). The enantiomeric purity was determined by HPLC analysis (Lux 5μ cellulose-4 column, hexane/*i*-PrOH 98:2, 0.7 mL/min, t_{major} = 6.56 min, t_{minor}= 6.27 min). ^{1}H NMR (500 MHz, $CDCl_3$) δ 7.33 (dt, *J* = 10.0, 7.5 Hz, 1H), 7.1 (d, *J* = 7.5 Hz, 1H), 7.07 (dd, *J* = 10.0, 1.5 Hz, 1H), 7.00 (t, *J* = 7.5 Hz, 1H), 3.67 (d, *J* = 10.5 Hz, 1H), 3.42 (d, *J* = 10.5 Hz, 1H), 2.15-2.09 (m, 1H), 1.89-1.85 (m, 1H), 1.81-1.71 (m, 3H), 1.57-1.54 (m, 1H), 1.44-1.38 (m, 1H), 1.32-1.28 (m, 1H); ^{13}C NMR (126 MHz, $CDCl_3$) δ 162.4 (d, *J* = 255.5 Hz), 141.3, 129.8 (d, *J* = 7.7 Hz), 122.3 (d, *J* = 2.9 Hz), 114.7 (d, *J* = 21.0 Hz), 114.0 (d, *J* = 22.8 Hz), 80.3, 65.9, 32.5, 30.7, 25.3, 25.1, 11.1; ^{19}F NMR (376 MHz, $CDCl_3$) δ -112.7; IR (neat) 2961, 1447, 763, 700 cm^{-1}; HRMS (EI+) exact mass calcd for $C_{13}H_{14}OFI$: m/z 332.0073 ([M]$^+$), found: m/z 332.0070. $[\alpha]_D^{23.5}$ = -48.4 (*c* 1.03, $CHCl_3$).

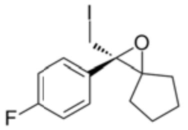

(*R*)-2-(4-fluorophenyl)-2-(iodomethyl)-1-oxaspiro[2.4]heptane (2ce)

Prepared according to the general procedure E with **1ce** (20.2 mg, 0.1 mmol) over the course of 51 h at -20 °C as a yellow liquid (33.2 mg, 97% yield, 96.5% *ee*). The enantiomeric purity was determined by HPLC analysis (ChiralPak PA-2 column, hexane/*i*-PrOH 98:2, 0.7 mL/min, t_{major} = 7.54 min, t_{minor}= 8.31 min). ^{1}H NMR (500 MHz, $CDCl_3$) δ 7.35-7.32 (m, 2H), 7.06-7.02 (m, 2H), 3.64 (d, *J* = 10.0 Hz, 1H), 3.43 (d, *J* = 10.0 Hz, 1H), 2.15-2.10 (m, 1H), 1.89-1.84 (m, 1H), 1.80-1.71 (m, 3H), 1.58-1.54 (m, 1H), 1.44-1.38 (m, 1H), 1.28-1.22 (m, 1H); ^{13}C NMR (126 MHz, $CDCl_3$) δ 162.2 (d, *J* = 246.1 Hz), 134.4, 128.5 (d, *J* = 8.5 Hz), 115.1 (d, *J* = 21.0 Hz), 80.0, 65.8, 32.6, 30.7, 25.4, 25.1, 11.9; ^{19}F NMR (376 MHz, $CDCl_3$) δ -114.3; IR (neat) 2961, 1447, 763, 700 cm^{-1}; HRMS (EI+) exact mass calcd for $C_{13}H_{14}OFI$: m/z 332.0073 ([M]$^+$), found: m/z 332.0070. $[\alpha]_D^{23.5}$ = -7.1 (*c* 1.20, $CHCl_3$).

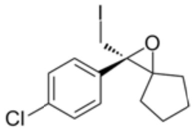

(*R*)-2-(4-chlorophenyl)-2-(iodomethyl)-1-oxaspiro[2.4]heptane (2cf)

Prepared according to the general procedure E with **1cf** (22.5 mg, 0.1 mmol) over the course of 51 h at -20 °C as a yellow liquid (32.5 mg, 92% yield, 98% *ee*). The enantiomeric purity was determined by HPLC analysis (ChiralPak PA-2 column, hexane/*i*-PrOH 98:2, 0.7 mL/min, t_{major} = 8.17 min, t_{minor}= 9.39 min). ^{1}H NMR (500 MHz, $CDCl_3$) δ 7.34-7.29 (m, 4H), 3.65 (d, *J* = 10.0 Hz, 1H), 3.42 (d, *J* = 10.0 Hz, 1H), 2.15-2.09 (m, 1H), 1.89-1.85 (m, 1H), 1.79-1.71 (m, 3H), 1.58-1.55 (m, 1H), 1.43-1.37 (m, 1H), 1.29-1.23 (m, 1H); ^{13}C NMR (126 MHz, $CDCl_3$) δ 137.2, 133.6, 128.4, 128.1, 80.1, 65.9, 32.6, 30.7, 25.3, 25.1, 11.4; IR (neat) 2961, 1447, 763, 700 cm^{-1}; HRMS (EI+) exact mass calcd for $C_{13}H_{14}OClI$: m/z 347.9778 ([M]$^+$), found: m/z 347.9781. $[\alpha]_D^{23.5}$ = -5.2 (*c* 1.22, $CHCl_3$).

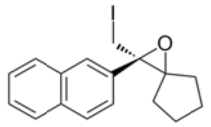

(*R*)-2-(iodomethyl)-2-(naphthalen-2-yl)-1-oxaspiro[2.4]heptane (2cg)

Prepared according to the general procedure E with **1cg** (23.6 mg, 0.1 mmol) over the course of 45 h at -20 °C as a yellow liquid (27.7 mg, 75% yield, 94% *ee*). The enantiomeric purity was determined by HPLC analysis (Lux 5μ cellulose-4 column, hexane/*i*-PrOH 98:2, 0.7 mL/min, t_{major} = 7.93 min, t_{minor}= 7.07 min). ^{1}H NMR (500 MHz, $CDCl_3$) δ 7.87-7.84 (m, 4H), 7.51-7.46 (m, 3H), 3.83 (d, *J* = 10.0 Hz, 1H), 3.53 (d, *J* = 10.0 Hz, 1H), 2.20-2.16 (m, 1H), 1.94-1.71 (m, 4H), 1.60-1.51 (m, 1H), 1.47-1.41 (m, 1H), 1.34-1.27 (m, 1H); ^{13}C NMR (126 MHz, $CDCl_3$) δ 136.1, 133.0, 132.9, 128.1, 127.9, 127.7, 126.3, 126.1, 126.0, 124.4, 80.3, 66.5, 32.7, 30.8, 25.4, 25.1, 12.0; IR (neat) 2959, 1422, 816, 654 cm^{-1}; HRMS (EI+) exact mass calcd for $C_{17}H_{17}OI$: m/z 364.0324 ($[M]^+$), found: m/z 364.0328. $[\alpha]_D^{23.5}$ = -6.5 (*c* 1.24, $CHCl_3$).

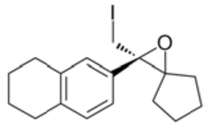

(*R*)-2-(iodomethyl)-2-(5,6,7,8-tetrahydronaphthalen-2-yl)-1-oxaspiro[2.4]heptane (2ch)

Prepared according to the general procedure E with **1ch** (23.9 mg, 0.1 mmol) over the course of 45 h at -20 °C as a yellow liquid (27.7 mg, 76% yield, 91% *ee*). The enantiomeric purity was determined by HPLC analysis (Lux 5μ cellulose-4 column, hexane/*i*-PrOH 98:2, 0.7 mL/min, t_{major} = 6.95 min, t_{minor}= 6.25 min). ^{1}H NMR (500 MHz, $CDCl_3$) δ 7.06-7.01 (m, 3H), 3.67 (d, *J* = 10.0 Hz, 1H), 3.44 (d, *J* = 10.0 Hz, 1H), 2.80-2.72 (m, 4H), 2.14-2.10 (m, 1H), 1.88-1,85 (m, 1H),1.81-1.72 (m, 6H), 1.58-1.53 (m, 2H), 1.49-1.42 (m, 1H), 1.32-1.26 (m, 1H); ^{13}C NMR (126 MHz, $CDCl_3$) δ 136.9, 136.5, 135.4, 128.7, 127.2, 123.8, 79.9, 66.2, 32.7, 30.8, 29.5, 29.2, 25.4, 25.1, 23.2, 12.6.; IR (neat) 2926, 1434, 825, 603 cm^{-1}; HRMS (EI+) exact mass calcd for $C_{17}H_{21}OI$: m/z 368.0637 ($[M]^+$), found: m/z 368.0633. $[\alpha]_D^{23.5}$ = -5.5 (*c* 1.27, $CHCl_3$).

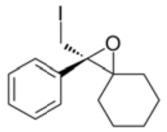

(*R*)-2-(iodomethyl)-2-phenyl-1-oxaspiro[2.5]octane (2d)

Prepared according to the general procedure E with **1d** (20.4 mg, 0.1 mmol) over the course of 44 h at -20 °C as a yellow liquid (32.7 mg, 98% yield, 92% *ee*). The enantiomeric purity was determined by HPLC analysis (ChiralPak OD-H column, hexane/*i*-PrOH 98:2, 0.7 mL/min, t_{major} = 6.56 min, t_{minor}= 7.01 min). ^{1}H NMR (500 MHz, $CDCl_3$) δ 7.39-7.29 (m, 5H), 3.63-3.59 (m, 2H), 1.89-1.70 (m, 4H), 1.53-1.47 (m, 3H), 1.44-1.39 (m, 1H), 1.22-1.19 (m, 2H); ^{13}C NMR (126 MHz, $CDCl_3$) δ 138.5, 128.0, 127.6, 72.8, 68.8, 32.6, 30.8, 25.4, 25.3, 24.4, 11.5; IR (neat) 2930, 1447, 703, 576 cm^{-1}; HRMS (EI+) exact mass calcd for $C_{14}H_{17}O$: m/z 201.1279 ($[M-I]^+$), found: m/z 201.1275. $[\alpha]_D^{23.5}$ = -36.9 (*c* 0.64, $CHCl_3$).

Gram scale synthesis of 2d: At nitrogen atmosphere, CH_2Cl_2 (30 mL) was added to a mixture of ion pair catalyst **C1** (269 mg, 0.25 mmol), ammonium salt **A8** (101 mg, 0.25 mmol) and NIS (1.34 g, 6.0 mmol) under -20 °C. After the mixture stirred for 5 min, **1d** (1.0 g) in CH_2Cl_2 (3.0 mL) was added dropwise. After 48 h, the reaction was quenched by the addition of $Na_2S_2O_3$ aqueous solution. The organic layer

was separated, and the aqueous layer was extracted with ethyl acetate for three times. Then the organic layer combined washed with brine, dried over Na_2SO_4, filtered and then concentrated. The residue was purified by column chromatography (ethyl acetate/petroleum ether = 1:100, v/v) to afford 1.47 g (90% yield) yellow liquid. Enantiomeric excess was found to be 90% measured by chiral HPLC (ChiralPak OD-H column, hexane/*i*-PrOH 98:2 0.7 mL/min, t_{major} = 6.02 min, t_{minor} = 6.38 min).

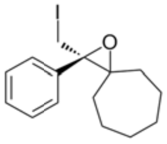

(*R*)-2-(iodomethyl)-2-phenyl-1-oxaspiro[2.6]nonane (2e)

Prepared according to the general procedure E with **1e** (21.7 mg, 0.1 mmol) over the course of 44 h at -20 °C as a yellow liquid (31.2 mg, 91% yield, 98% *ee*). The enantiomeric purity was determined by HPLC analysis (ChiralPak IC column, hexane/*i*-PrOH 95:5, 0.7 mL/min, t_{major} = 6.72 min, t_{minor}= 5.92 min). ^{1}H NMR (500 MHz, $CDCl_3$) δ 7.37-7.30 (m, 5H), 3.64 (d, *J* = 10.0 Hz, 1H), 3.59 (d, *J* = 10.0 Hz, 1H), 2.09-2.04 (m, 1H), 1.97-1.92 (m, 1H), 1.84-1.79 (m, 1H), 1.65-1.59 (m, 4H), 1.50-1.44 (m, 2H); 1.38-1.26 (m, 3H); ^{13}C NMR (126 MHz, $CDCl_3$) δ 138.8, 128.0, 127.6, 127.2, 74.6, 68.7, 34.2, 33.2, 29.2, 28.8, 24.7, 24.0, 12.5; IR (neat) 2924, 1447, 703, 573 cm^{-1}; HRMS (EI+) exact mass calcd for $C_{15}H_{19}O$: m/z 215.1436 ([M-I]$^+$), found: m/z 215.1440. $[\alpha]_D^{23.5}$ = -54.5 (*c*, 0.70, $CHCl_3$).

Gram scale synthesis of 2e: At nitrogen atmosphere, CH_2Cl_2 (25 mL) was added to a mixture of ion pair catalyst **C1** (252 mg, 0.23 mmol), ammonium salt **A8** (95 mg, 0.23 mmol) and NIS (1.25 g, 5.54 mmol) under -20 ^{o}C. After the mixture stirred for one minute, **1e** (1.0 g) in CH_2Cl_2 (3.0 mL) was added dropwise. After 50 h, the reaction was quenched by the addition of $Na_2S_2O_3$ aqueous solution. The organic layer was separated, and the aqueous layer was extracted with ethyl acetate for three times. Then the organic layer combined washed with brine, dried over Na_2SO_4, filtered and then concentrated. The residue was purified by column chromatography (ethyl acetate/petroleum ether = 1:100, v/v) to afford 1.40 g (89% yield) yellow liquid. Enantiomeric excess was found to be 98% measured by chiral HPLC (ChiralPak IC column, hexane/*i*-PrOH 95:5 0.7 mL/min, t_{major} = 6.63 min, t_{minor} = 5.86 min).

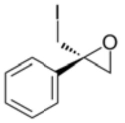

(*R*)-2-(iodomethyl)-2-phenyloxirane (2f)

Prepared according to the general procedure E with **1f** (13.5 mg, 0.1 mmol) over the course of 56 h at -20 °C as a yellow liquid (10.7 mg, 41% yield, 63% *ee*). The enantiomeric purity was determined by HPLC analysis (ChiralPak AD-H column, hexane/*i*-PrOH 98.5:1.5 0.7 mL/min, t_{major} = 23.06 min, t_{minor}= 20.66 min). ^{1}H NMR (500 MHz, $CDCl_3$) δ 7.38 (d, *J* = 7.5 Hz, 2H), 7.33 (t, *J* = 7.5 Hz, 2H), 7.29 (d, *J* = 7.5 Hz, 1H), 4.09 (d, *J* = 10.0 Hz, 1H), 4.04 (d, *J* = 10.0 Hz, 1H), 3.90 (d, *J* = 10.0 Hz, 1H), 3.56 (d, *J* = 10.0 Hz, 1H); ^{13}C NMR (126 MHz, $CDCl_3$) δ 138.5, 128.6, 128.1, 126.0, 73.7, 65.8, 12.9; IR (neat) 2961, 1447, 765, 574 cm^{-1}; HRMS (EI+) exact mass calcd for C_9H_9O: m/z 133.1672 ([M-I]$^+$), found: m/z 133.1674. $[\alpha]_D^{23.5}$ = -4.6 (*c* 0.31, $CHCl_3$).

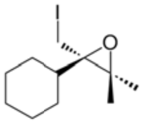

(*R*)-2-cyclohexyl-2-(iodomethyl)-3,3-dimethyloxirane (2g)

Prepared according to the general procedure E with **1g** (16.8 mg, 0.1 mmol) over the course of 74 h at

-20 °C as a yellow liquid (20.9 mg, 71% yield, 37% *ee*). The enantiomeric purity was determined by HPLC analysis (ChiralPak AD-H column, hexane/*i*-PrOH 98:2, 0.7 mL/min, t_{major} = 5.87 min, t_{minor}= 6.37 min). ^{1}H NMR (500 MHz, $CDCl_3$) δ 3.56 (d, *J* = 10.0 Hz, 1H), 3.02 (d, *J* = 10.0 Hz, 1H), 1.89-1.60 (m, 5H), 1.50 (tt, *J* = 10.0, 2.5 Hz, 1H), 1.39 (s, 3H), 1.37 (s, 3H), 1.36-1.35 (m, 1H), 1.27-1.19 (m, 3H); ^{13}C NMR (126 MHz, $CDCl_3$) δ 66.6, 66.0, 43.5, 31.3, 28.6, 27.1, 26.5, 26.3, 22.3, 20.3, 2.6; IR (neat) 2961, 1447, 763, 633 cm-1; HRMS (EI+) exact mass calcd for $C_{11}H_{19}OI$: m/z 294.0481 ($[M-I]^{+}$), found: m/z 294.0479. $[\alpha]_D^{23.5}$ = -10.5 (*c* 0.52, $CHCl_3$).

5. Wagner-Meerwein Rearrangement of epoxide and one-pot procedure

5.1 Wagner-Meerwein Rearrangement of 2c.

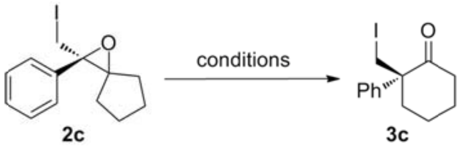

Table S6. Optimization of the rearrangement of 2c[a]

entry	Cat	solvent	C (mmol/ml)	T (°C)	t (h)	*ee* (%)[b]
1	BF_3·Et_2O (1 equiv)	DCM	0.1	0	1.5	91
2	BF_3·Et_2O (5 equiv)	DCM	0.1	0	1.5	91
3	BF_3·Et_2O (10 equiv)	DCM	0.1	0	1.5	89
4	BF_3 ·Et_2O (1 equiv)	DCM	0.1	rt	1.5	90
5	BF_3·Et_2O (1 equiv)	DCM	0.1	-20	2	91
6	BF_3·Et_2O (1 equiv)	DCM	0.1	-78	3	92
7	BF_3·Et_2O (1 equiv)	DCM	0.05	-78	3	92
8[b]	BF_3·Et_2O (1 equiv)	DCM	0.05	0	1.5	92
9	BF_3·Et_2O (1 equiv)	DCM	0.005	0	1.5	93
10	BF_3·Et_2O (1 equiv)	Tol	0.05	0	1.5	90
11	BF_3·Et_2O (1 equiv)	Hex	0.05	0	1.5	90
12	$AlEtCl_2$ (1 equiv)	DCM	0.05	0	1.5	32
13	TMSOTf (1 equiv)	DCM	0.05	0	1.5	92
14	TBSOTf (1 equiv)	DCM	0.05	0	1.5	89

[a] Reactions were performed with 2c in solvent at indicated concentration by addition of Lewis acid at indicated temperature (0.1 mmol). [b] Determined by HPLC using Lux 5μ cellulose-4 column. [b] Entry 8 was selected as the optimal condition.

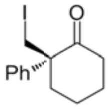

(S)-2-(iodomethyl)-2-phenylcyclohexanone (3c)

To a stirred solution of **2c** (31.4 mg, 0.1 mmol) in 2ml CH_2Cl_2 under nitrogen atmosphere at 0°C was added boron trifluoride etherate (10 ul, 0.1 mmol). Two hours later, saturated aqueous $NaHCO_3$ was added to the solution, and the resulting mixture was extracted with CH_2Cl_2. The organic layer was washed with brine, dried over Na_2SO_4, and evaporated in vacuo[3b]. The residue was purified by preparative column chromatography (ethyl acetate/petroleum ether = 1/30, v/v) and gave **3c** as a red brown liquid (29.1 mg, 93% yield, 93% *ee* for 0.1 mmol scale). The enantiomeric purity was determined by HPLC analysis (Lux 5μ cellulose-4 column, hexane/*i*-PrOH 98:2, 0.7 mL/min, t_{major} = 10.21 min, t_{minor}= 11.52 min). ^1H NMR (500 MHz, $CDCl_3$) δ 7.37 (t, *J* = 7.5 Hz, 2H), 7.31 (d, *J* = 7.5 Hz, 1H), 7.20 (d, *J* = 7.5 Hz, 2H), 3.61 (d, *J* = 10.0 Hz, 1H), 3.37 (d, *J* = 10.0 Hz, 1H), 2.81 (dq, *J* = 15.0, 3.0 Hz, 1H), 2.34-2.25 (m, 2H), 1.96-1.87 (m, 2H), 1.80-1.74 (m, 2H), 1.74-1.68 (m, 3H). ^{13}C NMR (126 MHz, $CDCl_3$) δ 210.2, 138.2, 129.1, 127.7, 126.8, 56.2, 40.2, 36.3, 27.8, 21.8, 19.5; IR (neat) 2939, 1704, 1448, 701 cm^{-1}; HRMS (ESI+) exact mass calcd for $C_{13}H_{16}IO$: m/z 315.0246 ($[M+H]^+$), found: m/z 315.0247; $[\alpha]_D^{23.5}$ = 104.7 (*c* 0.44, $CHCl_3$).

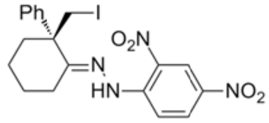

(*S*,*E*)-1-(2,4-dinitrophenyl)-2-(2-(iodomethyl)-2-phenylcyclohexy lidene)hydrazine (4)

To a solution of **3c** (31.3 mg, 0.1 mmol) in EtOH/H_2O (1.8 mL/0.6 mL), 2,4-dinitrophenylhydrazine (36.4, mg, 0.12 mmol, wetted with 50% water) and con.H_2SO_4 (0.2 mL) were added. The mixture was stirred at rt for 4 h. Then H_2O (3 mL) was added. The organic layer was extracted with ethyl acetate and washed with saturated $NaHCO_3$ (aq.), brine, dried over Na_2SO_4, filtered and then concentrated[1d]. The residue was purified by silica gel column chromatography (ethyl acetate/petroleum ether = 1:15, v/v) to afford the title product as a yellow solid (42.9 mg, 87% yield, 94% *ee*). The enantiomeric purity was determined by HPLC analysis (ChiralPak ID3 column, hexane/*i*-PrOH 9:1, 0.7 mL/min, t_{major} = 12.51 min, t_{minor}= 11.34 min). ^{1}H NMR (500 MHz, $CDCl_3$) δ 11.41 (s, 1H), 9.17 (s, 1H), 8.39 (d, *J* = 10.0 Hz, 1H), 8.22 (d, *J* = 10.0 Hz, 1H), 7.38 (t, *J* = 7.5 Hz, 2H), 7.32 (t, *J* = 7.5 Hz, 1H), 7.38 (d, *J* = 7.5 Hz, 2H), 3.84 (d, *J* = 10.0 Hz, 1H), 3.48 (d, *J* = 10.0 Hz, 1H), 2.82 (d, *J* = 15.0 Hz, 1H), 2.68 (d, *J* = 15.0 Hz, 1H), 2.09-2.03 (m, 1H), 1.98-1.91 (m, 2H), 1.85-1.78 (m, 1H), 1.73-1.66 (m, 1H), 1.59-1.57 (m, 1H); ^{13}C NMR (126 MHz, $CDCl_3$) δ 160.1, 145.6, 139.8, 138.1, 130.3, 129.2, 127.7, 127.0, 123.5, 117.0, 50.3, 36.3, 29.7, 26.2, 25.1, 21.7, 19.8; IR (neat) 3323, 2934, 1617, 1336, 7442 cm^{-1};HRMS (ESI+) exact mass calcd for $C_{19}H_{20}O_4N_4I$: m/z 495.0518 ([M+H]$^+$), found: m/z 495.0524. $[\alpha]_D^{23.5}$ = 126.4 (*c* 0.55, $CHCl_3$).

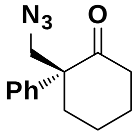

5

To a flask charged with NaN_3 (7.8 mg, 0.12 mmol, in 1mL DMSO) was added a solution of **5** (31.2mg, 0.1 mmol) in 1ml DMSO under nitrogen. The mixture was stirred at 80 °C for 5 hours and cooled to room temperature. Then the reaction was washed with water for 3 times, dried under reduced pressure[3a]. The residue was purified by preparative column chromatography (ethyl acetate/petroleum ether = 1:30, v/v) and gave **6** as a colorless liquid (20.3 mg, 86% yield, 92% *ee*). The enantiomeric purity was determined by HPLC analysis (Lux 5μ cellulose-4 column, hexane/iPrOH 98:2, 0.7 mL/min, t_{major} = 10.45 min, t_{minor}= 11.46 min). ^{1}H NMR (500 MHz, $CDCl_3$) δ 7.39 (t, *J* = 7.5 Hz, 2H), 7.34–7.28 (m, 1H), 7.22 (d, *J* = 7.5 Hz, 2H), 3.63 (d, *J* = 12.5 Hz, 1H), 3.34 (d, *J* = 12.5 Hz, 1H), 2.74 (dd, *J* = 14.4, 2.5 Hz, 1H), 2.39-2.26 (m, 2H), 2.00-1.86 (m, 2H), 1.82-1.66 (m, 3H); ^{13}C NMR (126 MHz, $CDCl_3$) δ 211.8, 137.9, 129.2, 127.7, 126.9, 59.9, 58.0, 39.9, 32.8, 27.9, 21.2; HRMS (ESI+) exact mass calcd for $C_{13}H_{16}N_3O$: m/z 230.1293 ([M+H]$^+$), found: m/z 230.1297; IR (neat) 2939, 2102, 1708, 702 cm^{-1}; $[\alpha]_D^{23.5}$ = 136.1 (*c* 0.34, $CHCl_3$).

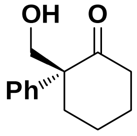

6

A flask charged with $AgBF_4$ (0.13 mmol) in CH_3NO_2 (1mL) under nitrogen atmosphere was added **5** (31.4mg, 0.1mmol, in 0.4 mL DMF). Then stirred at 40 °C for 4.5 h in dark and filtered through a short

pad of celite. The filtrate was washed successively with water, brine, and then dried. The obtained residue was dissolved in methanol, added Na_2CO_3 and stirred at room temperature for 1h. After the addition of water (2 ml), the solution was extracted with DCM. The organic layers were washed with water, brine and dried over Na_2SO_4. Evaporation of the solvent gave the corresponding alcohol, which could be purified by chromatography (ethyl acetate/petroleum ether = 1:6, v/v) as a colorless liquid (23.2 mg, 74% yield, 91% *ee*). The enantiomeric purity was determined by HPLC analysis (Lux 5μ cellulose-4 column, hexane/iPrOH 4:1, 0.7 mL/min, t_{major}= 9.14 min, t_{minor}= 8.19 min). ^{1}H NMR (500 MHz, $CDCl_3$) δ 7.31-7.16 (m, 5H), 3.85 (s, 1H), 3.14 (d, *J* = 13.5 Hz, 1H), 2.97 (d, *J* = 13.5 Hz, 1H), 2.73-2.66 (m, 1H), 2.56-2.53 (m, 1H), 2.26-2.11 (m, 2H), 1.91-1.86 (m, 2H), 1.69-1.65(m, 2H); ^{13}C NMR (126 MHz, $CDCl_3$) δ 213.2, 135.3, 130.0, 128.2, 127.0, 79.3, 43.3, 40.4, 38.6, 28.0, 22.8; HRMS (ESI+) exact mass calcd for $C_{13}H_{16}NaO_2$: m/z 227.1048 ($[M+Na]^+$), found: m/z 227.1049; IR (neat) 3448, 2920, 1709, 702, 669 cm-1; $[\alpha]_D^{23.5}$ = 50.0 (*c* 0.42, $CHCl_3$)

5.2 one-pot iodo-etherification/Wagner-Meerwein rearrangement reaction

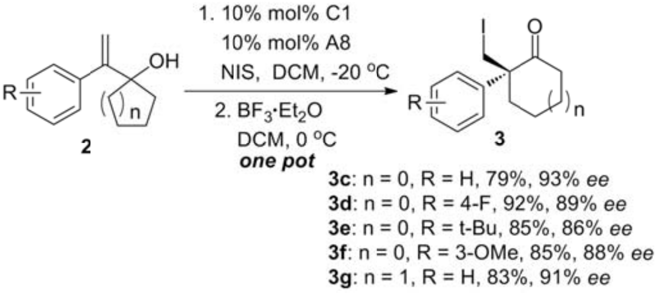

General procedure F: In a Schlenk tube, ion pair catalyst **C1** (0.01 mmol), ammonium salt **A8** (0.01 mmol), NIS (0.12 mmol) and CH_2Cl_2 (1 mL) was added, then the reaction mixture was cooled to -20 °C. Allyl alcohol **1** (0.1 mmol) in 1 mL CH_2Cl_2 was added dropwise. As the substrate disappeared indicated by TLC, boron trifluoride etherate (0.1 mmol) was added dropwise to the mixture at 0 °C. After 2.5 h, the solvent was removed under reduced pressure, and the residue was purified by silica gel column chromatography (ethyl acetate/petroleum ether = 1:25, v/v) to afford the product.

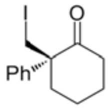

(S)-2-(iodomethyl)-2-phenylcyclohexanone (3c)

Prepared according to the general procedure **F** with **2c** (31.4 mg, 0.1 mmol) as a pale yellow liquid (24.8 mg, 79% yield, 93% *ee*). The enantiomeric purity was determined by HPLC analysis (Lux 5μ cellulose-4 column, hexane/*i*-PrOH 98:2, 0.7 mL/min, t_{major} = 10.59 min, t_{minor}= 12.38 min). The product shows no difference in spectra with those of **3c** obtained by stepwise reaction.

2.7mmol scale synthesis of 3c: In a Schlenk tube, ion pair catalyst **C1** (150 mg, 0.014 mmol), ammonium salt **A8** (58 mg, 0.014 mmol), NIS (720 mg, 3.2 mmol) and CH_2Cl_2 (50 mL) was added, then the reaction mixture was cooled to -20 °C. Allyl alcohol **1** (500 mg, 2.66 mmol) in 5 mL CH_2Cl_2 was added dropwise. After 39h boron trifluoride etherate (120 mg, 104 ul, 0.9 mmol,) was added dropwise to the mixture at 0 °C. After 2.5 h the reaction was quenched with saturated $Na_2S_2O_3$. The organic layer was separated and organic layer washed with CH_2Cl_2 for two times. Combined the organic layer, washed with brine, dried over Na_2SO_4 and evaporated under reduced pressure. The residue was purified by silica gel column chromatography (ethyl acetate/petroleum ether = 1:100, v/v) to afford the product (685 mg, 2.18 mmol, 82% yield, 92% *ee*). Enantiomeric excess was found to be 92%

measured by chiral HPLC (Lux 5μ cellulose-4 column, hexane/*i*-PrOH 98:2, 0.7 mL/min, t_{major} = 10.47 min, t_{minor}= 12.18 min).

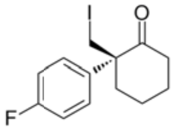

(S)-2-(4-fluorophenyl)-2-(iodomethyl)cyclohexanone (3d)

Prepared according to the general procedure **F** with **2ce** (22.3 mg, 0.1 mmol) as a white solid (32.2 mg, 92% yield, 89% *ee*). The enantiomeric purity was determined by HPLC analysis (ChiralPak IF-3 column, hexane/iPrOH 98:2, 0.7 mL/min, t_{major} = 9.96 min, t_{minor}= 13.29 min). ^{1}H NMR (500 MHz, $CDCl_3$) δ 7.18 -7.16 (m, 2H), 7.08-7.04 (m, 2H), 3.57 (dd, *J* = 10.0, 1.5 Hz, 1H), 3.37 (dd, *J* = 10.0, 1.5 Hz, 1H), 2.82-2.75 (m, 1H), 2.31-2.26 (m, 2H), 1.96-1.68 (m, 5H); ^{13}C NMR (126 MHz, $CDCl_3$) δ 210.1, 162.2 (d, *J* = 247.7 Hz), 134.1 (d, *J* = 3.4 Hz), 128.7 (d, *J* = 8.1 Hz), 116.1 (d, *J* = 21.4 Hz), 55.6, 40.1, 36.5, 27.8, 21.8, 19.7; ^{19}F NMR (376 MHz, $CDCl_3$) δ -114.3; IR (neat) 2942, 1721, 1443, 678 cm^{-1}; HRMS (ESI+) exact mass calcd for $C_{13}H_{15}FIO^+$: m/z 333.0152 ([M+H]$^+$), found: m/z 333.0152. $[\alpha]_D^{23.5}$ = 87.3 (*c* 0.94, $CHCl_3$).

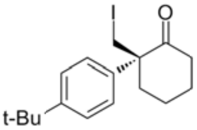

(S)-2-(4-(tert-butyl)phenyl)-2-(iodomethyl)cyclohexanone (3e)

Prepared according to the general procedure **F** with **2cb** (37.0 mg, 0.1 mmol) as a white solid (31.5 mg, 85% yield, 86% *ee*). The enantiomeric purity was determined by HPLC analysis (Lux 5μ cellulose-4 column, hexane/*i*-PrOH 99:1, 0.7 mL/min, t_{major} = 7.79 min, t_{minor}= 9.01 min). ^{1}H NMR (500 MHz, $CDCl_3$) δ 7.36 (d, *J* = 7.5 Hz, 2H), 7.11 (d, *J* = 7.5 Hz, 2H), 3.63 (d, *J* = 10.0 Hz, 1H), 3.29 (d, *J* = 10.0 Hz, 1H), 2.81-2.71 (m, 1H), 2.35-2.26 (m, 2H), 2.00-1.87 (m, 2H), 1.82-1.74 (m, 2H), 1.74-1.64 (m, 2H), 1.30 (s, 9H); ^{13}C NMR (126 MHz, $CDCl_3$) δ 210.2, 150.7, 134.9, 126.4, 126.0, 55.8, 40.0, 36.2, 34.5, 31.3, 27.7, 21.8, 19.2; IR (neat) 2933, 1692, 1447, 683 cm^{-1}; HRMS (ESI+) exact mass calcd for $C_{17}H_{24}IO^+$: m/z 371.0872 ([M+H]$^+$), found: m/z 371.0876. $[\alpha]_D^{23.5}$ = 69.5 (*c* 0.97, $CHCl_3$).

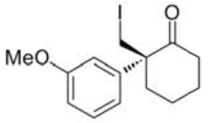

(S)-2-(iodomethyl)-2-(3-methoxyphenyl)cyclohexanone (3f)

Prepared according to the general procedure **F** with **2cc** (34.4 mg, 0.1 mmol) as a white solid (27.3 mg, 85% yield, 88% *ee*). The enantiomeric purity was determined by HPLC analysis (Lux 5μ cellulose-4 column, hexane/*i*-PrOH 99:1, 0.7 mL/min, t_{major} = 13.31 min, t_{minor}= 16.75 min). ^{1}H NMR (500 MHz, $CDCl_3$) δ 7.28 (t, *J* = 8.0 Hz, 1H), 6.84 (d, *J* = 8.0 Hz, 1H), 6.78 (d, *J* = 8.0 Hz, 1H), 6.74 (s, 1H), 3.80 (s, 3H), 3.59 (d, *J* = 10.0 Hz, 1H), 3.36 (d, *J* = 10.0 Hz, 1H), 2.81-2.74 (m, 1H), 2.35-2.27 (m, 2H), 1.98-1.86 (m, 2H), 1.83-1.77 (m, 2H), 1.74-1.63 (m, 1H).; ^{13}C NMR (126 MHz, $CDCl_3$) δ 210.0, 160.1, 139.7, 130.1, 119.1, 113.2, 112.5, 56.1, 55.3, 40.2, 36.4, 27.7, 21.8, 19.2; IR (neat) 2937, 1712, 1437, 692 cm^{-1}; HRMS (ESI+) exact mass calcd for $C_{14}H_{18}IO_2^+$: m/z 345.0351 ([M+H]$^+$), found: m/z 345.0352. $[\alpha]_D^{23.5}$ = 79.7 (*c* 0.92, $CHCl_3$).

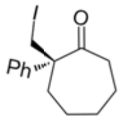

(S)-2-(iodomethyl)-2-phenylcycloheptanone (3g)

Prepared according to the general procedure **F** with **2d** (20.2 mg, 0.1 mmol) as a pale yellow liquid (27.2 mg, 83% yield, 91% *ee*).. The enantiomeric purity was determined by HPLC analysis (Lux 5μ cellulose-4 column, hexane/*i*-PrOH 98:2, 0.7 mL/min, t_{major} = 8.37 min, t_{minor}= 10.50 min). ^{1}H NMR (500 MHz, $CDCl_3$) δ 7.35 (t, *J* = 7.5 Hz, 2H), 7.29 (t, *J* = 7.5 Hz, 1H), 7.20 (d, *J* = 7.5 Hz, 2H), 3.81 (d, *J* = 10.0 Hz, 1H), 3.54 (d, *J* = 10.0 Hz, 1H), 2.59-2.51 (m, 2H), 2.35-2.33 (m, 1H), 2.23-2.18 (m, 1H), 1.96-1.93 (m, 1H), 1.86-1.81 (m, 2H), 1.62-1.44 (m, 3H), 1.27-1.22 (m, 1H); ^{13}C NMR (126 MHz, $CDCl_3$) δ 210.8, 140.5, 128.7, 127.7, 126.8, 58.6, 41.6, 33.1, 30.4, 26.8, 24.2, 19.3; IR (neat) 2936, 1702, 1048, 706 cm^{-1}; HRMS (ESI+) exact mass calcd for $C_{13}H_{16}IO$: m/z 329.0402 ([M+H]$^+$), found: m/z 329.0405. $[\alpha]_D^{23.5}$ = 94.7 (*c* 0.53, $CHCl_3$).

6. References

[1] (a) Yu, W. Y.; Bensimon, C.; Alper, H. *Chem. Eur. J.* **1997**, *3*, 417. (b) Tomioka, T.; Sankranti, R.; Yamada, T.; Clark, C. *Org. Lett.*, **2013**, *15*, 5099-5101. (c) Wang, B.; Wong, O. A.; Zhao, M.-X.; Shi, Y; *J. Org. Chem.*, **2008**, *73*, 9539-9543. (d) Qin, Y.; You, S. L. *Org. Lett.* **2014**, *16*, 1810. (e) Dabdoub, M. J.; Jacob, R. G.; Ferreira, J. T. B.; Dabdoub, V. B.; Marques, F. A. *Tetraheldron. Lett.* **1999**, *40*, 7159.

[2] Hamilton, G. L.; Kang, E. J.; Mba, M.; Toste, F. D. *Science*. **2007**, *317*, 496.

[3] (a) Suzuki, T.; Ota, Y.; Kasuya, Y.; Mutsuga, M.; Kawamura, Y.; Tsumoto, H.; Nakagawa, H.; Finn, M. G.; Miyata, N. *Angew. Chem., Int. Ed*. **2010**, *49*, 6817. (b) Kita, Y.; Matsuda, S.; Inoguchi, R.; Ganesh, J. K.; Fujioka, H. *J. Org. Chem.* **2006**, *71*, 5191 (c) Abad, A.; Agulló, C.; Cuñat, A. C.; Navarro, I.; *Synthesis,* **2005**, *19*, 3355. (d) Manas, M. M.; Teixido, M.; *J. Heterocycl. Chem.* **1988**, *25*, 1439.

7. Copies of spectrum

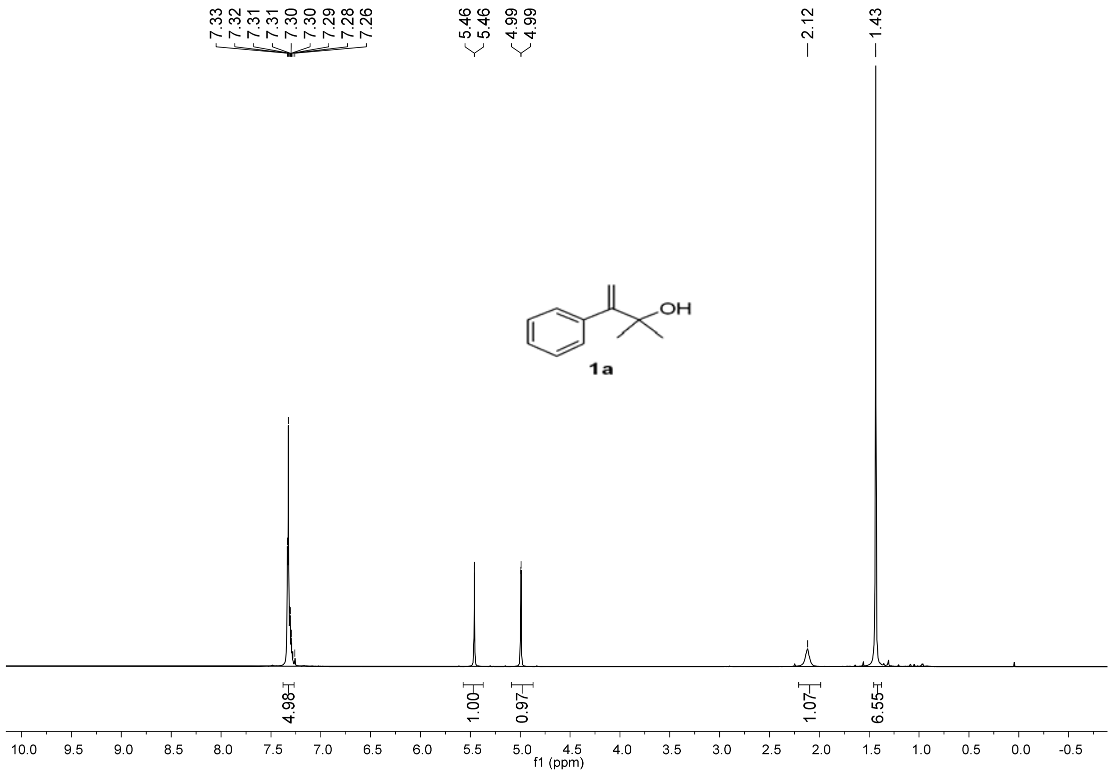

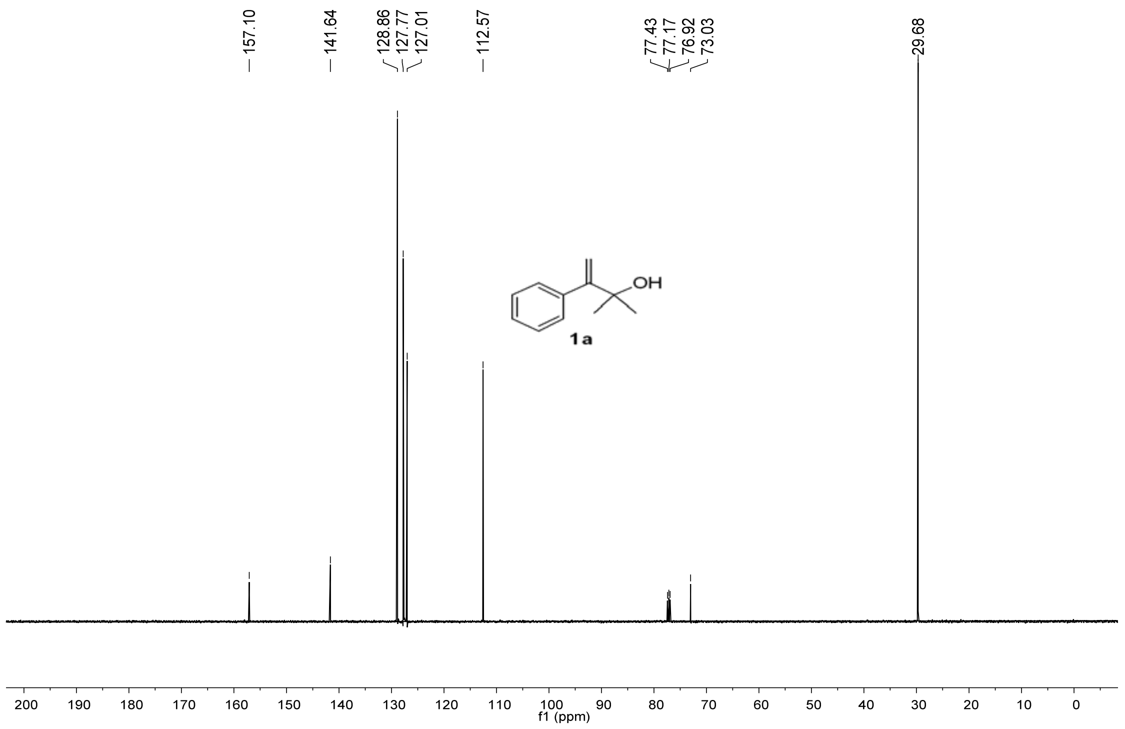

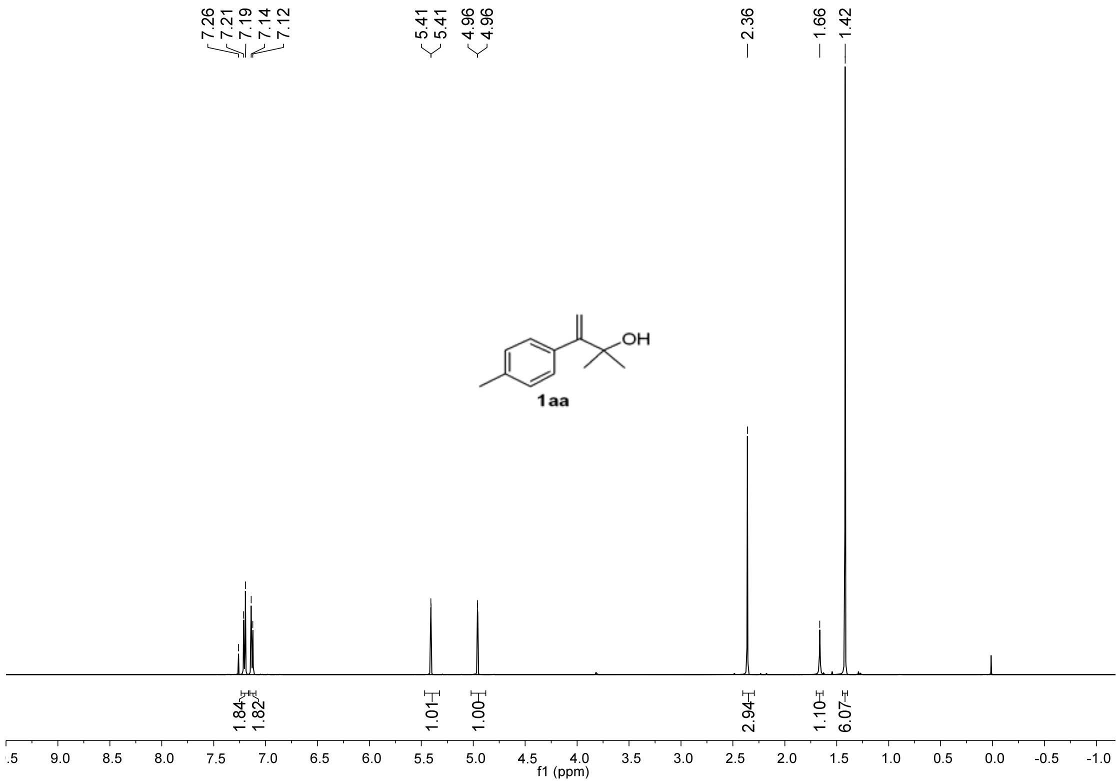
7.26
7.21
7.19
7.14
7.12
5.41
5.41
4.96
4.96
2.36
1.66
1.42
OH
1aa
1.84
1.82
1.01
1.00
2.94
1.10
6.07
f1 (ppm)

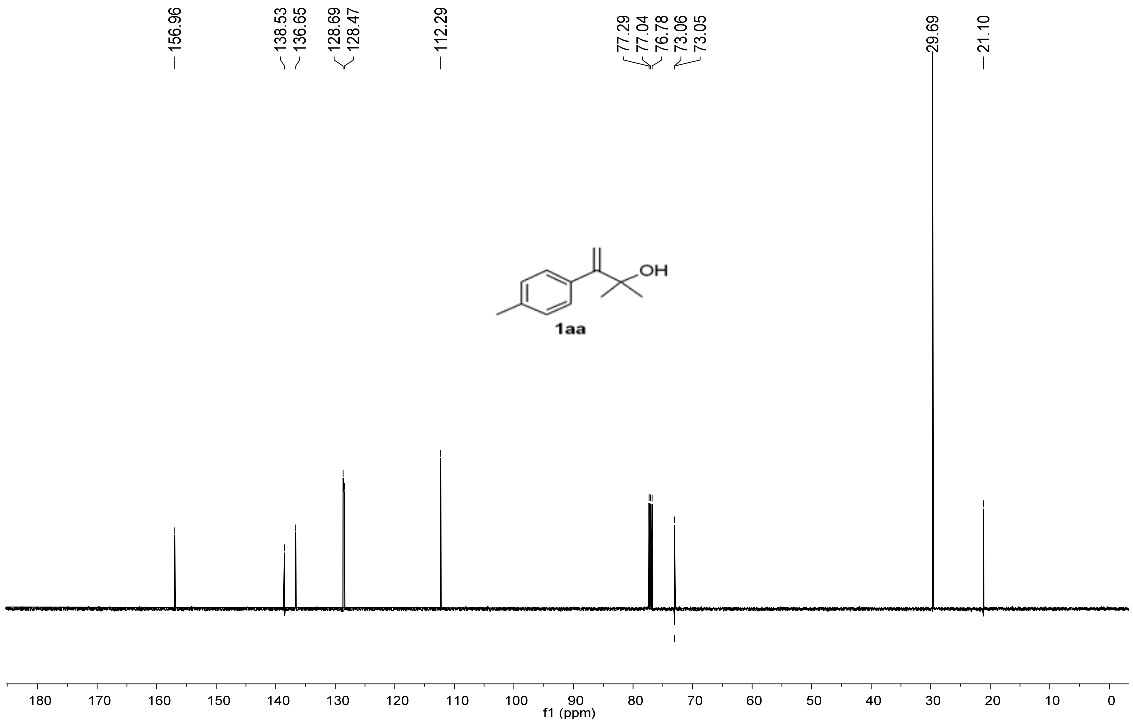
156.96
138.53
136.65
128.69
128.47
112.29
77.29
77.04
76.78
73.06
73.05
29.69
21.10
OH
1aa
f1 (ppm)

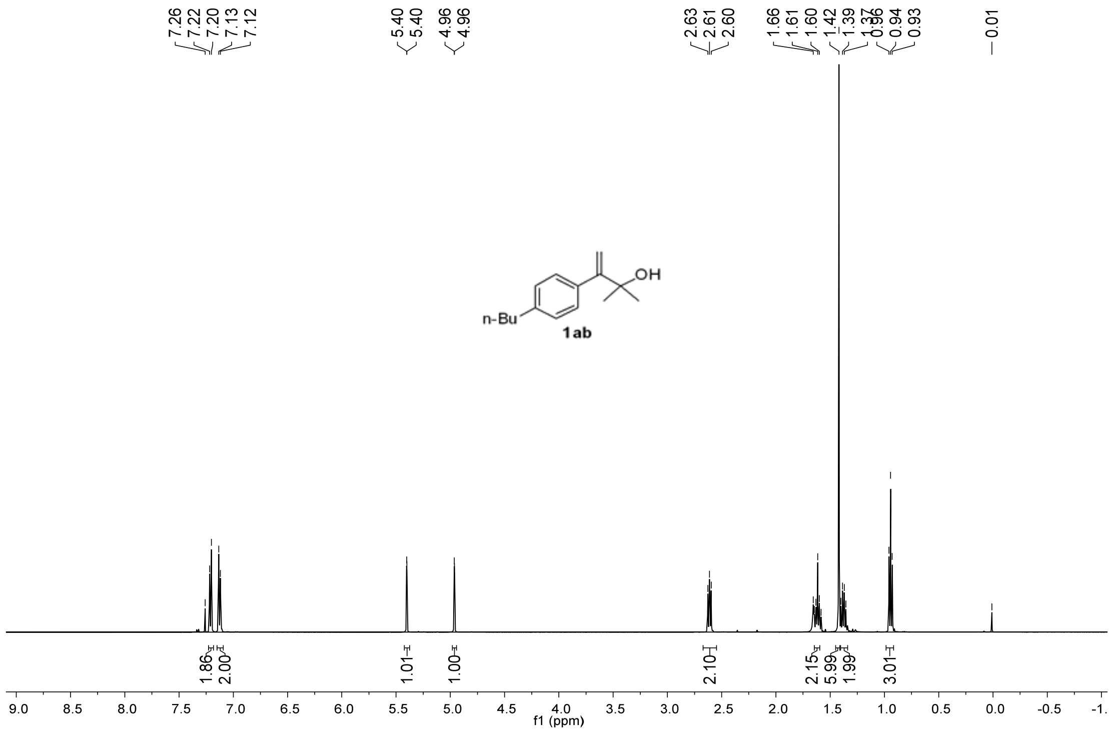
7.26
7.22
7.20
7.13
7.12
5.40
5.40
4.96
4.96
2.63
2.61
2.60
1.66
1.61
1.60
1.42
1.39
1.37
0.96
0.94
0.93
-0.01
OH
n-Bu
1ab
1.86
2.00
1.01
1.00
2.10
2.15
5.99
1.99
3.01
9.0 8.5 8.0 7.5 7.0 6.5 6.0 5.5 5.0 4.5 4.0 3.5 3.0 2.5 2.0 1.5 1.0 0.5 0.0 -0.5
f1 (ppm)

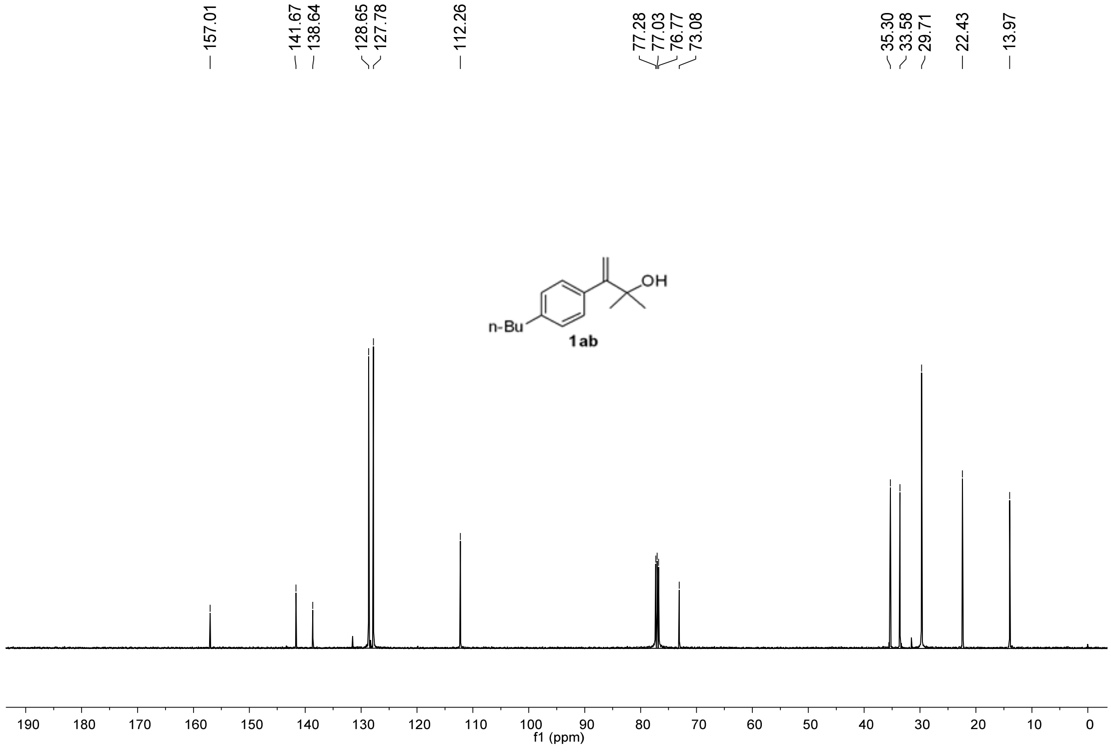
157.01
141.67
138.64
128.65
127.78
112.26
77.28
77.03
76.77
73.08
35.30
33.58
29.71
22.43
13.97
OH
n-Bu
1ab
190 180 170 160 150 140 130 120 110 100 90 80 70 60 50 40 30 20 10 0
f1 (ppm)

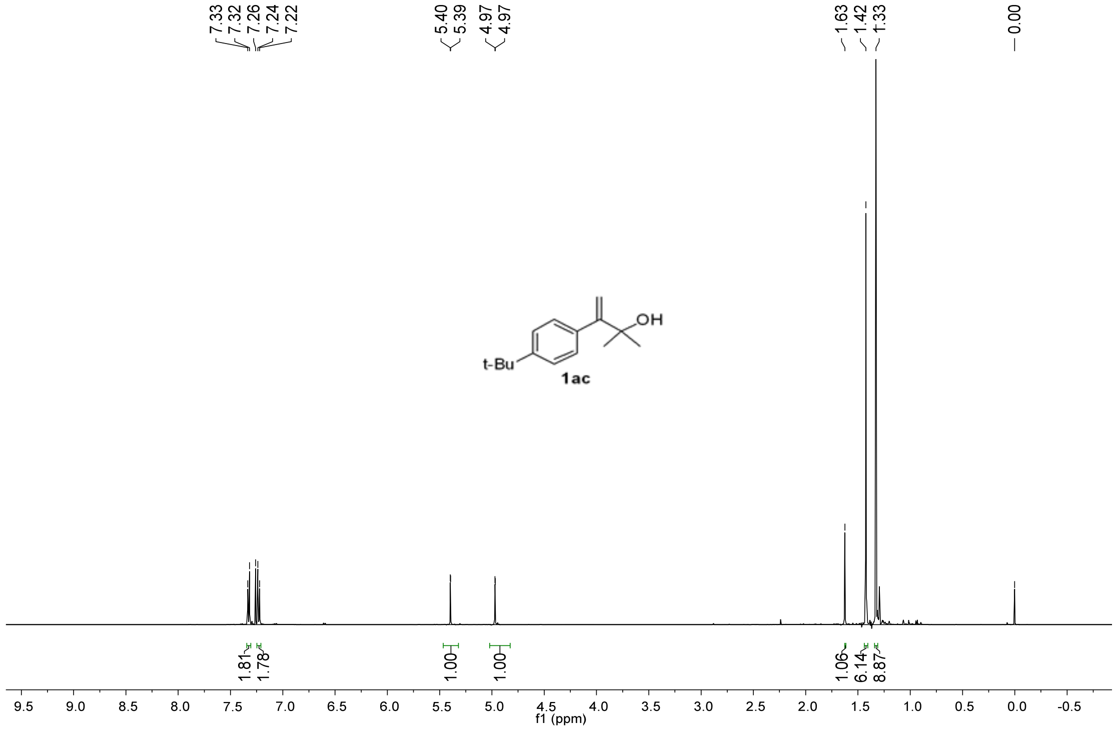
7.33
7.32
7.26
7.24
7.22
5.40
5.39
4.97
4.97
1.63
1.42
1.33
0.00
t-Bu
OH
1ac
1.81
1.78
1.00
1.00
1.06
6.14
8.87
9.5 9.0 8.5 8.0 7.5 7.0 6.5 6.0 5.5 5.0 4.5 4.0 3.5 3.0 2.5 2.0 1.5 1.0 0.5 0.0 -0.5
f1 (ppm)

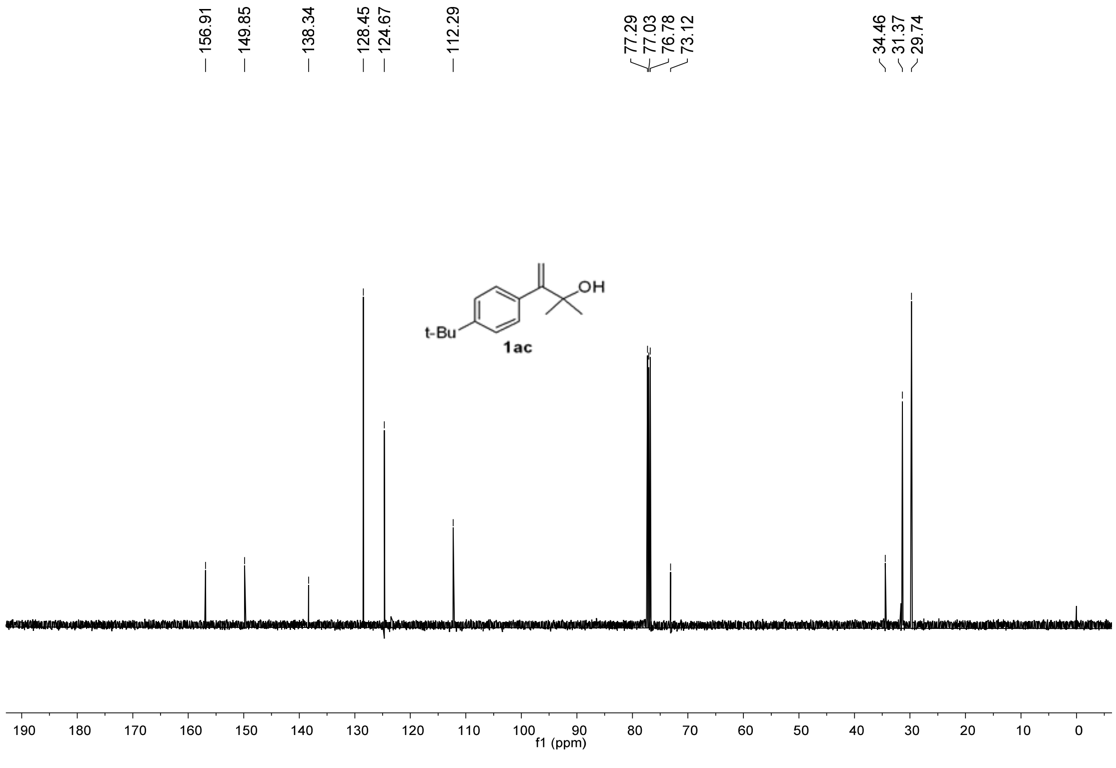
156.91
149.85
138.34
128.45
124.67
112.29
77.29
77.03
76.78
73.12
34.46
31.37
29.74
t-Bu
OH
1ac
190 180 170 160 150 140 130 120 110 100 90 80 70 60 50 40 30 20 10 0
f1 (ppm)

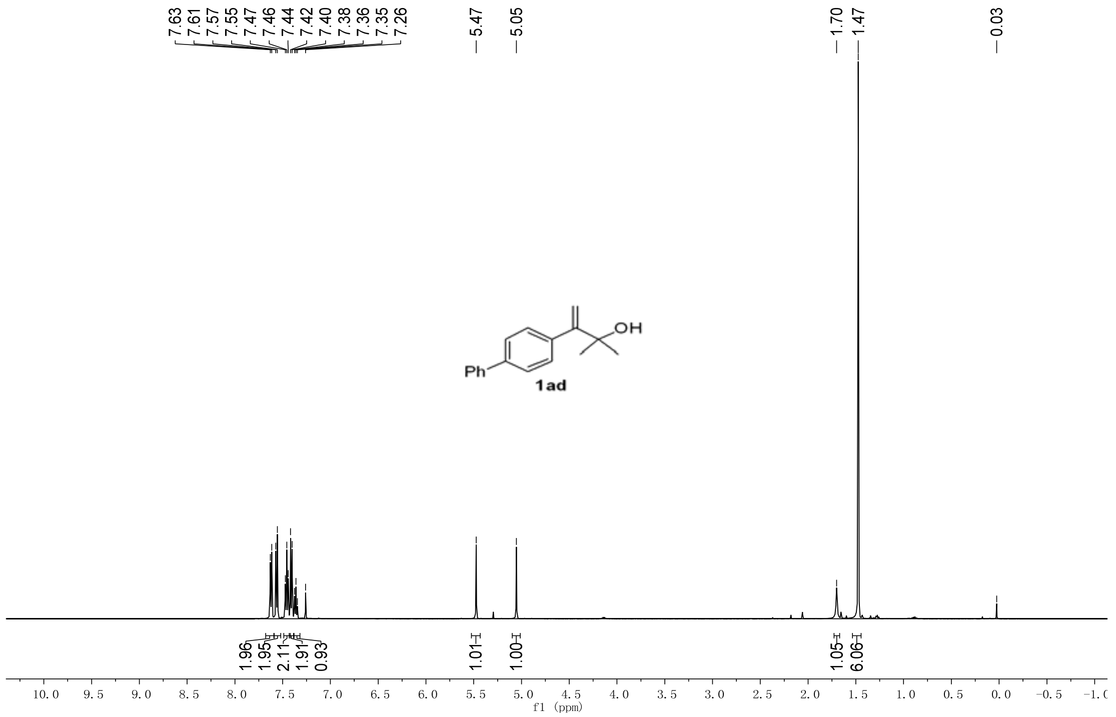

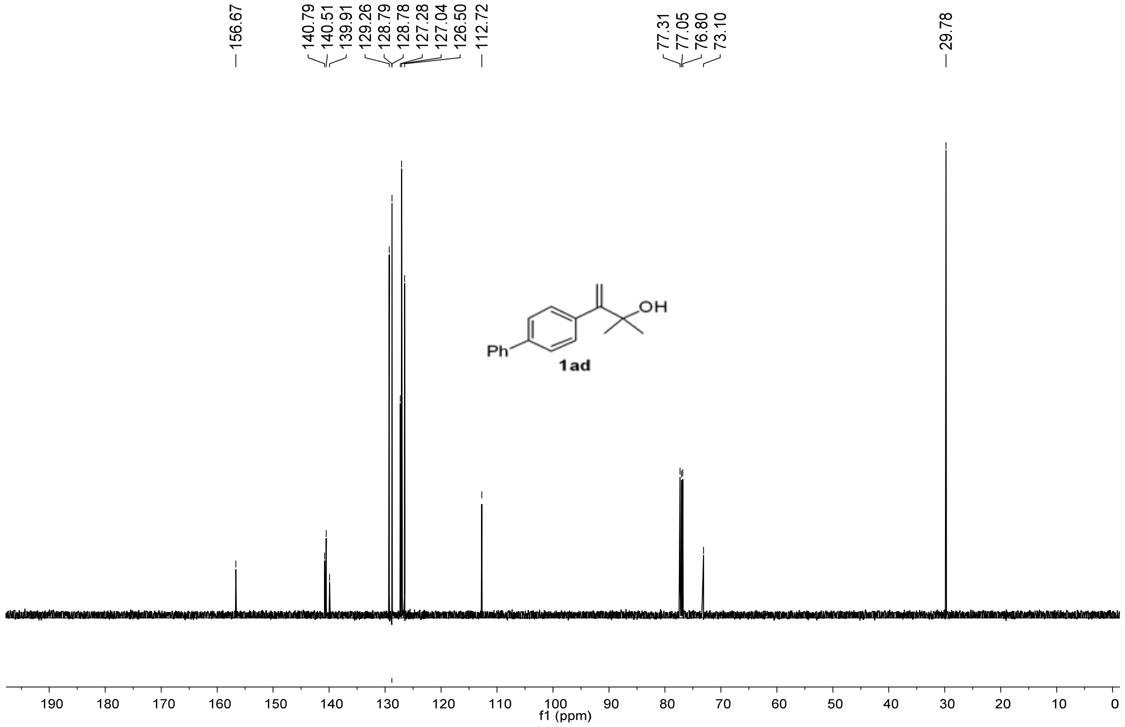

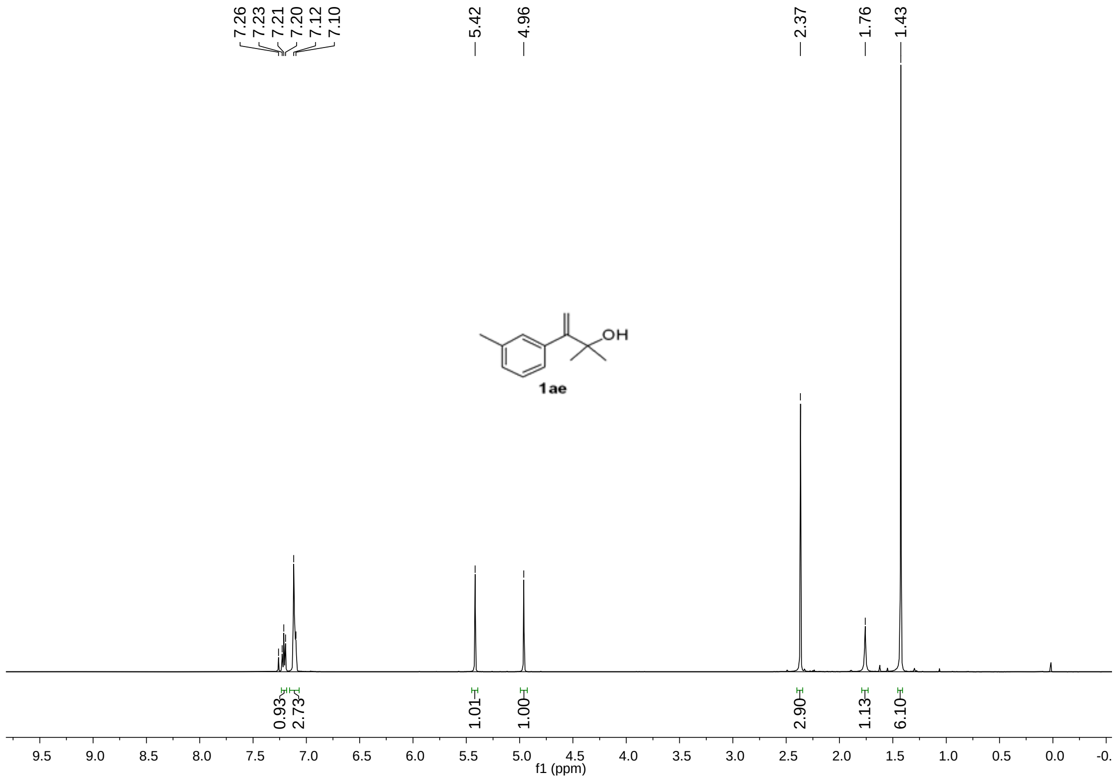
7.26
7.23
7.21
7.20
7.12
7.10
5.42
4.96
2.37
1.76
1.43
OH
1ae
0.93
2.73
1.01
1.00
2.90
1.13
6.10
f1 (ppm)

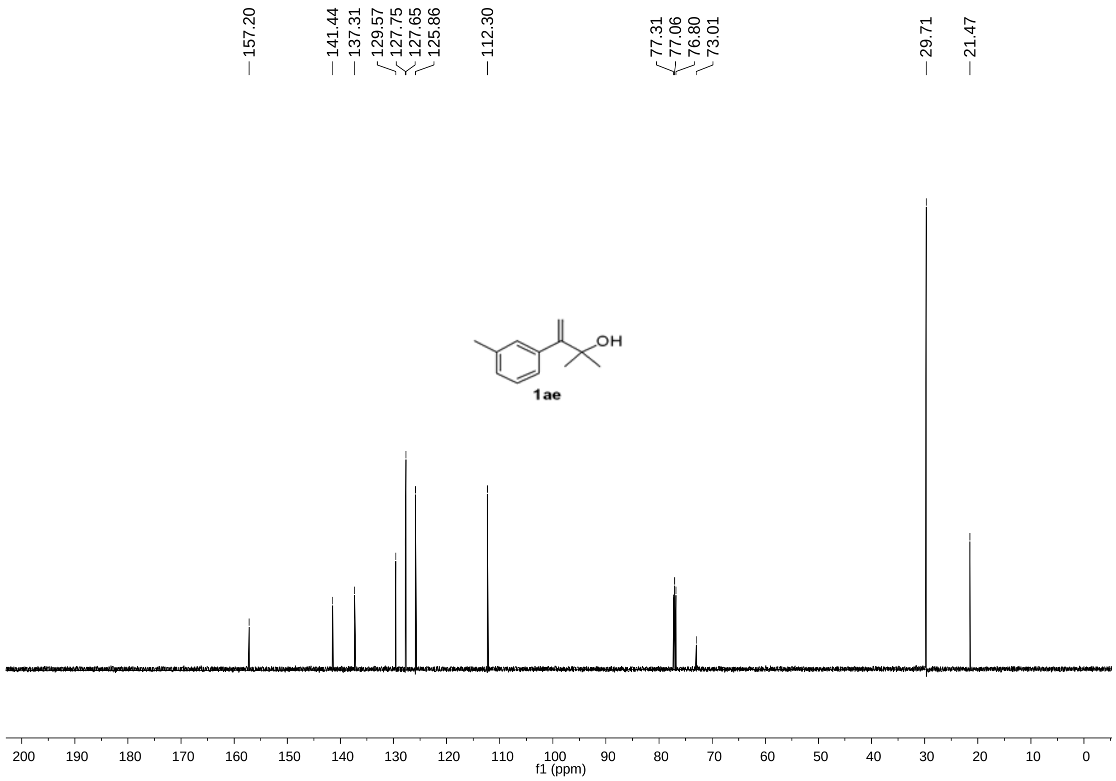
157.20
141.44
137.31
129.57
127.75
127.65
125.86
112.30
77.31
77.06
76.80
73.01
29.71
21.47
OH
1ae
f1 (ppm)

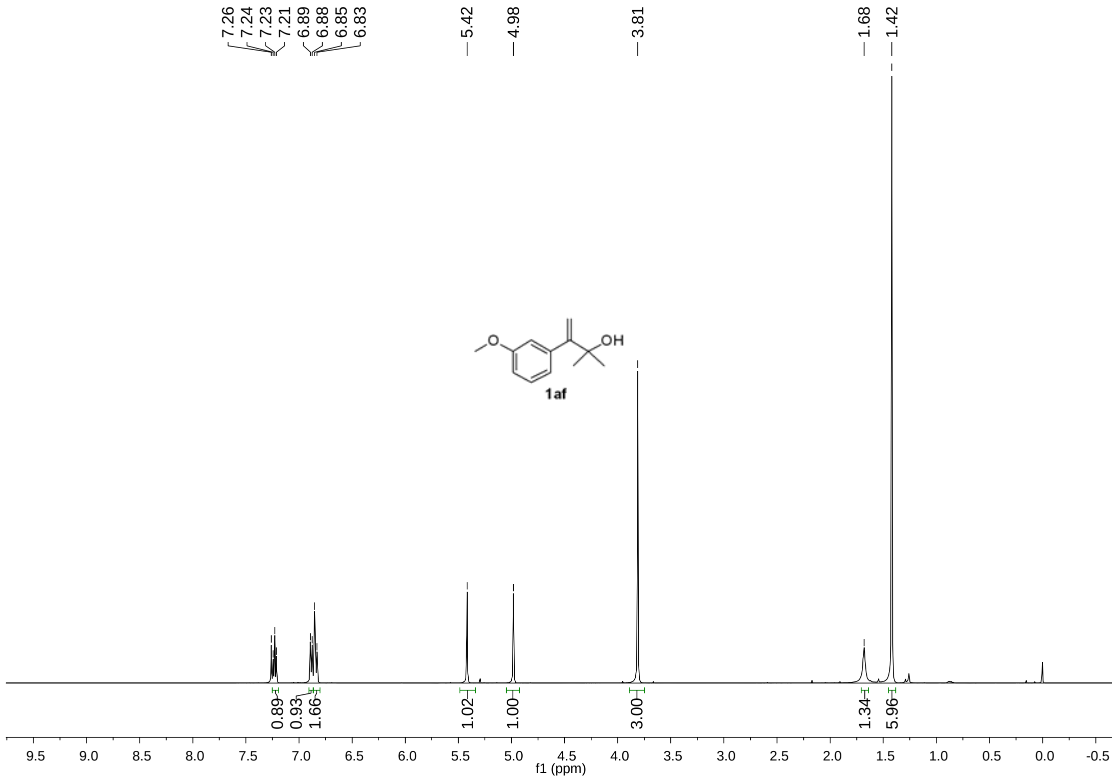
7.26
7.24
7.23
7.21
6.89
6.88
6.85
6.83
5.42
4.98
3.81
1.68
1.42
OH
1af
0.89
0.93
1.66
1.02
1.00
3.00
1.34
5.96
f1 (ppm)

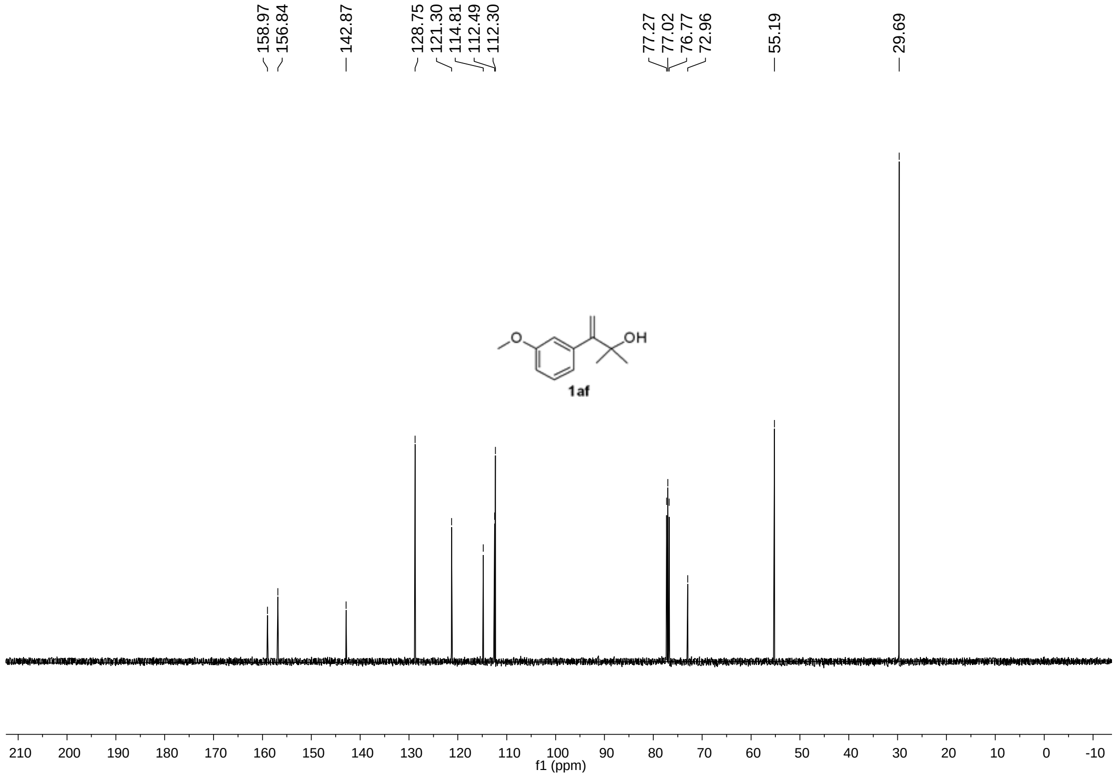
158.97
156.84
142.87
128.75
121.30
114.81
112.49
112.30
77.27
77.02
76.77
72.96
55.19
29.69
OH
1af
f1 (ppm)

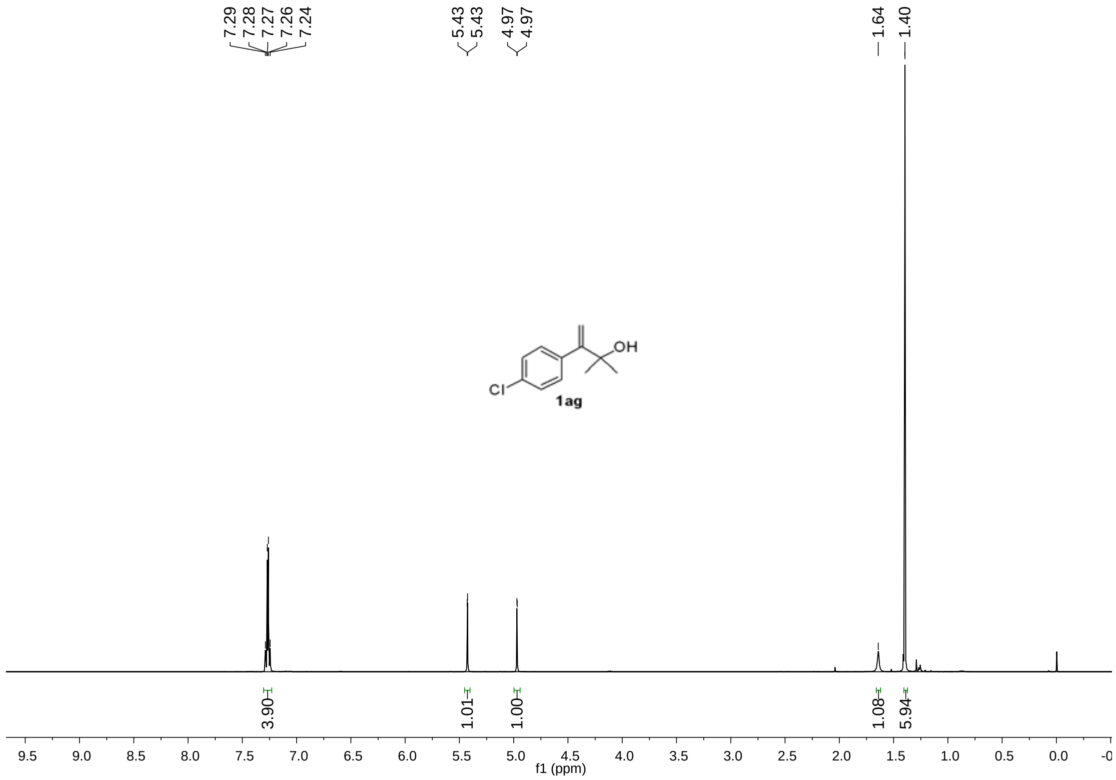
7.29
7.28
7.27
7.26
7.24
5.43
5.43
4.97
4.97
1.64
1.40
Cl
OH
1ag
3.90
1.01
1.00
1.08
5.94
f1 (ppm)

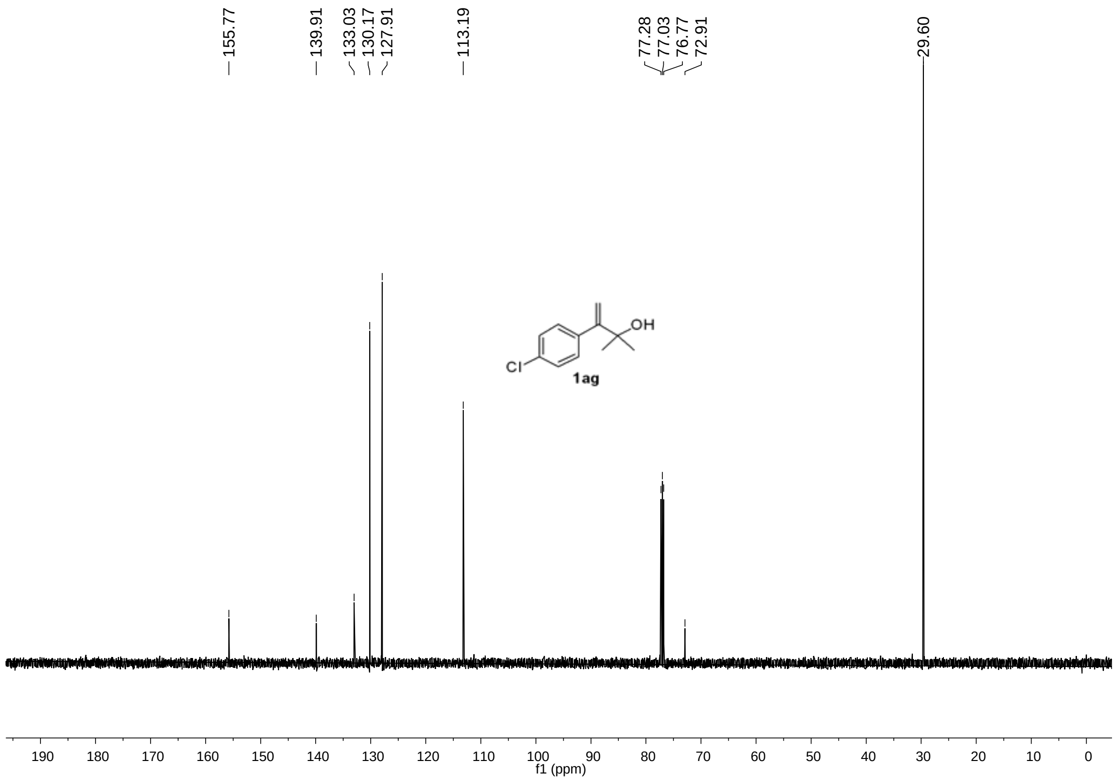
155.77
139.91
133.03
130.17
127.91
113.19
77.28
77.03
76.77
72.91
29.60
Cl
OH
1ag
f1 (ppm)

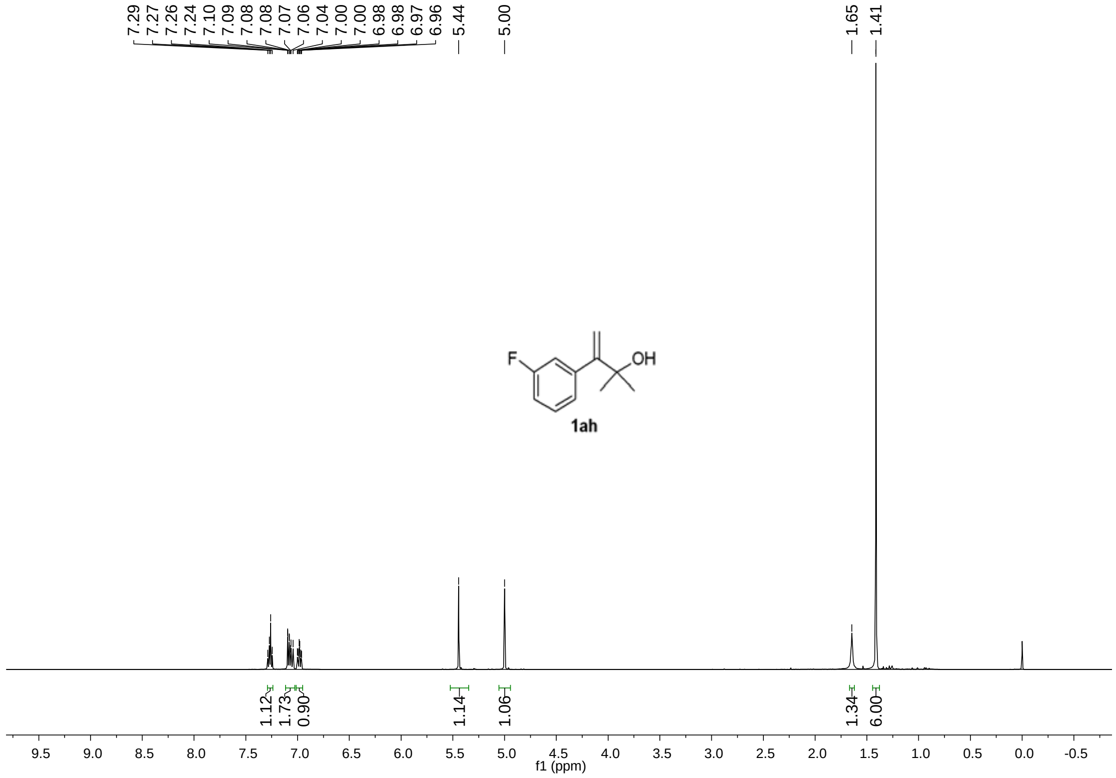

7.29
7.27
7.26
7.24
7.10
7.09
7.08
7.08
7.07
7.06
7.04
7.00
7.00
6.98
6.98
6.97
6.96
5.44
5.00
1.65
1.41
F
OH
1ah
1.12
1.73
0.90
1.14
1.06
1.34
6.00
f1 (ppm)

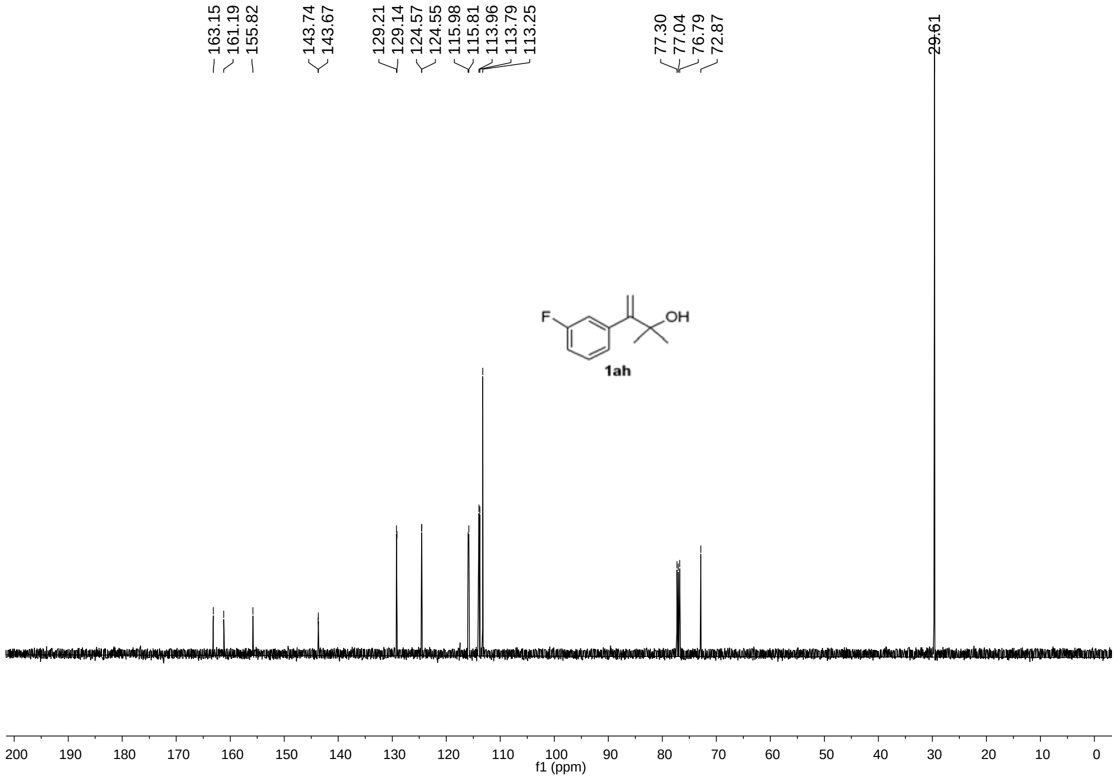

163.15
161.19
155.82
143.74
143.67
129.21
129.14
124.57
124.55
115.98
115.81
113.96
113.79
113.25
77.30
77.04
76.79
72.87
29.61
F
OH
1ah
f1 (ppm)

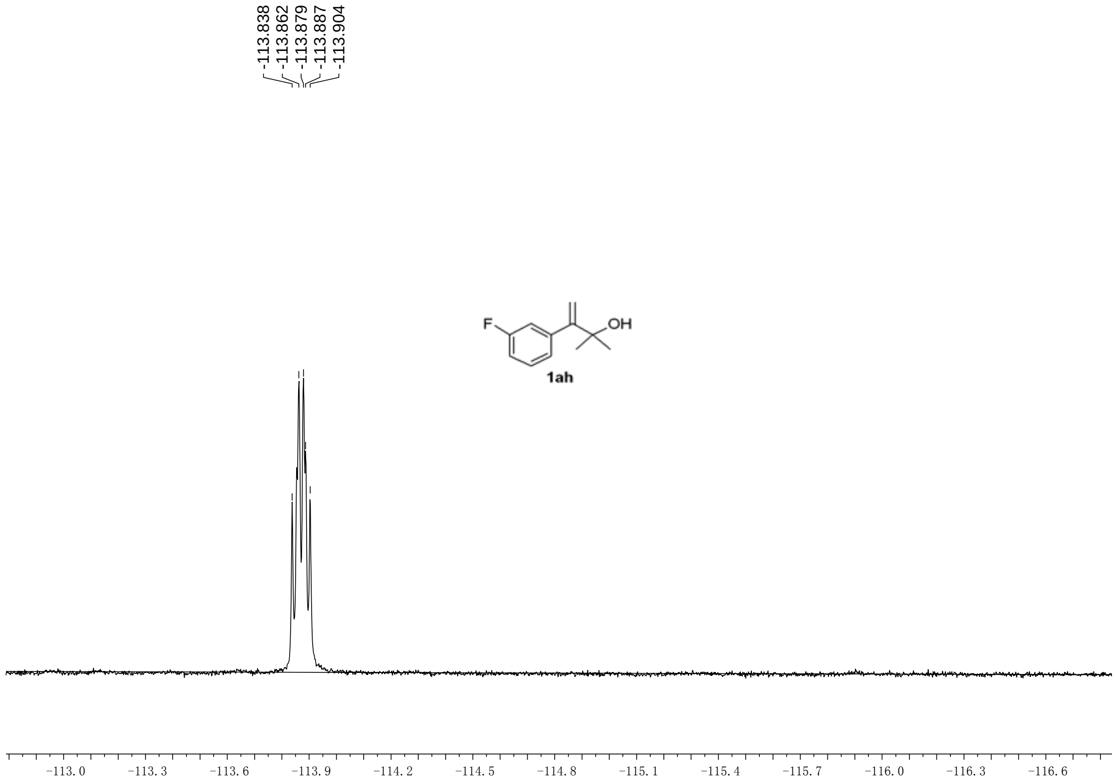
-113.838
-113.862
-113.879
-113.887
-113.904
F
OH
1ah
−113.0 −113.3 −113.6 −113.9 −114.2 −114.5 −114.8 −115.1 −115.4 −115.7 −116.0 −116.3 −116.6

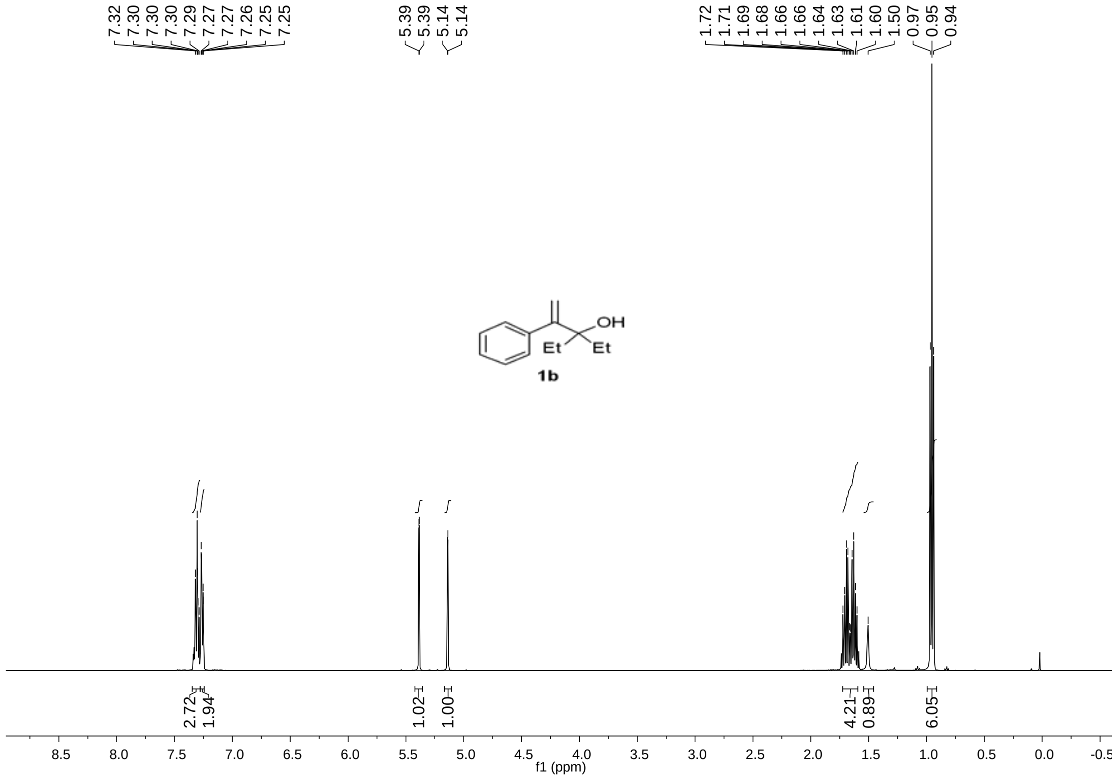
7.32
7.30
7.30
7.29
7.27
7.27
7.26
7.25
7.25
5.39
5.39
5.14
5.14
1.72
1.71
1.69
1.68
1.66
1.66
1.64
1.63
1.61
1.60
1.50
0.97
0.95
0.94
OH
Et
Et
1b
2.72
1.94
1.02
1.00
4.21
0.89
6.05
8.5 8.0 7.5 7.0 6.5 6.0 5.5 5.0 4.5 4.0 3.5 3.0 2.5 2.0 1.5 1.0 0.5 0.0 -0.5
f1 (ppm)

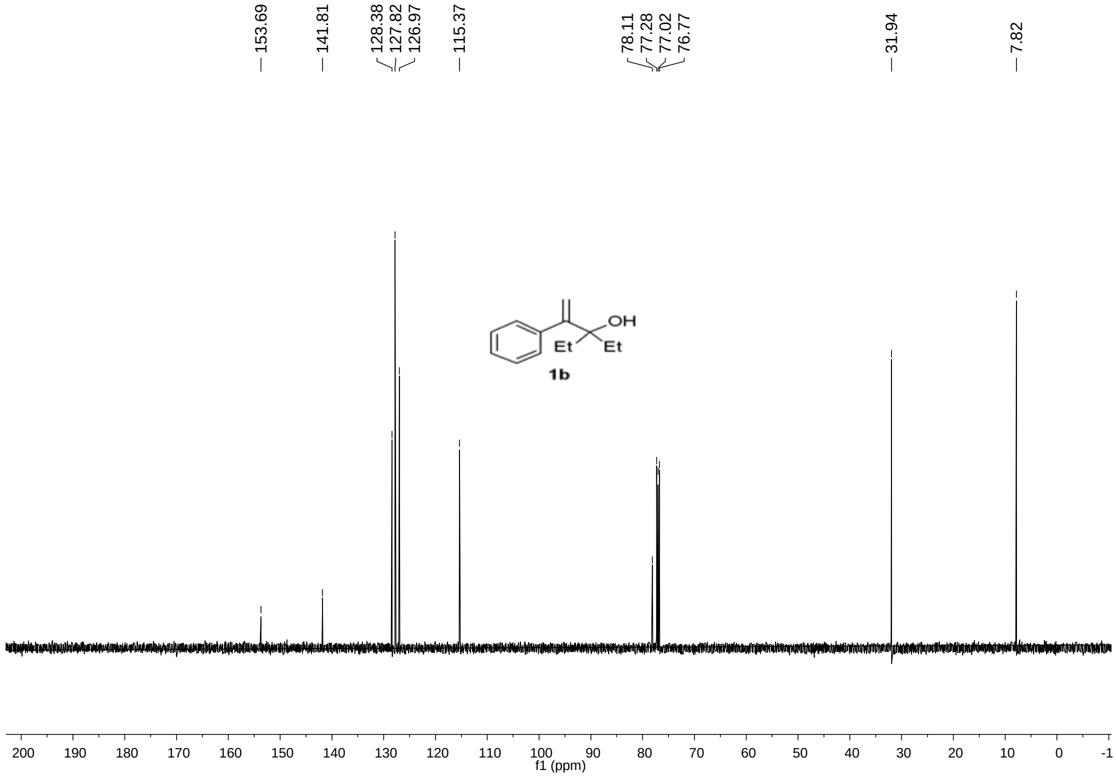

153.69
141.81
128.38
127.82
126.97
115.37
78.11
77.28
77.02
76.77
31.94
7.82
OH
Et
Et
1b
200 190 180 170 160 150 140 130 120 110 100 90 80 70 60 50 40 30 20 10 0
f1 (ppm)

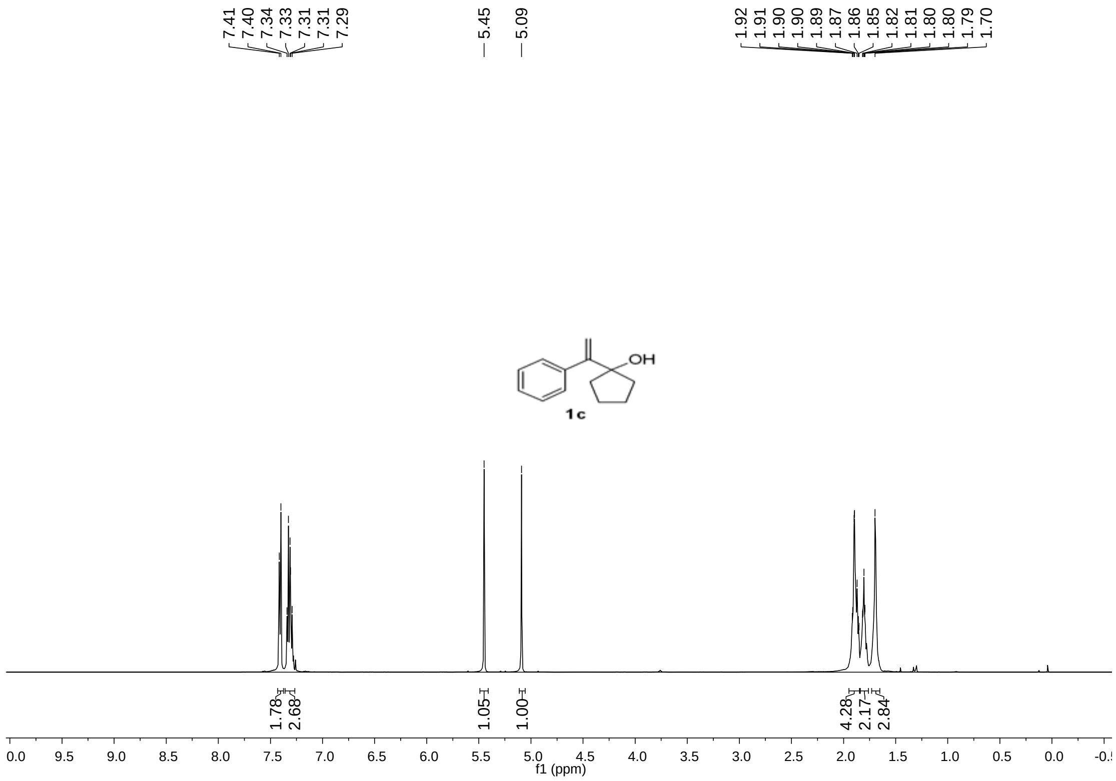

7.41
7.40
7.34
7.33
7.31
7.31
7.29
5.45
5.09
1.92
1.91
1.90
1.90
1.89
1.87
1.86
1.85
1.82
1.81
1.80
1.80
1.79
1.70
OH
1c
1.78
2.68
1.05
1.00
4.28
2.17
2.84
9.5 9.0 8.5 8.0 7.5 7.0 6.5 6.0 5.5 5.0 4.5 4.0 3.5 3.0 2.5 2.0 1.5 1.0 0.5 0.0
f1 (ppm)

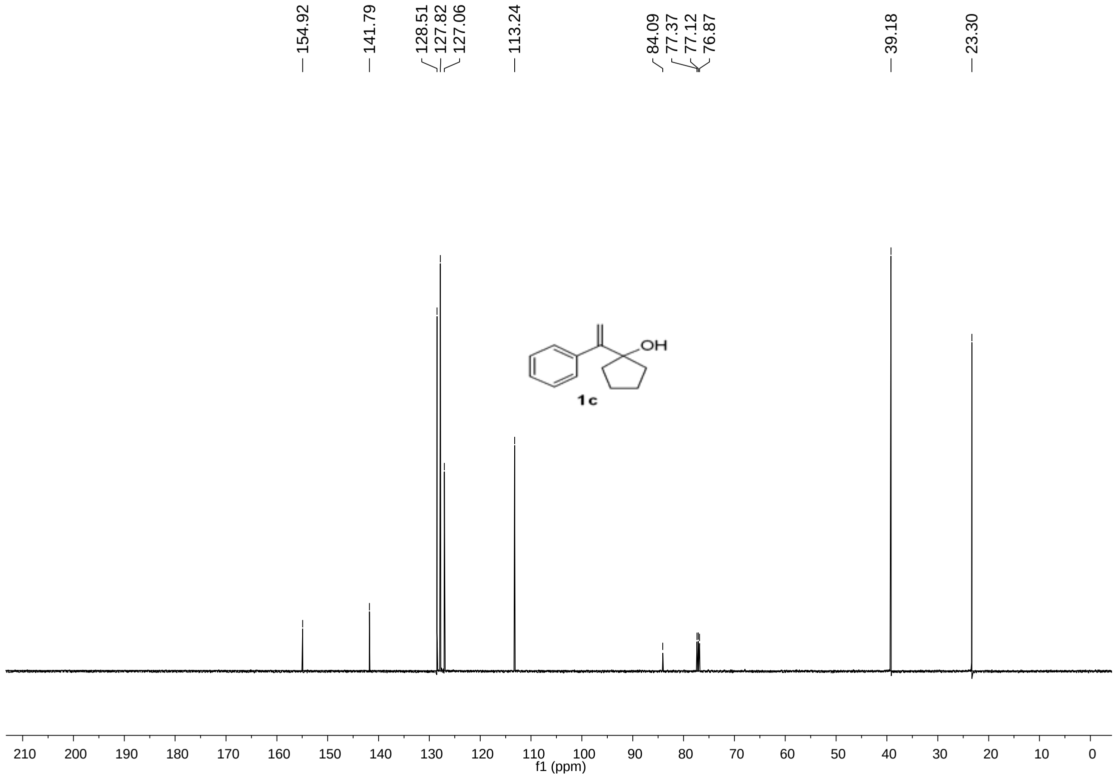
154.92
141.79
128.51
127.82
127.06
113.24
84.09
77.37
77.12
76.87
39.18
23.30
OH
1c
210 200 190 180 170 160 150 140 130 120 110 100 90 80 70 60 50 40 30 20 10 0
f1 (ppm)

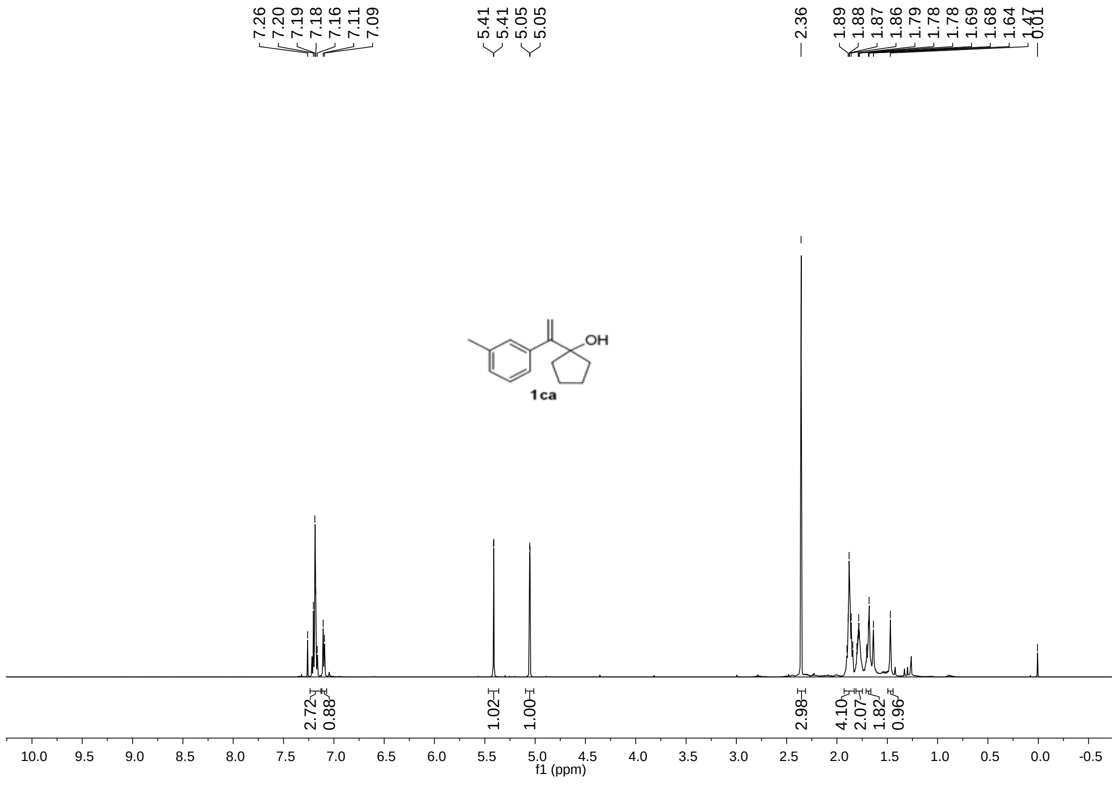
7.26
7.20
7.19
7.18
7.16
7.11
7.09
5.41
5.41
5.05
5.05
2.36
1.89
1.88
1.87
1.86
1.79
1.78
1.78
1.69
1.68
1.64
1.47
0.01
OH
1ca
2.72
0.88
1.02
1.00
2.98
4.10
2.07
1.82
0.96
10.0 9.5 9.0 8.5 8.0 7.5 7.0 6.5 6.0 5.5 5.0 4.5 4.0 3.5 3.0 2.5 2.0 1.5 1.0 0.5 0.0 -0.5
f1 (ppm)

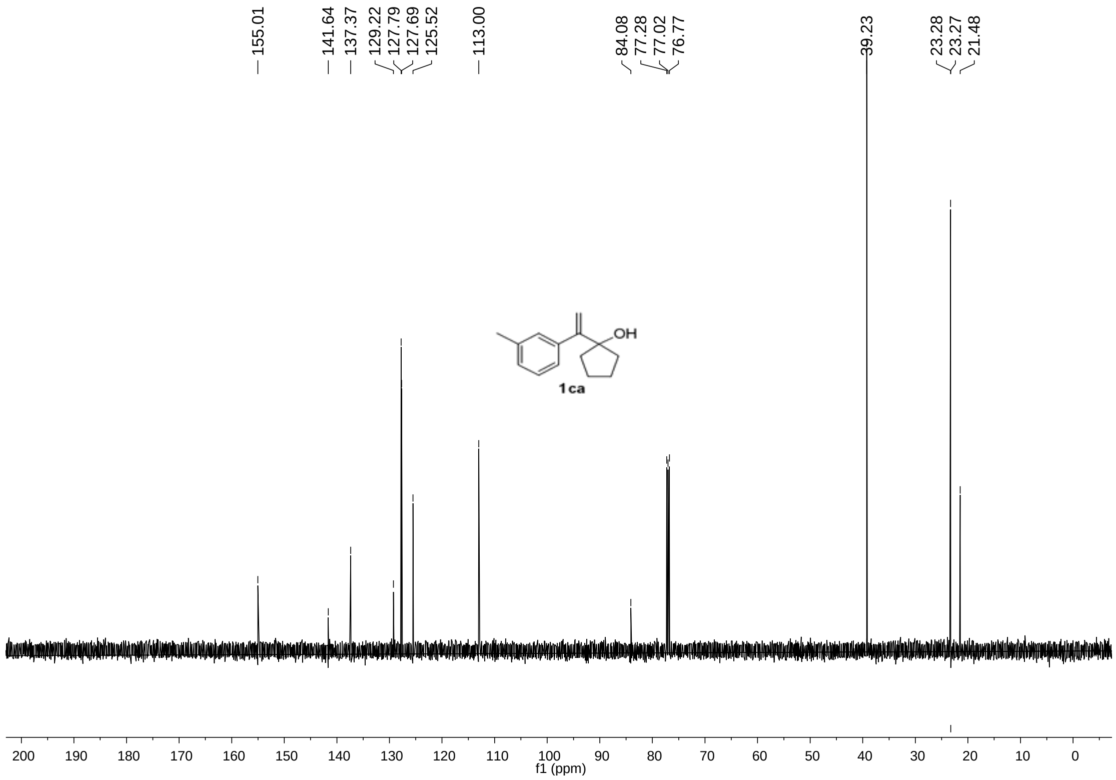

155.01
141.64
137.37
129.22
127.79
127.69
125.52
113.00
84.08
77.28
77.02
76.77
39.23
23.28
23.27
21.48
OH
1ca
200
190
180
170
160
150
140
130
120
110
100
90
80
70
60
50
40
30
20
10
0
f1 (ppm)

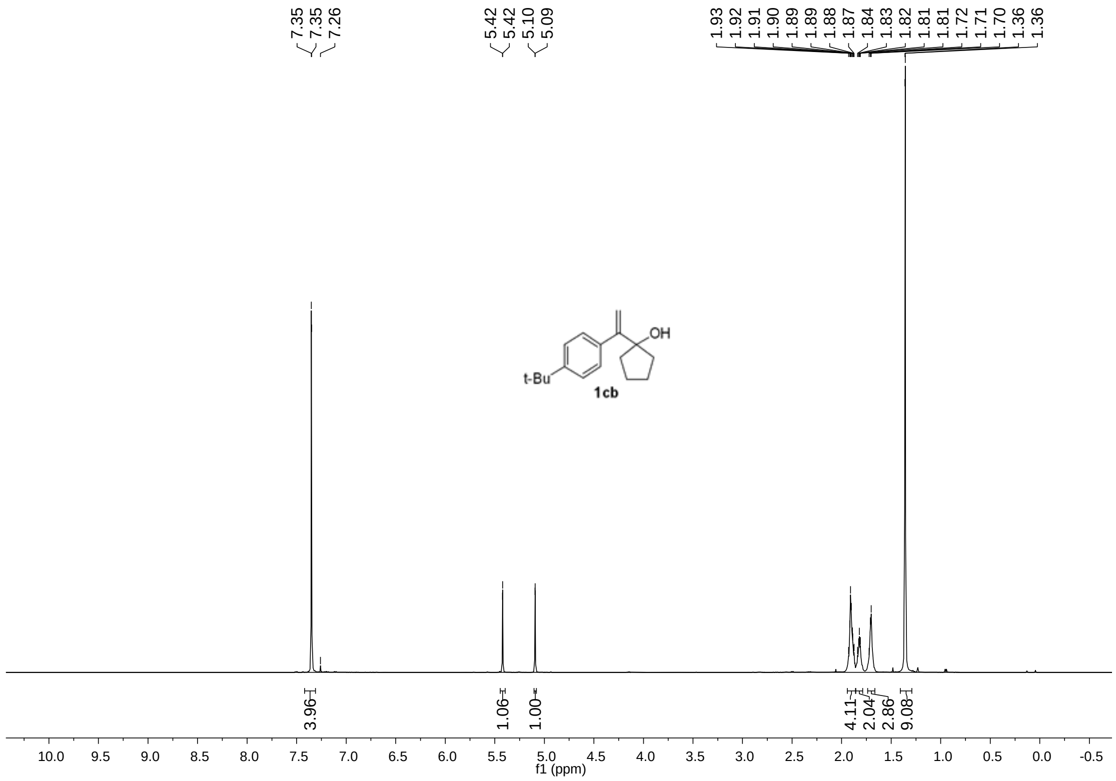

7.35
7.35
7.26
5.42
5.42
5.10
5.09
1.93
1.92
1.91
1.90
1.89
1.89
1.88
1.87
1.84
1.83
1.82
1.81
1.81
1.72
1.71
1.70
1.36
1.36
OH
t-Bu
1cb
3.96
1.06
1.00
4.11
2.04
2.86
9.08
10.0
9.5
9.0
8.5
8.0
7.5
7.0
6.5
6.0
5.5
5.0
4.5
4.0
3.5
3.0
2.5
2.0
1.5
1.0
0.5
0.0
-0.5
f1 (ppm)

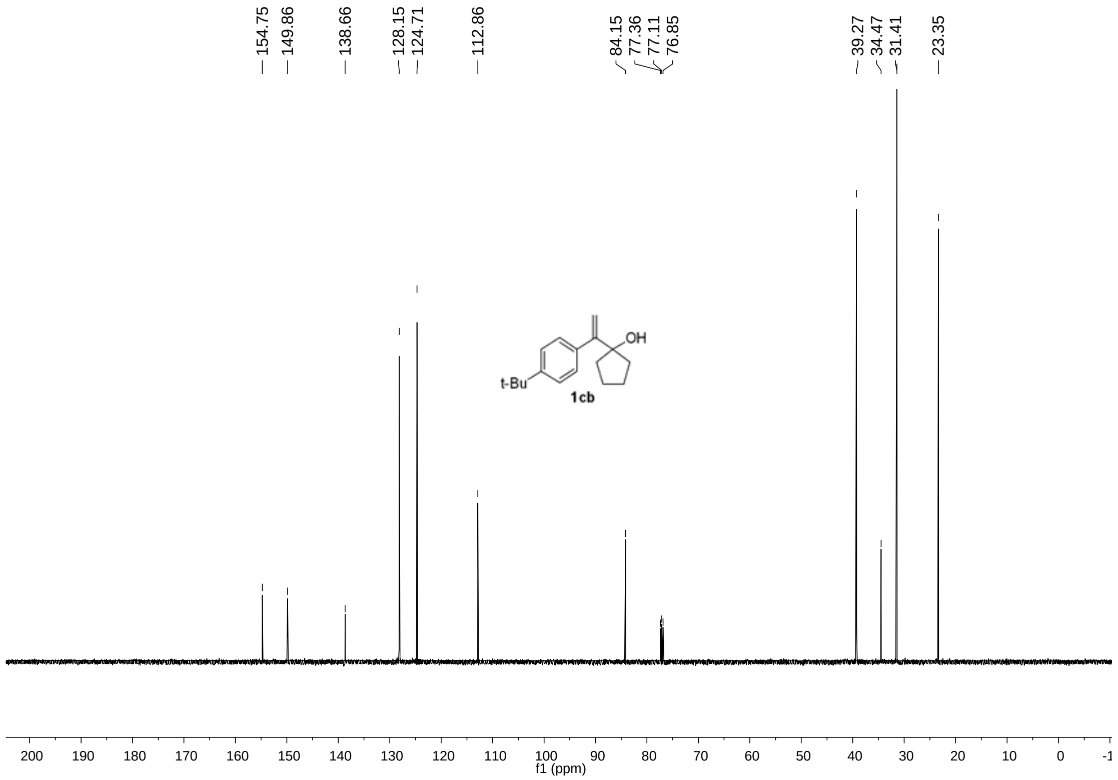
154.75
149.86
138.66
128.15
124.71
112.86
84.15
77.36
77.11
76.85
39.27
34.47
31.41
23.35
OH
t-Bu
1cb
200 190 180 170 160 150 140 130 120 110 100 90 80 70 60 50 40 30 20 10 0 -1
f1 (ppm)

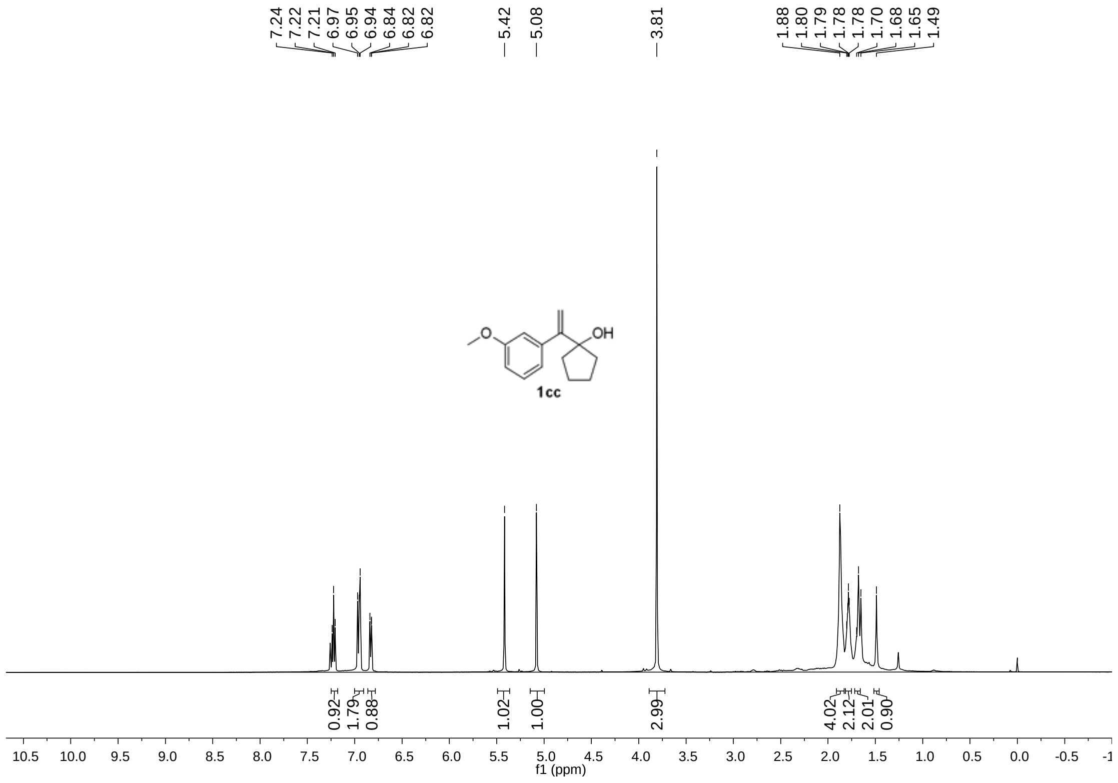
7.24
7.22
7.21
6.97
6.95
6.94
6.84
6.82
6.82
5.42
5.08
3.81
1.88
1.80
1.79
1.78
1.78
1.70
1.68
1.65
1.49
O
OH
1cc
0.92
1.79
0.88
1.02
1.00
2.99
4.02
2.12
2.01
0.90
10.5 10.0 9.5 9.0 8.5 8.0 7.5 7.0 6.5 6.0 5.5 5.0 4.5 4.0 3.5 3.0 2.5 2.0 1.5 1.0 0.5 0.0 -0.5 -1
f1 (ppm)

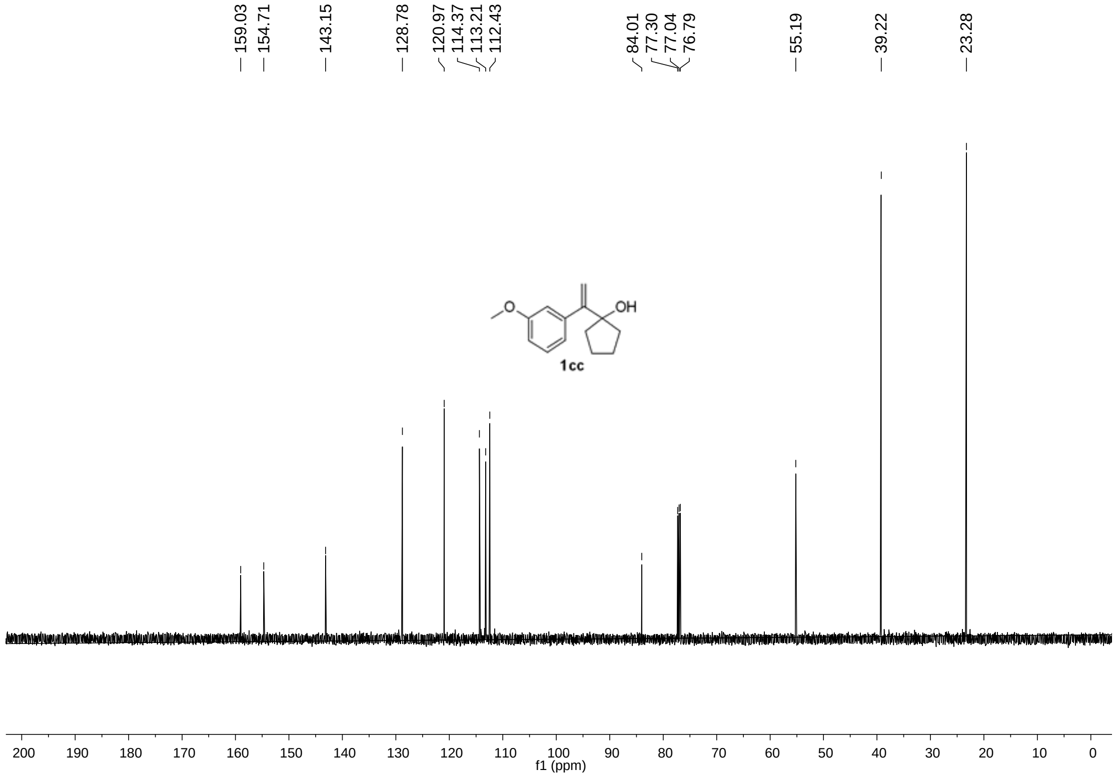
159.03
154.71
143.15
128.78
120.97
114.37
113.21
112.43
84.01
77.30
77.04
76.79
55.19
39.22
23.28
OH
1cc
f1 (ppm)

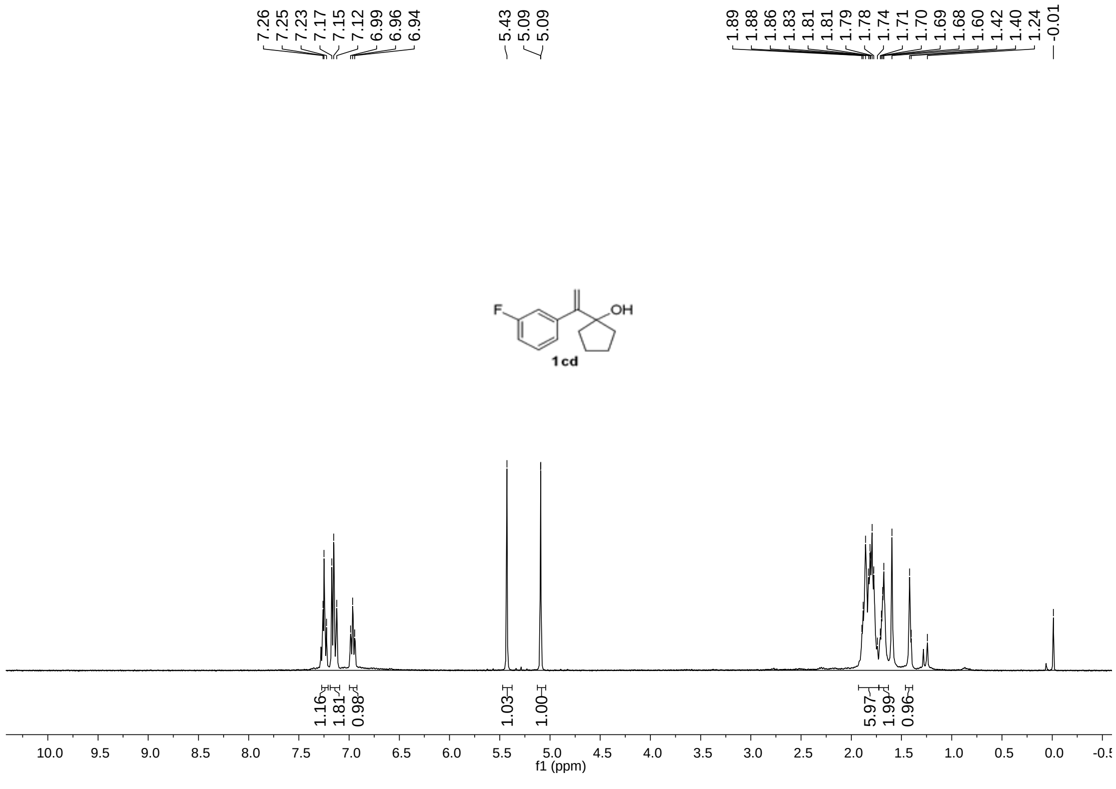
7.26
7.25
7.23
7.17
7.15
7.12
6.99
6.96
6.94
5.43
5.09
5.09
1.89
1.88
1.86
1.83
1.81
1.81
1.79
1.78
1.74
1.71
1.70
1.69
1.68
1.60
1.42
1.40
1.24
-0.01
F
OH
1cd
1.16
1.81
0.98
1.03
1.00
5.97
1.99
0.96
f1 (ppm)

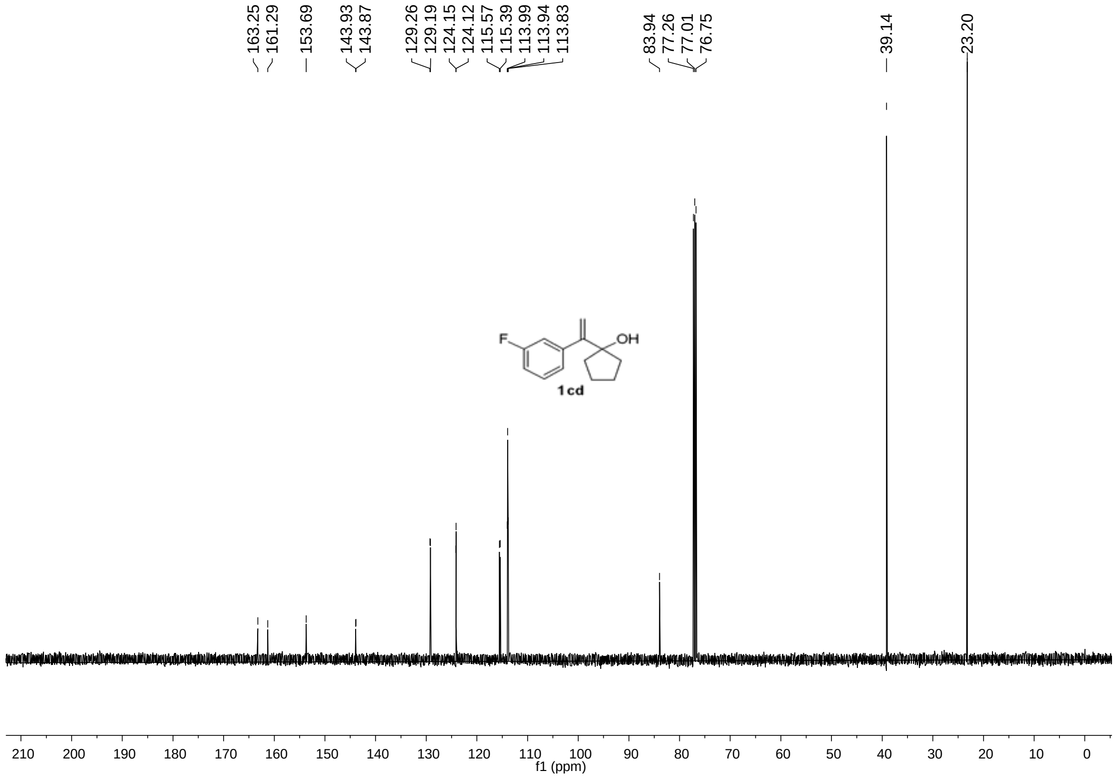
163.25
161.29
153.69
143.93
143.87
129.26
129.19
124.15
124.12
115.57
115.39
113.99
113.94
113.83
83.94
77.26
77.01
76.75
39.14
23.20
F
OH
1cd
210 200 190 180 170 160 150 140 130 120 110 100 90 80 70 60 50 40 30 20 10 0
f1 (ppm)

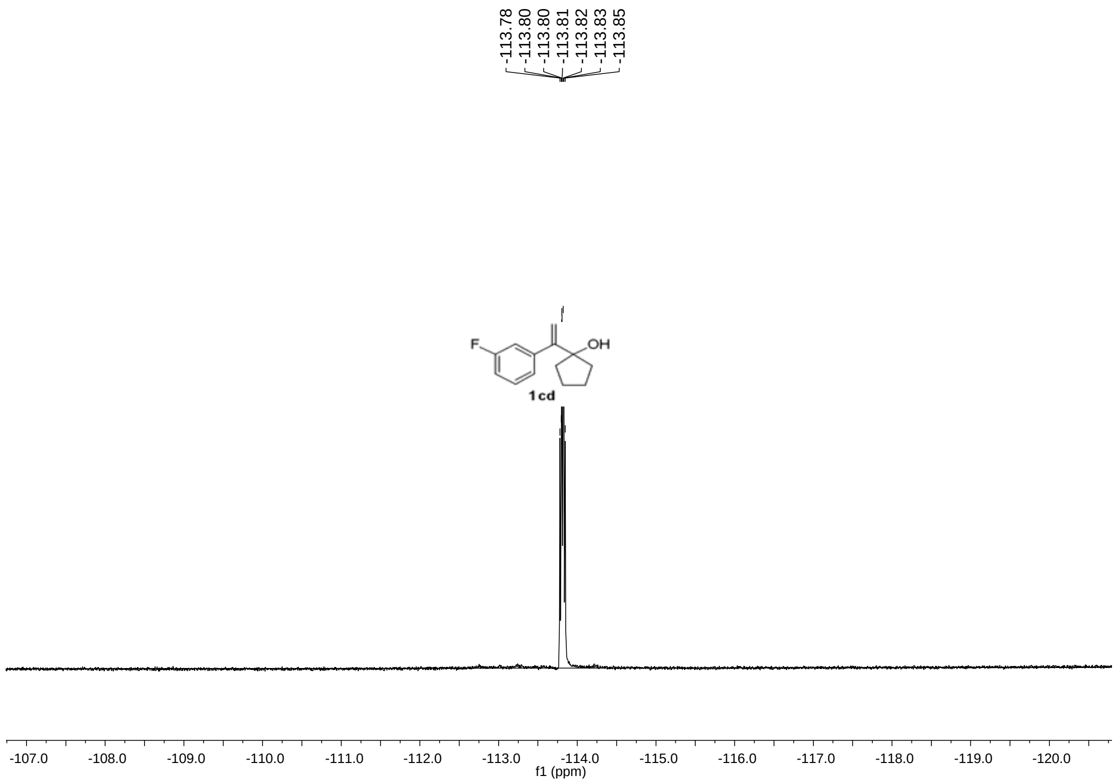
-113.78
-113.80
-113.80
-113.81
-113.82
-113.83
-113.85
F
OH
1cd
-107.0 -108.0 -109.0 -110.0 -111.0 -112.0 -113.0 -114.0 -115.0 -116.0 -117.0 -118.0 -119.0 -120.0
f1 (ppm)

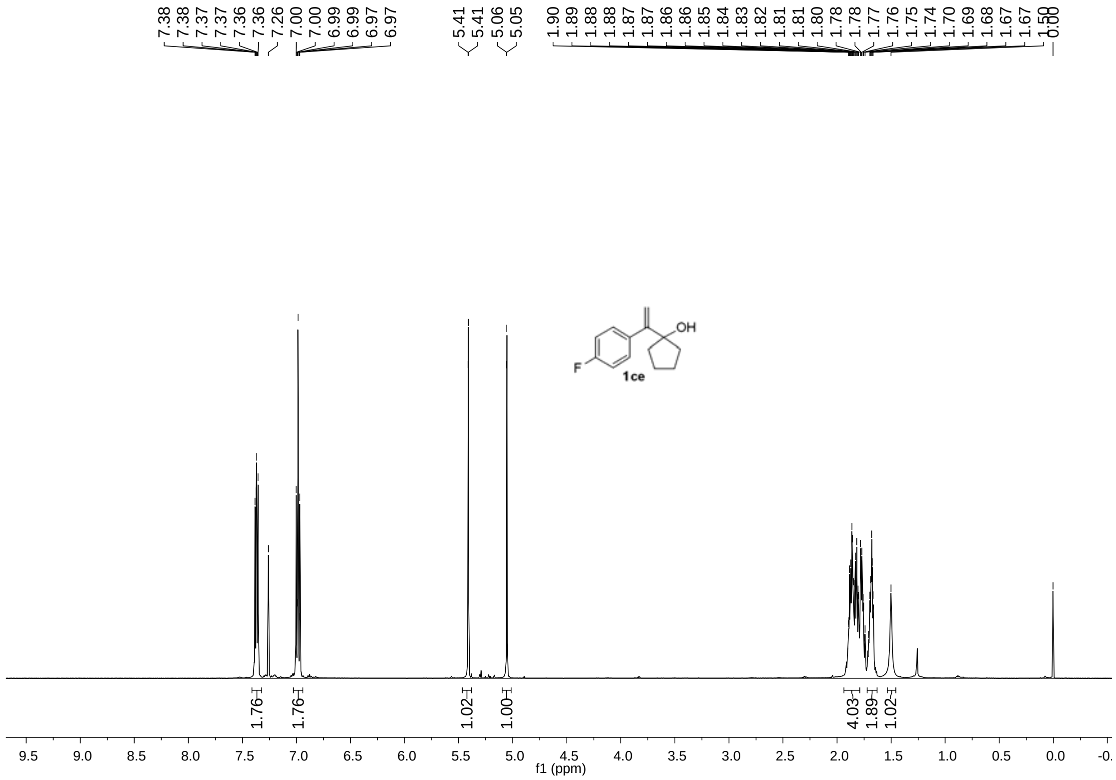
7.38 7.38 7.37 7.36 7.36 7.26 7.00 7.00 6.99 6.99 6.97 6.97
5.41 5.41 5.06 5.05
1.90 1.89 1.88 1.87 1.87 1.86 1.86 1.85 1.84 1.83 1.82 1.81 1.81 1.80 1.78 1.78 1.77 1.76 1.75 1.74 1.70 1.69 1.68 1.67 1.67 1.50 0.00
OH
F
1ce
1.76 1.76 1.02 1.00 4.03 1.89 1.02
9.5 9.0 8.5 8.0 7.5 7.0 6.5 6.0 5.5 5.0 4.5 4.0 3.5 3.0 2.5 2.0 1.5 1.0 0.5 0.0
f1 (ppm)

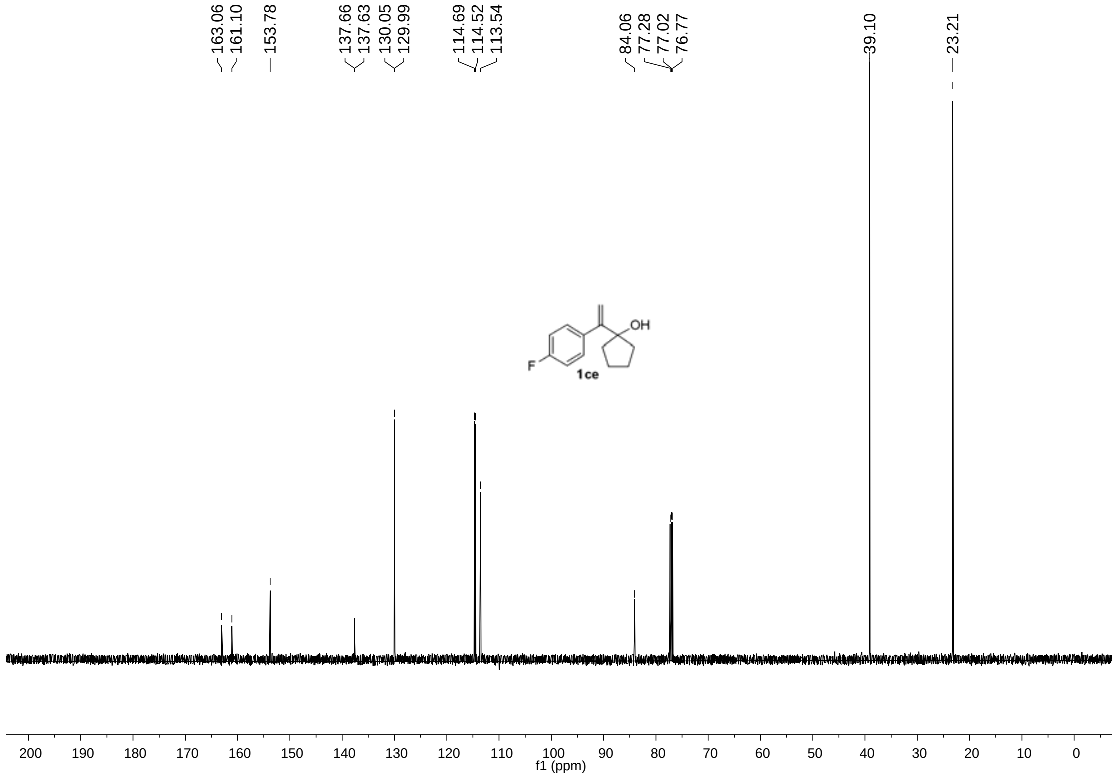
163.06 161.10 153.78
137.66 137.63 130.05 129.99
114.69 114.52 113.54
84.06 77.28 77.02 76.77
39.10 23.21
OH
F
1ce
200 190 180 170 160 150 140 130 120 110 100 90 80 70 60 50 40 30 20 10 0
f1 (ppm)

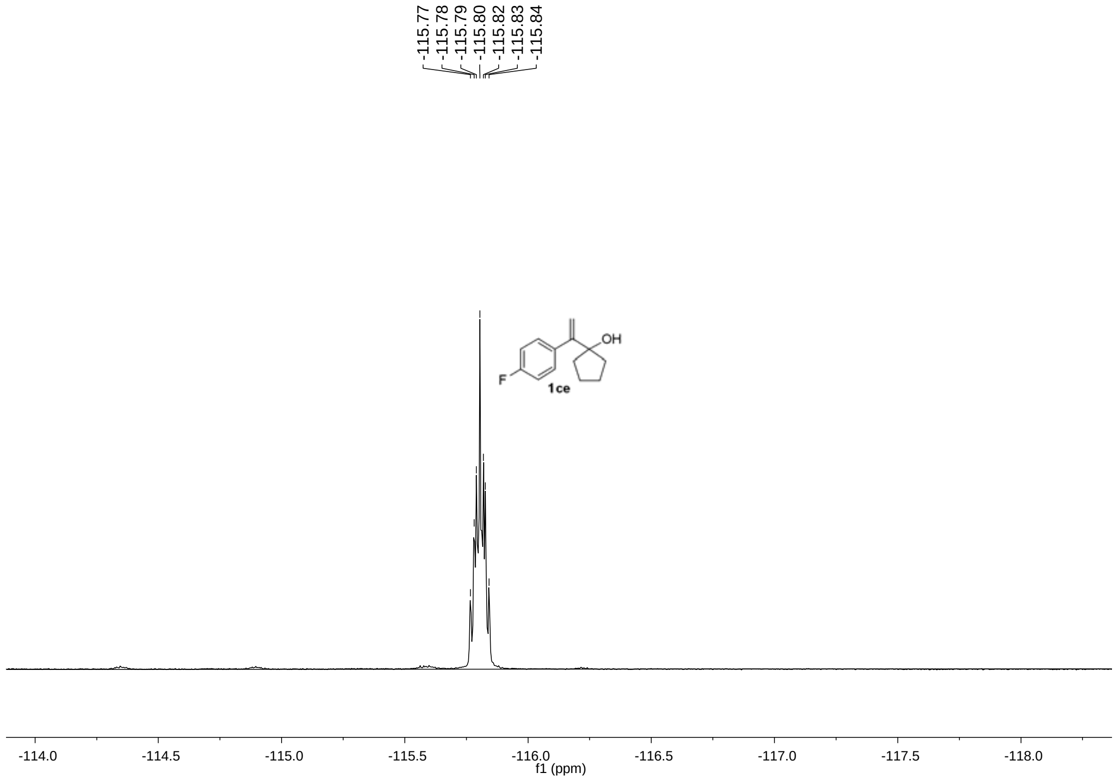
-115.77
-115.78
-115.79
-115.80
-115.82
-115.83
-115.84
F
OH
1ce
-114.0
-114.5
-115.0
-115.5
-116.0
f1 (ppm)
-116.5
-117.0
-117.5
-118.0

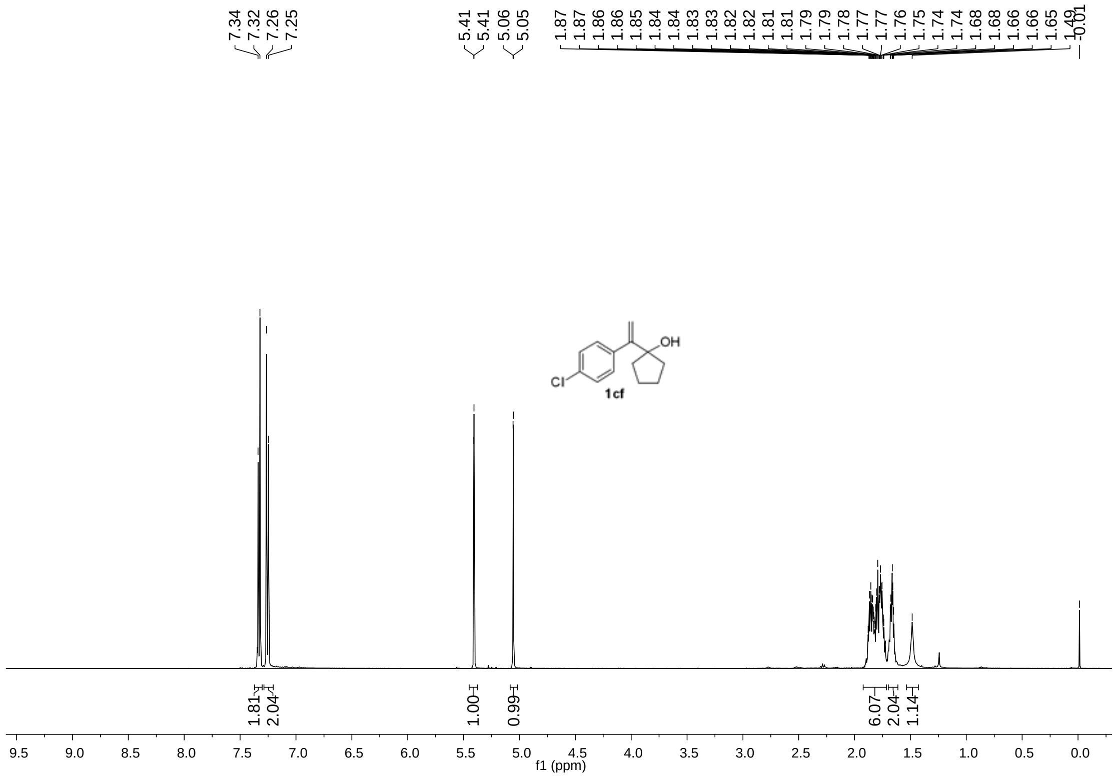
7.34
7.32
7.26
7.25
5.41
5.41
5.06
5.05
1.87
1.87
1.86
1.86
1.85
1.84
1.84
1.83
1.83
1.82
1.82
1.81
1.81
1.79
1.78
1.77
1.76
1.75
1.74
1.74
1.68
1.68
1.66
1.66
1.65
1.49
-0.01
Cl
OH
1cf
1.81
2.04
1.00
0.99
6.07
2.04
1.14
9.5
9.0
8.5
8.0
7.5
7.0
6.5
6.0
5.5
5.0
f1 (ppm)
4.5
4.0
3.5
3.0
2.5
2.0
1.5
1.0
0.5
0.0

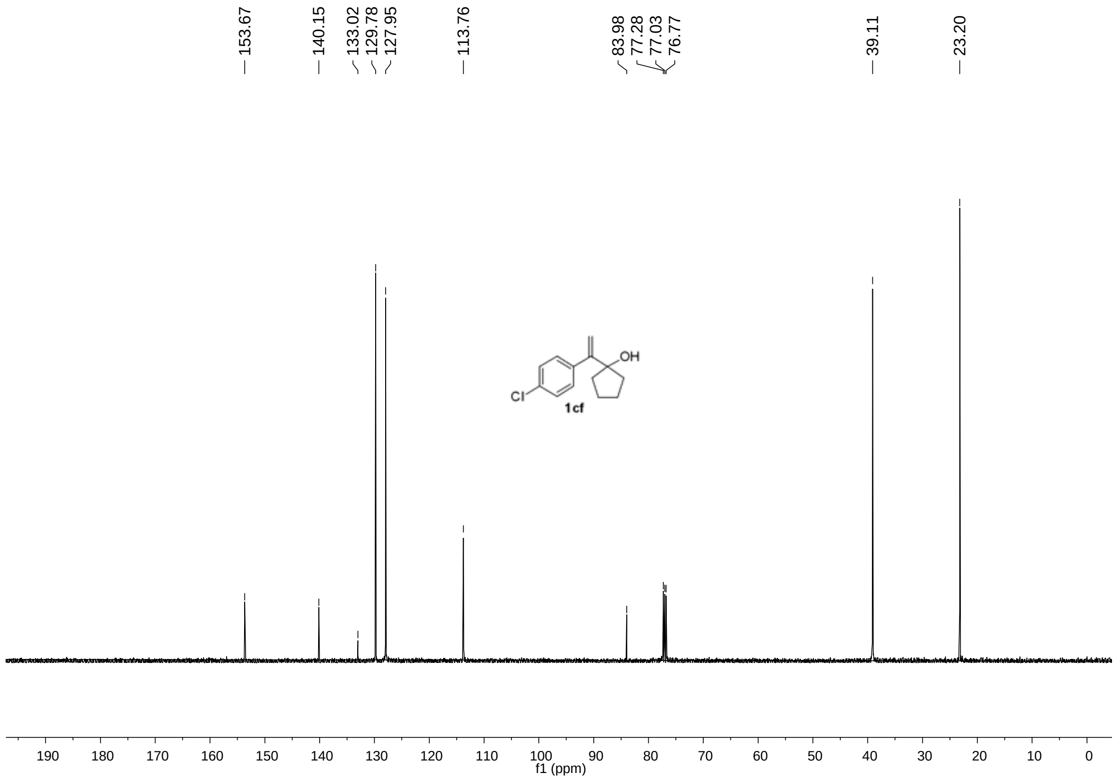
153.67
140.15
133.02
129.78
127.95
113.76
83.98
77.28
77.03
76.77
39.11
23.20
Cl
OH
1cf
190 180 170 160 150 140 130 120 110 100 90 80 70 60 50 40 30 20 10 0
f1 (ppm)

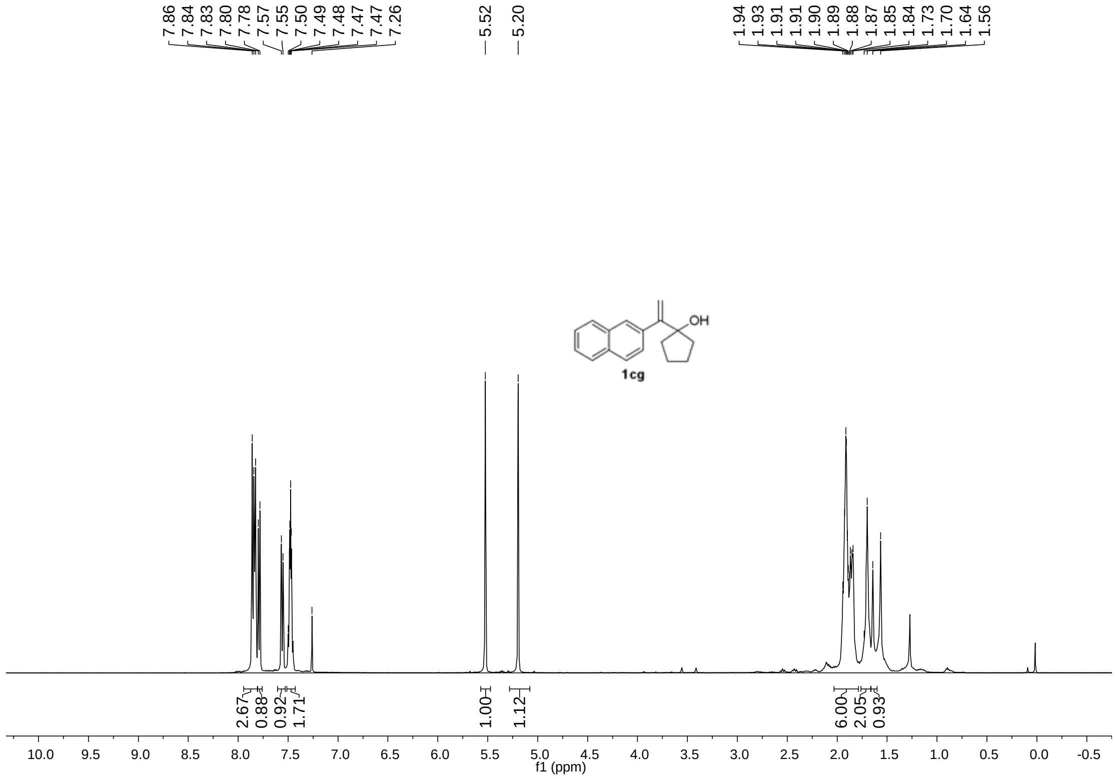
7.86
7.84
7.83
7.80
7.78
7.57
7.55
7.50
7.49
7.48
7.47
7.47
7.26
5.52
5.20
1.94
1.93
1.91
1.91
1.90
1.89
1.88
1.87
1.85
1.84
1.73
1.70
1.64
1.56
OH
1cg
2.67
0.88
0.92
1.71
1.00
1.12
6.00
2.05
0.93
10.0 9.5 9.0 8.5 8.0 7.5 7.0 6.5 6.0 5.5 5.0 4.5 4.0 3.5 3.0 2.5 2.0 1.5 1.0 0.5 0.0 -0.5
f1 (ppm)

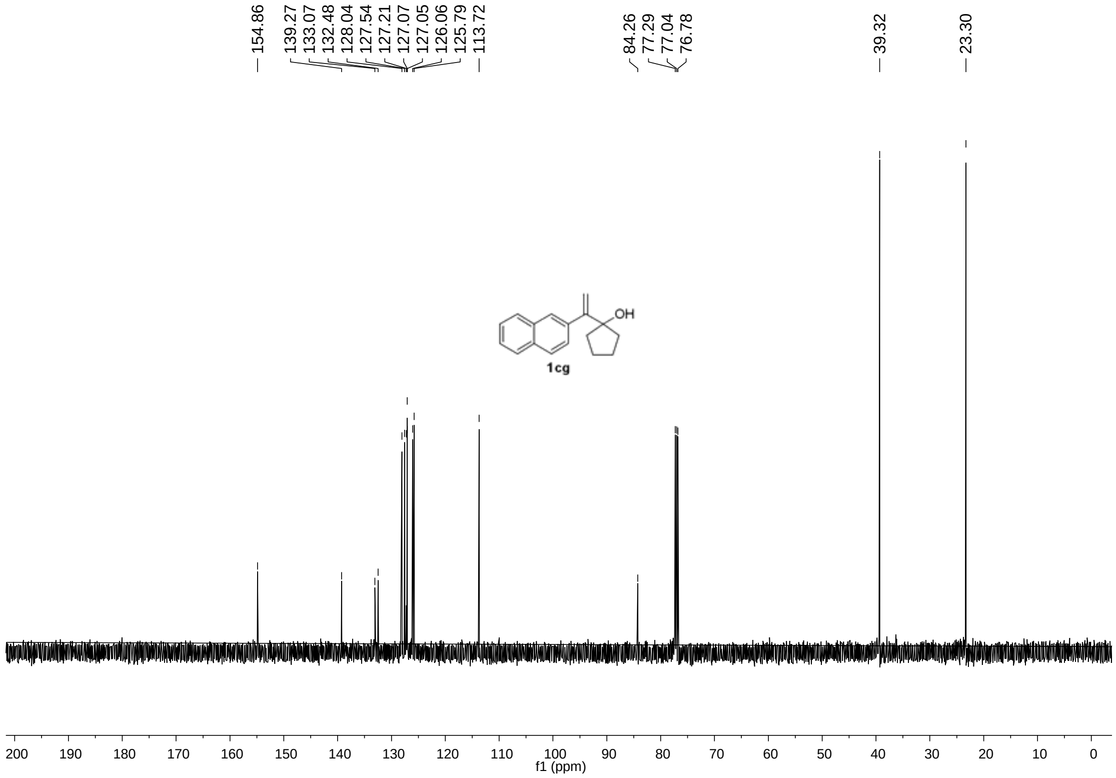
154.86
139.27
133.07
132.48
128.04
127.54
127.21
127.07
127.05
126.06
125.79
113.72
84.26
77.29
77.04
76.78
39.32
23.30
OH
1cg
f1 (ppm)

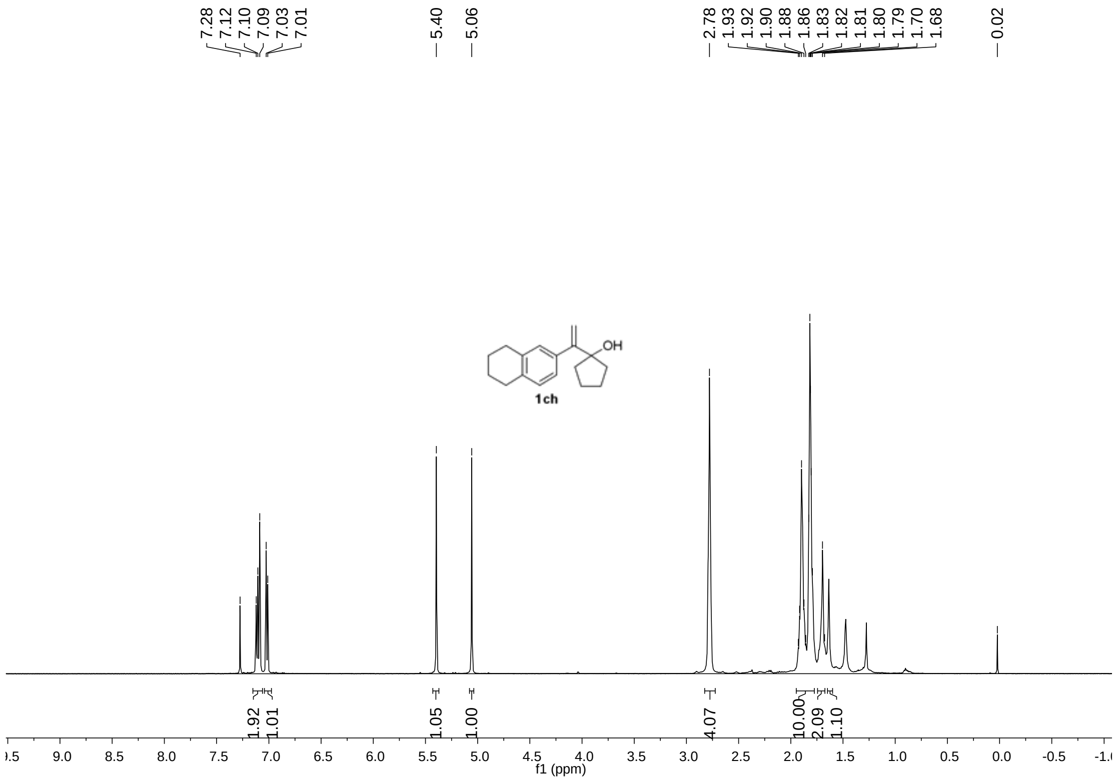
7.28
7.12
7.10
7.09
7.03
7.01
5.40
5.06
2.78
1.93
1.92
1.90
1.88
1.86
1.83
1.82
1.81
1.80
1.79
1.70
1.68
0.02
OH
1ch
1.92
1.01
1.05
1.00
4.07
10.00
2.09
1.10
f1 (ppm)

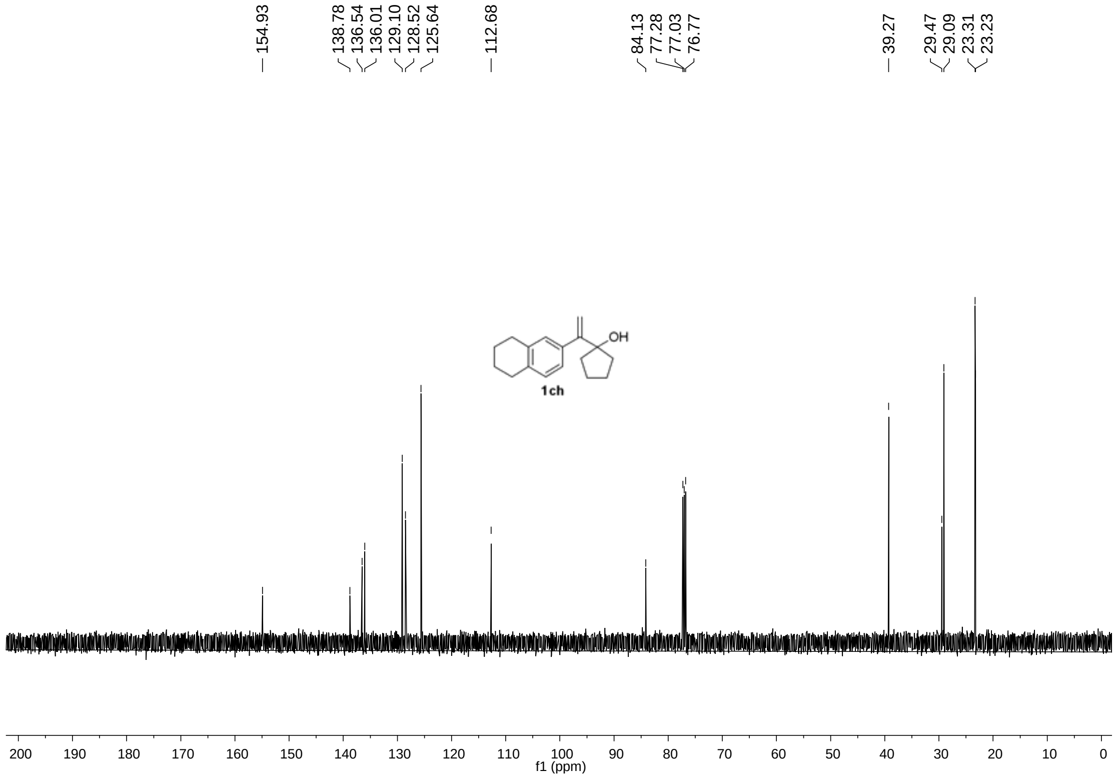
154.93
138.78
136.54
136.01
129.10
128.52
125.64
112.68
84.13
77.28
77.03
76.77
39.27
29.47
29.09
23.31
23.23
OH
1ch
200 190 180 170 160 150 140 130 120 110 100 90 80 70 60 50 40 30 20 10 0
f1 (ppm)

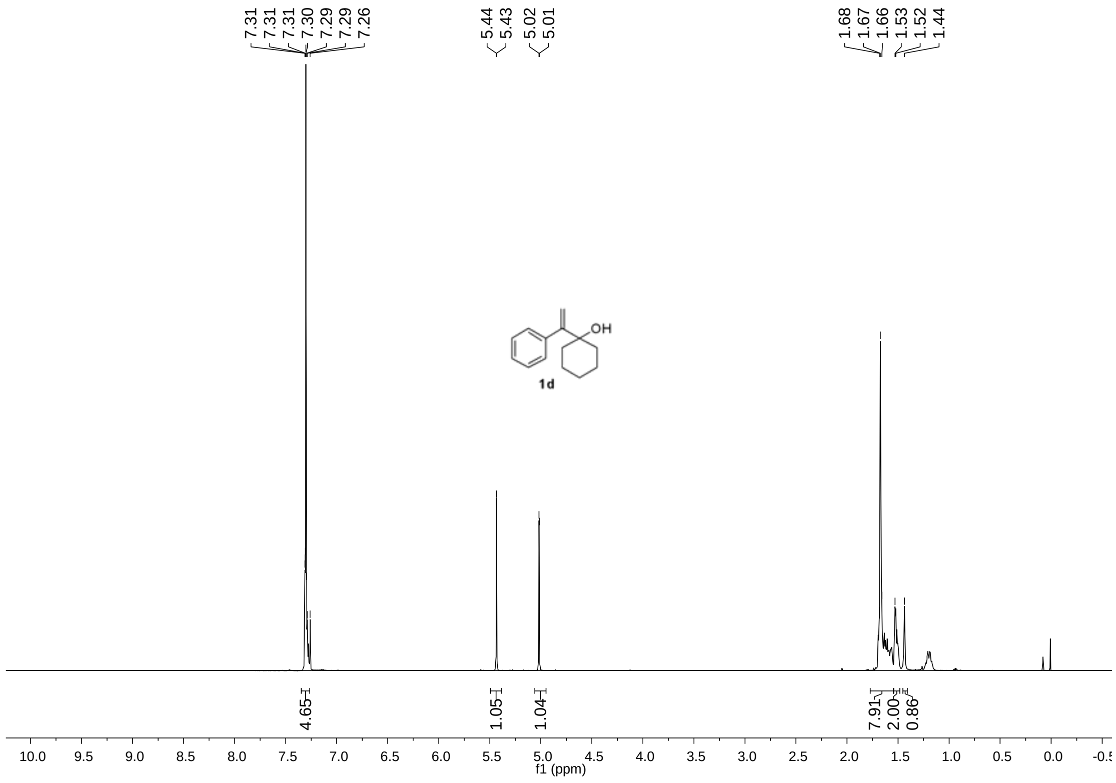
7.31
7.31
7.31
7.30
7.29
7.29
7.26
5.44
5.43
5.02
5.01
1.68
1.67
1.66
1.53
1.52
1.44
OH
1d
4.65
1.05
1.04
7.91
2.00
0.86
10.0 9.5 9.0 8.5 8.0 7.5 7.0 6.5 6.0 5.5 5.0 4.5 4.0 3.5 3.0 2.5 2.0 1.5 1.0 0.5 0.0
f1 (ppm)

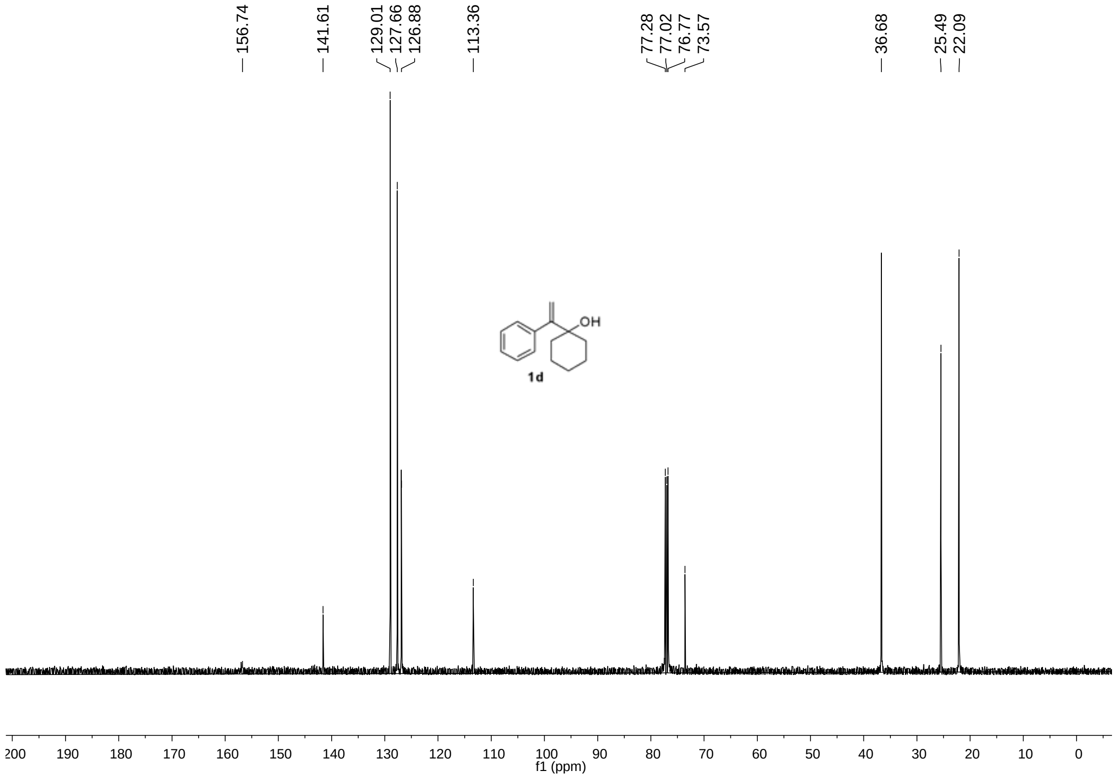
156.74
141.61
129.01
127.66
126.88
113.36
77.28
77.02
76.77
73.57
36.68
25.49
22.09
OH
1d
200 190 180 170 160 150 140 130 120 110 100 90 80 70 60 50 40 30 20 10 0
f1 (ppm)

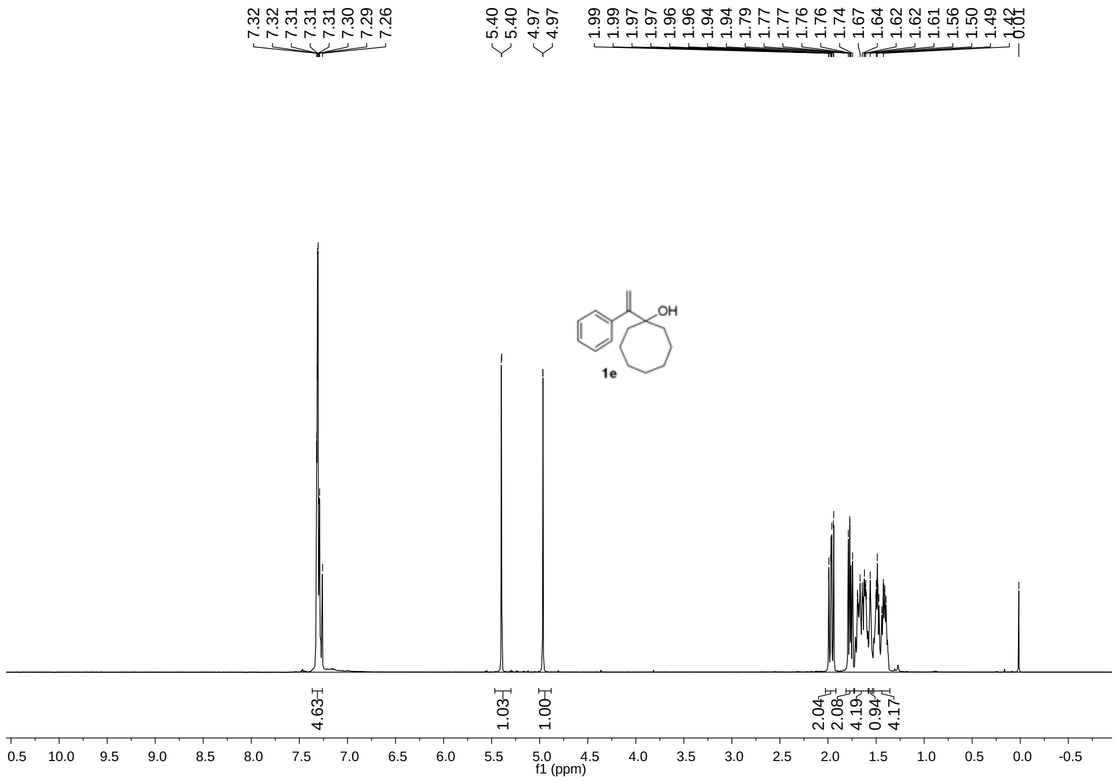
7.32
7.32
7.31
7.31
7.31
7.30
7.29
7.26
5.40
5.40
4.97
4.97
1.99
1.99
1.97
1.97
1.96
1.96
1.94
1.94
1.79
1.77
1.77
1.76
1.74
1.67
1.64
1.62
1.61
1.56
1.50
1.49
1.42
0.01
OH
1e
4.63
1.03
1.00
2.04
2.08
4.19
0.94
4.17
0.5 10.0 9.5 9.0 8.5 8.0 7.5 7.0 6.5 6.0 5.5 5.0 4.5 4.0 3.5 3.0 2.5 2.0 1.5 1.0 0.5 0.0 -0.5
f1 (ppm)

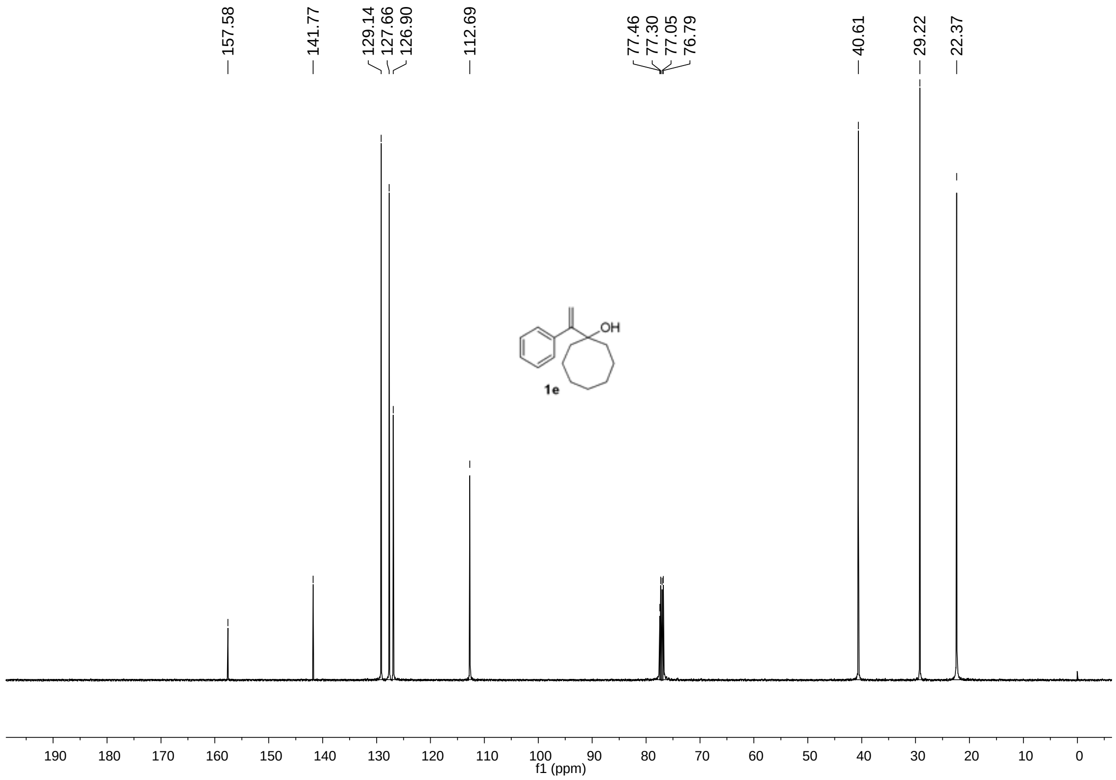
157.58
141.77
129.14
127.66
126.90
112.69
77.46
77.30
77.05
76.79
40.61
29.22
22.37
OH
1e
190 180 170 160 150 140 130 120 110 100 90 80 70 60 50 40 30 20 10 0
f1 (ppm)

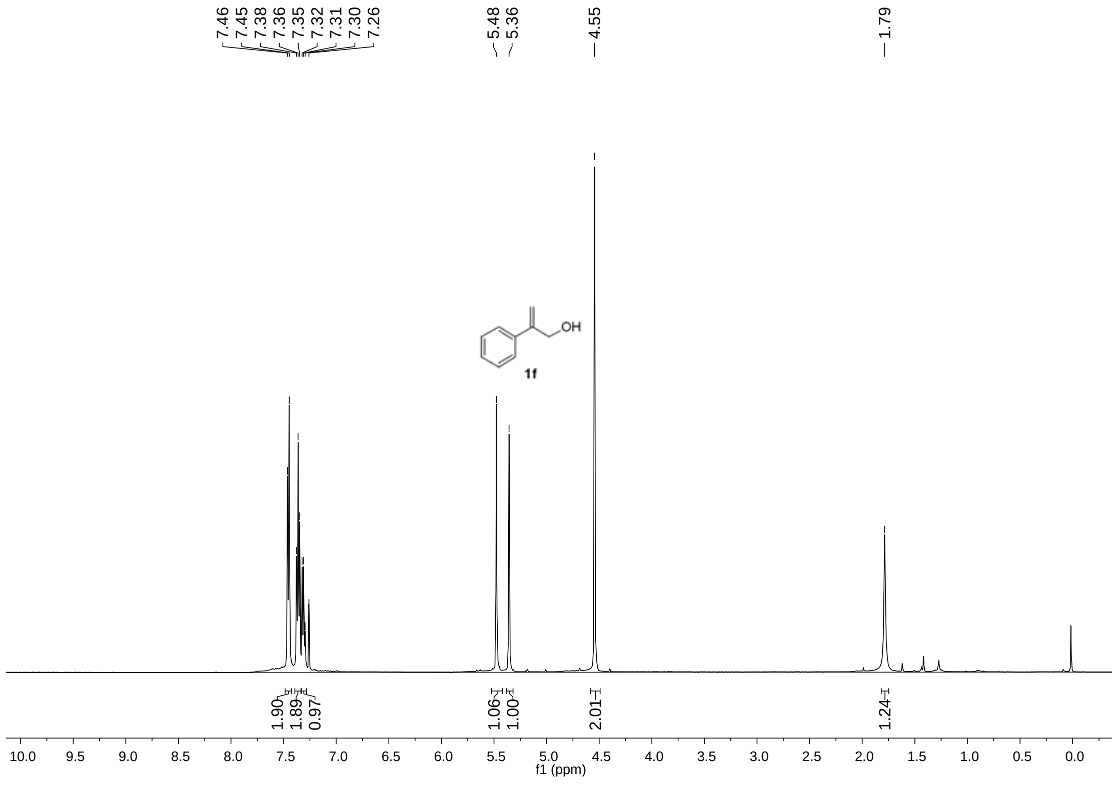
7.46
7.45
7.38
7.36
7.35
7.32
7.31
7.30
7.26
5.48
5.36
4.55
1.79
OH
1f
1.90
1.89
0.97
1.06
1.00
2.01
1.24
10.0 9.5 9.0 8.5 8.0 7.5 7.0 6.5 6.0 5.5 5.0 4.5 4.0 3.5 3.0 2.5 2.0 1.5 1.0 0.5 0.0
f1 (ppm)

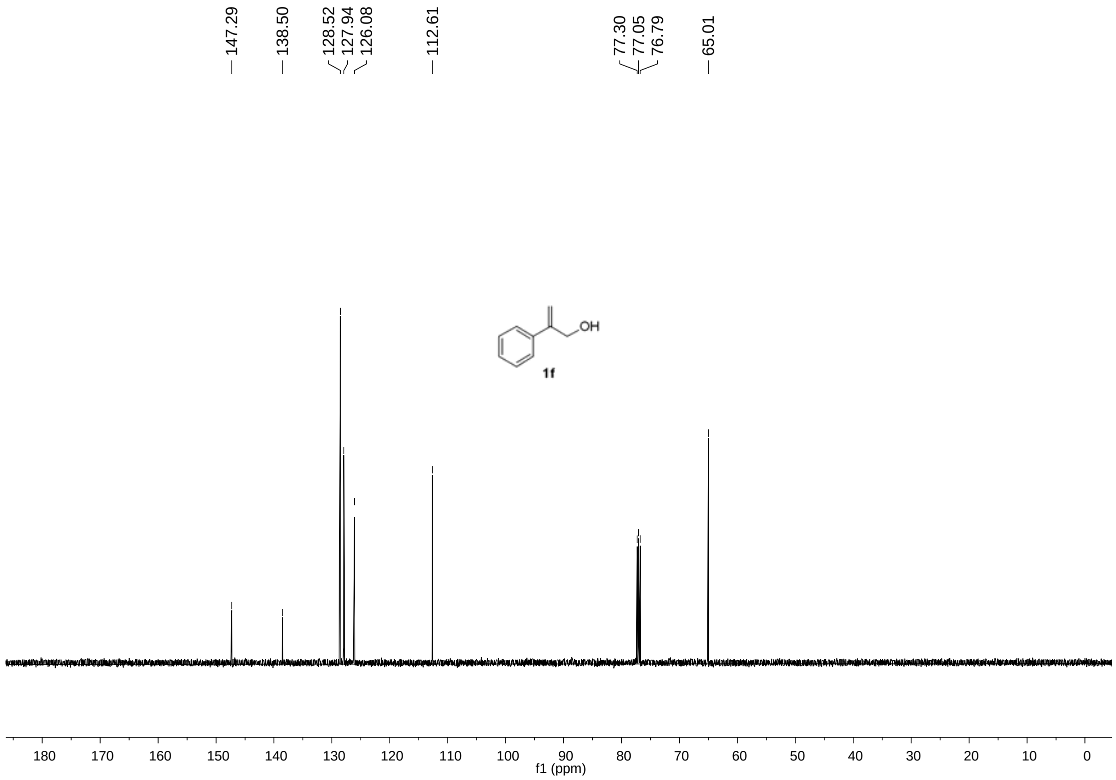

147.29
138.50
128.52
127.94
126.08
112.61
77.30
77.05
76.79
65.01
OH
1f
180 170 160 150 140 130 120 110 100 90 80 70 60 50 40 30 20 10 0
f1 (ppm)

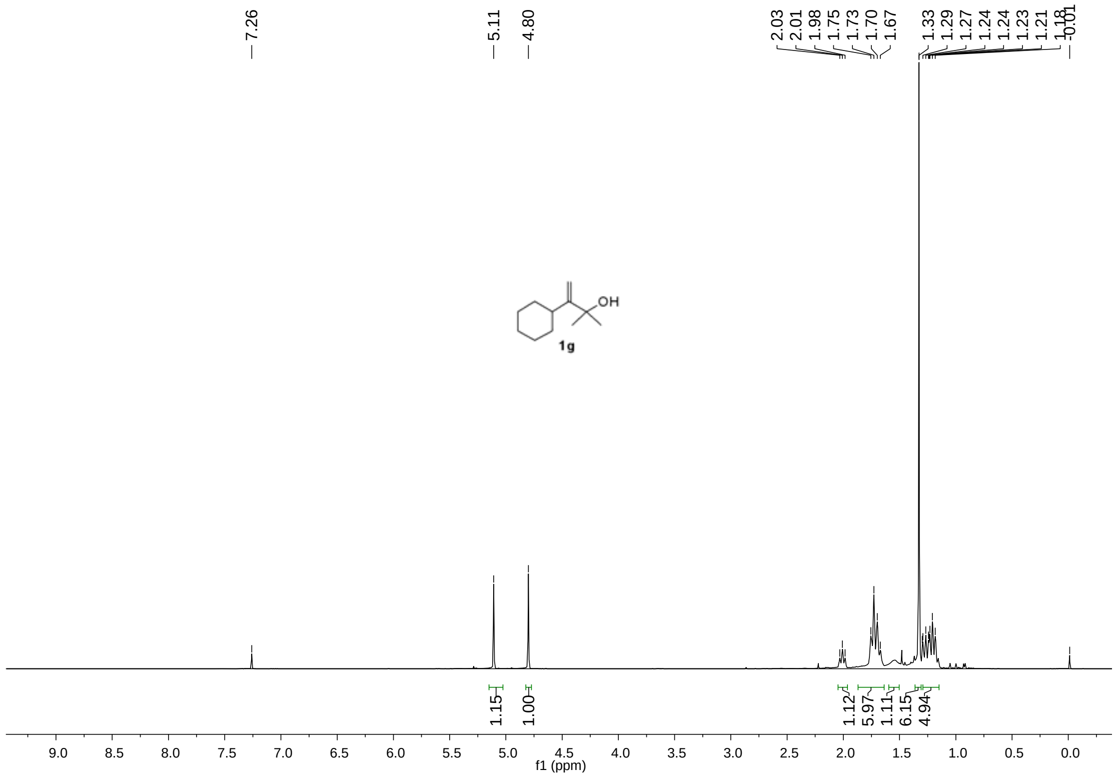

7.26
5.11
4.80
2.03 2.01 1.98 1.75 1.73 1.70 1.67
1.33 1.29 1.27 1.24 1.24 1.23 1.21 1.18
-0.01
OH
1g
1.15 1.00 1.12 5.97 1.11 6.15 4.94
9.0 8.5 8.0 7.5 7.0 6.5 6.0 5.5 5.0 4.5 4.0 3.5 3.0 2.5 2.0 1.5 1.0 0.5 0.0
f1 (ppm)

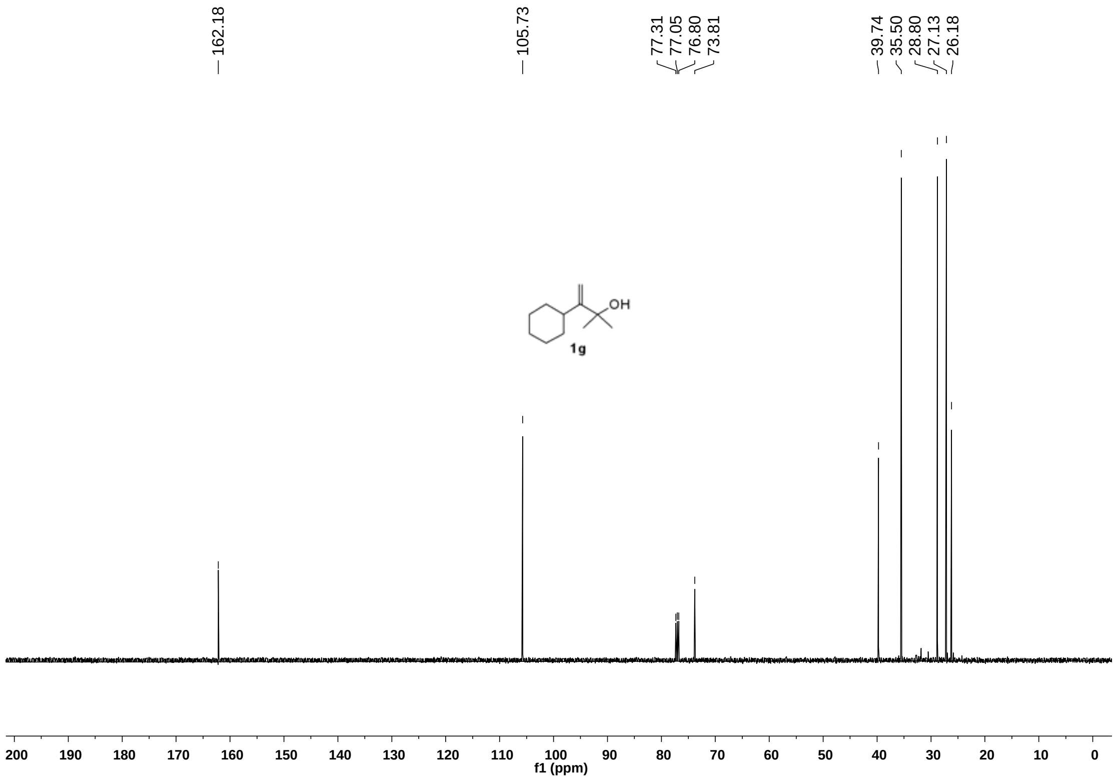
162.18
105.73
77.31
77.05
76.80
73.81
39.74
35.50
28.80
27.13
26.18
OH
1g
200 190 180 170 160 150 140 130 120 110 100 90 80 70 60 50 40 30 20 10 0
f1 (ppm)

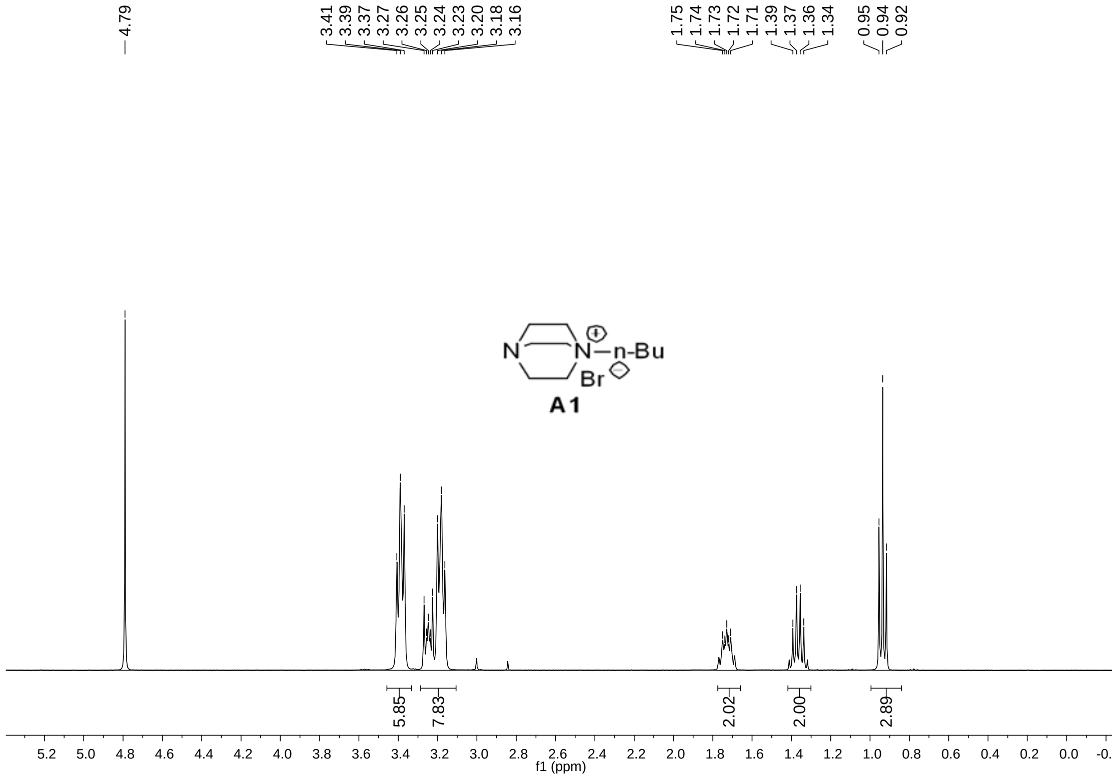
4.79
3.41
3.39
3.37
3.27
3.26
3.25
3.24
3.23
3.20
3.18
3.16
1.75
1.74
1.73
1.72
1.71
1.39
1.37
1.36
1.34
0.95
0.94
0.92
N
N
n-Bu
Br
A1
5.85
7.83
2.02
2.00
2.89
5.2 5.0 4.8 4.6 4.4 4.2 4.0 3.8 3.6 3.4 3.2 3.0 2.8 2.6 2.4 2.2 2.0 1.8 1.6 1.4 1.2 1.0 0.8 0.6 0.4 0.2 0.0 -0.
f1 (ppm)

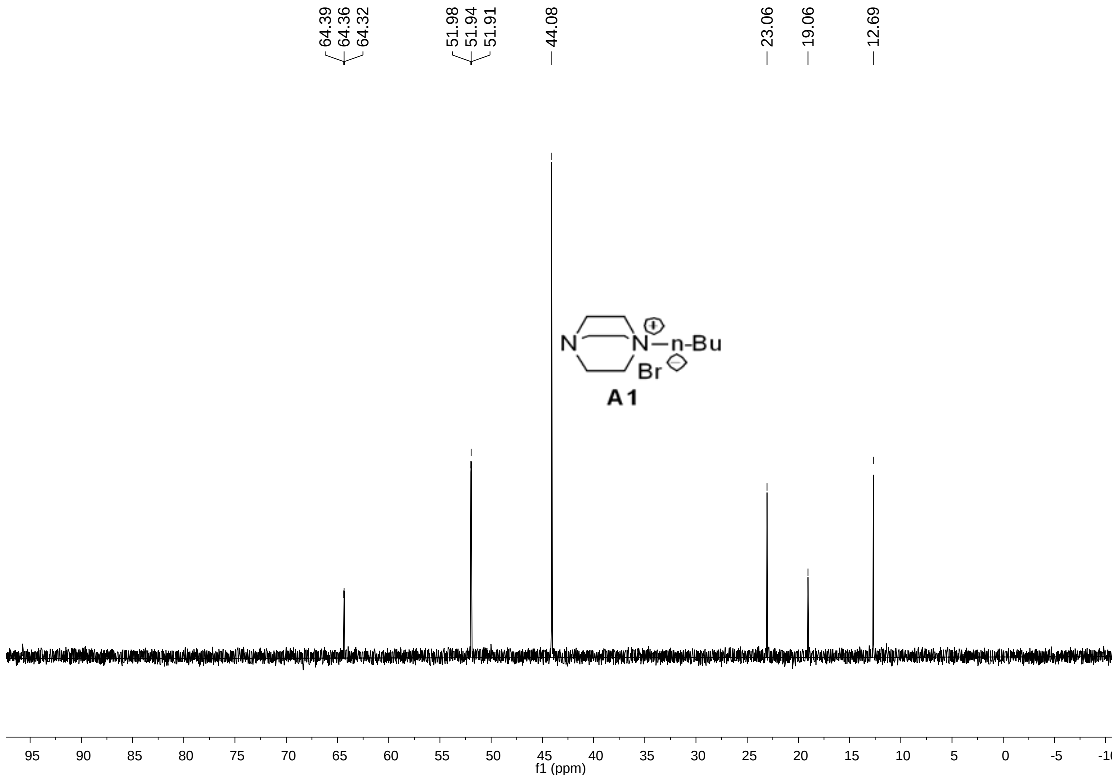
N
N
n-Bu
Br
A1
f1 (ppm)

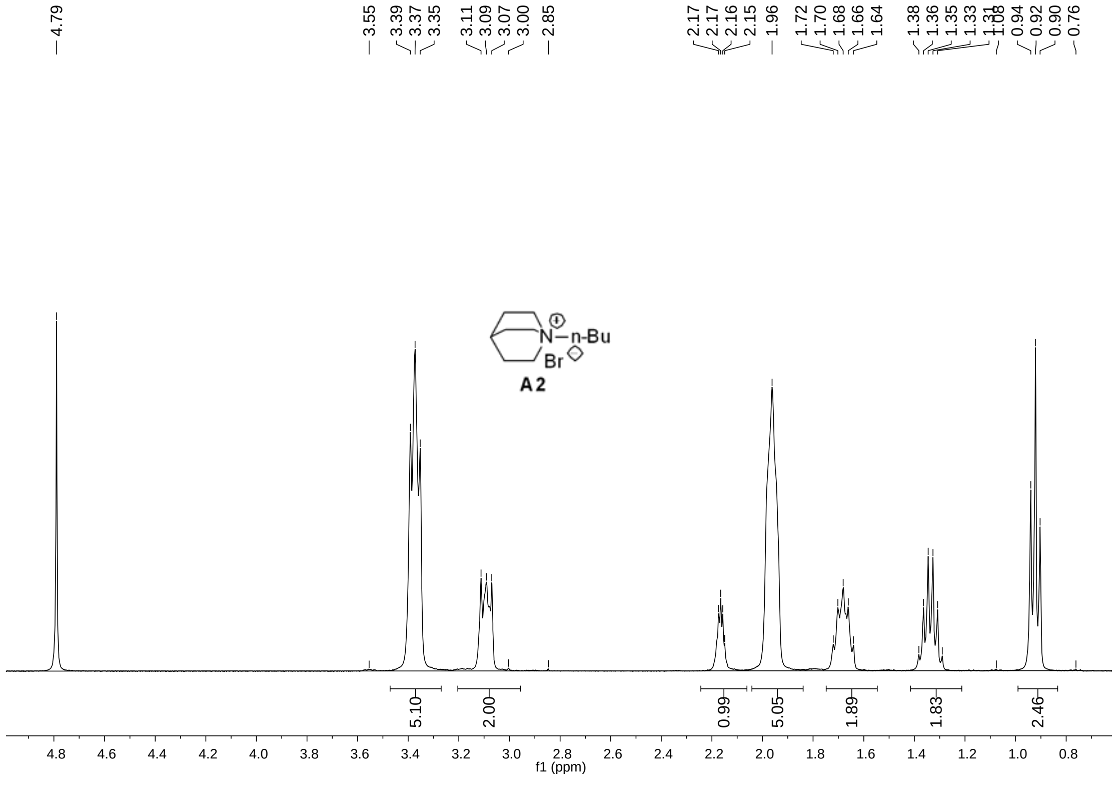
N
n-Bu
Br
A2
f1 (ppm)

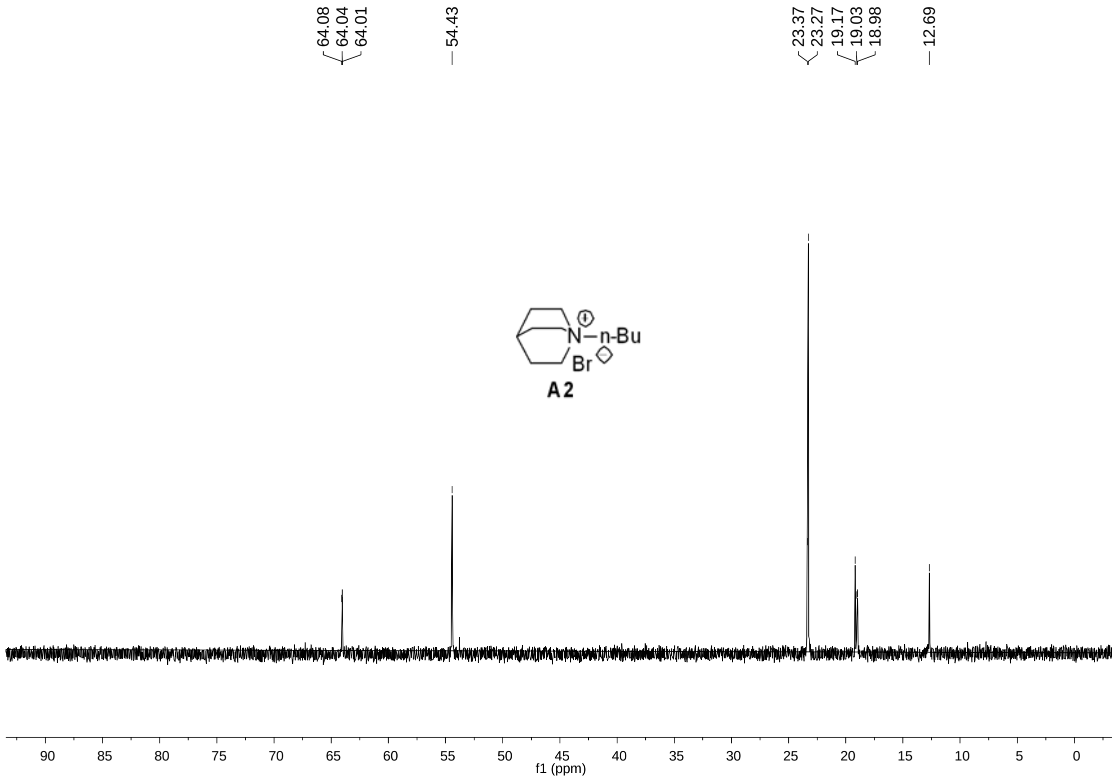
64.08
64.04
64.01
54.43
23.37
23.27
19.17
19.03
18.98
12.69
N−n-Bu
Br
A2
90 85 80 75 70 65 60 55 50 45 40 35 30 25 20 15 10 5 0
f1 (ppm)

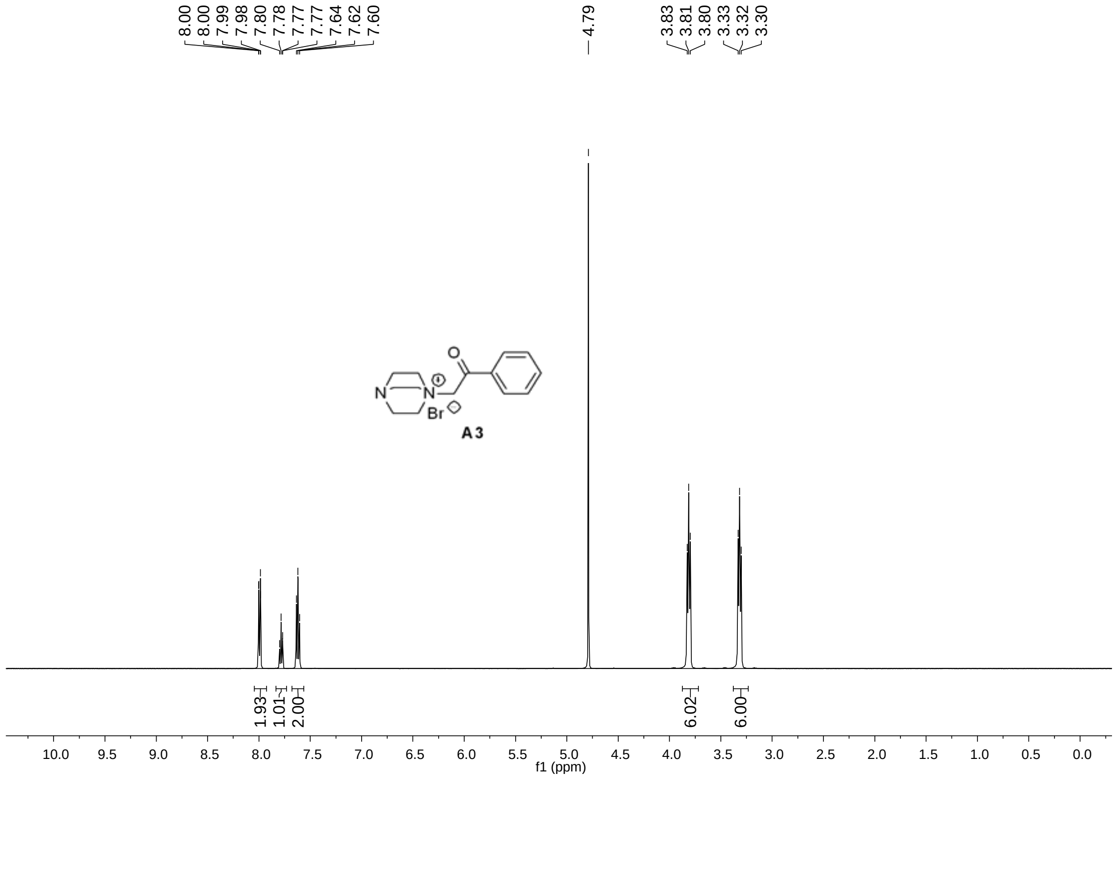
8.00
8.00
7.99
7.98
7.80
7.78
7.77
7.77
7.64
7.62
7.60
4.79
3.83
3.81
3.80
3.33
3.32
3.30
N
N
Br
A3
1.93
1.01
2.00
6.02
6.00
10.0 9.5 9.0 8.5 8.0 7.5 7.0 6.5 6.0 5.5 5.0 4.5 4.0 3.5 3.0 2.5 2.0 1.5 1.0 0.5 0.0
f1 (ppm)

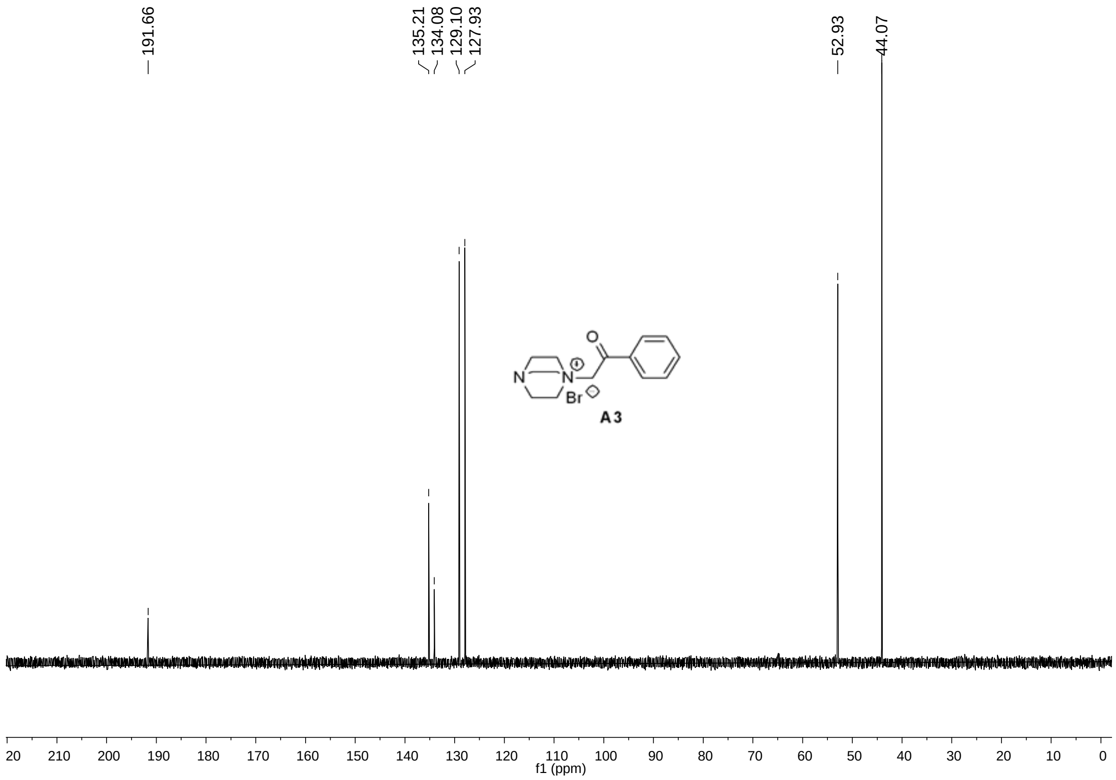
191.66
135.21
134.08
129.10
127.93
52.93
44.07
N
N
Br
O
A3
f1 (ppm)

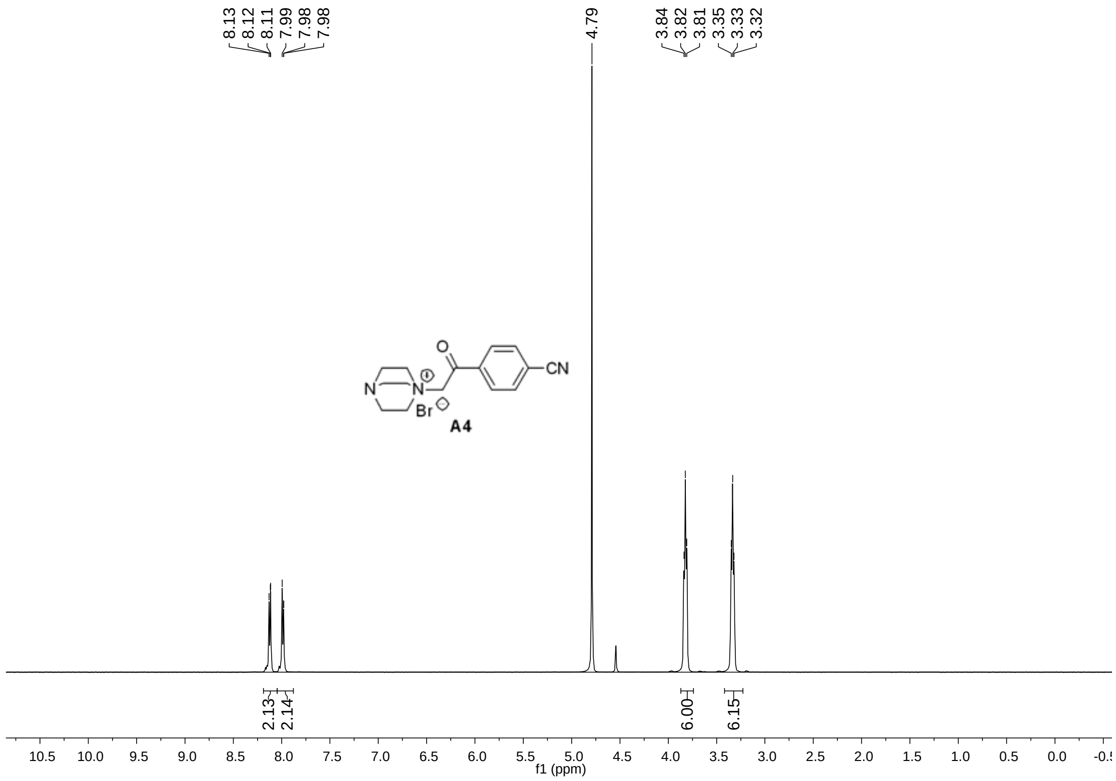
8.13
8.12
8.11
7.99
7.98
7.98
4.79
3.84
3.82
3.81
3.35
3.33
3.32
N
N
Br
O
CN
A4
2.13
2.14
6.00
6.15
f1 (ppm)

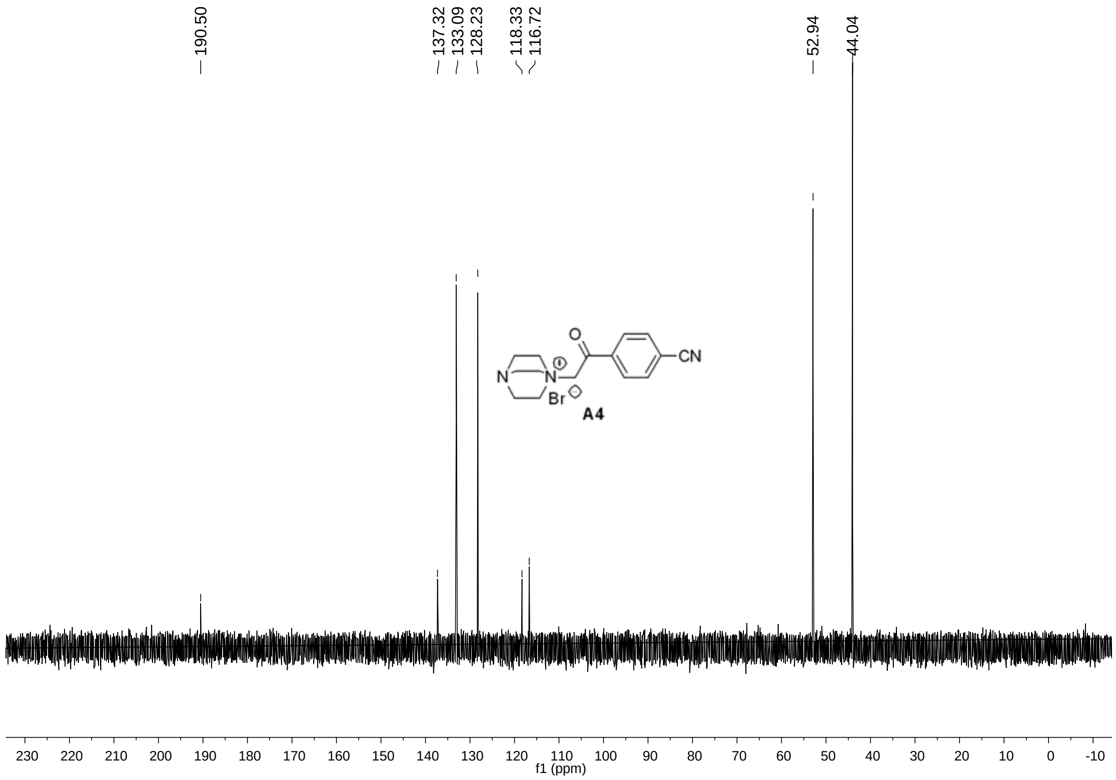
190.50
137.32
133.09
128.23
118.33
116.72
52.94
44.04
N
N
O
CN
Br
A4
230 220 210 200 190 180 170 160 150 140 130 120 110 100 90 80 70 60 50 40 30 20 10 0 -10
f1 (ppm)

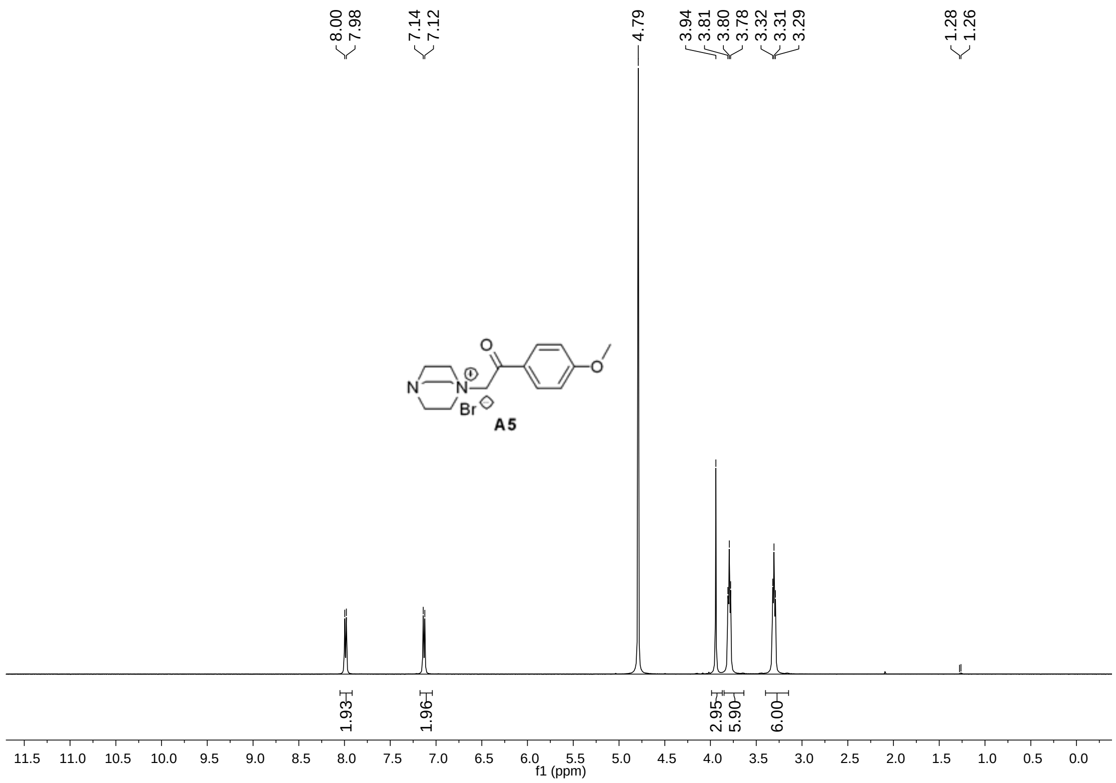
8.00
7.98
7.14
7.12
4.79
3.94
3.81
3.80
3.78
3.32
3.31
3.29
1.28
1.26
N
N
O
O
Br
A5
1.93
1.96
2.95
5.90
6.00
11.5 11.0 10.5 10.0 9.5 9.0 8.5 8.0 7.5 7.0 6.5 6.0 5.5 5.0 4.5 4.0 3.5 3.0 2.5 2.0 1.5 1.0 0.5 0.0
f1 (ppm)

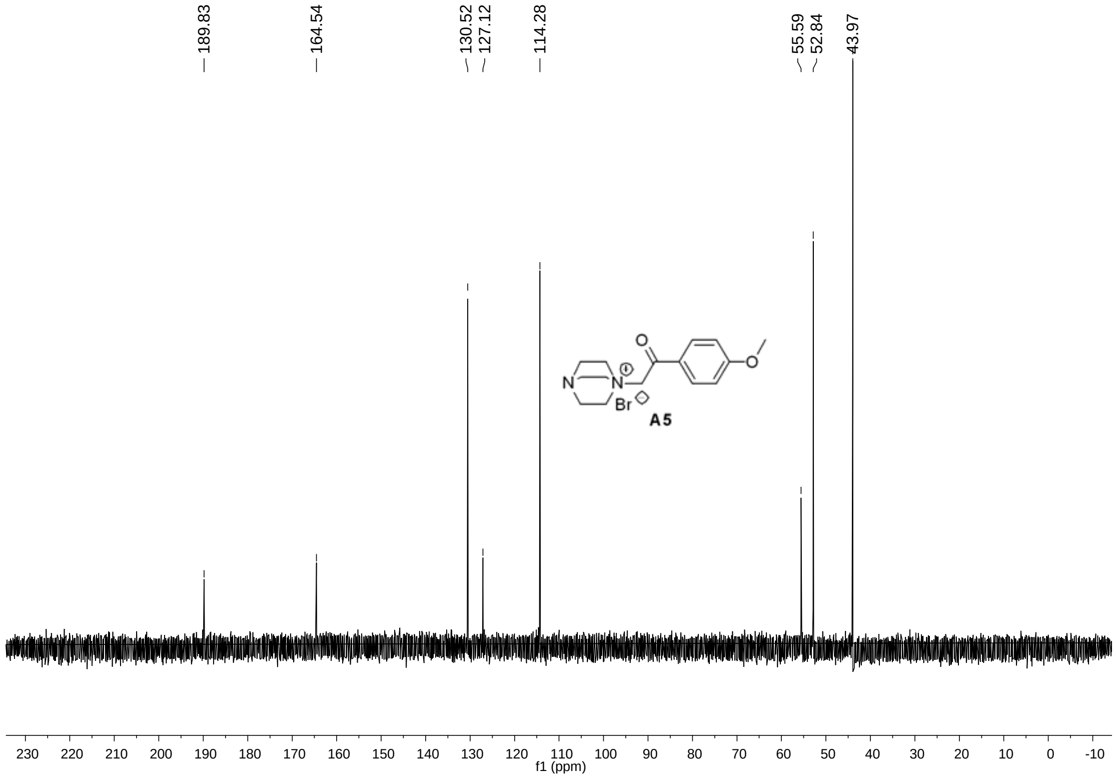
189.83
164.54
130.52
127.12
114.28
55.59
52.84
43.97
A5
f1 (ppm)

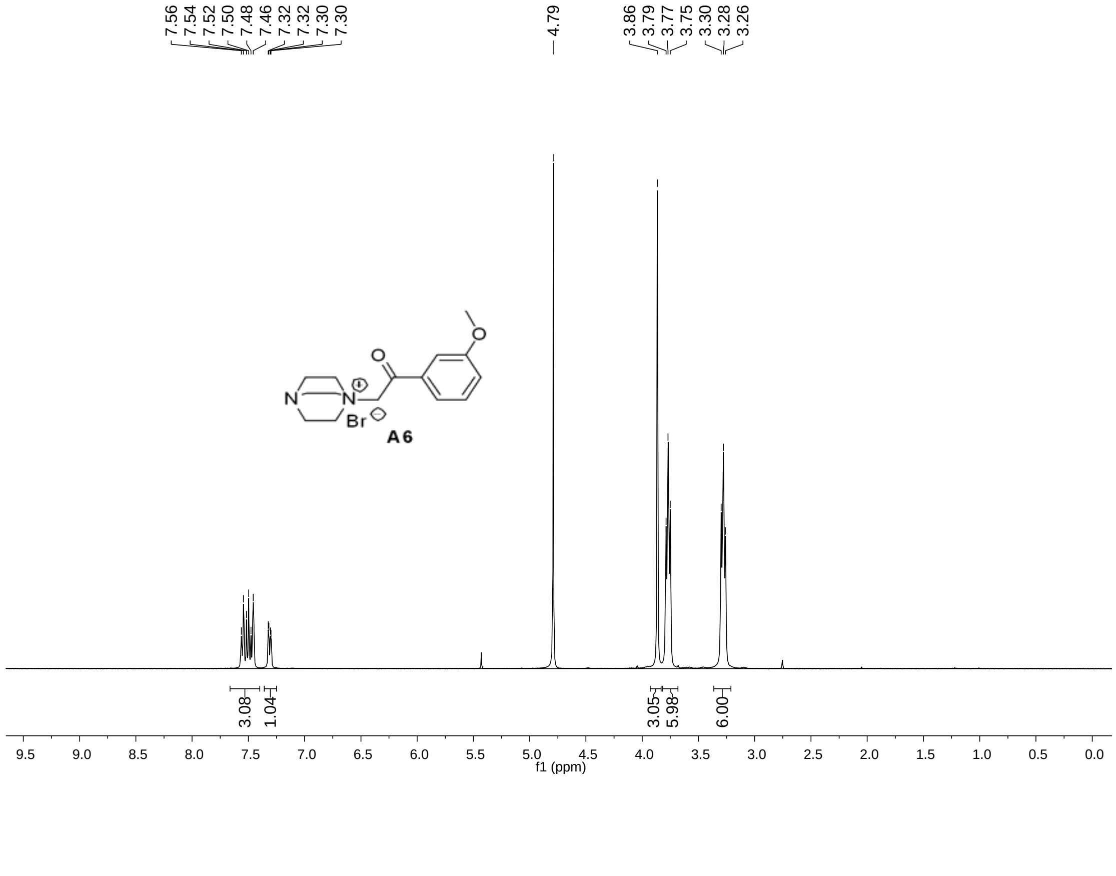
7.56
7.54
7.52
7.50
7.48
7.46
7.32
7.32
7.30
7.30
4.79
3.86
3.79
3.77
3.75
3.30
3.28
3.26
A6
3.08
1.04
3.05
5.98
6.00
f1 (ppm)

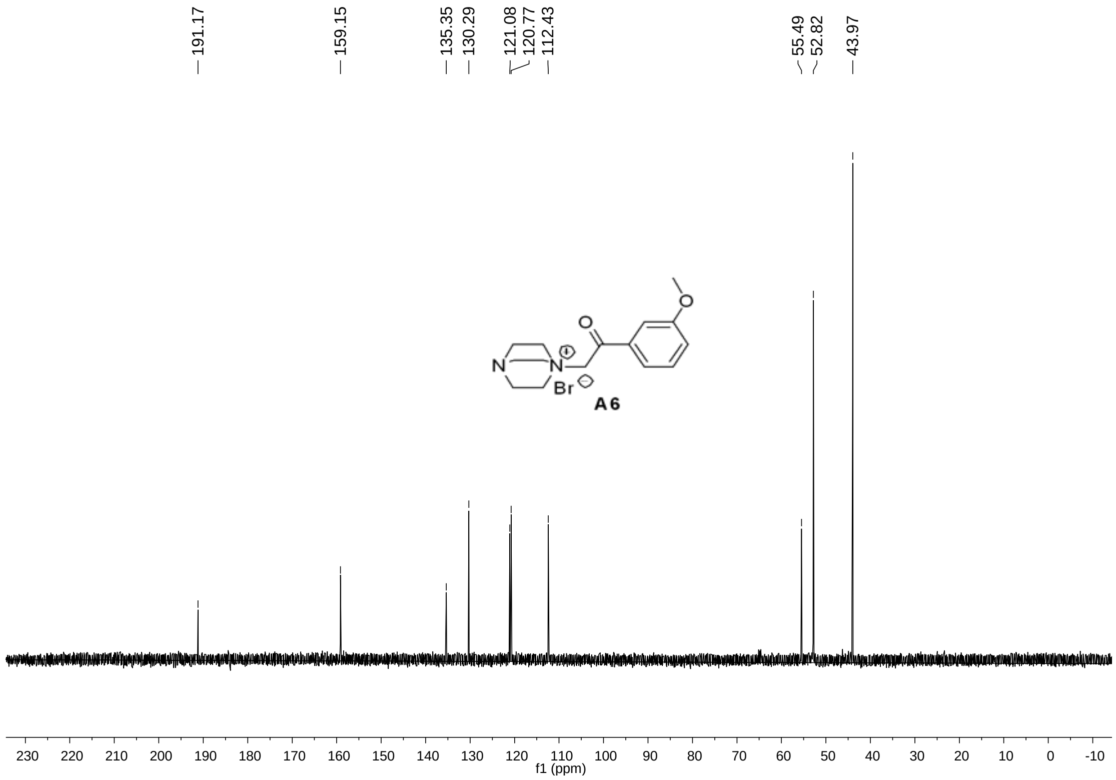
191.17
159.15
135.35
130.29
121.08
120.77
112.43
55.49
52.82
43.97
A6
f1 (ppm)

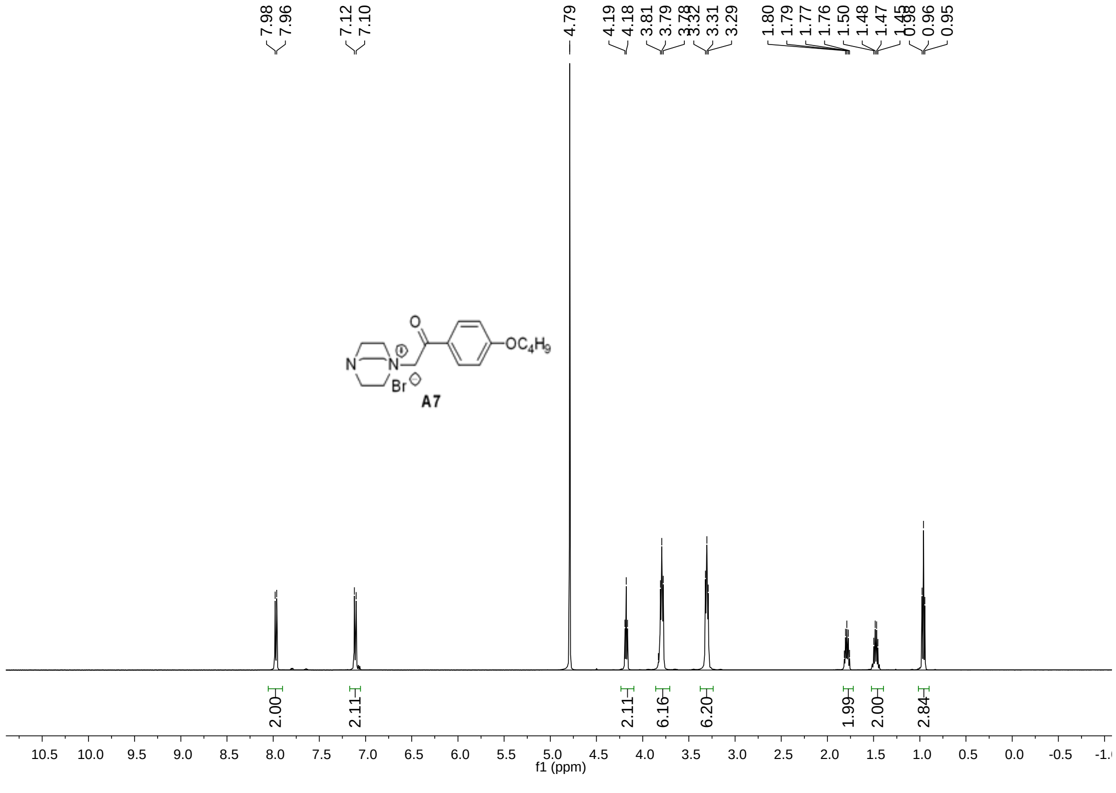
7.98
7.96
7.12
7.10
4.79
4.19
4.18
3.81
3.79
3.31
3.29
1.80
1.79
1.77
1.76
1.50
1.48
1.47
0.96
0.95
2.00
2.11
2.11
6.16
6.20
1.99
2.00
2.84
A7
f1 (ppm)

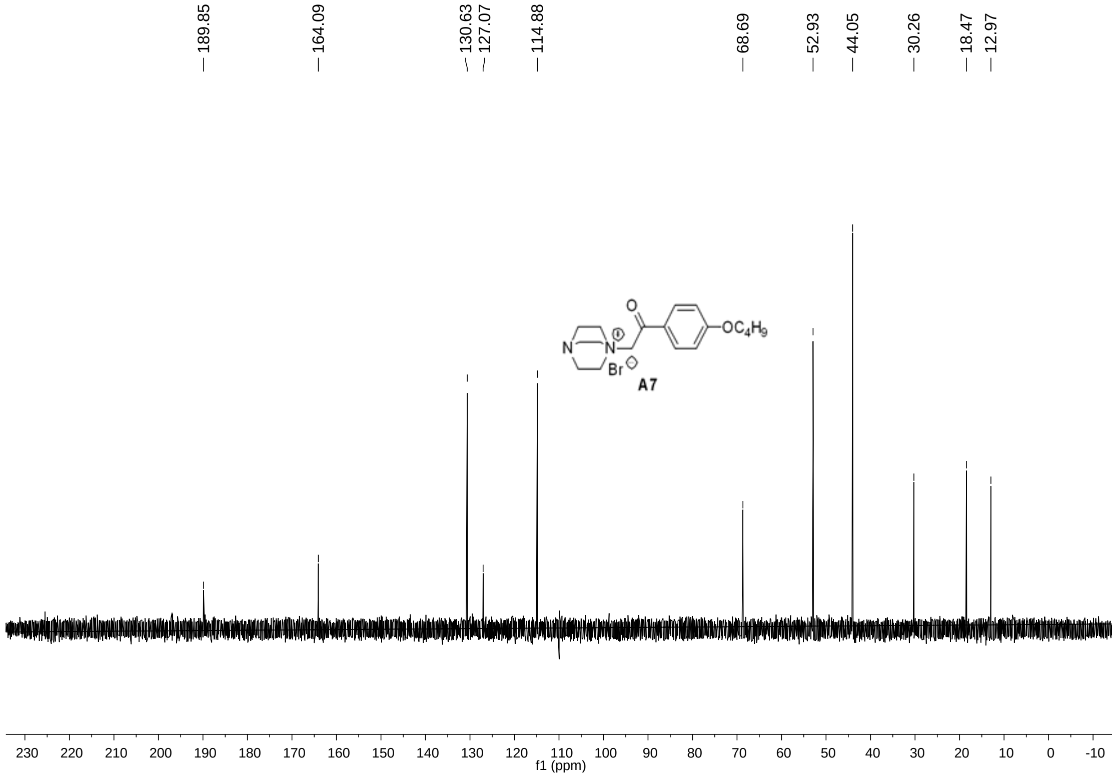
189.85
164.09
130.63
127.07
114.88
68.69
52.93
44.05
30.26
18.47
12.97
A7
f1 (ppm)

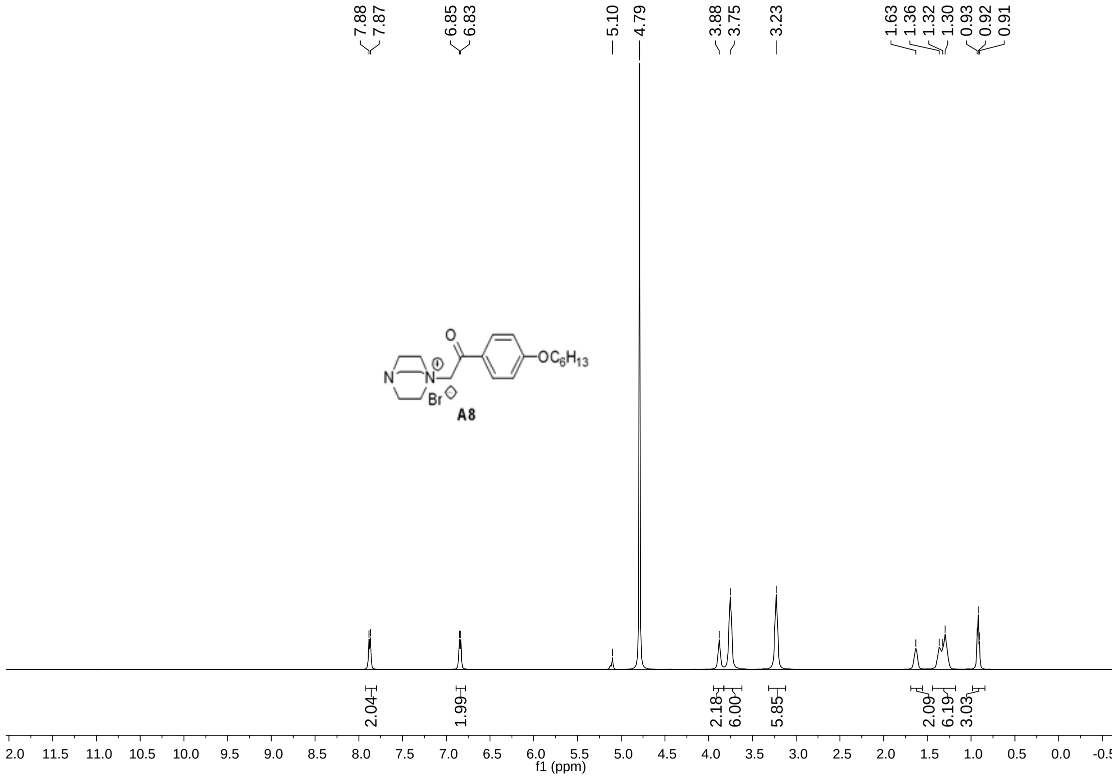
7.88
7.87
6.85
6.83
5.10
4.79
3.88
3.75
3.23
1.63
1.36
1.32
1.30
0.93
0.92
0.91
2.04
1.99
2.18
6.00
5.85
2.09
6.19
3.03
A8
f1 (ppm)

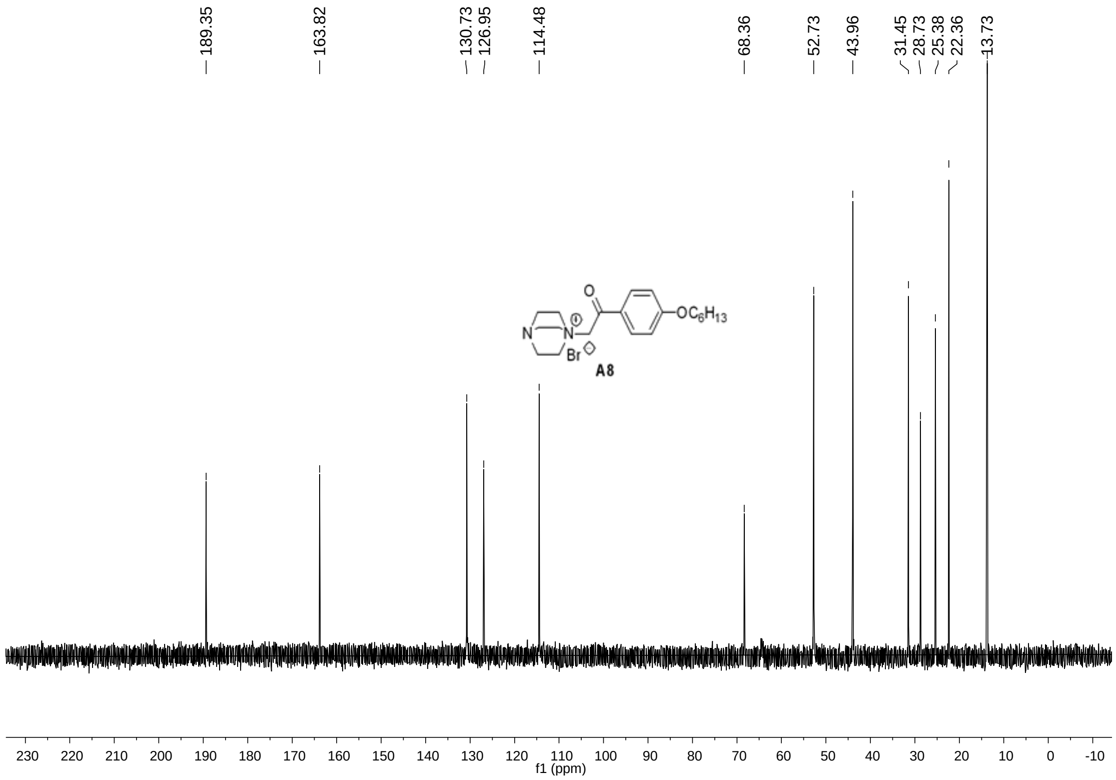

189.35
163.82
130.73
126.95
114.48
68.36
52.73
43.96
31.45
28.73
25.38
22.36
13.73
N
N
Br
O
OC6H13
A8
230 220 210 200 190 180 170 160 150 140 130 120 110 100 90 80 70 60 50 40 30 20 10 0 -10
f1 (ppm)

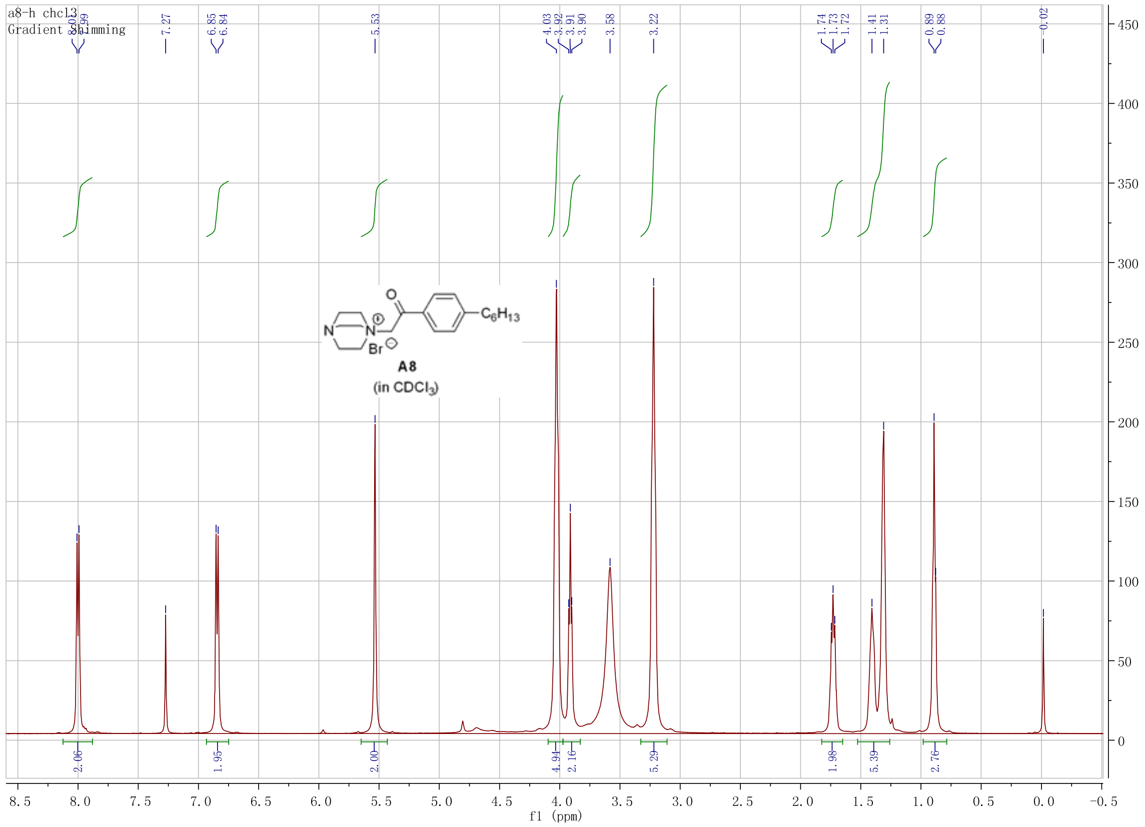

a8-h chcl3
Gradient shimming
7.27
6.85
6.84
5.53
4.03
3.92
3.91
3.90
3.58
3.22
1.74
1.73
1.72
1.41
1.31
0.89
0.88
0.02
N
N
Br
O
C6H13
A8
(in CDCl3)
2.06
1.95
2.00
4.94
2.16
5.29
1.98
5.39
2.76
8.5 8.0 7.5 7.0 6.5 6.0 5.5 5.0 4.5 4.0 3.5 3.0 2.5 2.0 1.5 1.0 0.5 0.0 -0.5
f1 (ppm)
450 400 350 300 250 200 150 100 50 0

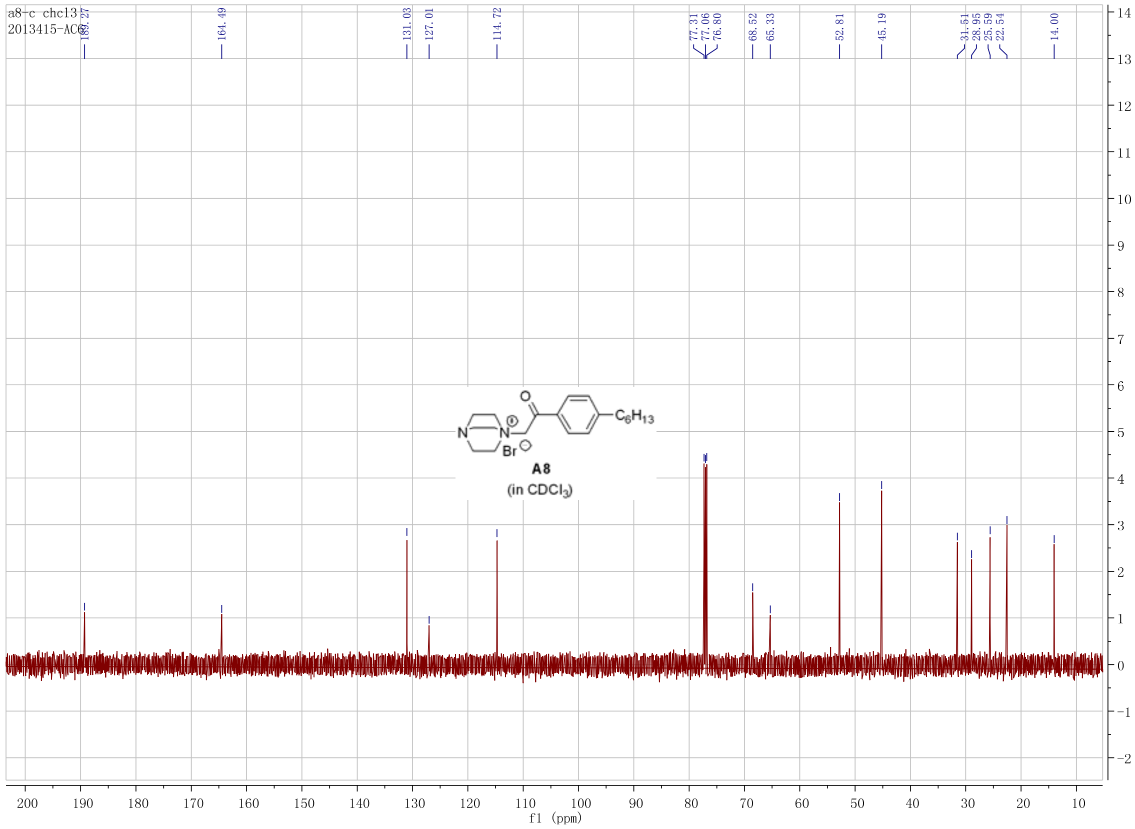

a8-c chcl3
2013415-AC6
189.27
164.49
131.03
127.01
114.72
77.31
77.06
76.80
68.52
65.33
52.81
45.19
31.51
28.95
25.59
22.54
14.00
A8
(in CDCl3)
f1 (ppm)

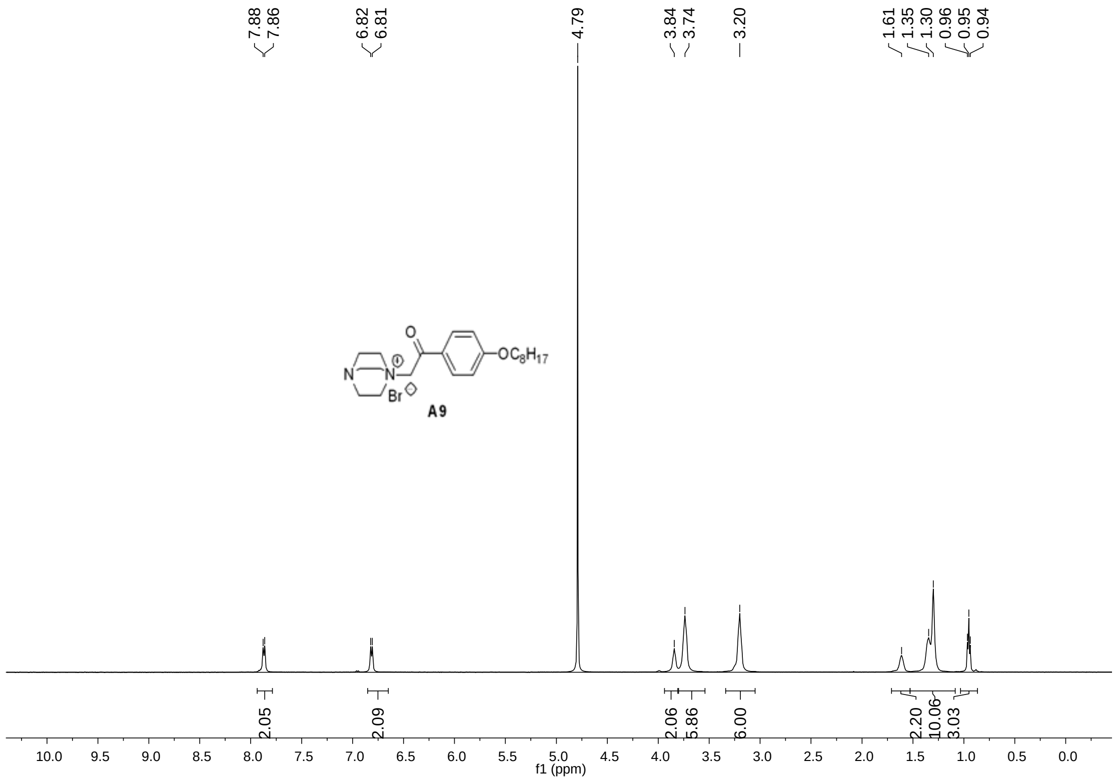

7.88
7.86
6.82
6.81
4.79
3.84
3.74
3.20
1.61
1.35
1.30
0.96
0.95
0.94
A9
2.05
2.09
2.06
5.86
6.00
2.20
10.06
3.03
f1 (ppm)

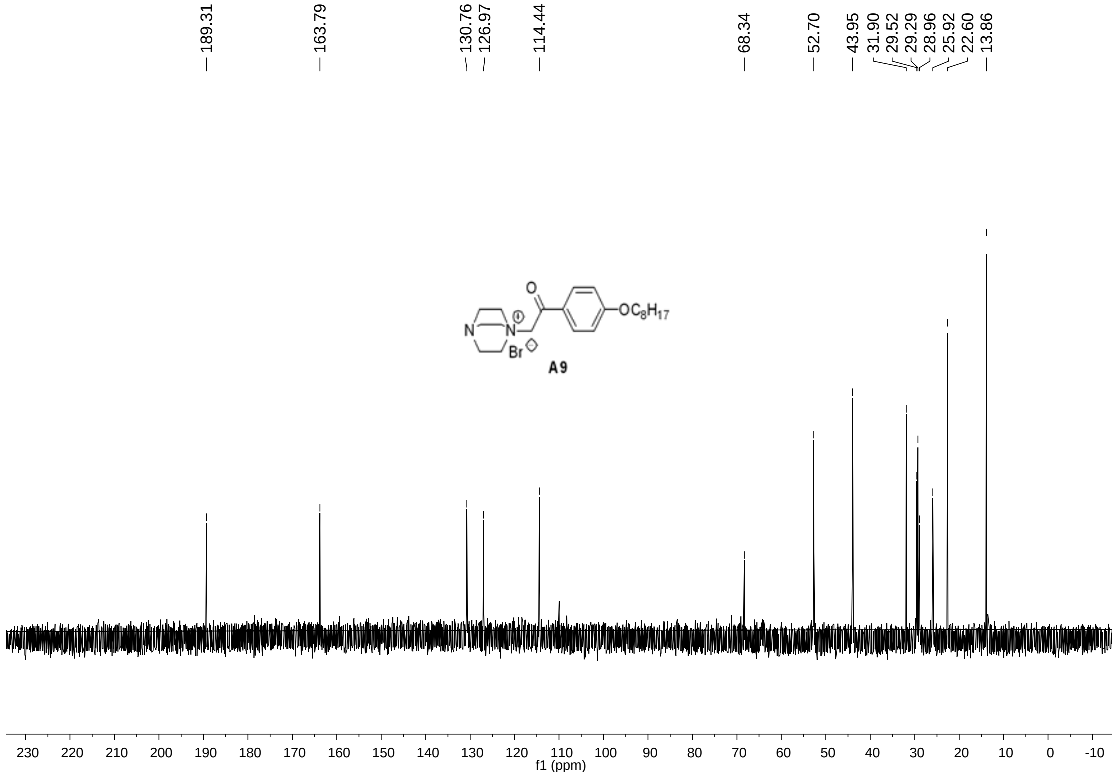
189.31
163.79
130.76
126.97
114.44
68.34
52.70
43.95
31.90
29.52
29.29
28.96
25.92
22.60
13.86
OC8H17
N
N
Br
O
A9
230 220 210 200 190 180 170 160 150 140 130 120 110 100 90 80 70 60 50 40 30 20 10 0 -10
f1 (ppm)

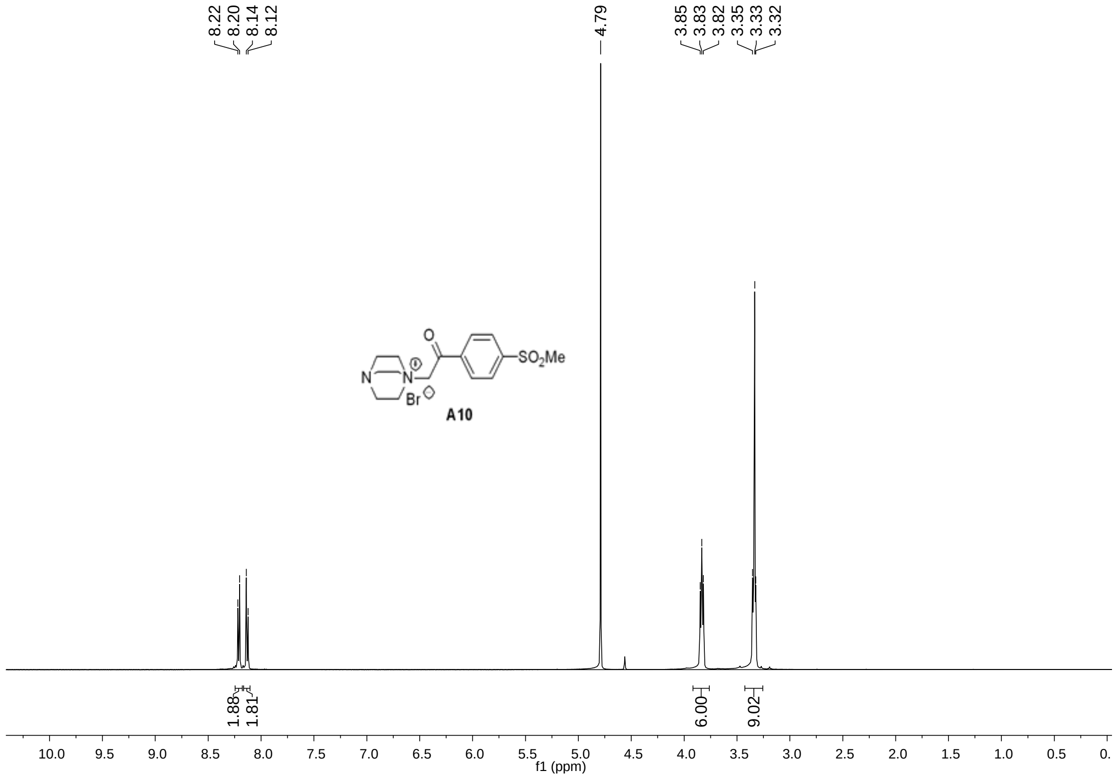
8.22
8.20
8.14
8.12
4.79
3.85
3.83
3.82
3.35
3.33
3.32
SO2Me
N
N
Br
O
A10
1.88
1.81
6.00
9.02
10.0 9.5 9.0 8.5 8.0 7.5 7.0 6.5 6.0 5.5 5.0 4.5 4.0 3.5 3.0 2.5 2.0 1.5 1.0 0.5 0.
f1 (ppm)

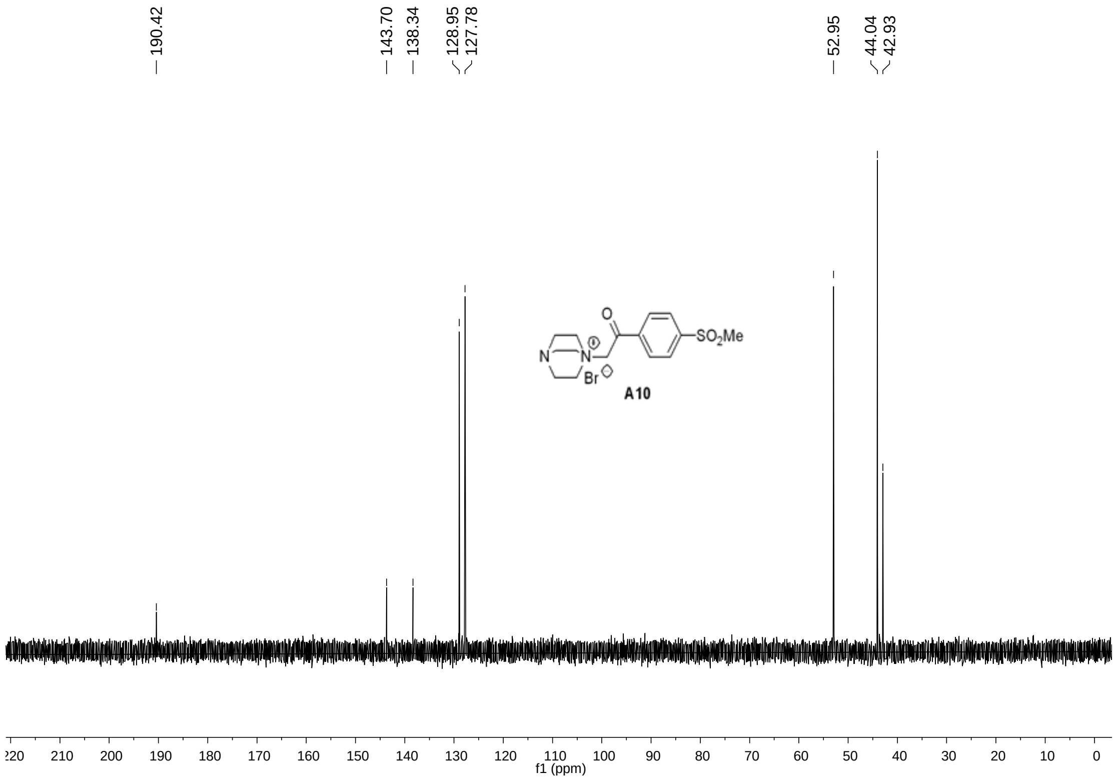
190.42
143.70
138.34
128.95
127.78
52.95
44.04
42.93
SO2Me
Br
A10
220 210 200 190 180 170 160 150 140 130 120 110 100 90 80 70 60 50 40 30 20 10 0
f1 (ppm)

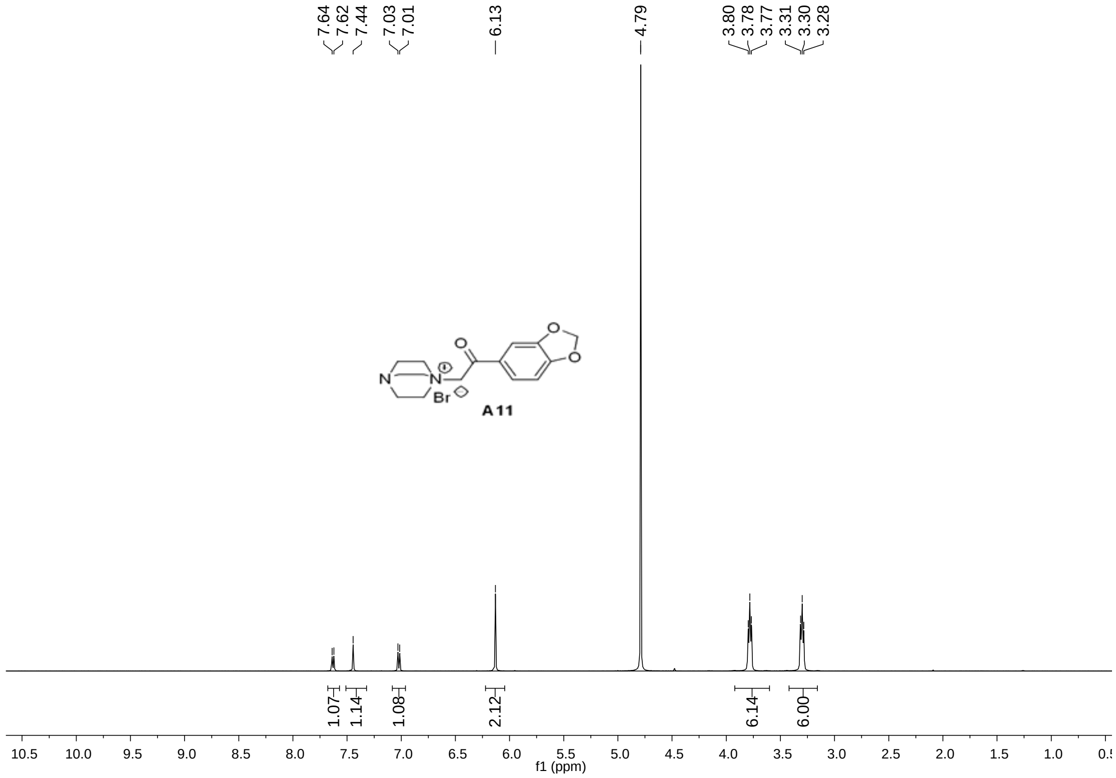
7.64
7.62
7.44
7.03
7.01
6.13
4.79
3.80
3.78
3.77
3.31
3.30
3.28
Br
A11
1.07
1.14
1.08
2.12
6.14
6.00
10.5 10.0 9.5 9.0 8.5 8.0 7.5 7.0 6.5 6.0 5.5 5.0 4.5 4.0 3.5 3.0 2.5 2.0 1.5 1.0
f1 (ppm)

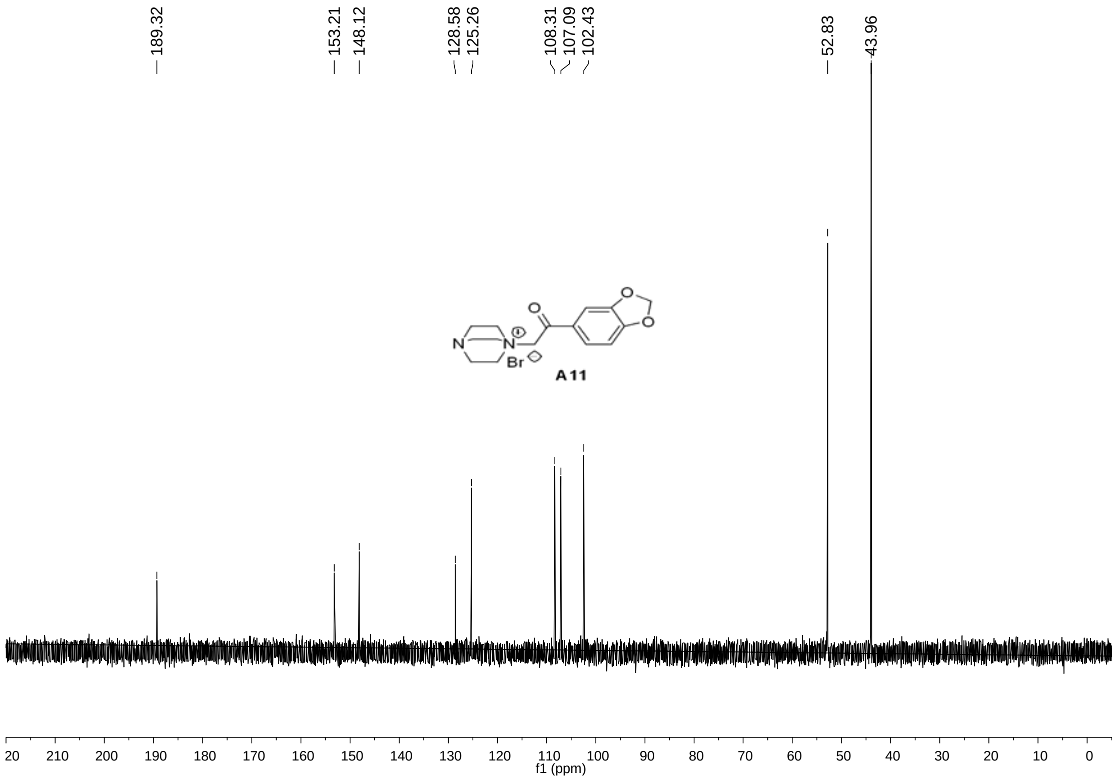
189.32
153.21
148.12
128.58
125.26
108.31
107.09
102.43
52.83
43.96
N
Br
O
A 11
f1 (ppm)

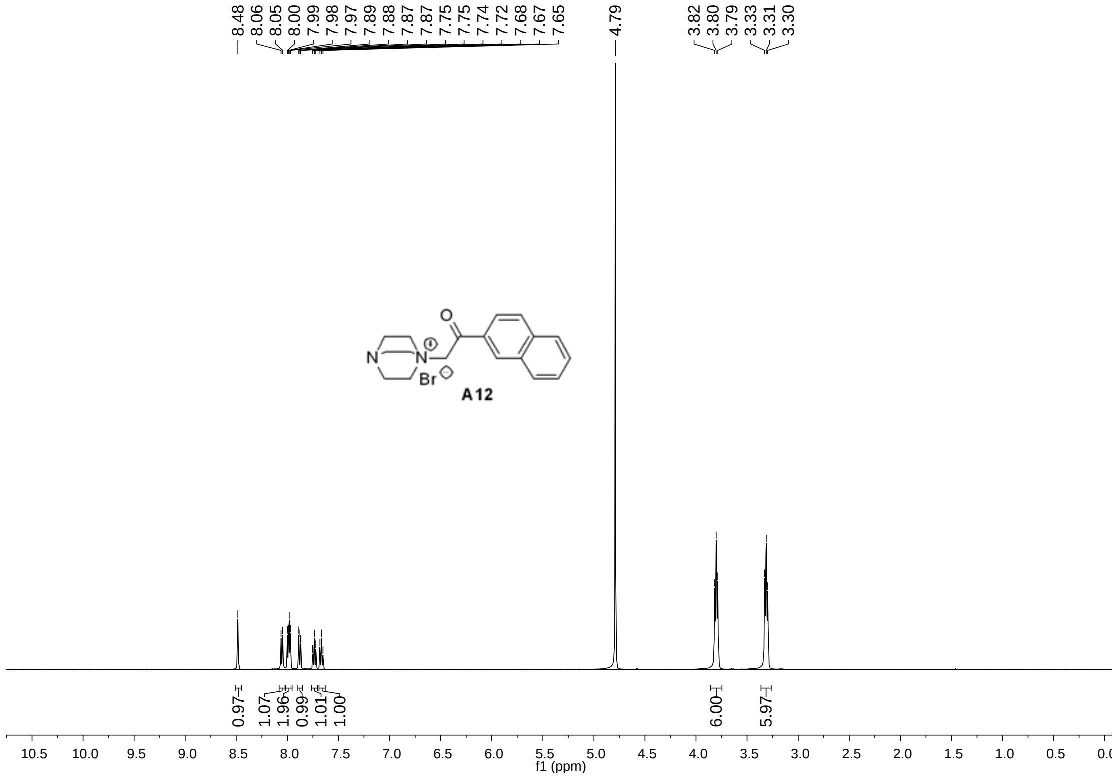
8.48
8.06
8.05
8.00
7.99
7.98
7.97
7.89
7.88
7.87
7.87
7.75
7.75
7.74
7.72
7.68
7.67
7.65
4.79
3.82
3.80
3.79
3.33
3.31
3.30
N
Br
O
A 12
0.97
1.07
1.96
0.99
1.01
1.00
6.00
5.97
f1 (ppm)

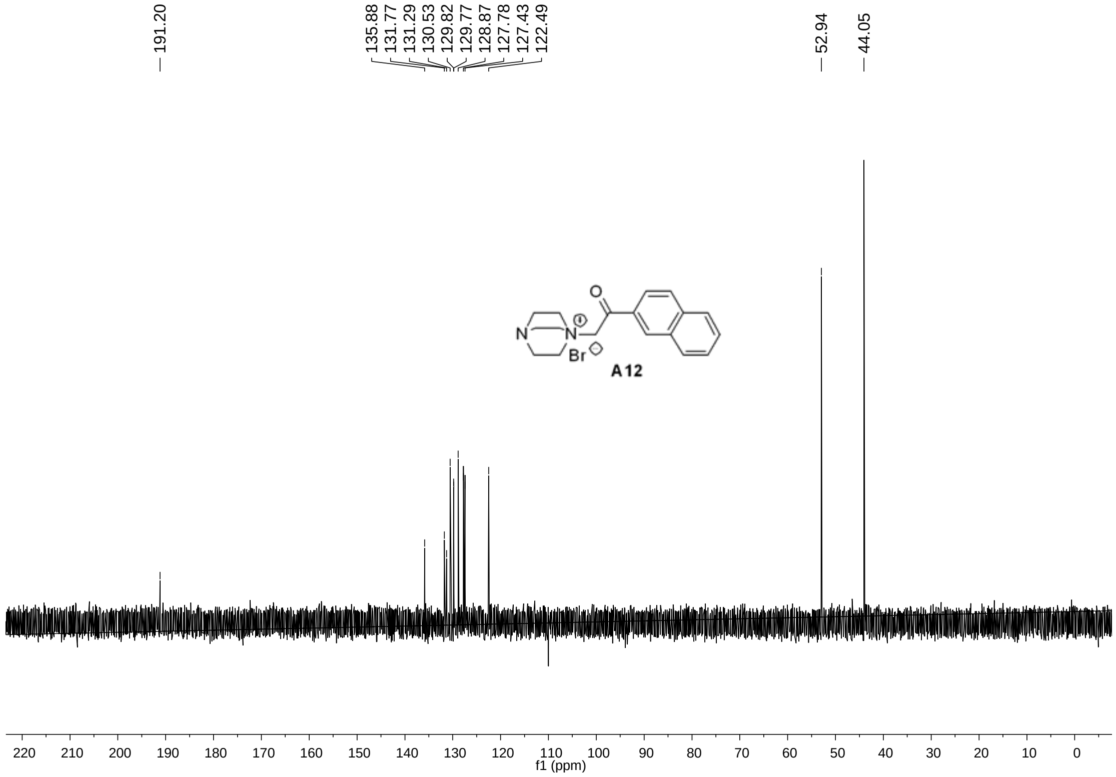
191.20
135.88
131.77
131.29
130.53
129.82
129.77
128.87
127.78
127.43
122.49
52.94
44.05
N
N
Br
O
A12
220 210 200 190 180 170 160 150 140 130 120 110 100 90 80 70 60 50 40 30 20 10 0
f1 (ppm)

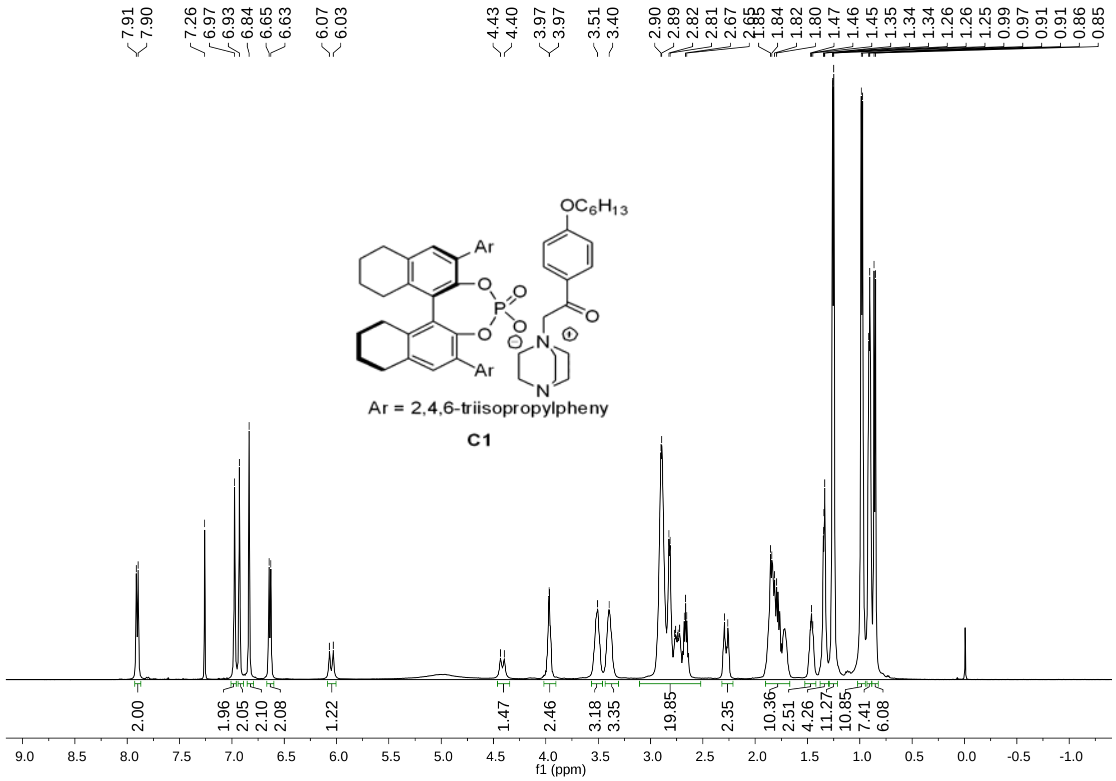
7.91
7.90
7.26
6.97
6.93
6.84
6.65
6.63
6.07
6.03
4.43
4.40
3.97
3.97
3.51
3.40
2.90
2.89
2.82
2.81
2.67
2.65
1.85
1.84
1.82
1.80
1.47
1.46
1.45
1.35
1.34
1.26
1.25
0.99
0.97
0.91
0.91
0.86
0.85
OC6H13
Ar
O
P
O
O
O
N
N
Ar = 2,4,6-triisopropylpheny
C1
2.00
1.96
2.05
2.10
2.08
1.22
1.47
2.46
3.18
3.35
19.85
2.35
10.36
2.51
4.26
11.27
10.85
7.41
6.08
9.0 8.5 8.0 7.5 7.0 6.5 6.0 5.5 5.0 4.5 4.0 3.5 3.0 2.5 2.0 1.5 1.0 0.5 0.0 -0.5 -1.0
f1 (ppm)

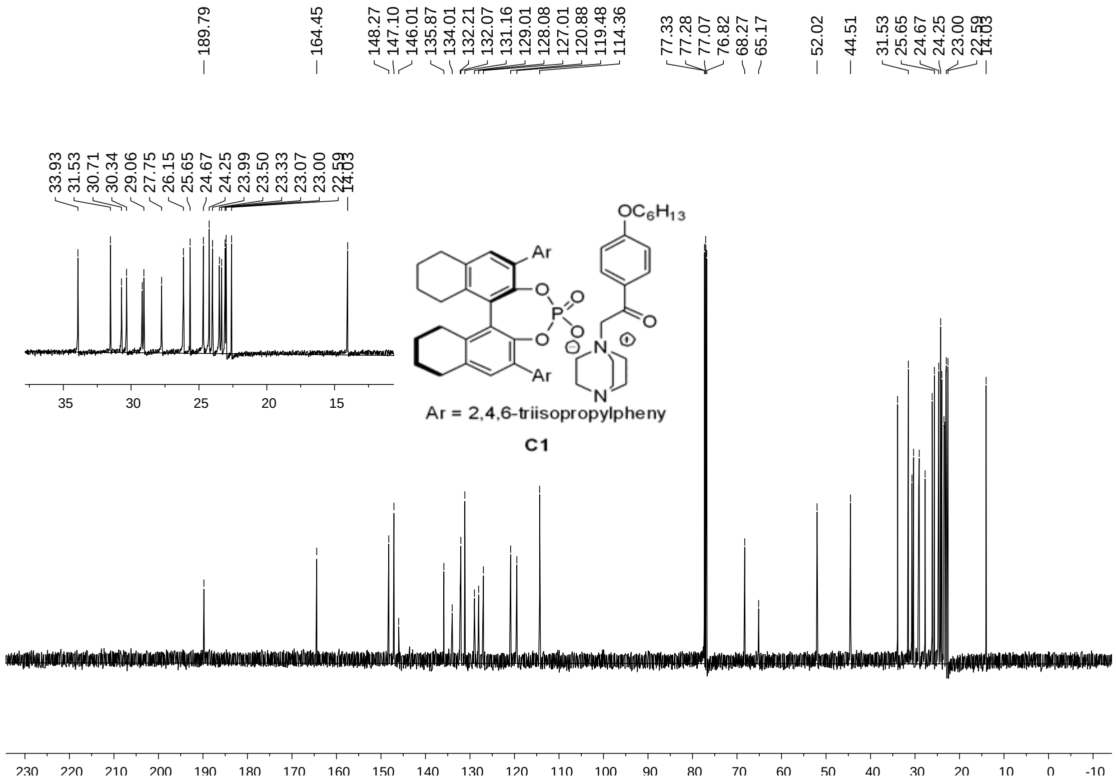

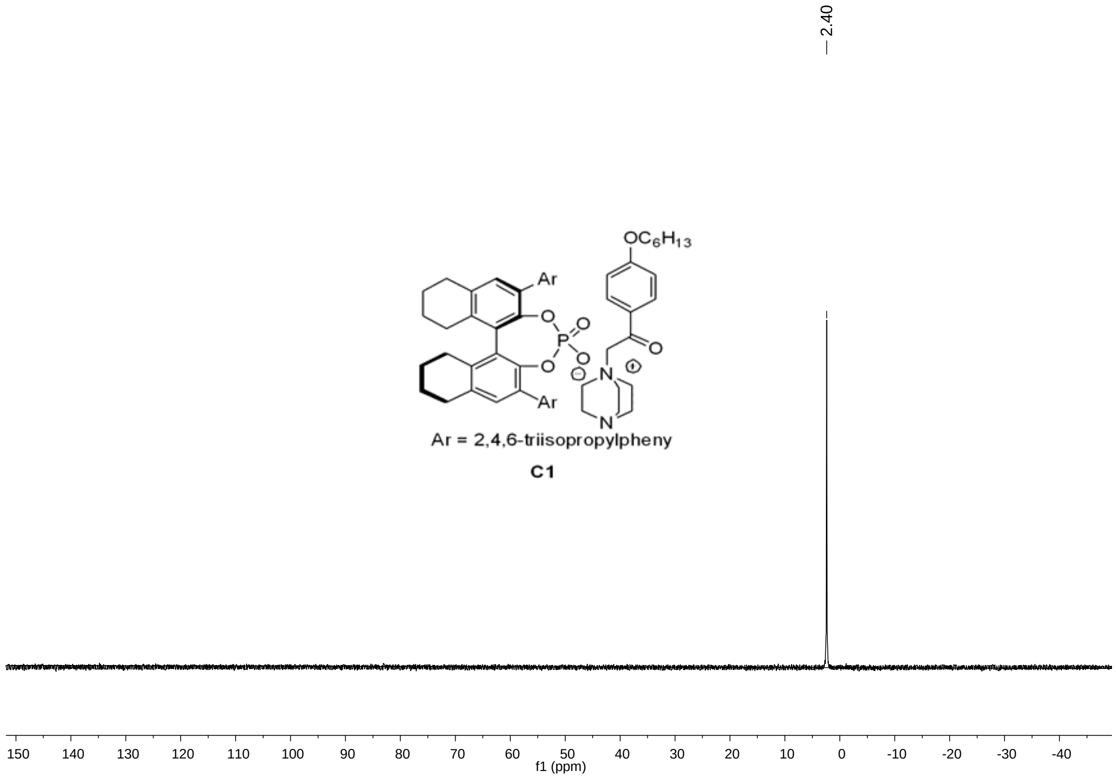

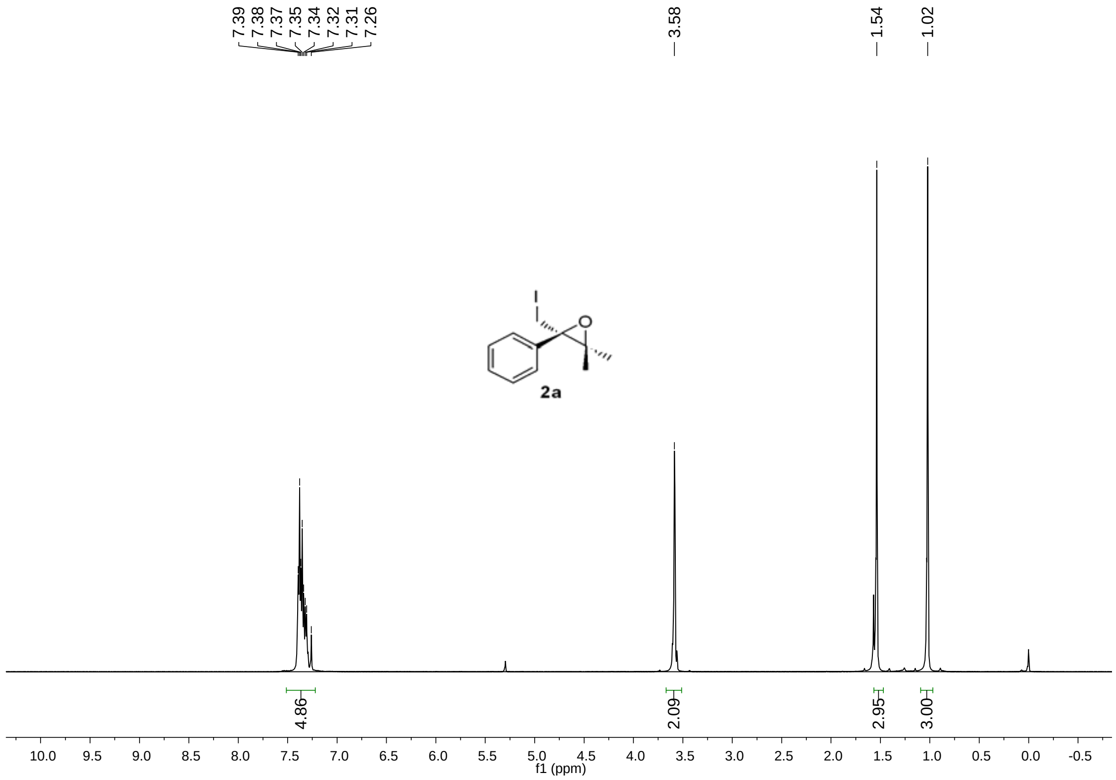
7.39
7.38
7.37
7.35
7.34
7.32
7.31
7.26
3.58
1.54
1.02
2a
4.86
2.09
2.95
3.00
10.0 9.5 9.0 8.5 8.0 7.5 7.0 6.5 6.0 5.5 5.0 4.5 4.0 3.5 3.0 2.5 2.0 1.5 1.0 0.5 0.0 -0.5
f1 (ppm)

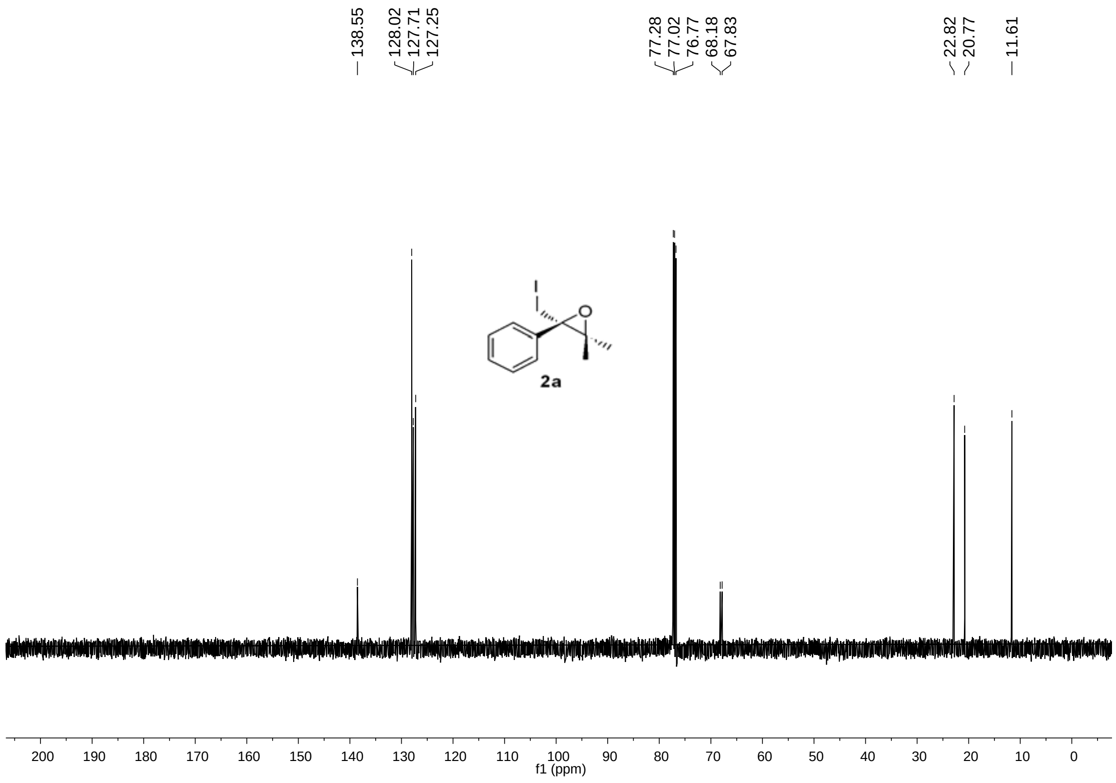
138.55
128.02
127.71
127.25
77.28
77.02
76.77
68.18
67.83
22.82
20.77
11.61
2a
200 190 180 170 160 150 140 130 120 110 100 90 80 70 60 50 40 30 20 10 0
f1 (ppm)

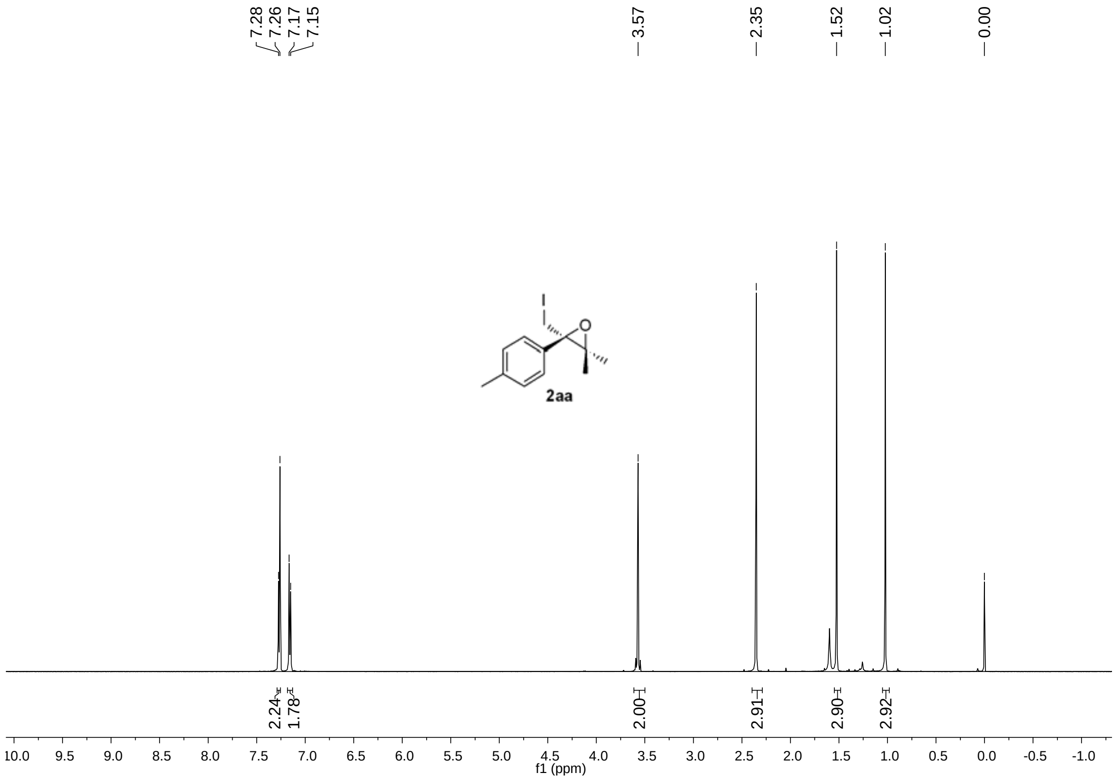

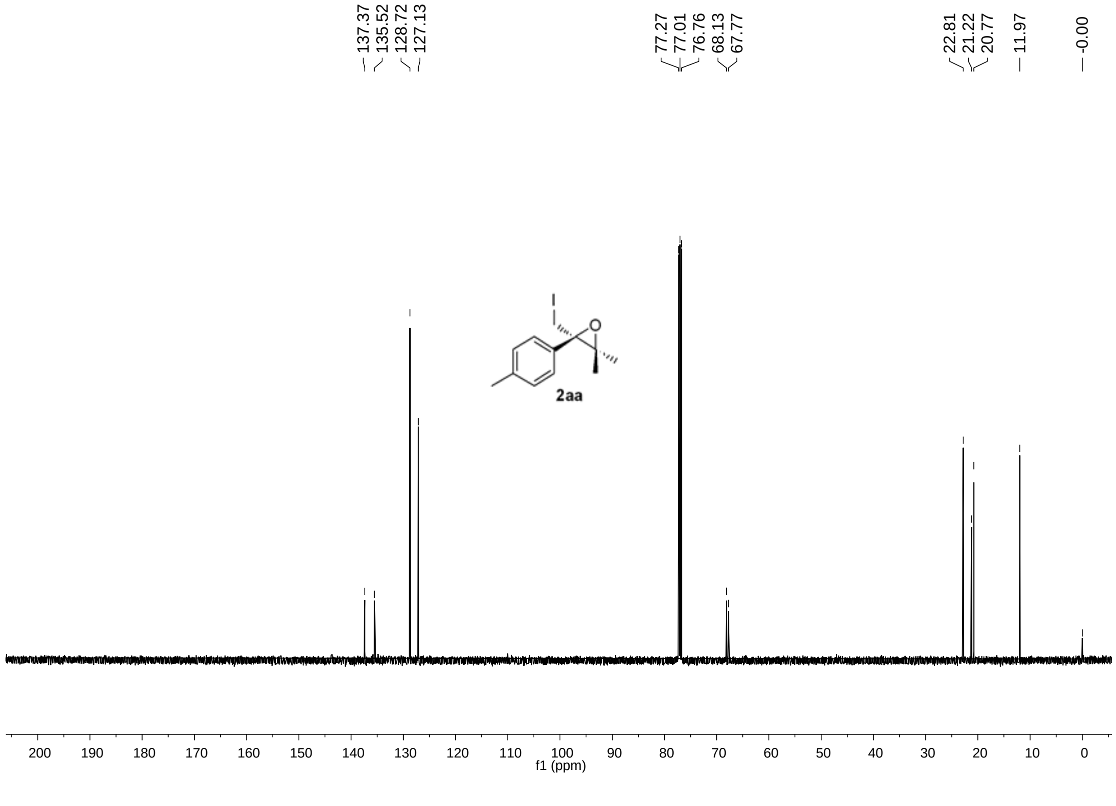

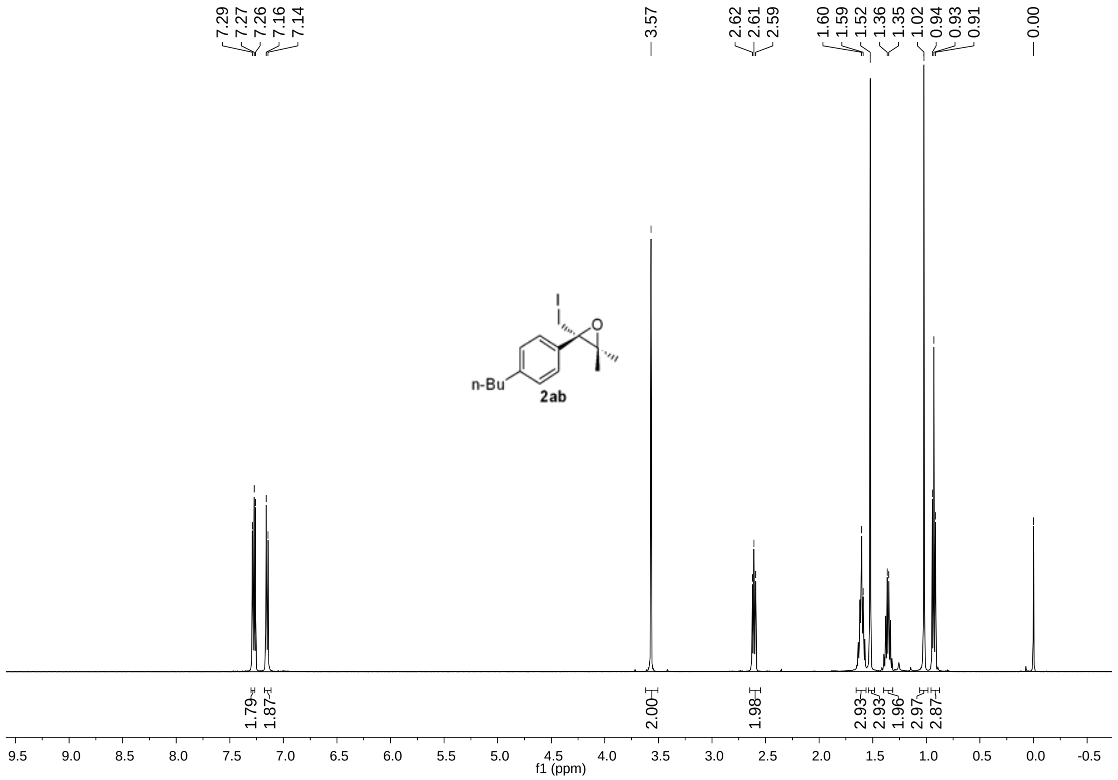
7.29
7.27
7.26
7.16
7.14
3.57
2.62
2.61
2.59
1.60
1.59
1.52
1.36
1.35
1.02
0.94
0.93
0.91
0.00
n-Bu
2ab
1.79
1.87
2.00
1.98
2.93
2.93
1.96
2.97
2.87
f1 (ppm)

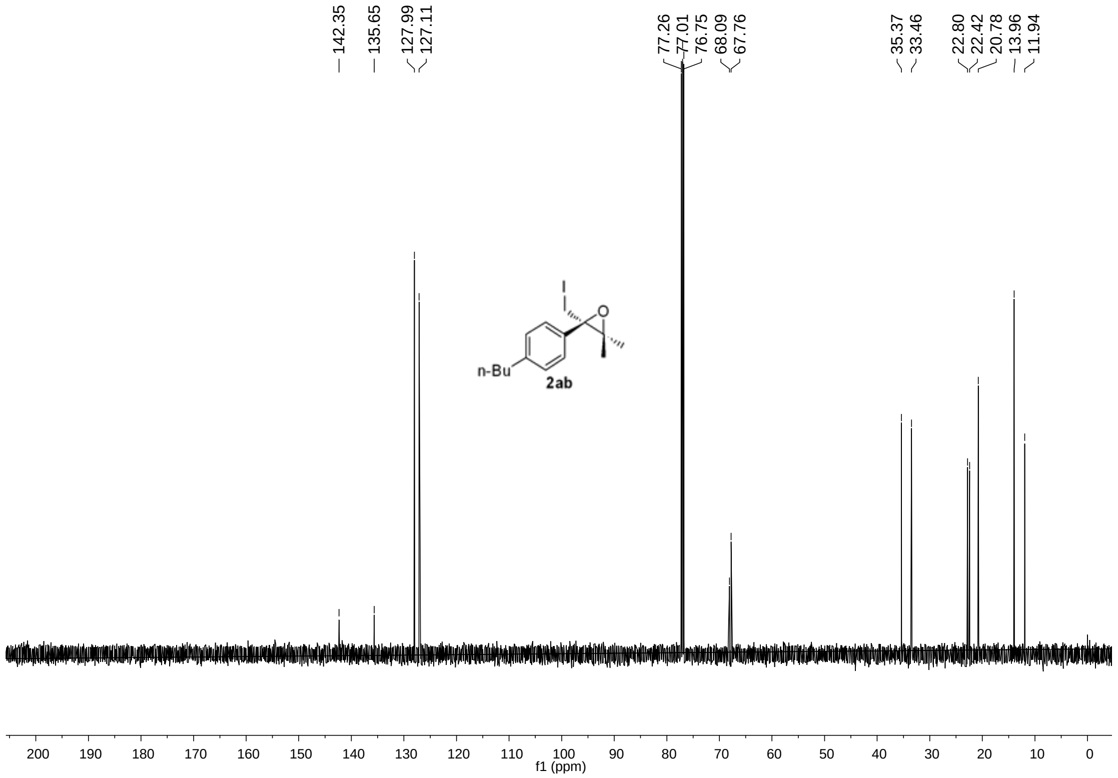
142.35
135.65
127.99
127.11
77.26
77.01
76.75
68.09
67.76
35.37
33.46
22.80
22.42
20.78
13.96
11.94
n-Bu
2ab
f1 (ppm)

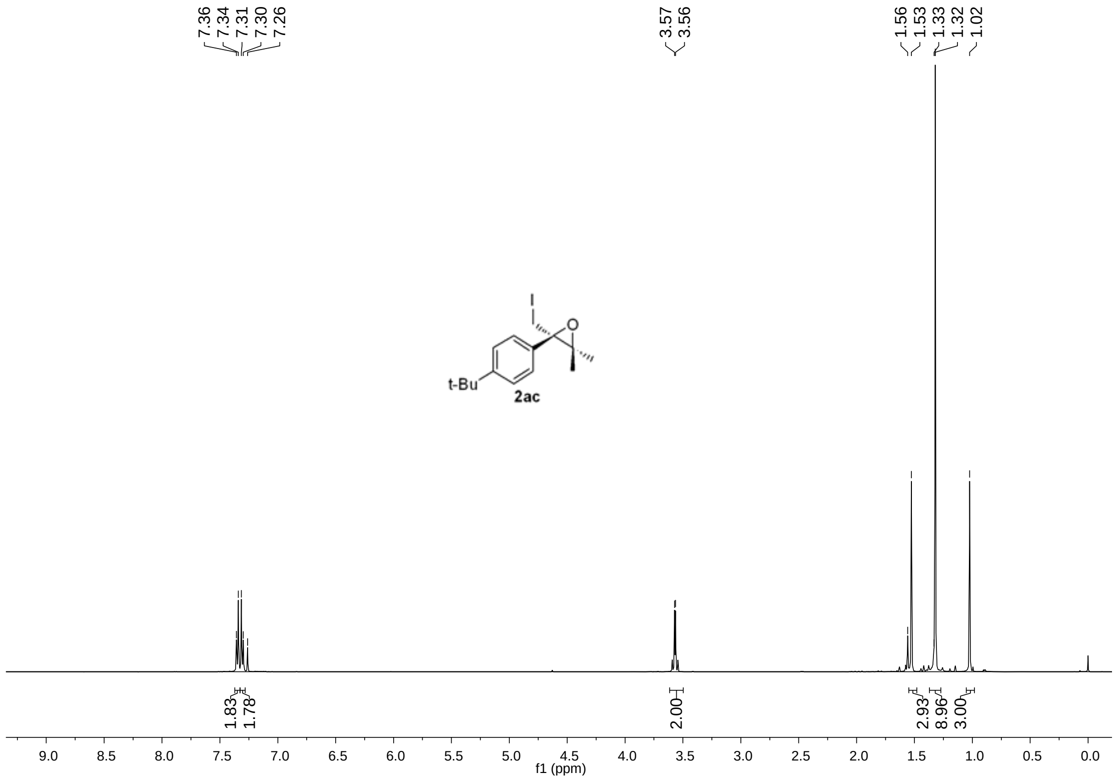
7.36
7.34
7.31
7.30
7.26
3.57
3.56
1.56
1.53
1.33
1.32
1.02
t-Bu
2ac
1.83
1.78
2.00
2.93
8.96
3.00
f1 (ppm)

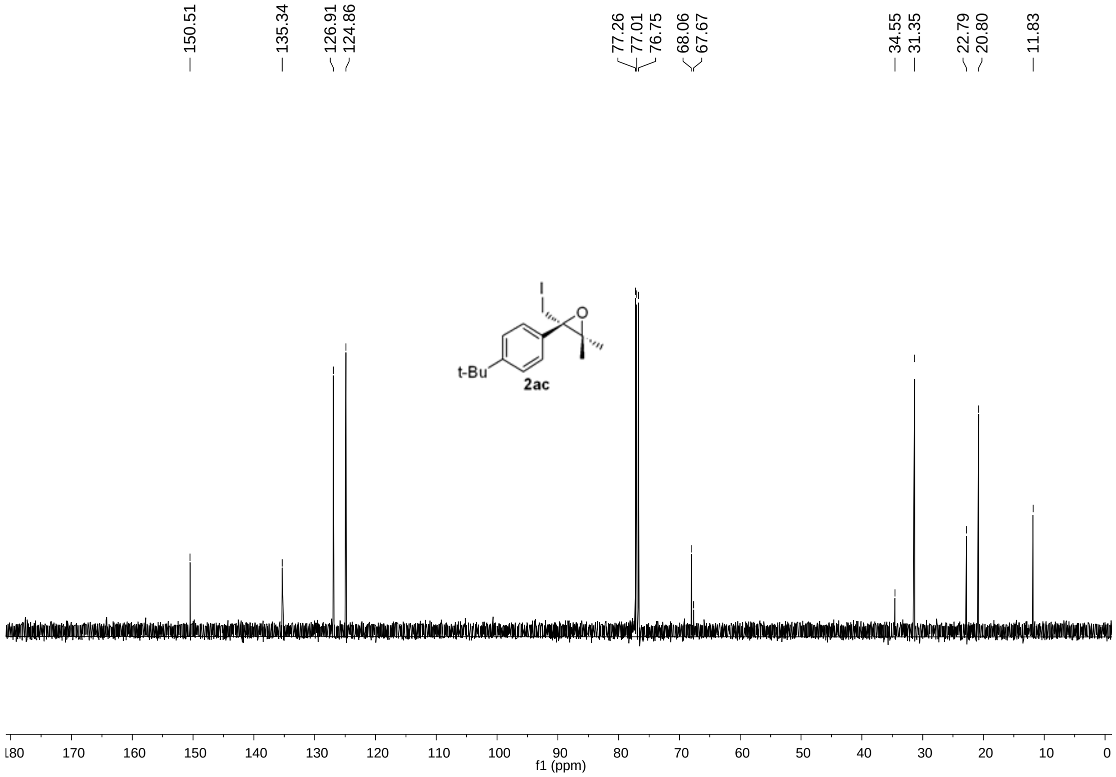
150.51
135.34
126.91
124.86
77.26
77.01
76.75
68.06
67.67
34.55
31.35
22.79
20.80
11.83
t-Bu
2ac
f1 (ppm)

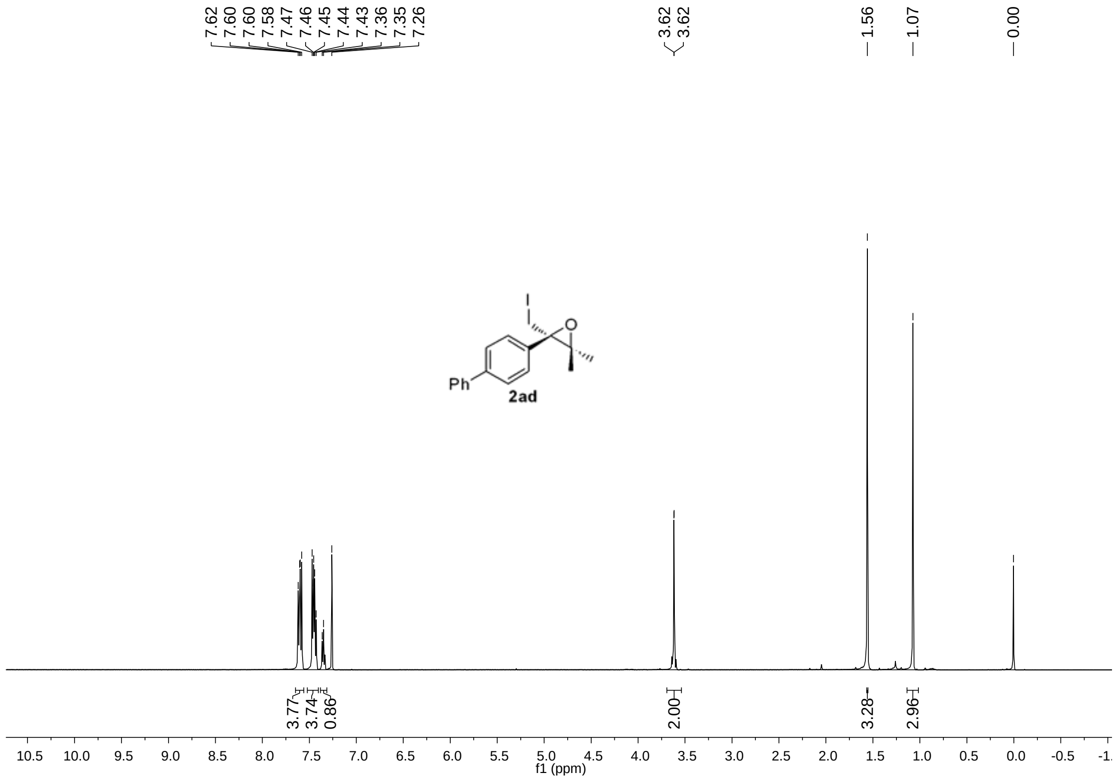
7.62 7.60 7.60 7.58 7.47 7.46 7.45 7.44 7.43 7.36 7.35 7.26
3.62 3.62
1.56
1.07
0.00
Ph
2ad
3.77 3.74 0.86
2.00
3.28
2.96
f1 (ppm)

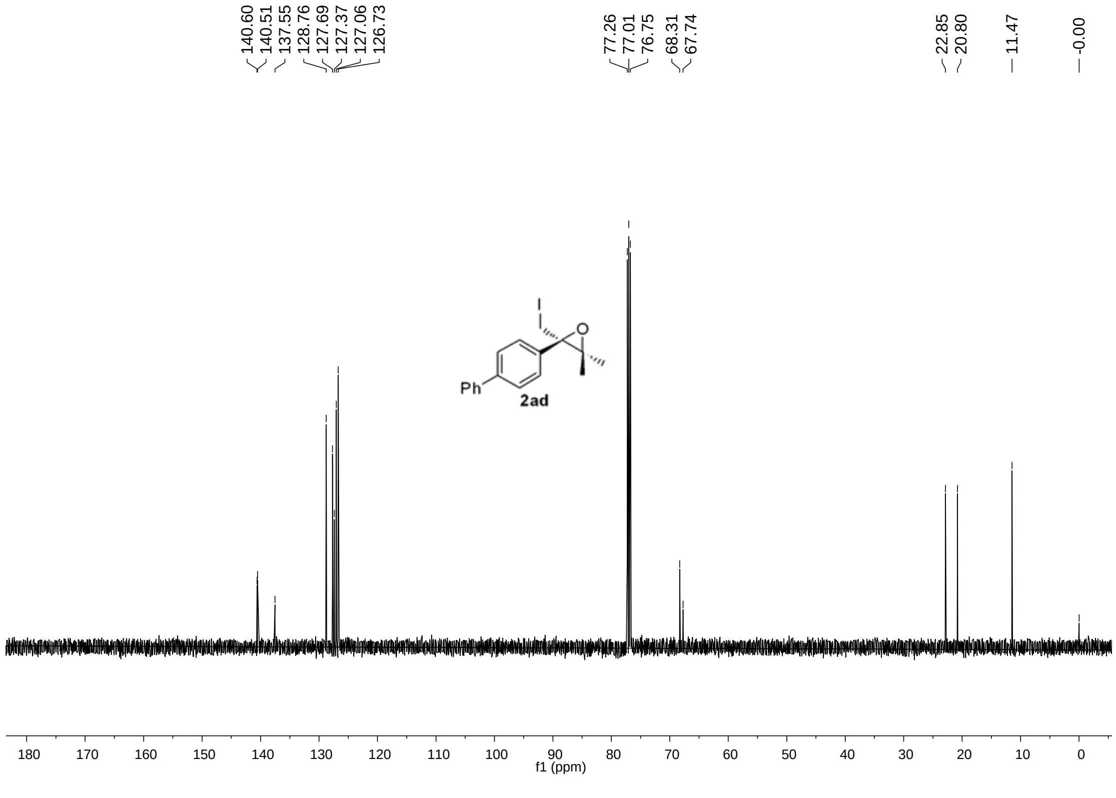
140.60 140.51 137.55 128.76 127.69 127.37 127.06 126.73
77.26 77.01 76.75 68.31 67.74
22.85 20.80
11.47
-0.00
Ph
2ad
f1 (ppm)

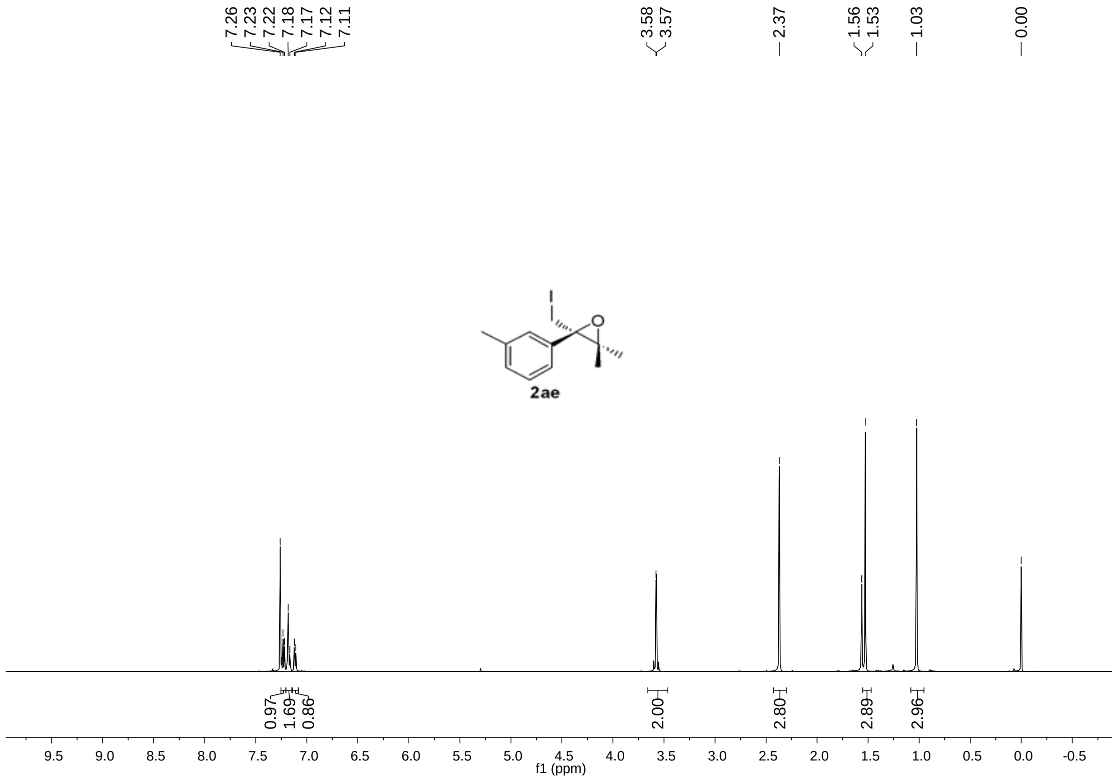
7.26
7.23
7.22
7.18
7.17
7.12
7.11
3.58
3.57
2.37
1.56
1.53
1.03
0.00
2ae
0.97
1.69
0.86
2.00
2.80
2.89
2.96
f1 (ppm)

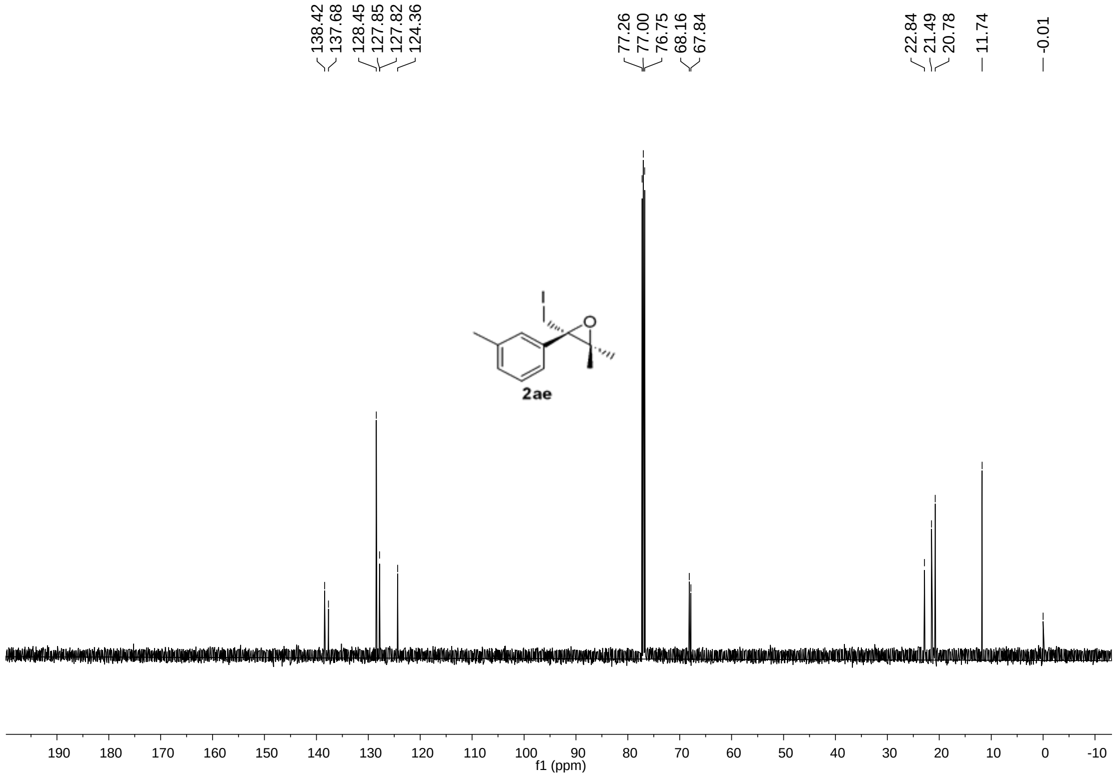
138.42
137.68
128.45
127.85
127.82
124.36
77.26
77.00
76.75
68.16
67.84
22.84
21.49
20.78
11.74
-0.01
2ae
f1 (ppm)

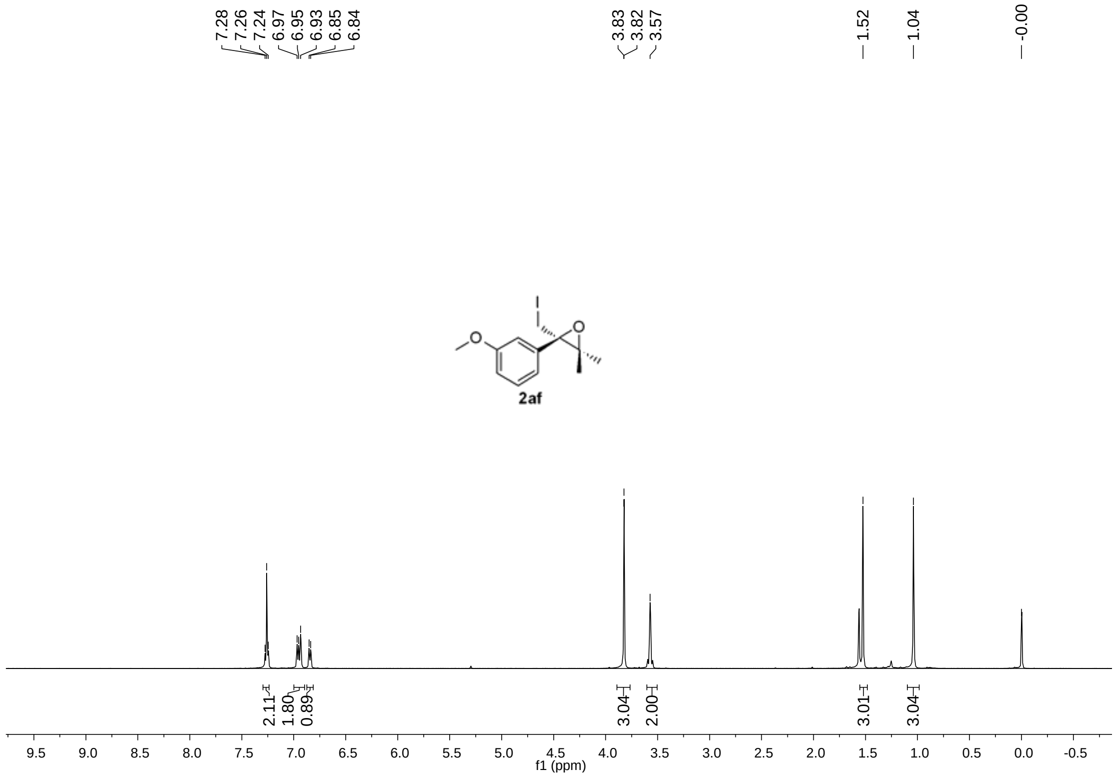

7.28
7.26
7.24
6.97
6.95
6.93
6.85
6.84
3.83
3.82
3.57
1.52
1.04
-0.00
2af
2.11
1.80
0.89
3.04
2.00
3.01
3.04
9.5 9.0 8.5 8.0 7.5 7.0 6.5 6.0 5.5 5.0 4.5 4.0 3.5 3.0 2.5 2.0 1.5 1.0 0.5 0.0 -0.5
f1 (ppm)

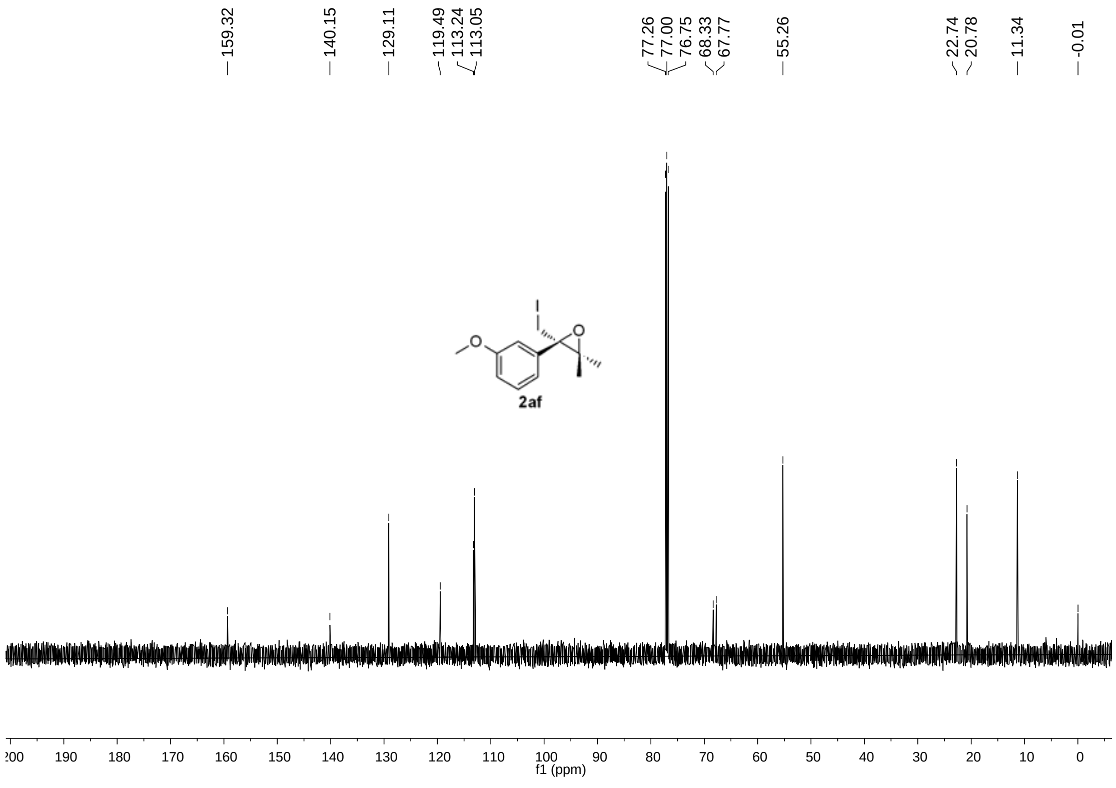

159.32
140.15
129.11
119.49
113.24
113.05
77.26
77.00
76.75
68.33
67.77
55.26
22.74
20.78
11.34
-0.01
2af
190 180 170 160 150 140 130 120 110 100 90 80 70 60 50 40 30 20 10 0
f1 (ppm)

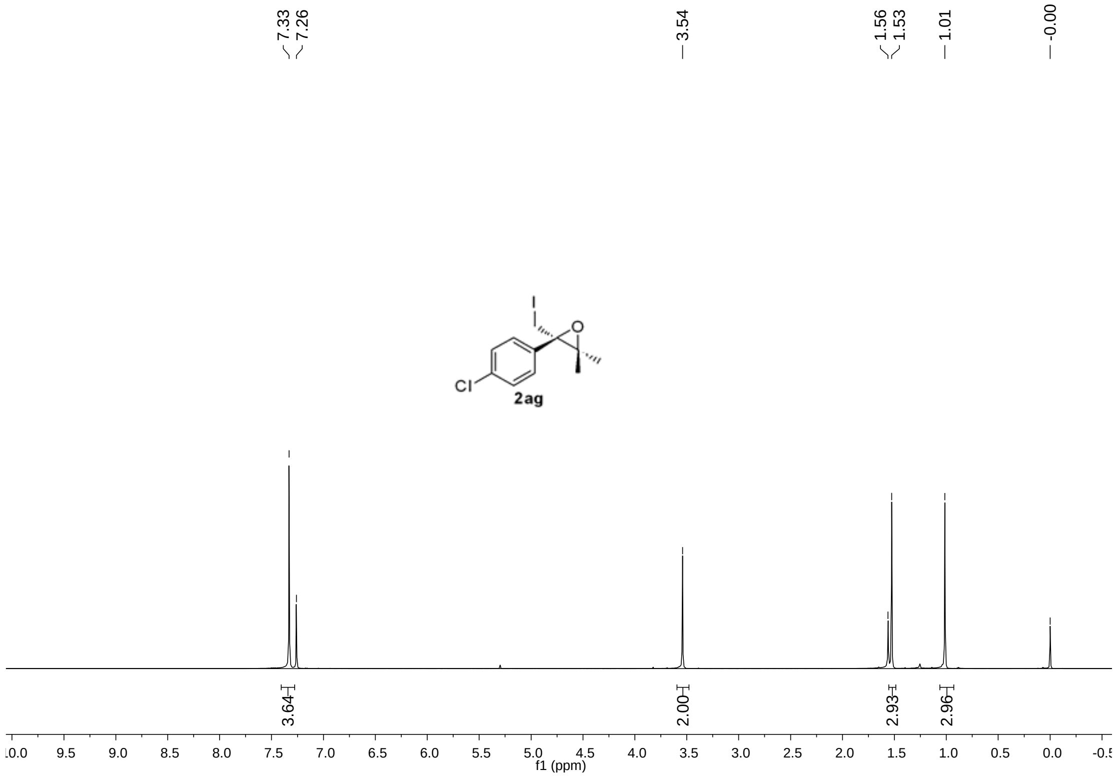
7.33
7.26
3.54
1.56
1.53
1.01
-0.00
Cl
I
O
2ag
3.64
2.00
2.93
2.96
f1 (ppm)

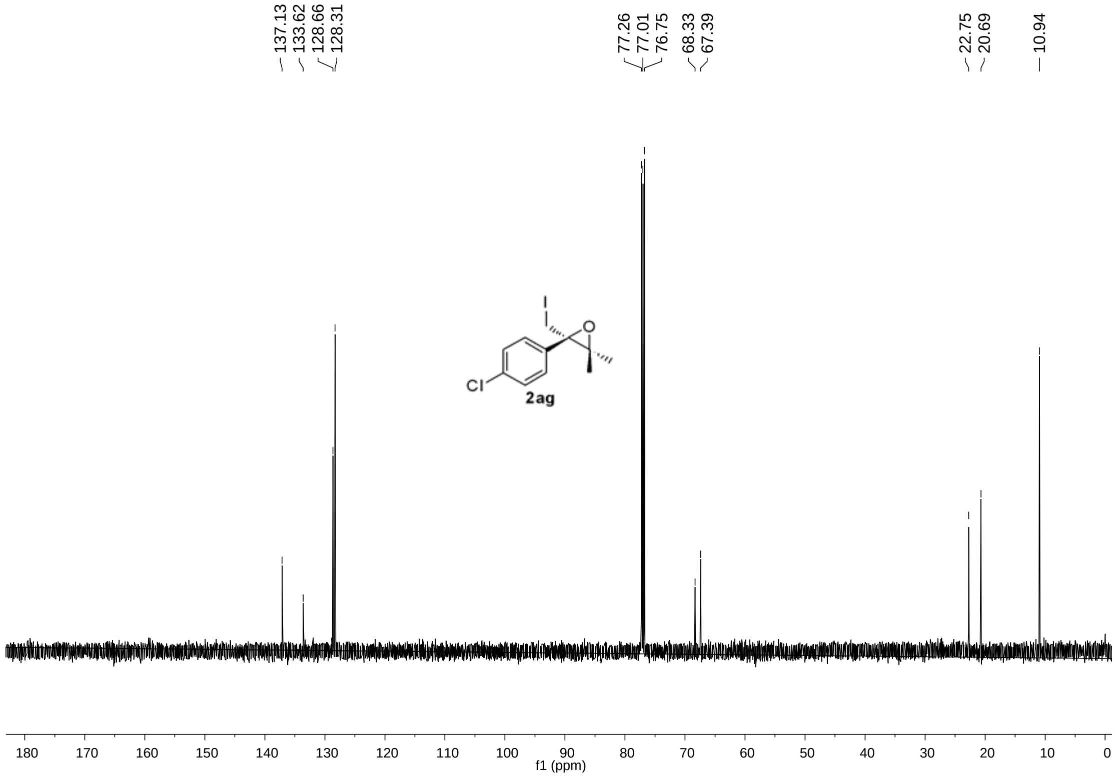
137.13
133.62
128.66
128.31
77.26
77.01
76.75
68.33
67.39
22.75
20.69
10.94
Cl
I
O
2ag
f1 (ppm)

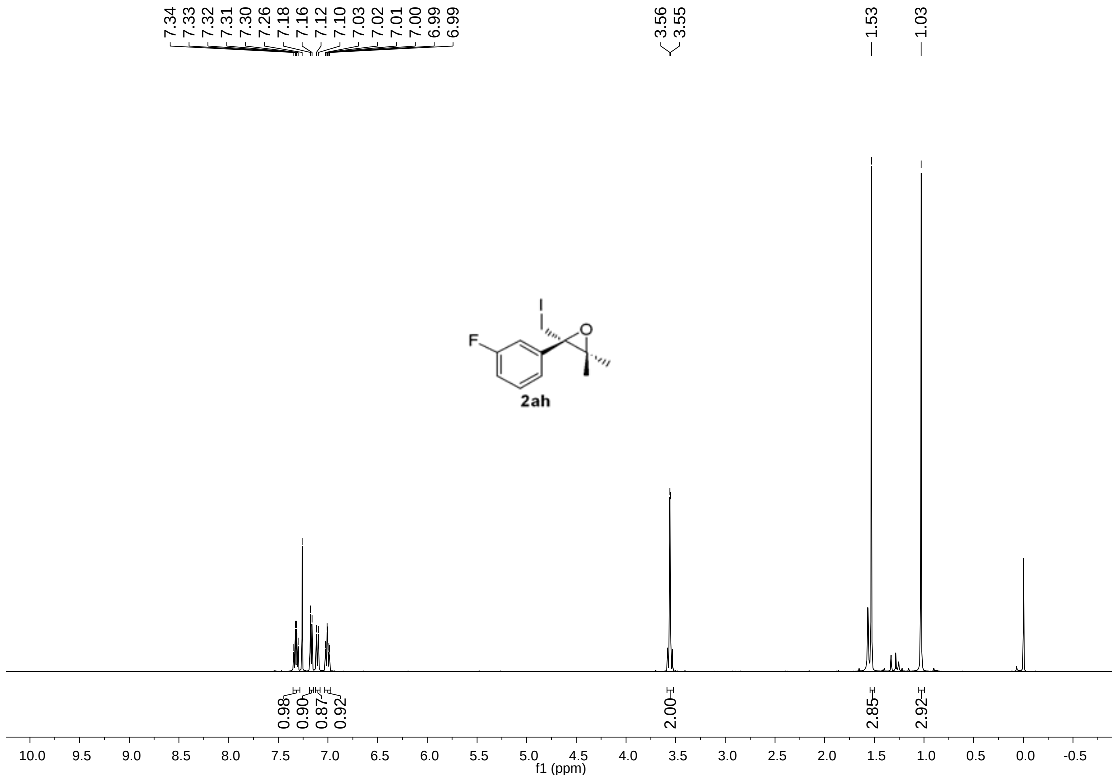
7.34 7.33 7.32 7.31 7.30 7.26 7.18 7.16 7.12 7.10 7.03 7.02 7.01 7.00 6.99 6.99
3.56 3.55
1.53
1.03
F
I
O
2ah
0.98 0.90 0.87 0.92
2.00
2.85
2.92
10.0 9.5 9.0 8.5 8.0 7.5 7.0 6.5 6.0 5.5 5.0 4.5 4.0 3.5 3.0 2.5 2.0 1.5 1.0 0.5 0.0 -0.5
f1 (ppm)

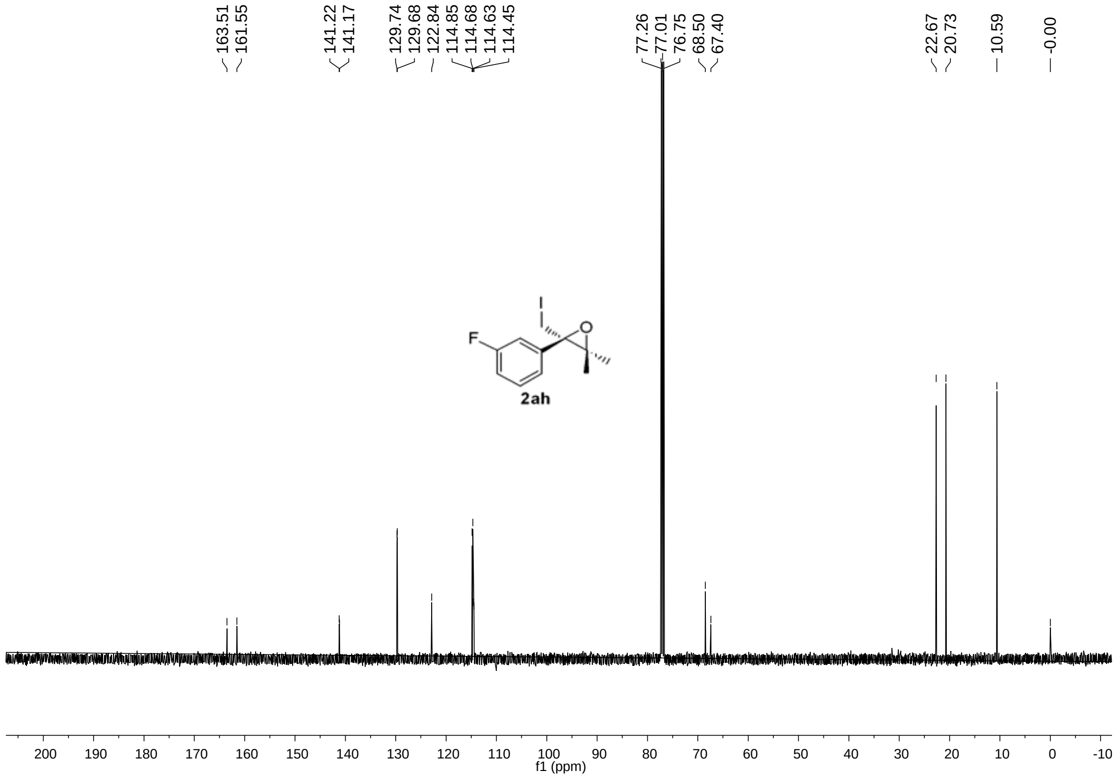
163.51 161.55
141.22 141.17
129.74 129.68 122.84 114.85 114.68 114.63 114.45
77.26 77.01 76.75 68.50 67.40
22.67 20.73
10.59
-0.00
F
I
O
2ah
200 190 180 170 160 150 140 130 120 110 100 90 80 70 60 50 40 30 20 10 0 -10
f1 (ppm)

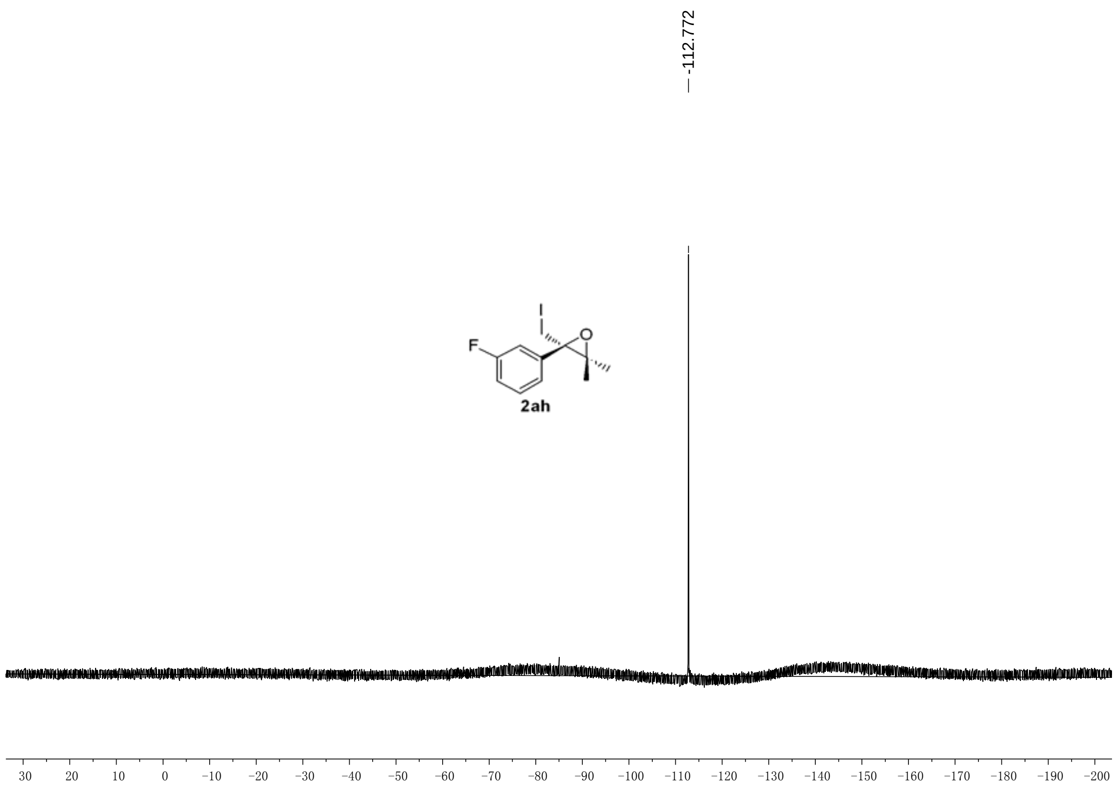
-112.772
F
I
O
2ah

j

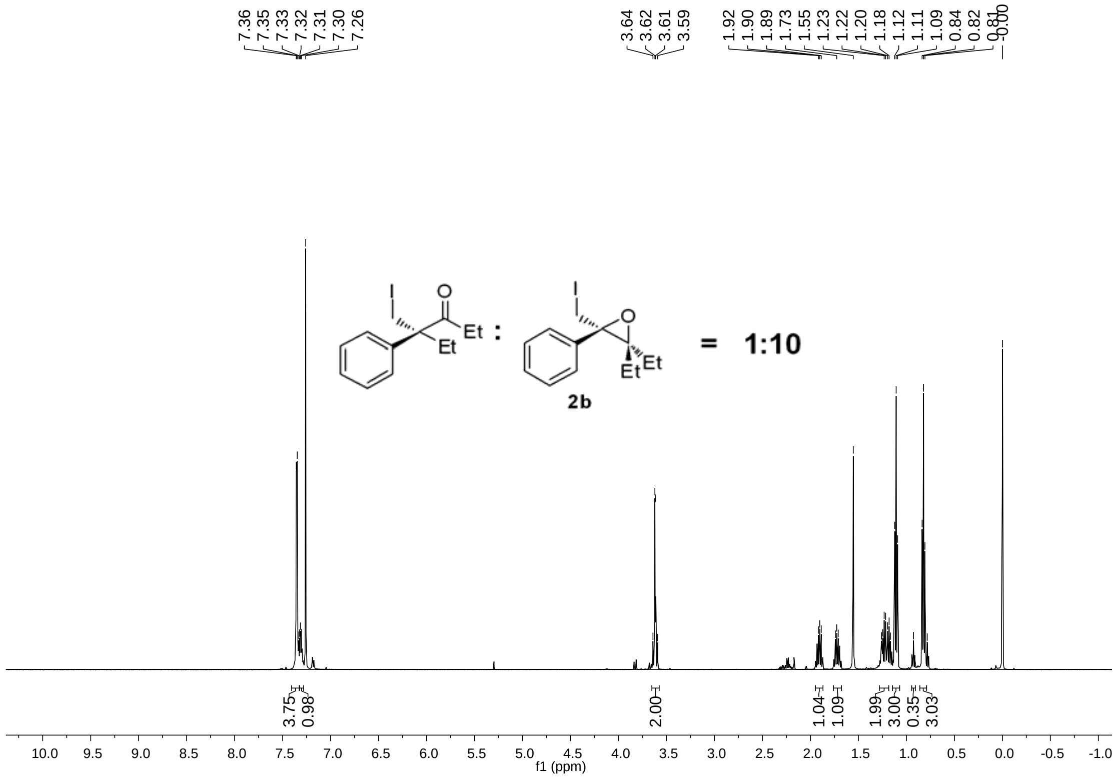
7.36
7.35
7.33
7.32
7.31
7.30
7.26
3.64
3.62
3.61
3.59
1.92
1.90
1.89
1.73
1.55
1.23
1.22
1.20
1.18
1.12
1.11
1.09
0.84
0.82
0.81
-0.00
Et
Et
Et
Et
2b
= 1:10
3.75
0.98
2.00
1.04
1.09
1.99
3.00
0.35
3.03
f1 (ppm)

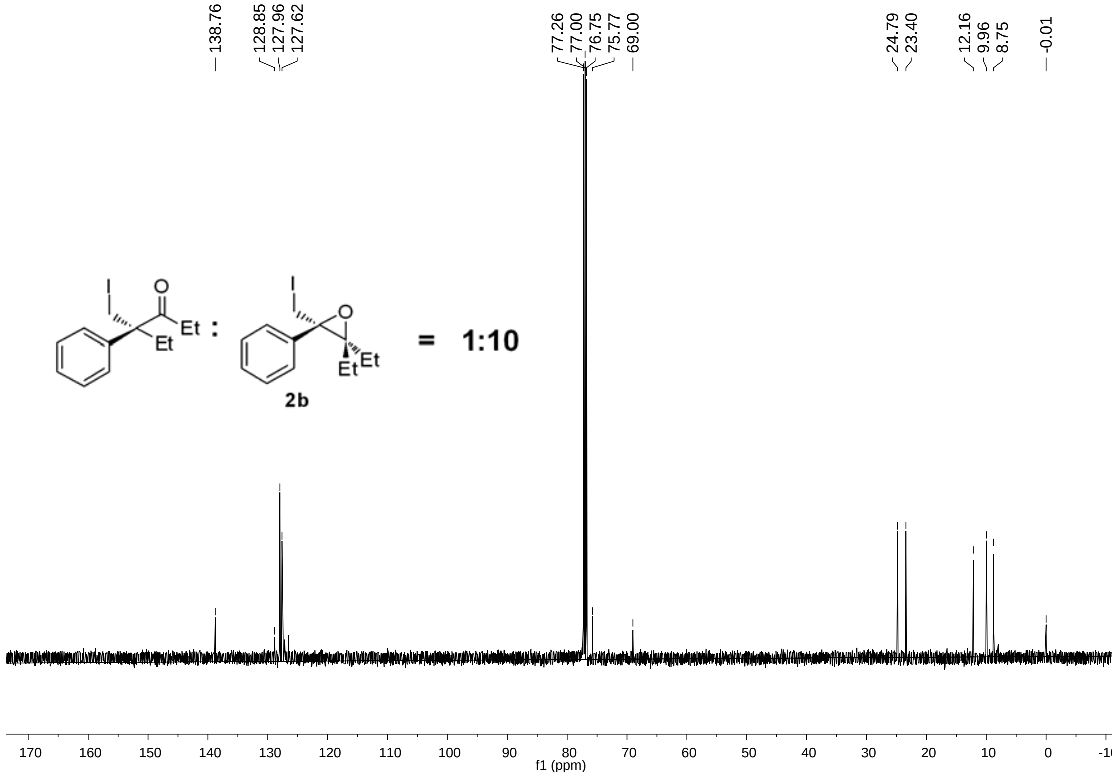
138.76
128.85
127.96
127.62
77.26
77.00
76.75
75.77
69.00
24.79
23.40
12.16
9.96
8.75
-0.01
Et
Et
Et
Et
= 1:10
2b
170 160 150 140 130 120 110 100 90 80 70 60 50 40 30 20 10 0
f1 (ppm)

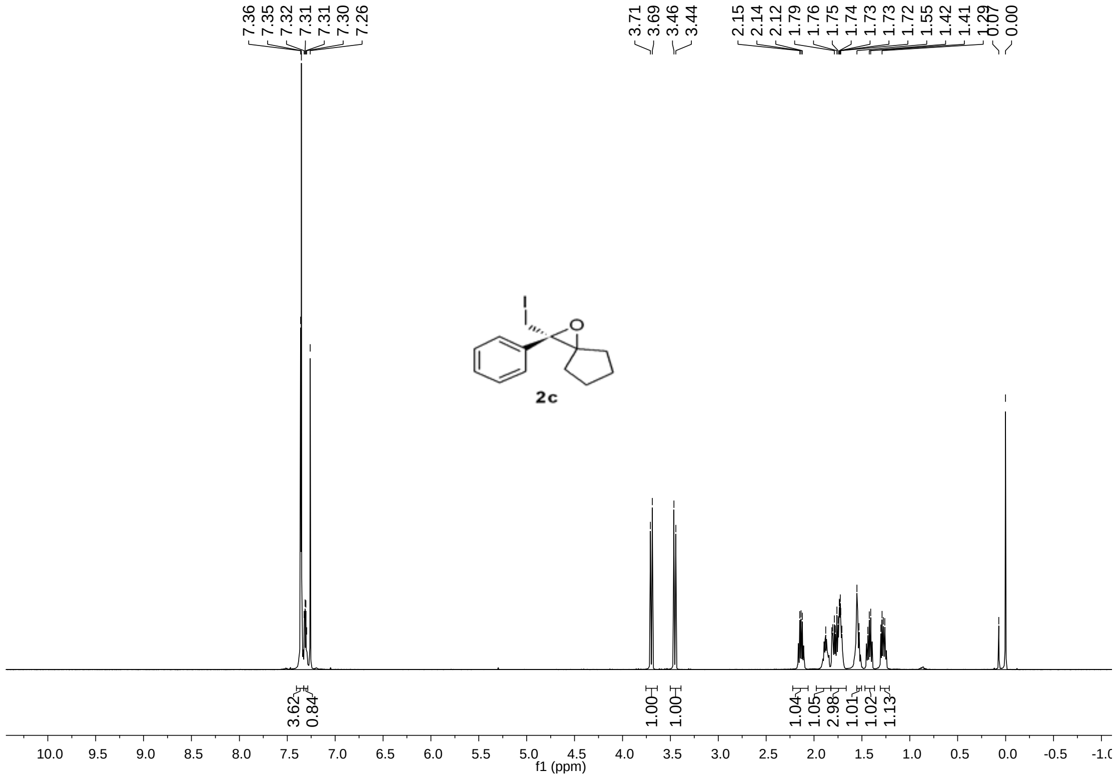
7.36
7.35
7.32
7.31
7.31
7.30
7.26
3.71
3.69
3.46
3.44
2.15
2.14
2.12
1.79
1.76
1.75
1.74
1.73
1.73
1.72
1.55
1.42
1.41
1.29
0.07
0.00
2c
3.62
0.84
1.00
1.00
1.04
1.05
2.98
1.01
1.02
1.13
10.0 9.5 9.0 8.5 8.0 7.5 7.0 6.5 6.0 5.5 5.0 4.5 4.0 3.5 3.0 2.5 2.0 1.5 1.0 0.5 0.0 -0.5
f1 (ppm)

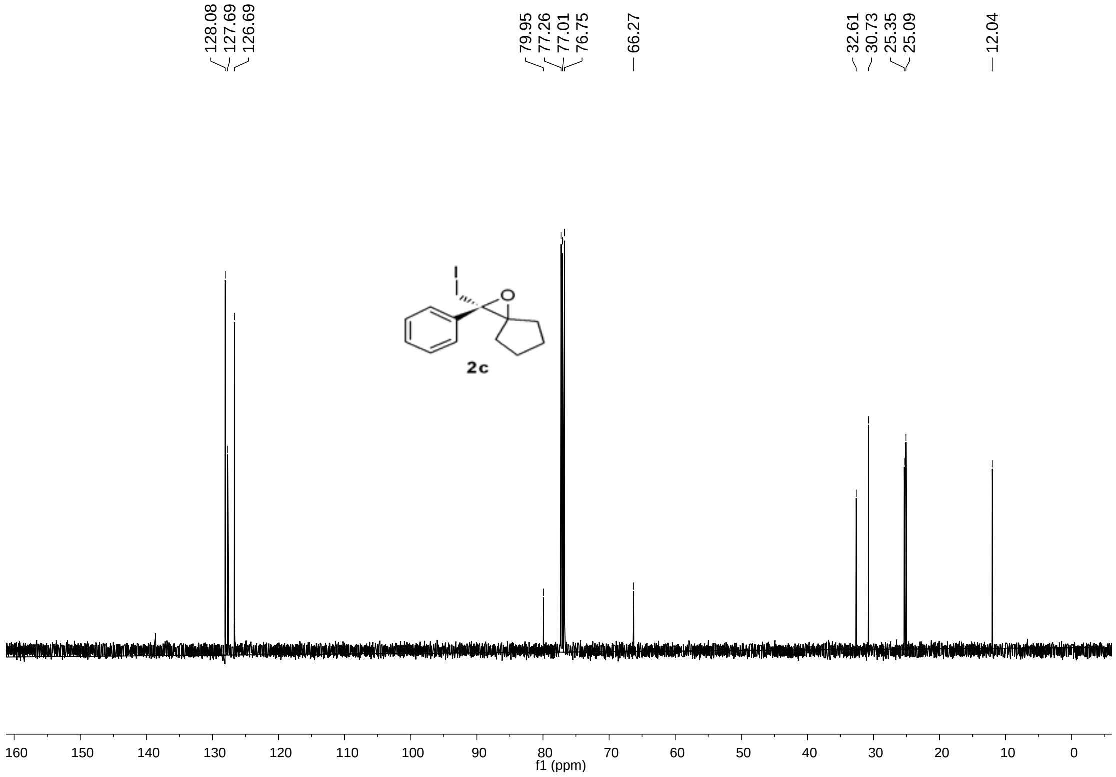
128.08
127.69
126.69
79.95
77.26
77.01
76.75
66.27
32.61
30.73
25.35
25.09
12.04
2c
f1 (ppm)

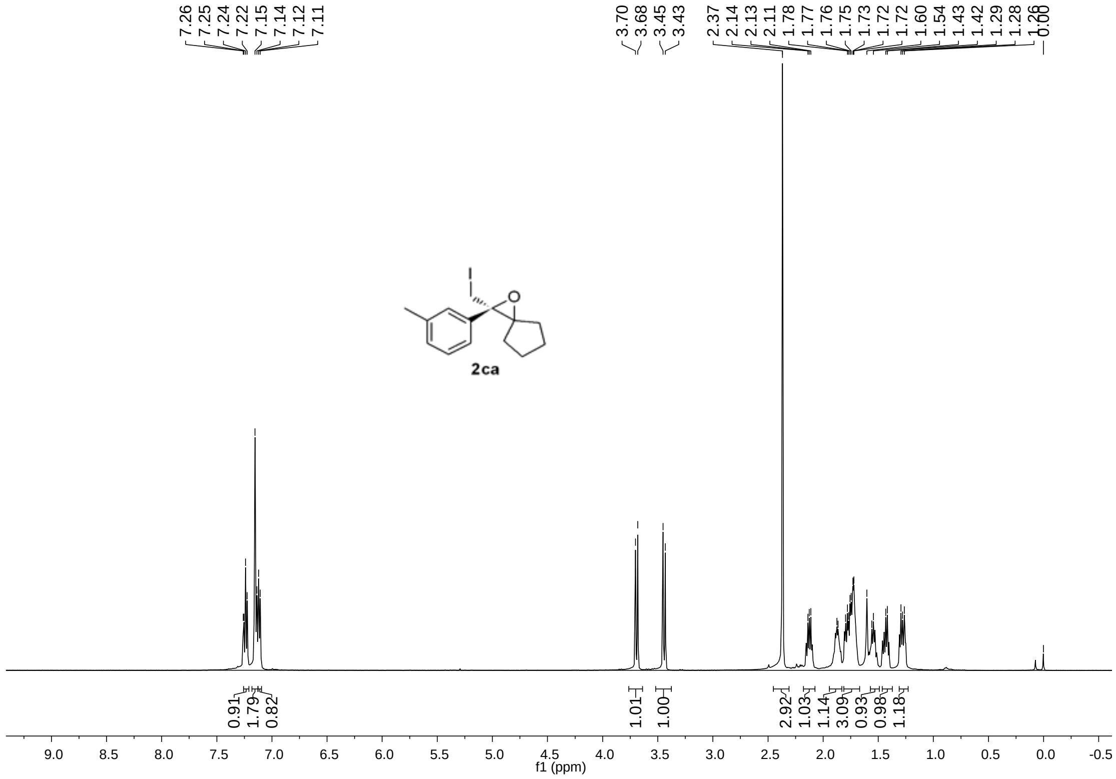
7.26
7.25
7.24
7.22
7.15
7.14
7.12
7.11
3.70
3.68
3.45
3.43
2.37
2.14
2.13
2.11
1.78
1.77
1.76
1.75
1.73
1.72
1.72
1.60
1.54
1.43
1.42
1.29
1.28
1.26
0.00
2ca
0.91
1.79
0.82
1.01
1.00
2.92
1.03
1.14
3.09
0.93
0.98
1.18
f1 (ppm)

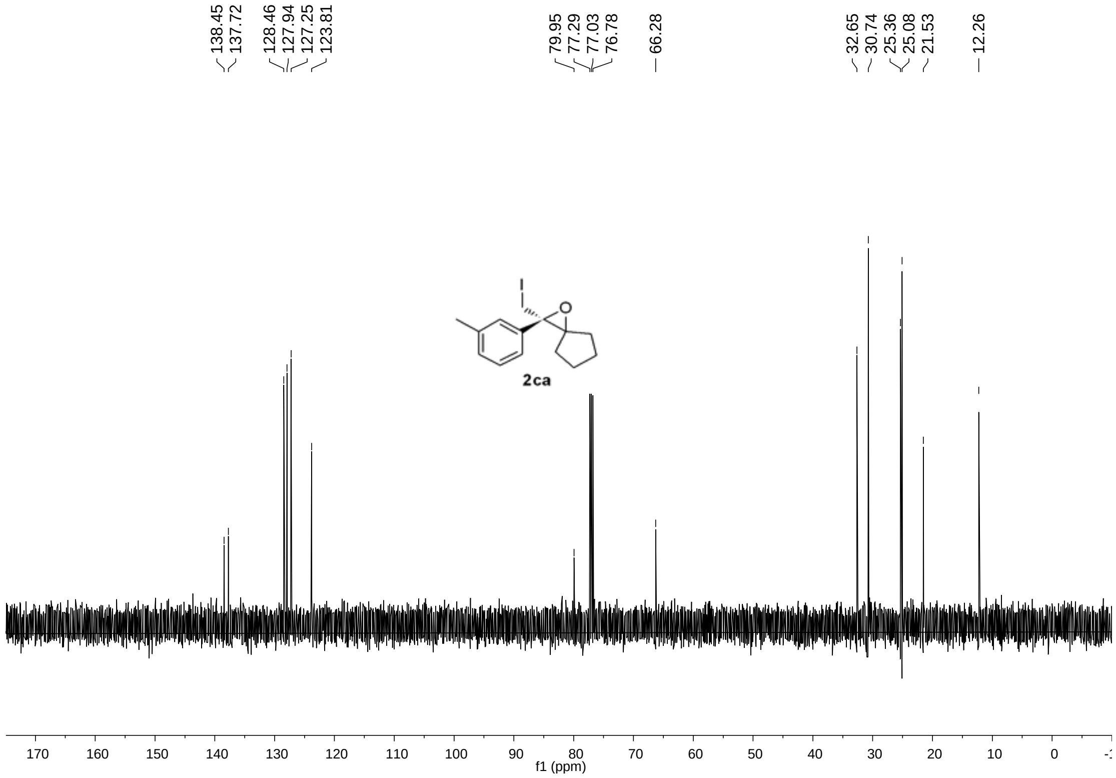
138.45
137.72
128.46
127.94
127.25
123.81
79.95
77.29
77.03
76.78
66.28
32.65
30.74
25.36
25.08
21.53
12.26
2ca
f1 (ppm)

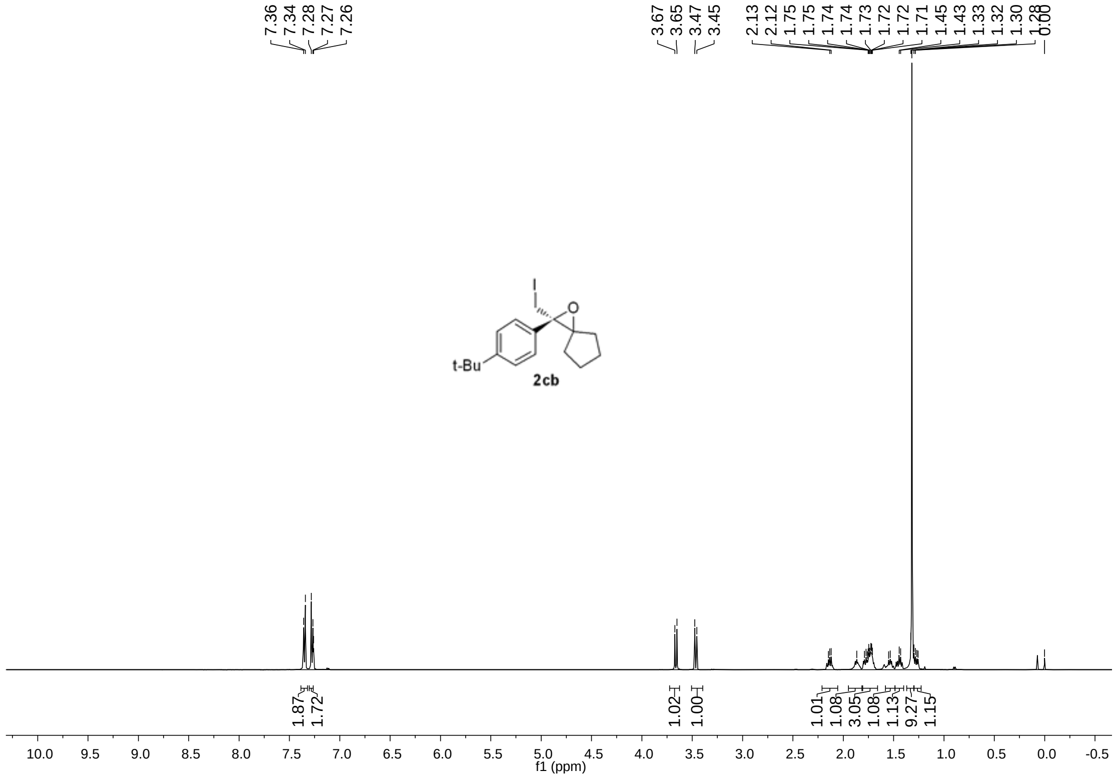
7.36
7.34
7.28
7.27
7.26
3.67
3.65
3.47
3.45
2.13
2.12
1.75
1.75
1.74
1.74
1.73
1.72
1.72
1.71
1.45
1.43
1.33
1.32
1.30
1.28
0.00
t-Bu
2cb
1.87
1.72
1.02
1.00
1.01
1.08
3.05
1.08
1.13
9.27
1.15
f1 (ppm)

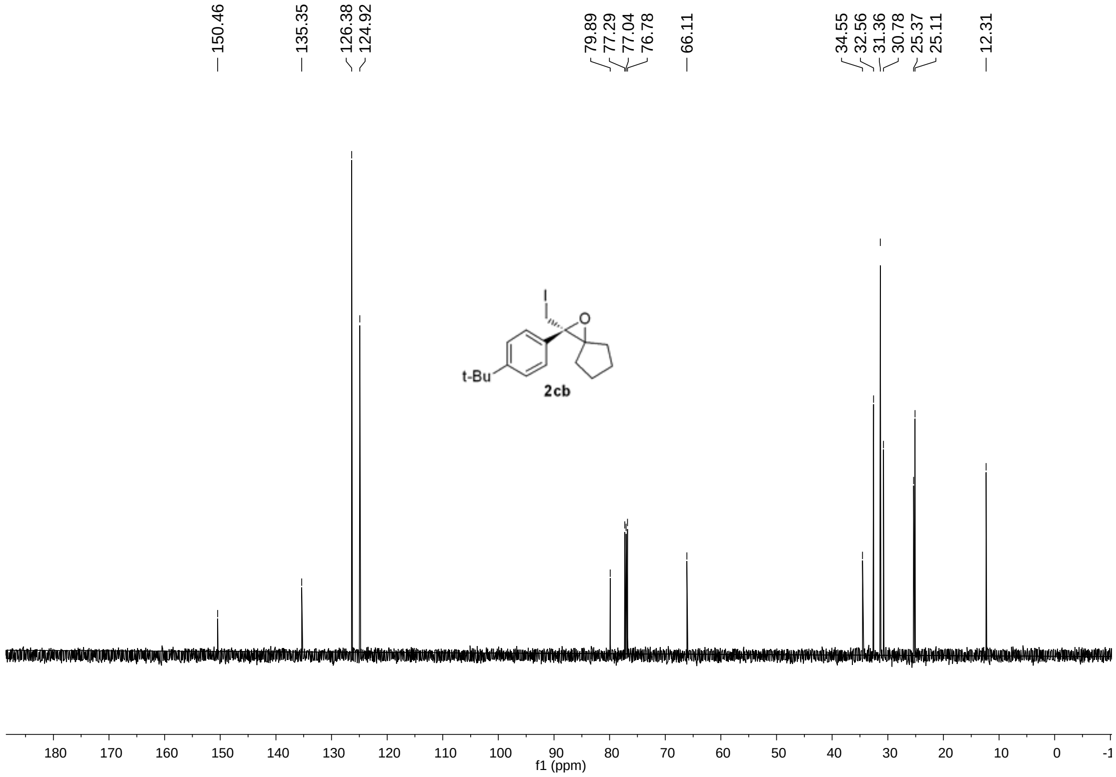
150.46
135.35
126.38
124.92
79.89
77.29
77.04
76.78
66.11
34.55
32.56
31.36
30.78
25.37
25.11
12.31
t-Bu
I
O
2cb
f1 (ppm)

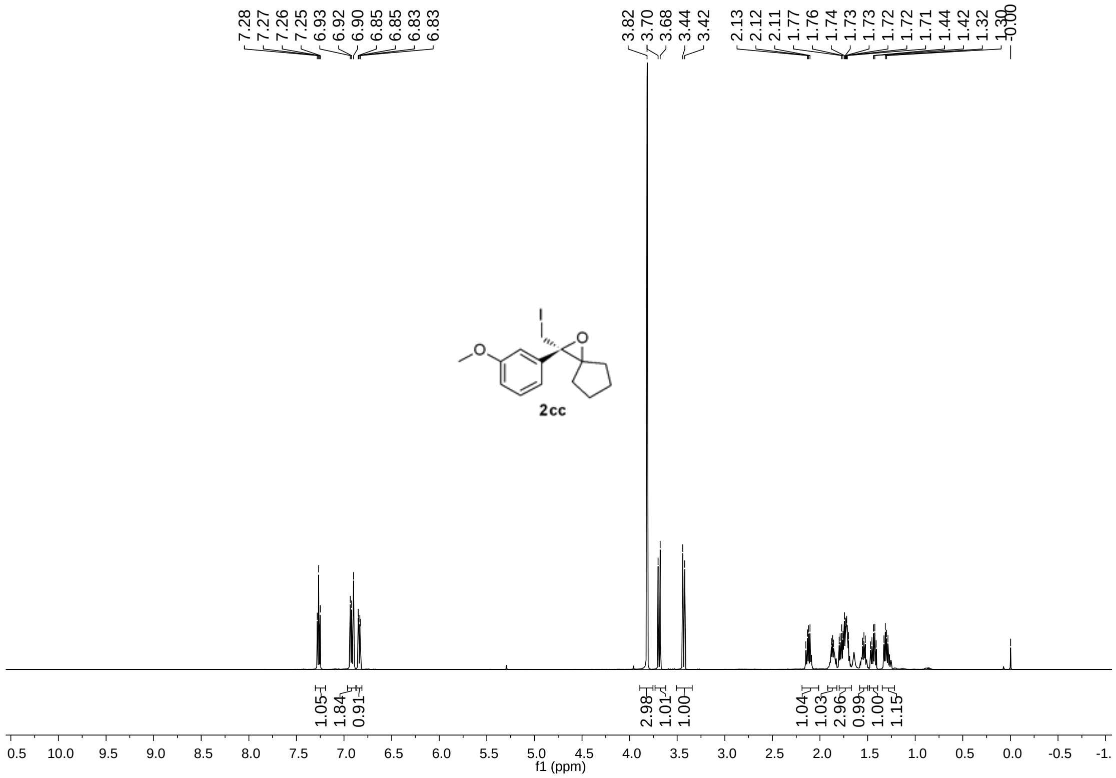
7.28
7.27
7.26
7.25
6.93
6.92
6.90
6.85
6.85
6.83
6.83
3.82
3.70
3.68
3.44
3.42
2.13
2.12
2.11
1.77
1.76
1.74
1.73
1.73
1.72
1.72
1.71
1.44
1.42
1.32
1.30
0.00
I
O
2cc
1.05
1.84
0.91
2.98
1.01
1.00
1.04
1.03
2.96
0.99
1.00
1.15
f1 (ppm)

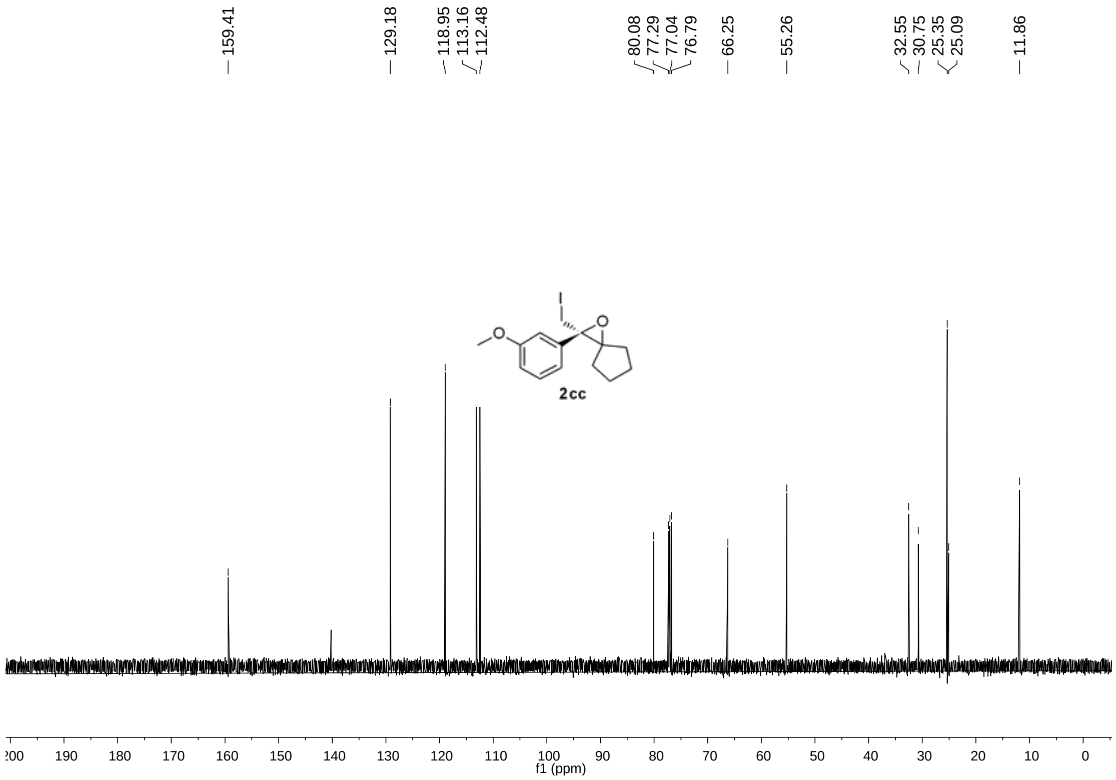
159.41
129.18
118.95
113.16
112.48
80.08
77.29
77.04
76.79
66.25
55.26
32.55
30.75
25.35
25.09
11.86
2cc
200 190 180 170 160 150 140 130 120 110 100 90 80 70 60 50 40 30 20 10 0
f1 (ppm)

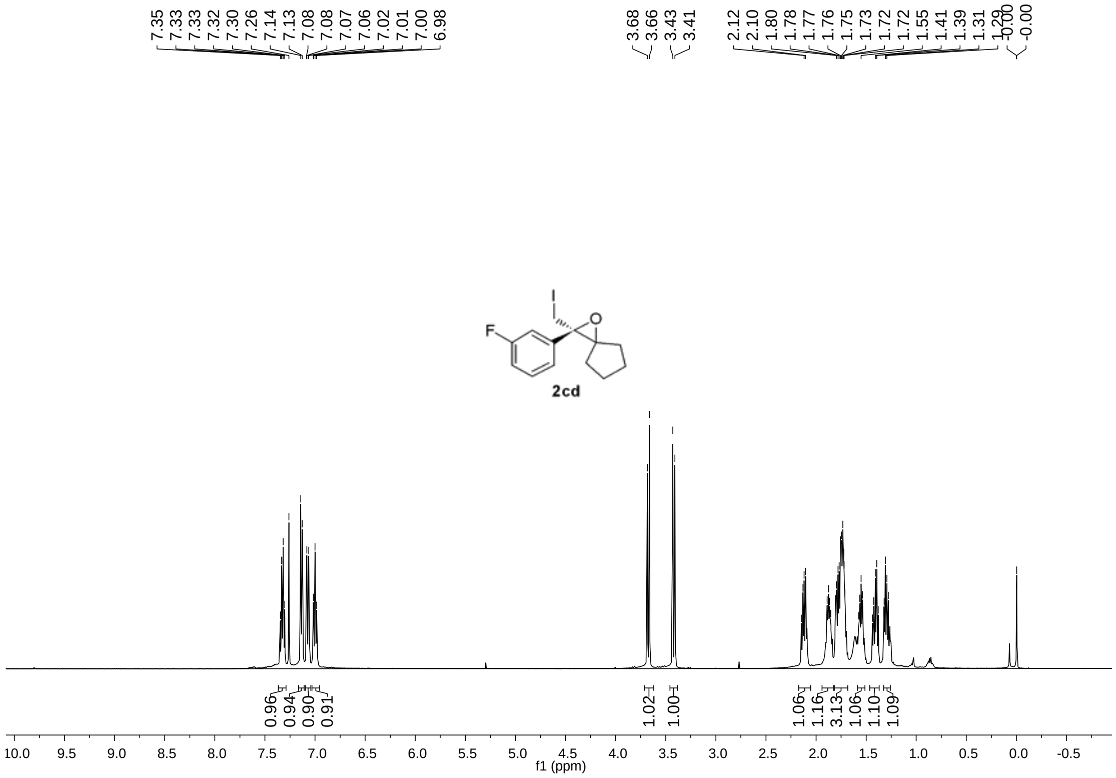
7.35
7.33
7.33
7.32
7.30
7.26
7.14
7.13
7.08
7.08
7.07
7.06
7.02
7.01
7.00
6.98
3.68
3.66
3.43
3.41
2.12
2.10
1.80
1.78
1.77
1.76
1.75
1.73
1.72
1.72
1.55
1.41
1.39
1.31
1.29
0.00
-0.00
F
2cd
0.96
0.94
0.90
0.91
1.02
1.00
1.06
1.16
3.13
1.06
1.10
1.09
10.0 9.5 9.0 8.5 8.0 7.5 7.0 6.5 6.0 5.5 5.0 4.5 4.0 3.5 3.0 2.5 2.0 1.5 1.0 0.5 0.0 -0.5
f1 (ppm)

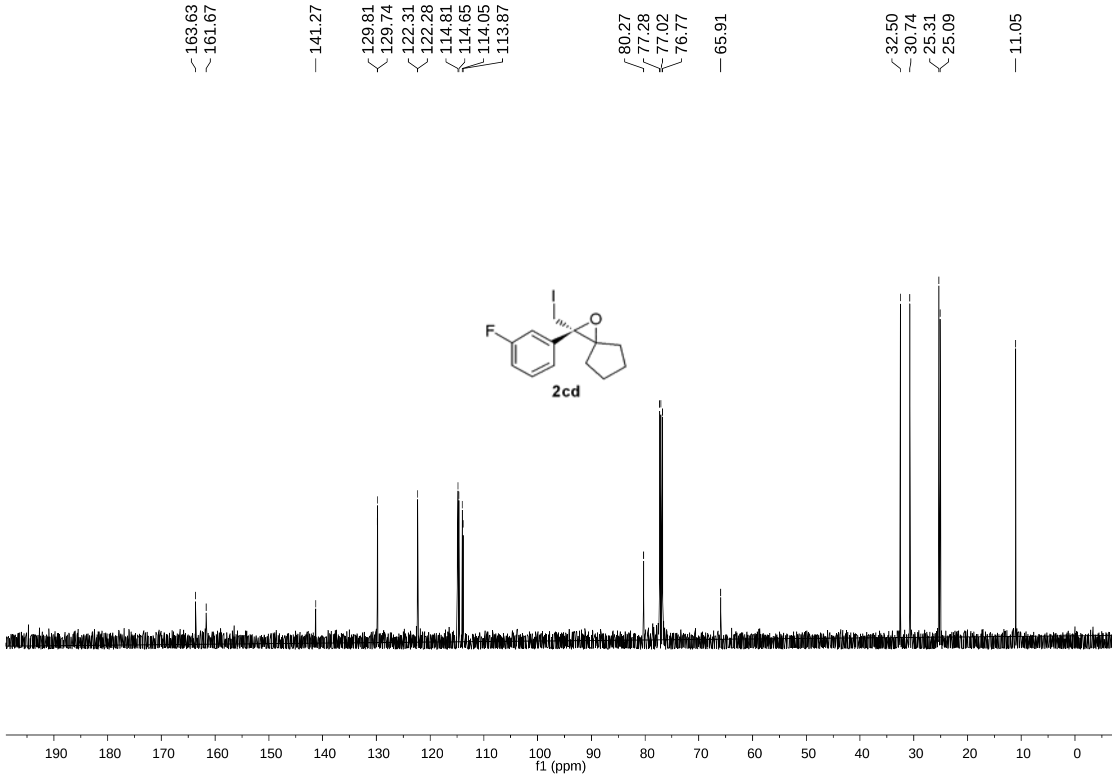
163.63
161.67
141.27
129.81
129.74
122.31
122.28
114.81
114.65
114.05
113.87
80.27
77.28
77.02
76.77
65.91
32.50
30.74
25.31
25.09
11.05
F
I
O
2cd
f1 (ppm)

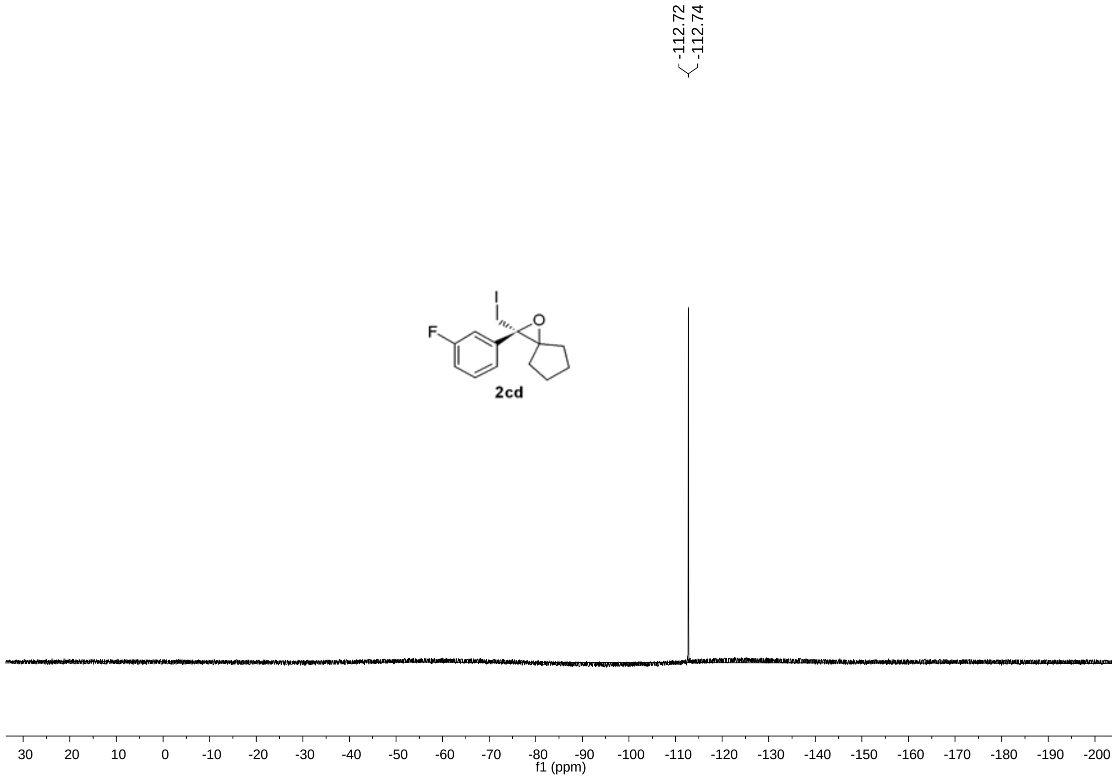
-112.72
-112.74
F
I
O
2cd
f1 (ppm)

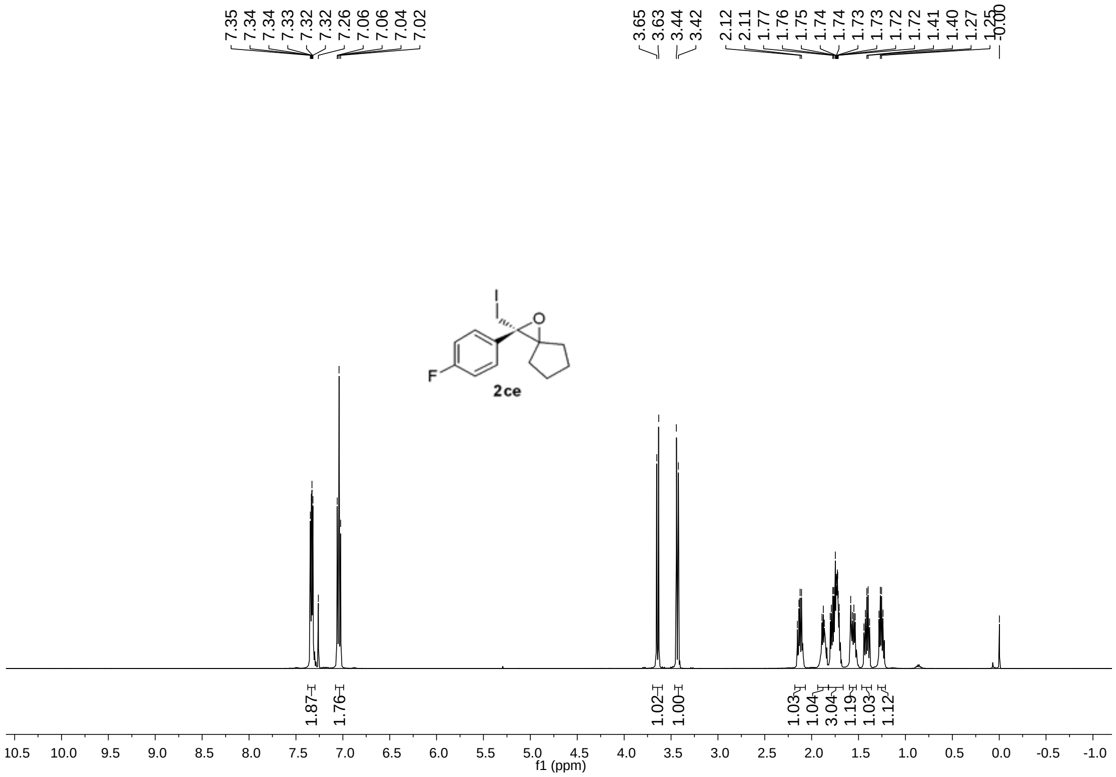
7.35 7.34 7.34 7.33 7.32 7.32 7.26 7.06 7.06 7.04 7.02
3.65 3.63 3.44 3.42
2.12 2.11 1.77 1.76 1.75 1.74 1.74 1.73 1.73 1.72 1.72 1.41 1.40 1.27 1.25 -0.00
I
O
F
2ce
1.87 1.76 1.02 1.00 1.03 1.04 3.04 1.19 1.03 1.12
10.5 10.0 9.5 9.0 8.5 8.0 7.5 7.0 6.5 6.0 5.5 5.0 4.5 4.0 3.5 3.0 2.5 2.0 1.5 1.0 0.5 0.0 -0.5 -1.0
f1 (ppm)

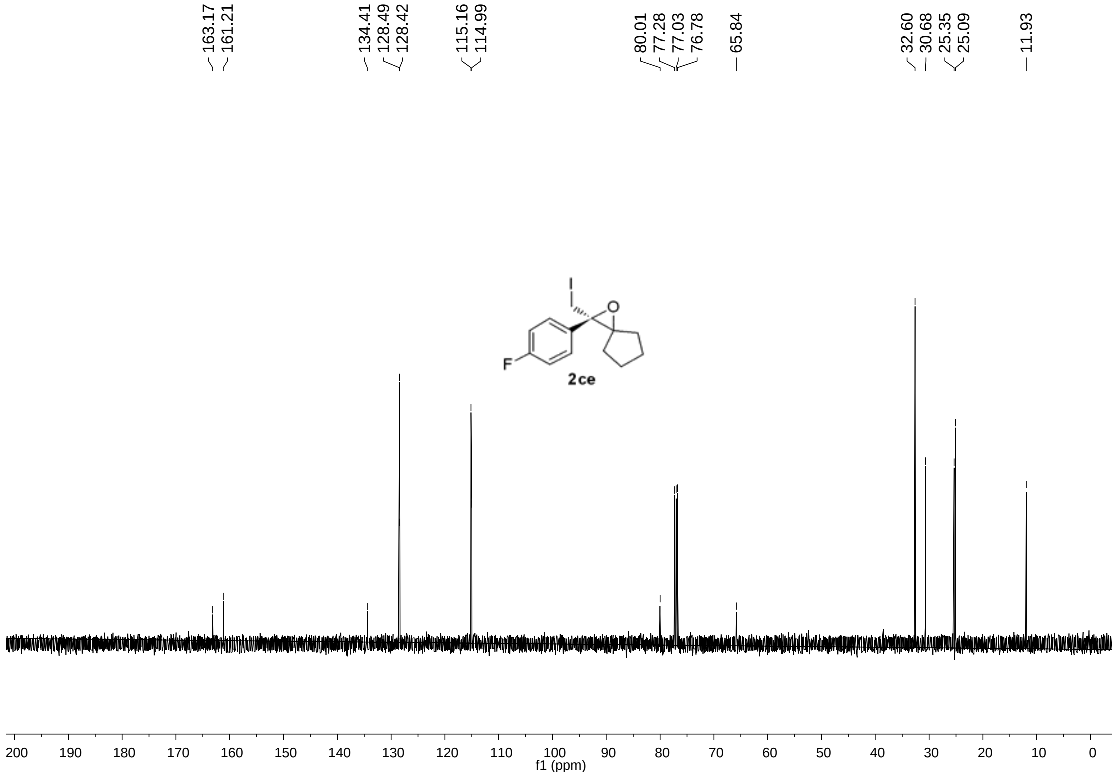
163.17 161.21
134.41 128.49 128.42
115.16 114.99
80.01 77.28 77.03 76.78
65.84
32.60 30.68 25.35 25.09
11.93
I
O
F
2ce
200 190 180 170 160 150 140 130 120 110 100 90 80 70 60 50 40 30 20 10 0
f1 (ppm)

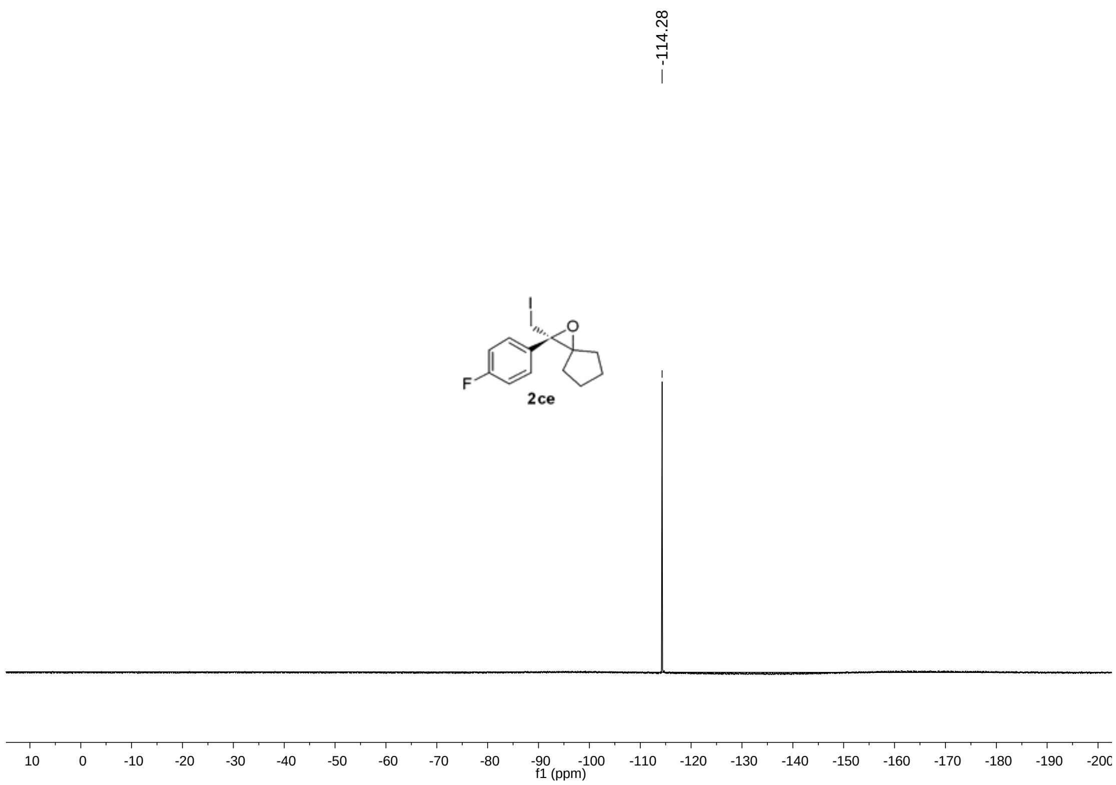
-114.28
F
2ce
10
0
-10
-20
-30
-40
-50
-60
-70
-80
-90
-100
-110
-120
-130
-140
-150
-160
-170
-180
-190
-200
f1 (ppm)

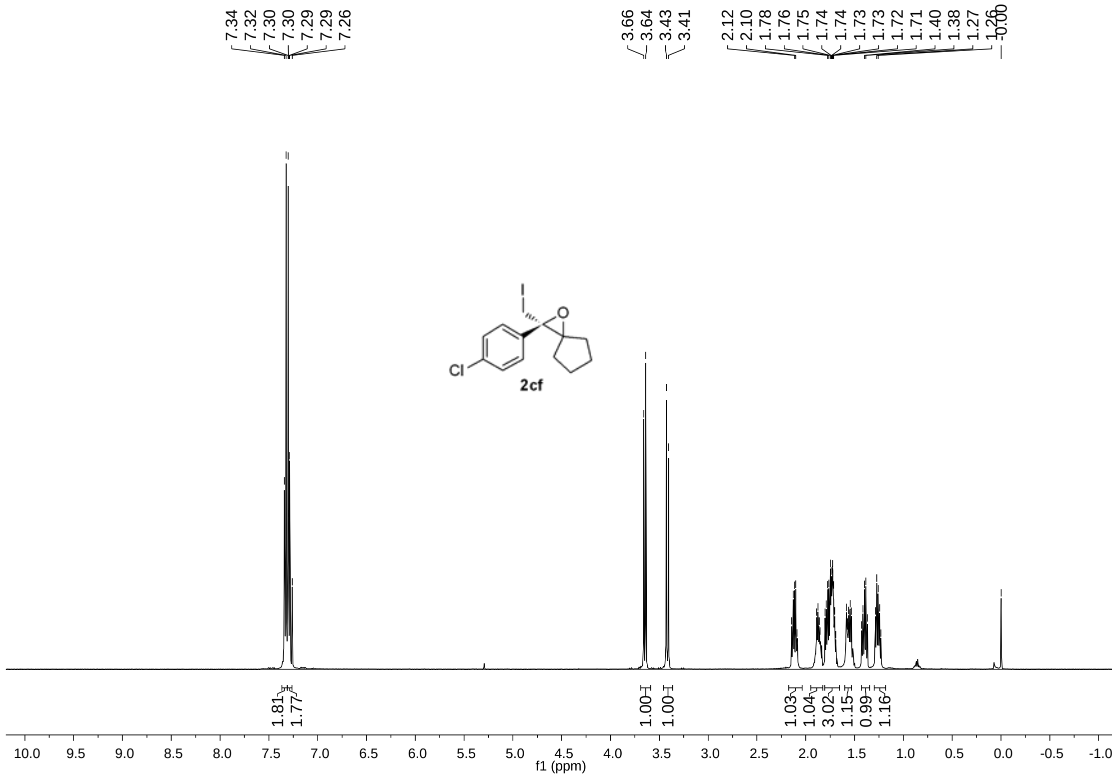
7.34
7.32
7.30
7.30
7.29
7.29
7.26
3.66
3.64
3.43
3.41
2.12
2.10
1.78
1.76
1.75
1.74
1.74
1.73
1.73
1.72
1.71
1.40
1.38
1.27
1.26
0.00
Cl
2cf
1.81
1.77
1.00
1.00
1.03
1.04
3.02
1.15
0.99
1.16
10.0
9.5
9.0
8.5
8.0
7.5
7.0
6.5
6.0
5.5
5.0
4.5
4.0
3.5
3.0
2.5
2.0
1.5
1.0
0.5
0.0
-0.5
-1.0
f1 (ppm)

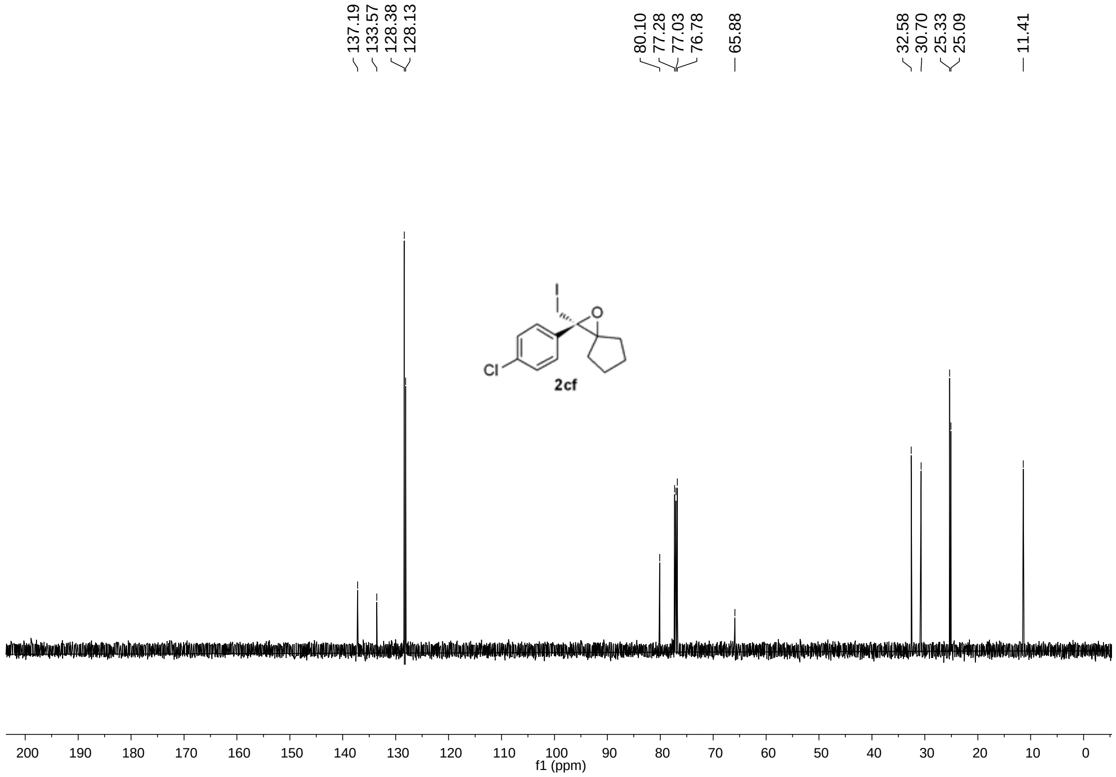
137.19
133.57
128.38
128.13
80.10
77.28
77.03
76.78
65.88
32.58
30.70
25.33
25.09
11.41
Cl
O
I
2cf
f1 (ppm)

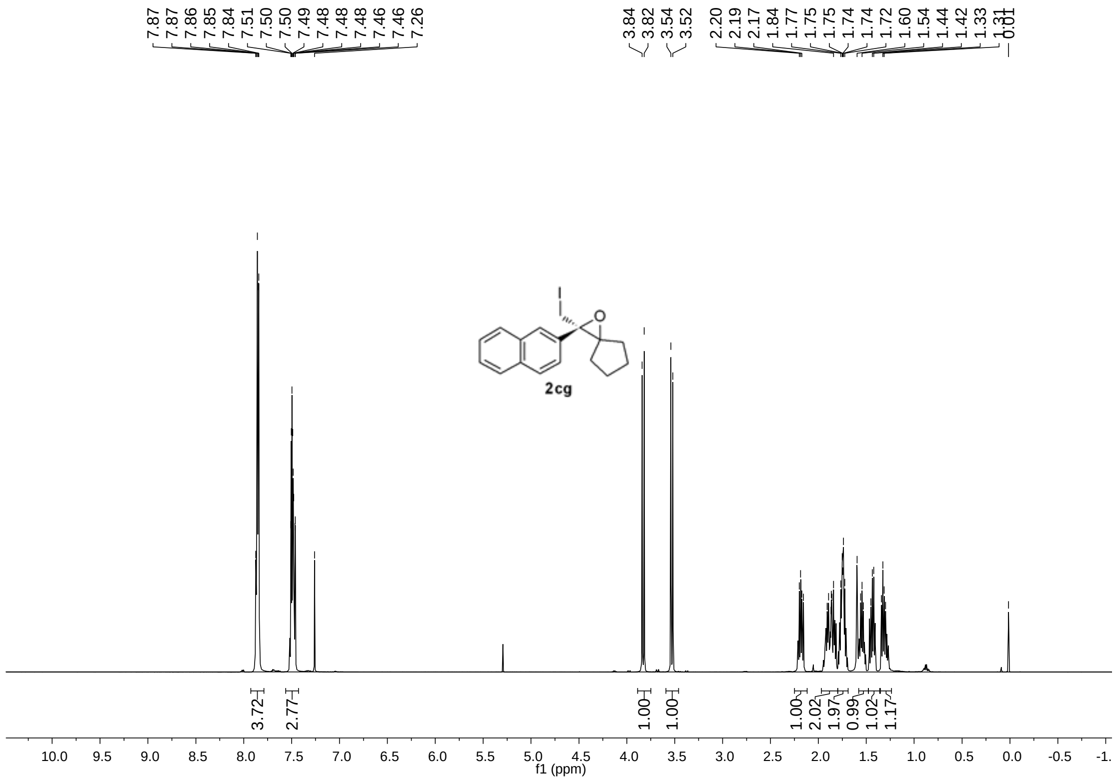
7.87
7.87
7.86
7.85
7.84
7.51
7.50
7.50
7.49
7.48
7.48
7.48
7.46
7.46
7.26
3.84
3.82
3.54
3.52
2.20
2.19
2.17
1.84
1.77
1.75
1.75
1.74
1.74
1.72
1.60
1.54
1.44
1.42
1.33
1.31
0.01
I
O
2cg
3.72
2.77
1.00
1.00
1.00
2.02
1.97
0.99
1.02
1.17
f1 (ppm)

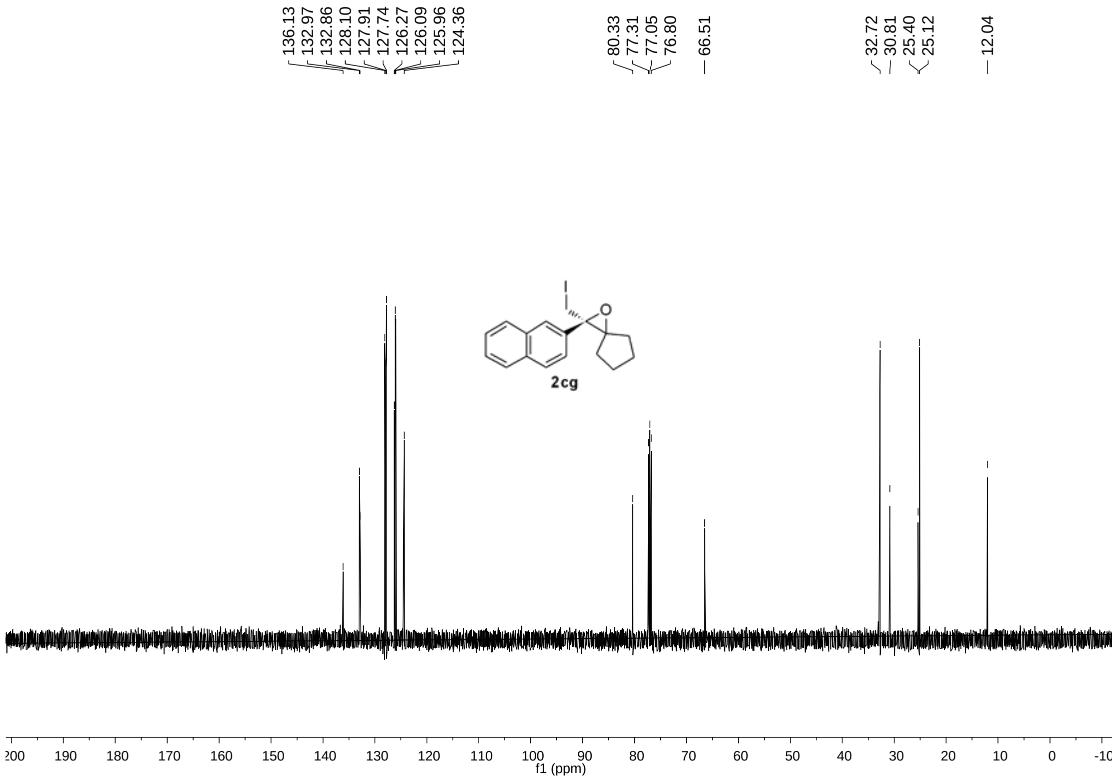
136.13
132.97
132.86
128.10
127.91
127.74
126.27
126.09
125.96
124.36
80.33
77.31
77.05
76.80
66.51
32.72
30.81
25.40
25.12
12.04
2cg
f1 (ppm)

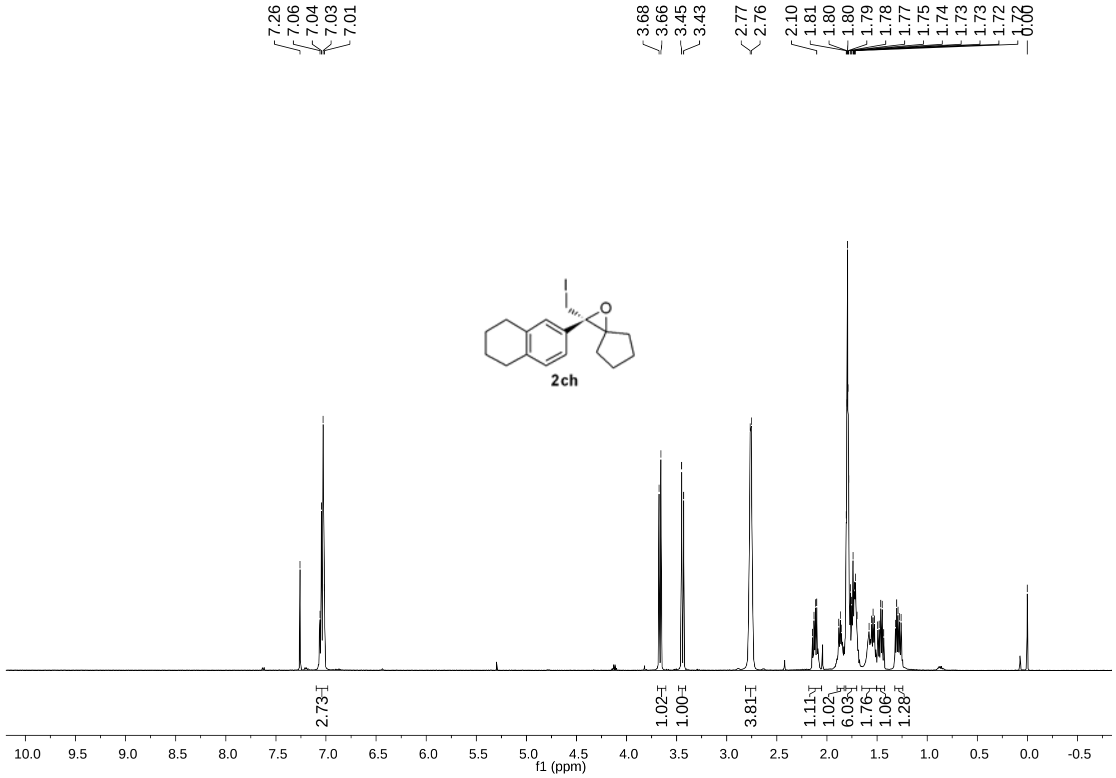
7.26
7.06
7.04
7.03
7.01
3.68
3.66
3.45
3.43
2.77
2.76
2.10
1.81
1.80
1.80
1.79
1.78
1.77
1.75
1.74
1.73
1.73
1.72
1.72
0.00
2ch
2.73
1.02
1.00
3.81
1.11
1.02
6.03
1.76
1.06
1.28
f1 (ppm)

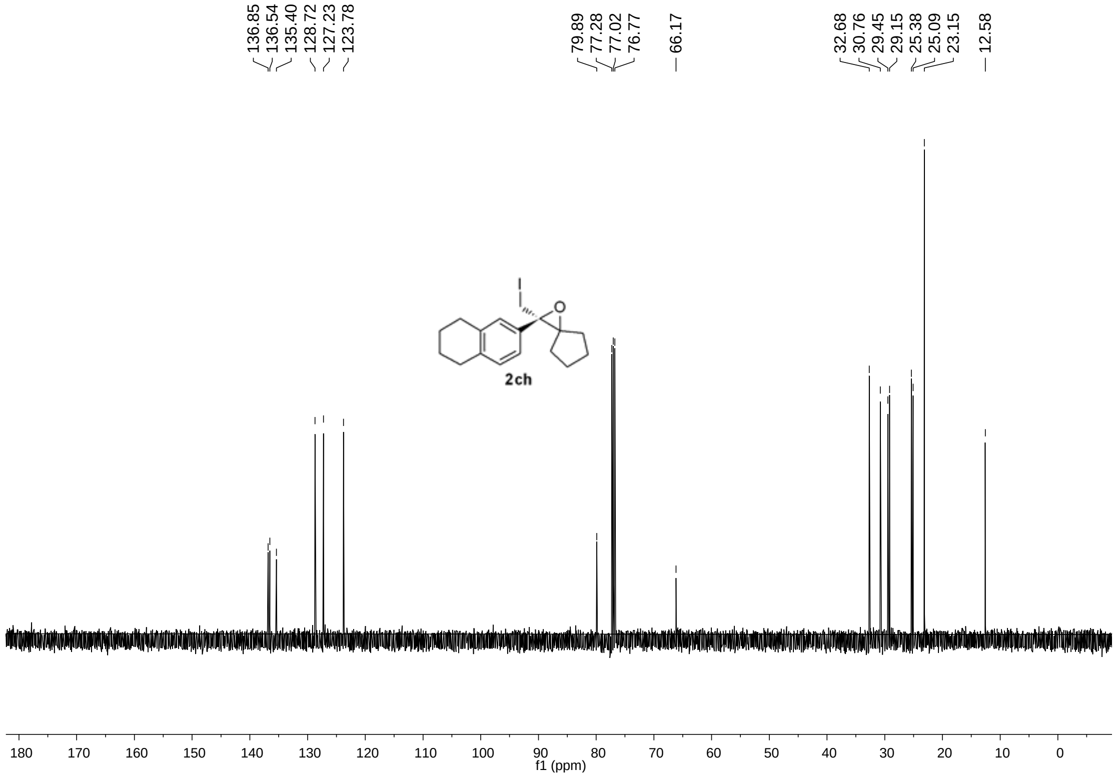
136.85
136.54
135.40
128.72
127.23
123.78
79.89
77.28
77.02
76.77
66.17
32.68
30.76
29.45
29.15
25.38
25.09
23.15
12.58
I
O
2ch
180 170 160 150 140 130 120 110 100 90 80 70 60 50 40 30 20 10 0
f1 (ppm)

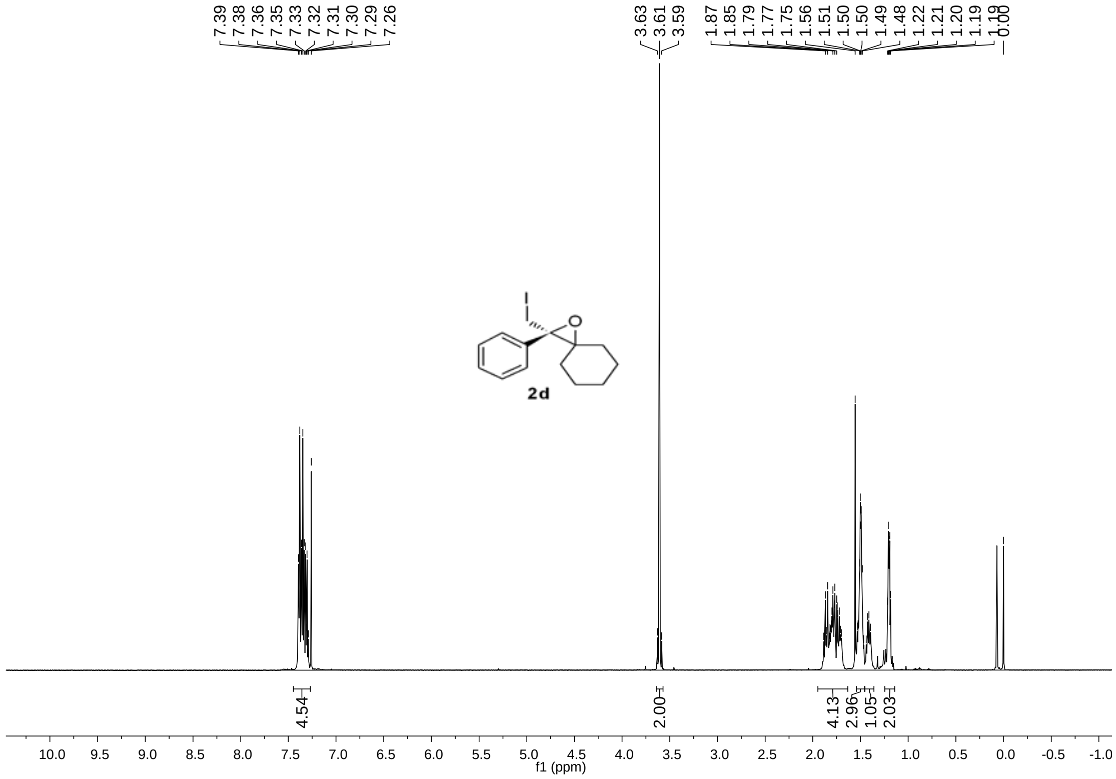
7.39
7.38
7.36
7.35
7.33
7.32
7.31
7.30
7.29
7.26
3.63
3.61
3.59
1.87
1.85
1.79
1.77
1.75
1.56
1.51
1.50
1.50
1.49
1.48
1.22
1.21
1.20
1.19
1.19
0.00
I
O
2d
4.54
2.00
4.13
2.96
1.05
2.03
10.0 9.5 9.0 8.5 8.0 7.5 7.0 6.5 6.0 5.5 5.0 4.5 4.0 3.5 3.0 2.5 2.0 1.5 1.0 0.5 0.0 -0.5 -1.0
f1 (ppm)

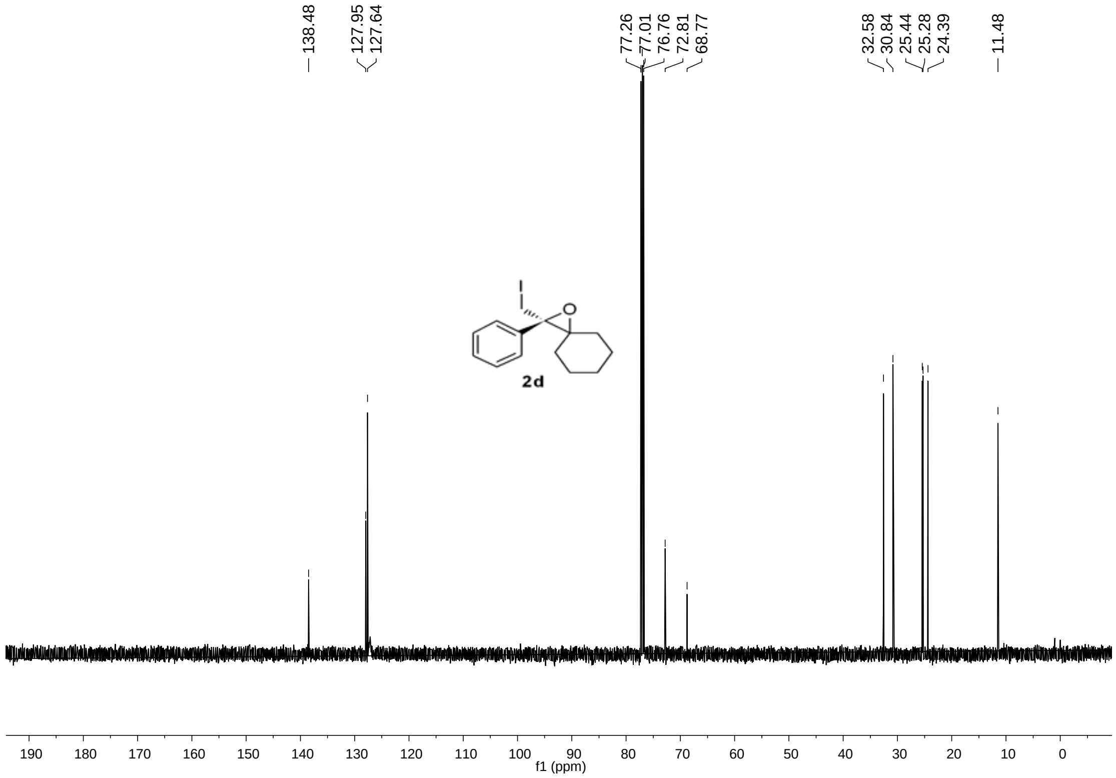
138.48
127.95
127.64
77.26
77.01
76.76
72.81
68.77
32.58
30.84
25.44
25.28
24.39
11.48
I
O
2d
f1 (ppm)

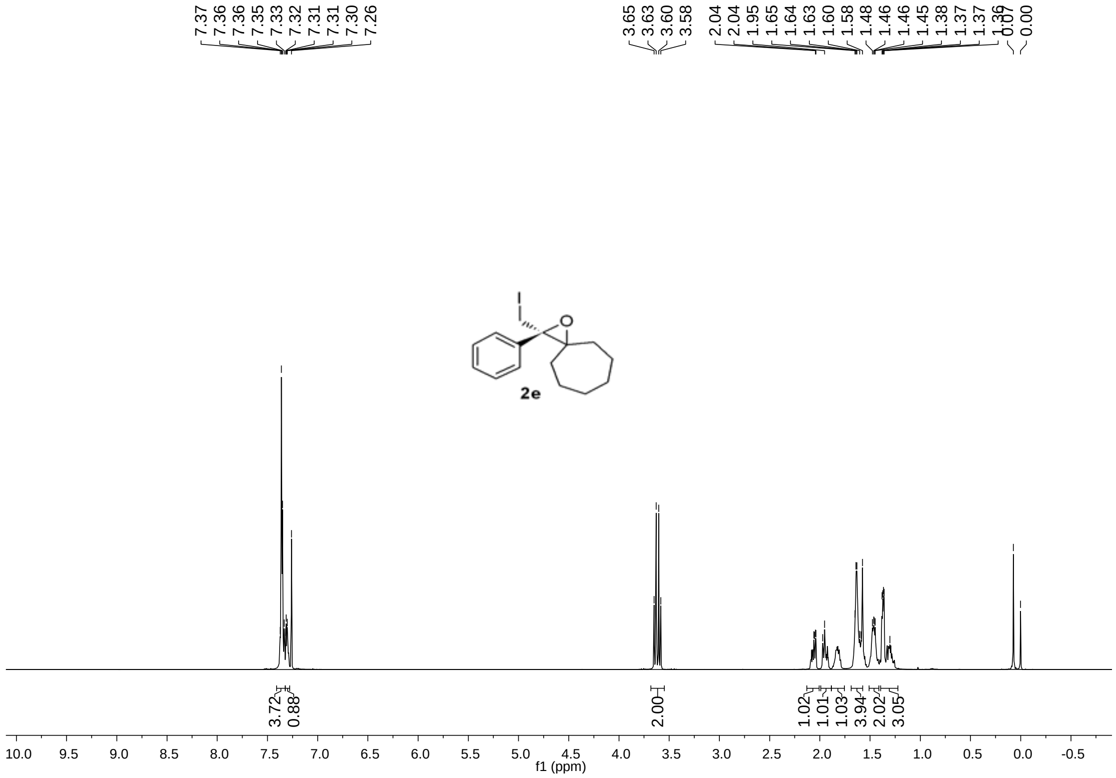
7.37
7.36
7.36
7.35
7.33
7.32
7.31
7.31
7.30
7.26
3.65
3.63
3.60
3.58
2.04
2.04
1.95
1.65
1.64
1.63
1.60
1.58
1.48
1.46
1.46
1.45
1.38
1.37
1.37
1.36
0.07
0.00
I
O
2e
3.72
0.88
2.00
1.02
1.01
1.03
3.94
2.02
3.05
f1 (ppm)

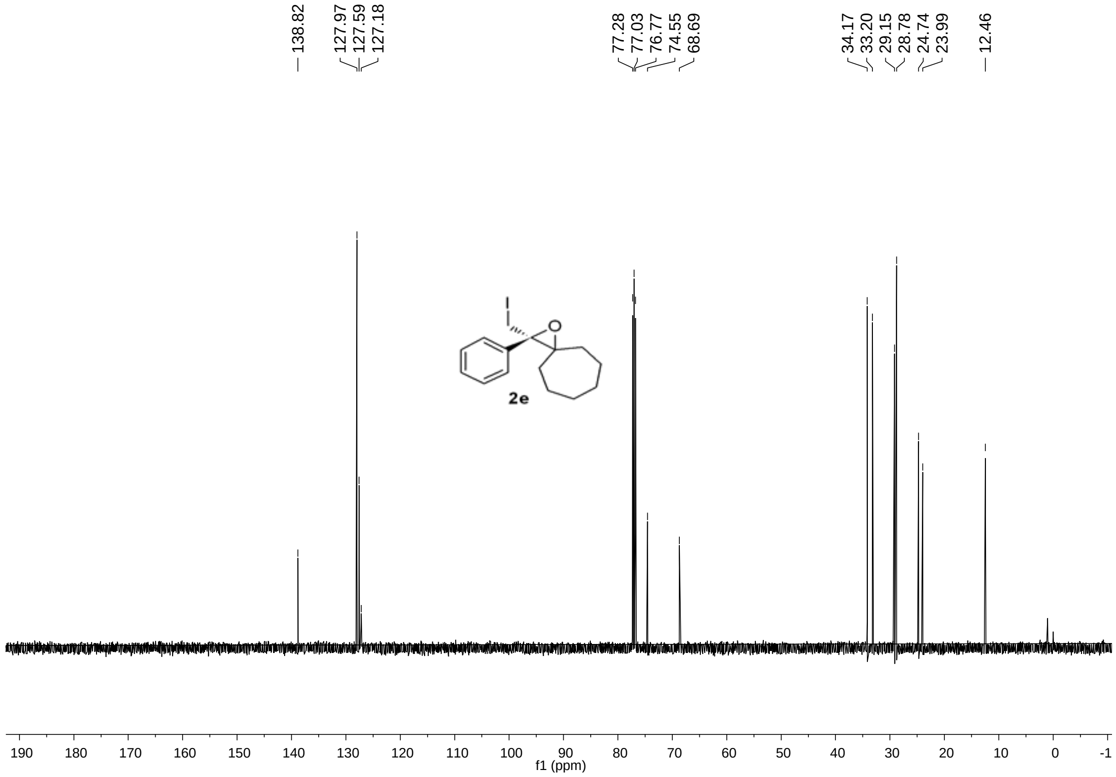
138.82
127.97
127.59
127.18
77.28
77.03
76.77
74.55
68.69
34.17
33.20
29.15
28.78
24.74
23.99
12.46
2e
f1 (ppm)

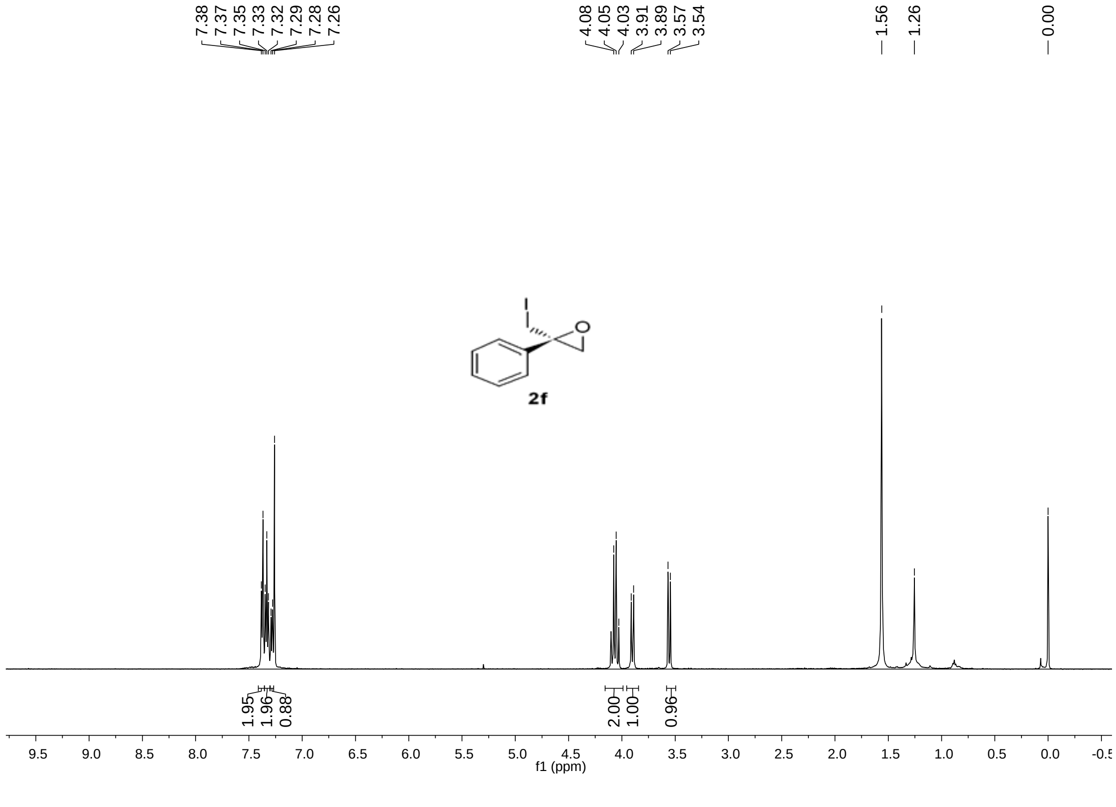
7.38
7.37
7.35
7.33
7.32
7.29
7.28
7.26
4.08
4.05
4.03
3.91
3.89
3.57
3.54
1.56
1.26
0.00
2f
1.95
1.96
0.88
2.00
1.00
0.96
f1 (ppm)

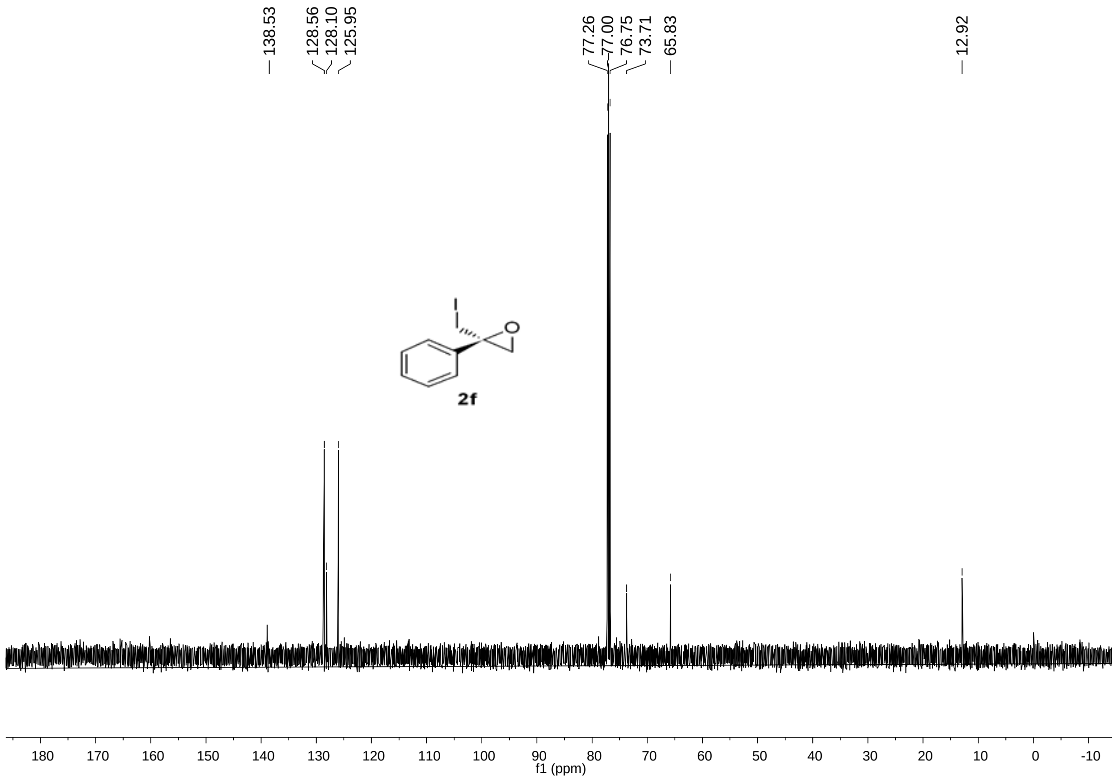
138.53
128.56
128.10
125.95
77.26
77.00
76.75
73.71
65.83
12.92
I
O
2f
180 170 160 150 140 130 120 110 100 90 80 70 60 50 40 30 20 10 0 -10
f1 (ppm)

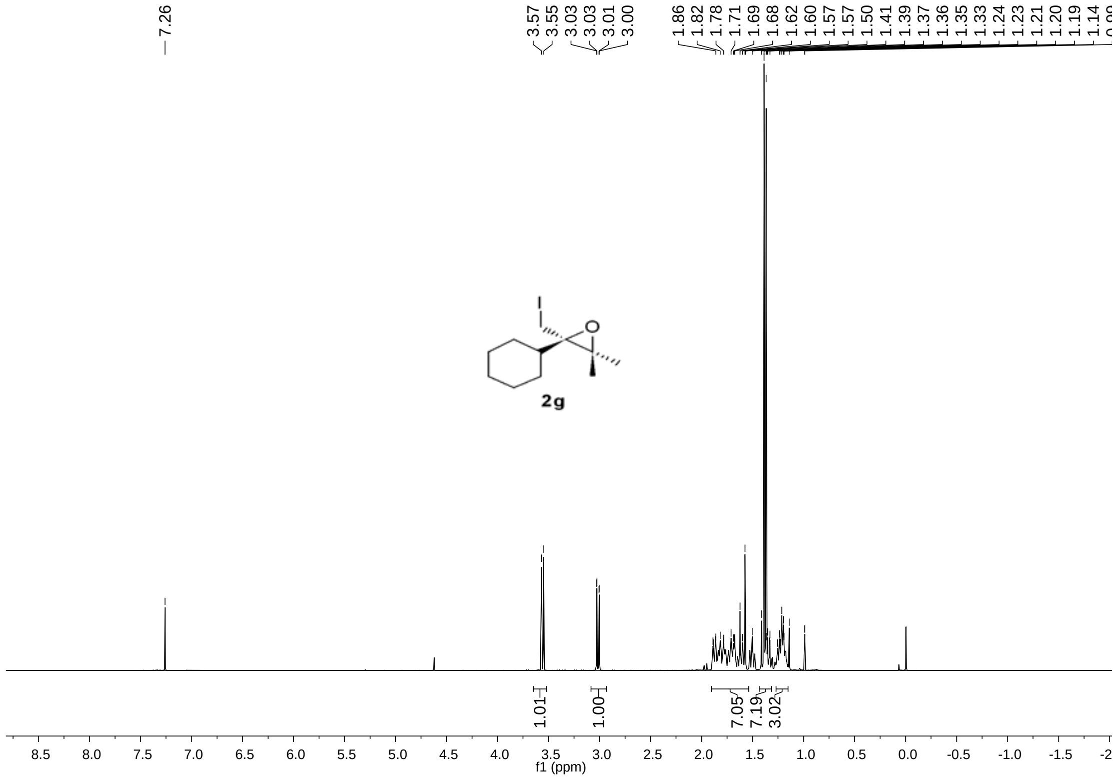
7.26
3.57
3.55
3.03
3.03
3.01
3.00
1.86
1.82
1.78
1.71
1.69
1.68
1.62
1.60
1.57
1.57
1.50
1.41
1.39
1.37
1.36
1.35
1.33
1.24
1.23
1.21
1.20
1.19
1.14
I
O
2g
1.01
1.00
7.05
7.19
3.02
8.5 8.0 7.5 7.0 6.5 6.0 5.5 5.0 4.5 4.0 3.5 3.0 2.5 2.0 1.5 1.0 0.5 0.0 -0.5 -1.0 -1.5 -2
f1 (ppm)

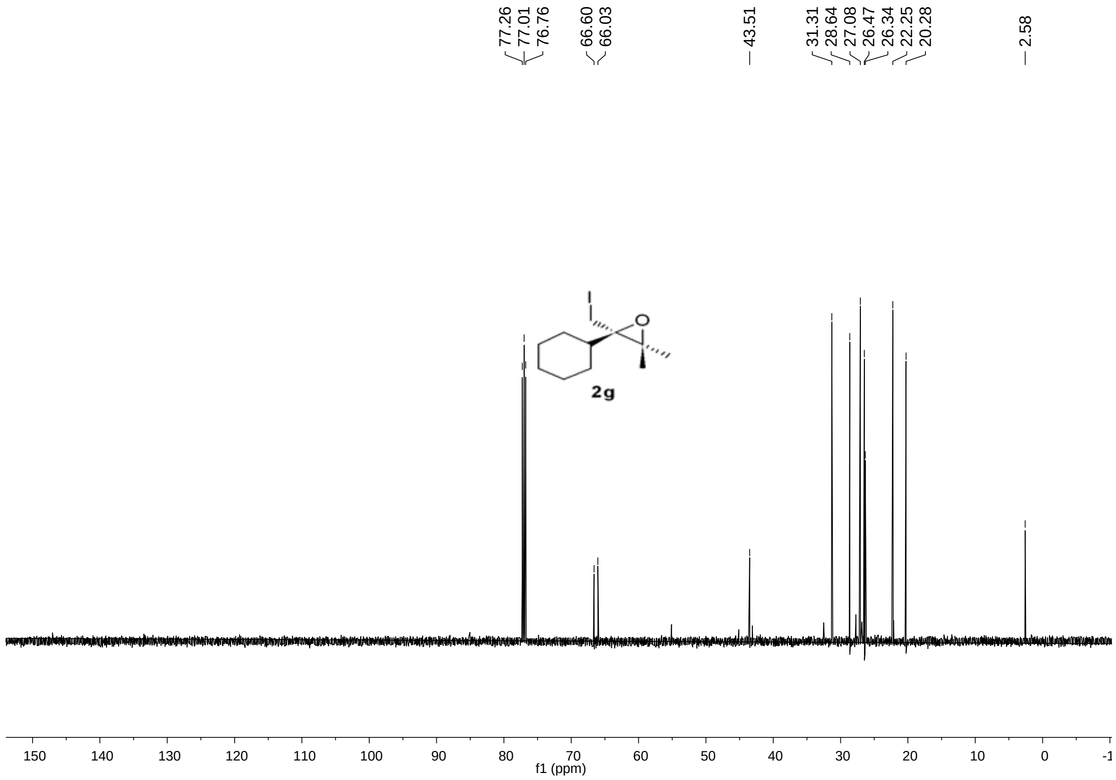
77.26
77.01
76.76
66.60
66.03
43.51
31.31
28.64
27.08
26.47
26.34
22.25
20.28
2.58
2g
150 140 130 120 110 100 90 80 70 60 50 40 30 20 10 0
f1 (ppm)

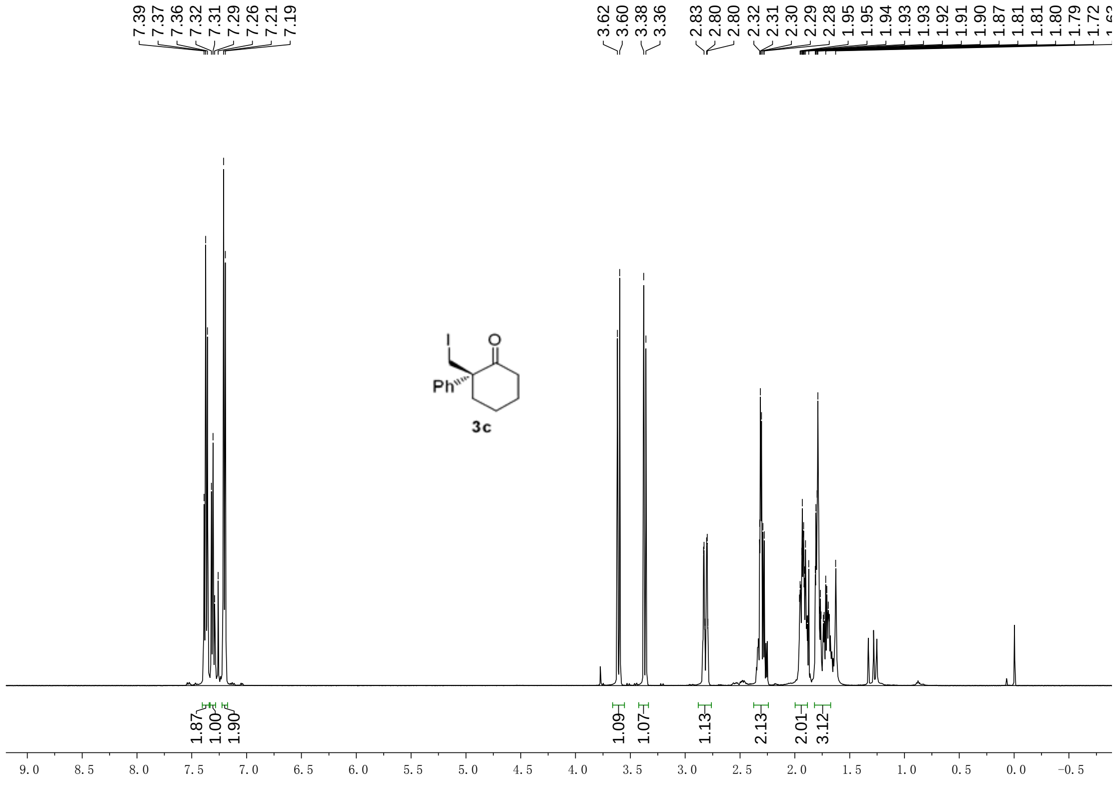
7.39
7.37
7.36
7.32
7.31
7.29
7.26
7.21
7.19
3.62
3.60
3.38
3.36
2.83
2.80
2.80
2.32
2.31
2.30
2.29
2.28
1.95
1.95
1.94
1.93
1.93
1.92
1.91
1.90
1.87
1.81
1.81
1.80
1.79
1.72
O
I
Ph
3c
1.87
1.00
1.90
1.09
1.07
1.13
2.13
2.01
3.12
9.0 8.5 8.0 7.5 7.0 6.5 6.0 5.5 5.0 4.5 4.0 3.5 3.0 2.5 2.0 1.5 1.0 0.5 0.0 -0.5

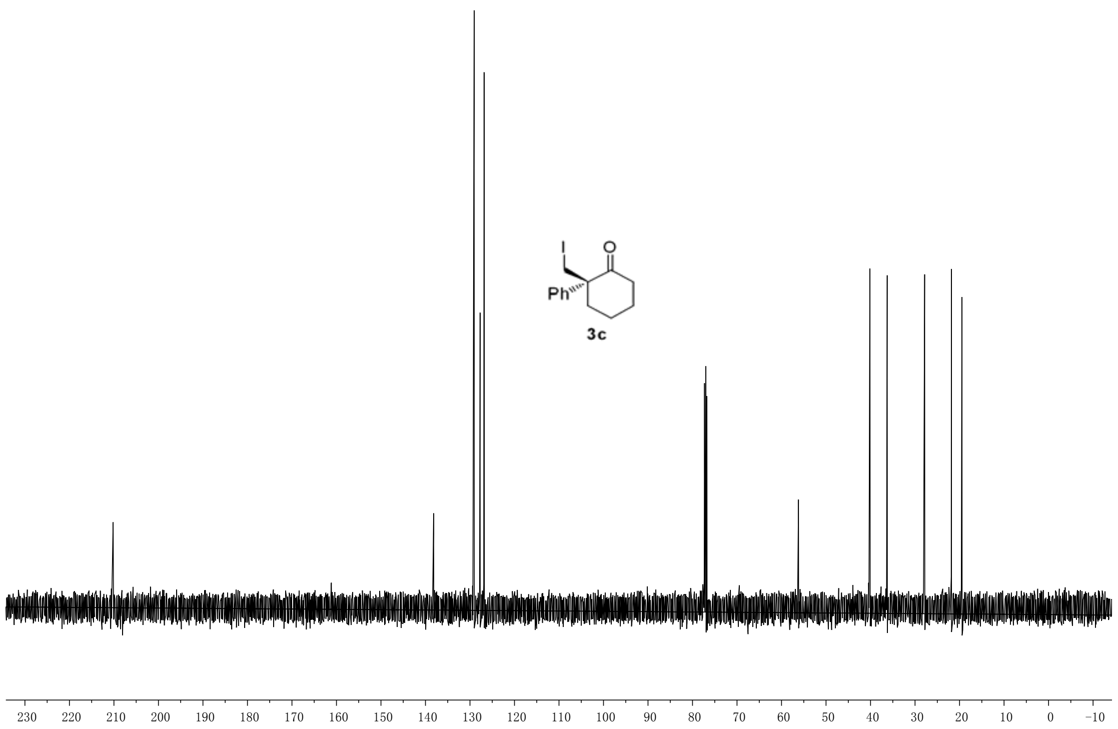

Ph
3c

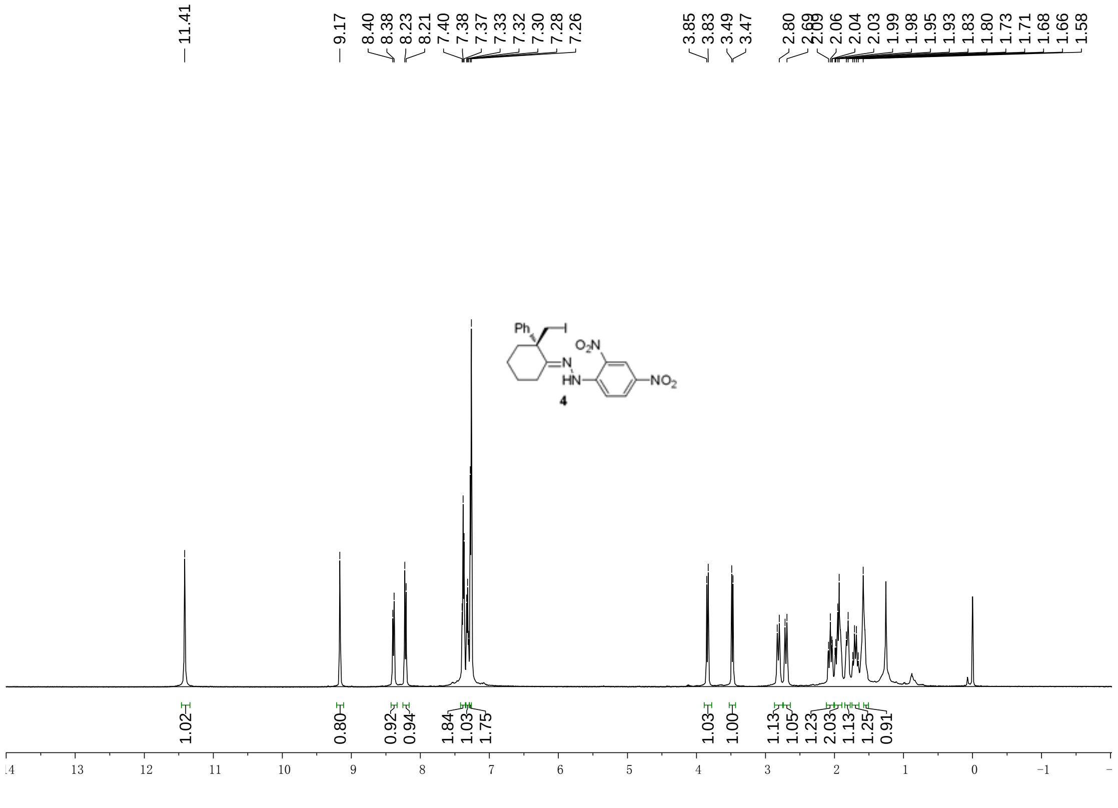

Ph
4

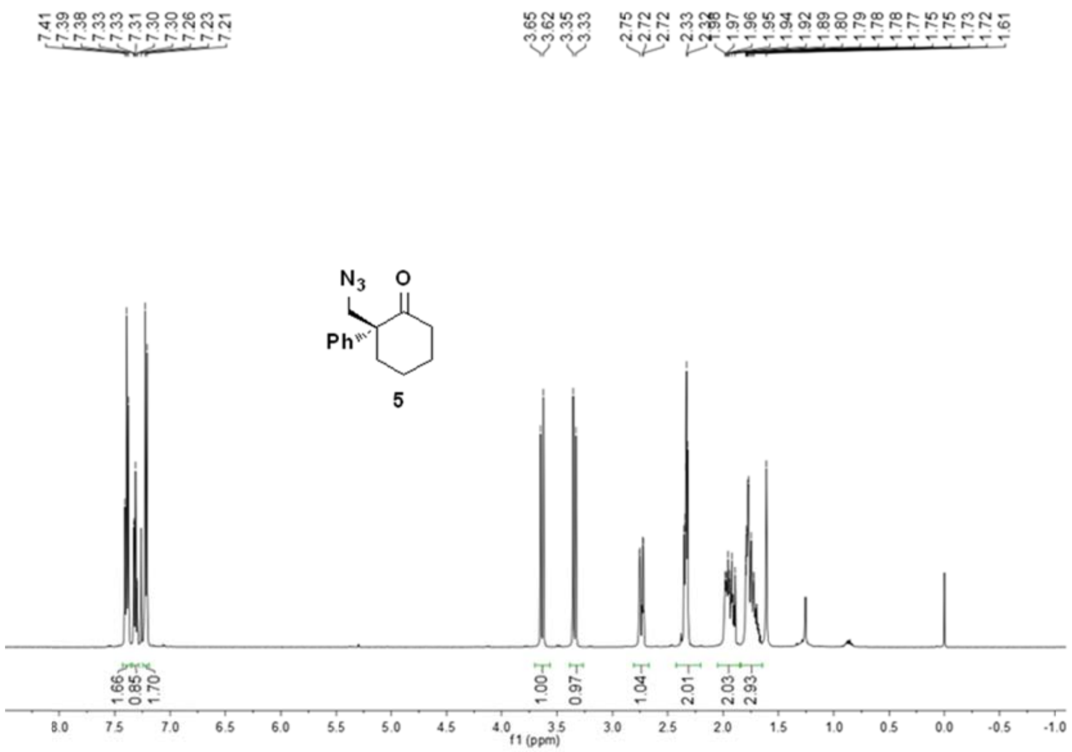
7.41
7.39
7.38
7.33
7.33
7.31
7.30
7.30
7.26
7.23
7.21
3.65
3.62
3.35
3.33
2.75
2.72
2.72
2.33
2.32
1.98
1.97
1.96
1.95
1.94
1.92
1.89
1.80
1.79
1.78
1.78
1.77
1.75
1.75
1.73
1.72
1.61
N3
O
Ph
5
1.66
0.85
1.70
1.00
0.97
1.04
2.01
2.03
2.93
f1 (ppm)

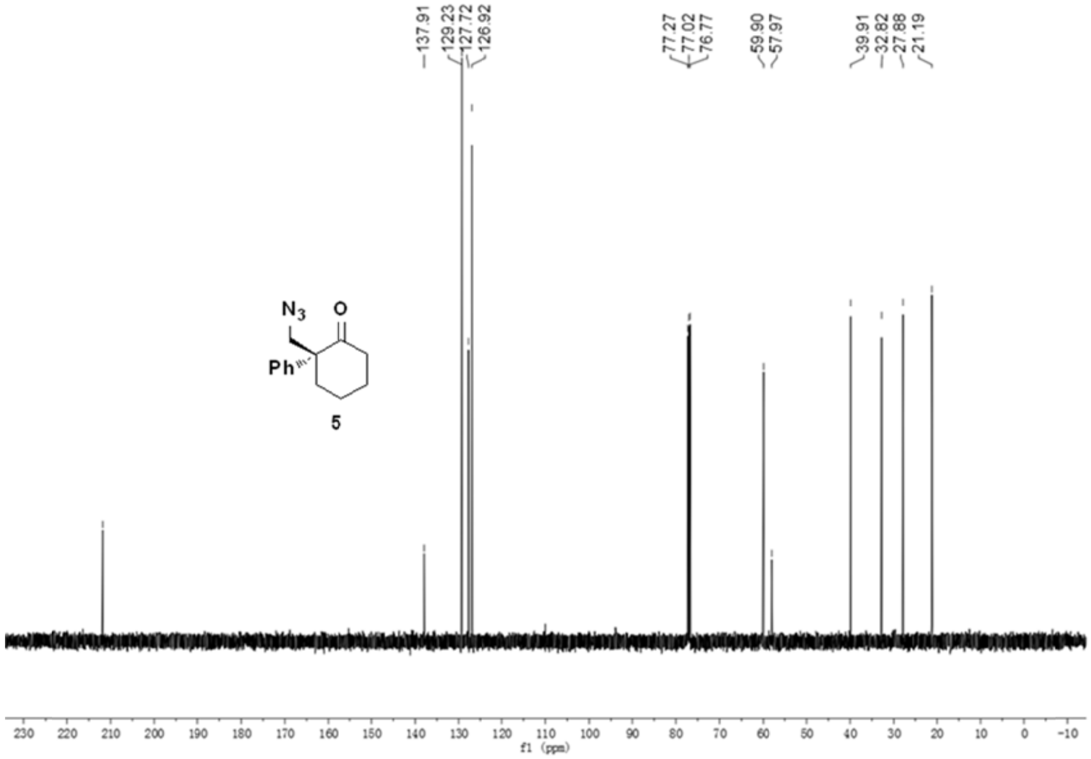
137.91
129.23
127.72
126.92
77.27
77.02
76.77
59.90
57.97
39.91
32.82
27.88
21.19
N3
O
Ph
5
f1 (ppm)

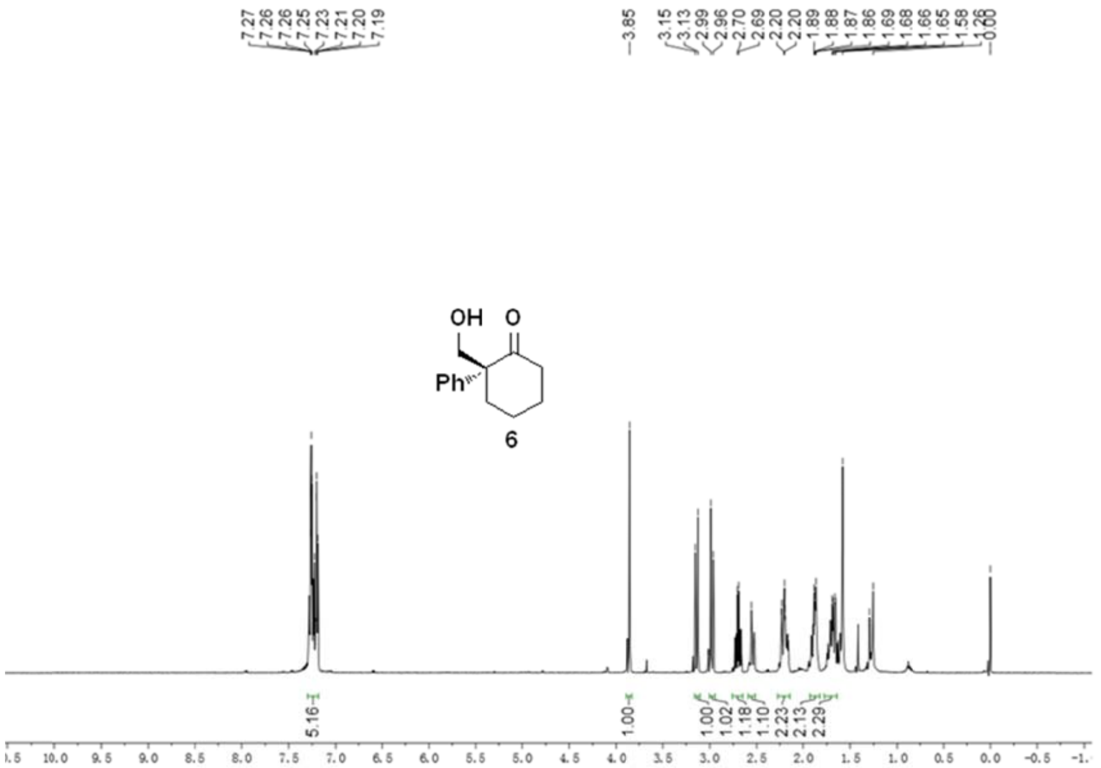
7.27
7.26
7.26
7.25
7.23
7.21
7.20
7.19
3.85
3.15
3.13
2.99
2.96
2.70
2.69
2.20
2.20
1.89
1.87
1.86
1.69
1.68
1.66
1.65
1.58
1.26
0.00
OH
O
Ph
6
5.16
1.00
1.00
1.02
1.18
1.10
2.23
2.13
2.29

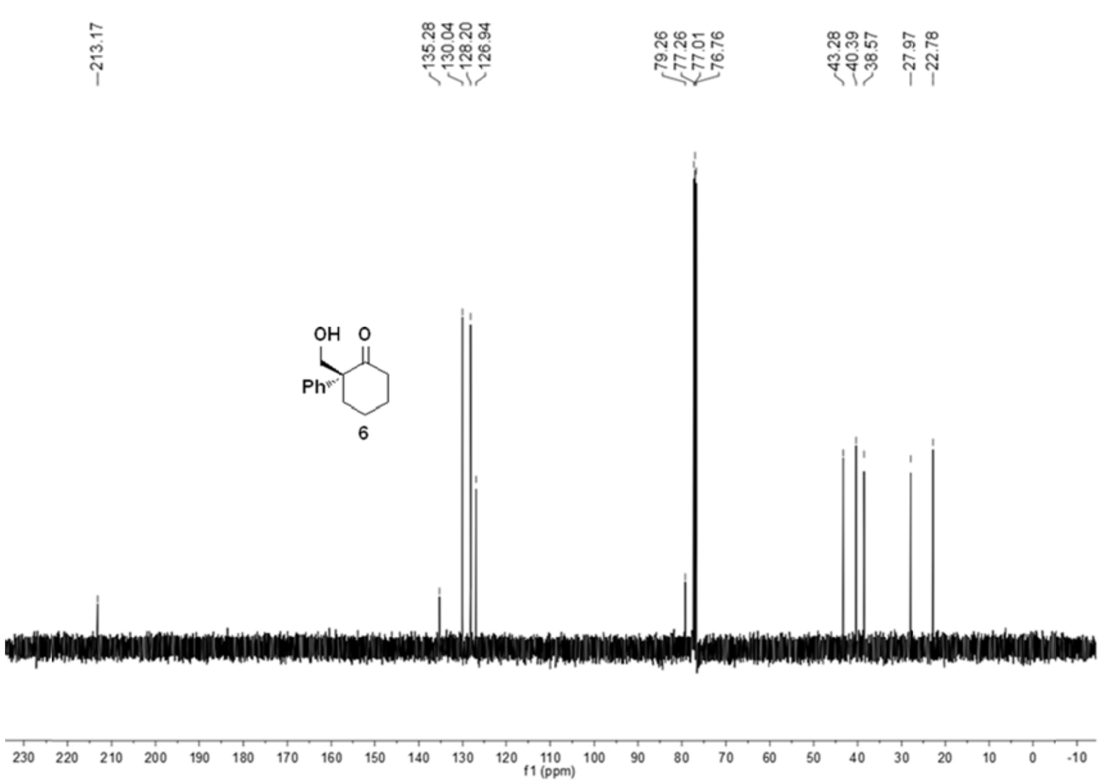
213.17
135.28
130.04
128.20
126.94
79.26
77.26
77.01
76.76
43.28
40.39
38.57
27.97
22.78
OH
O
Ph
6
f1 (ppm)

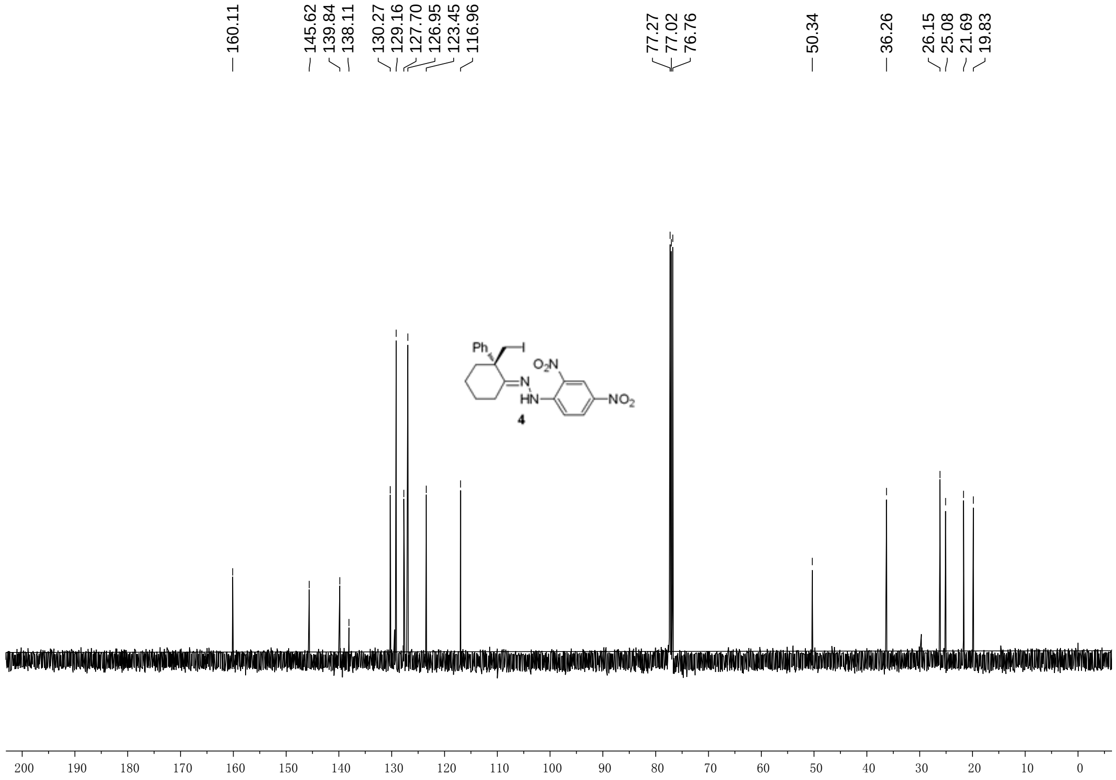

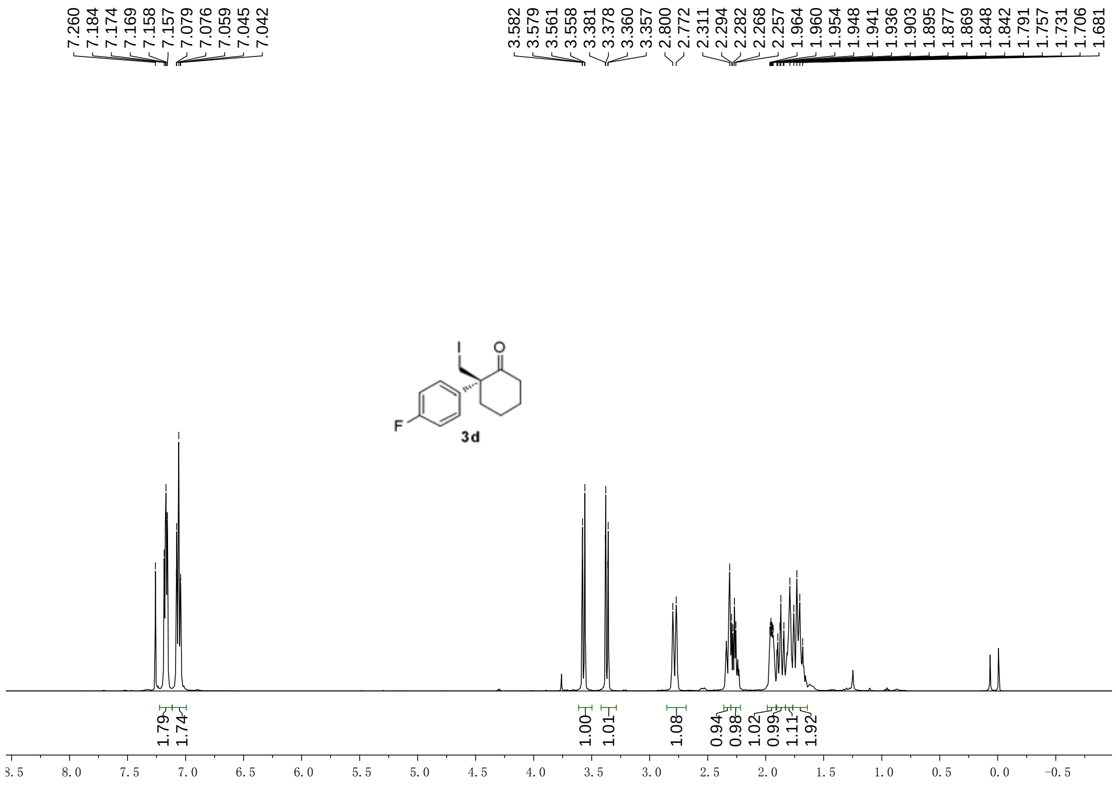

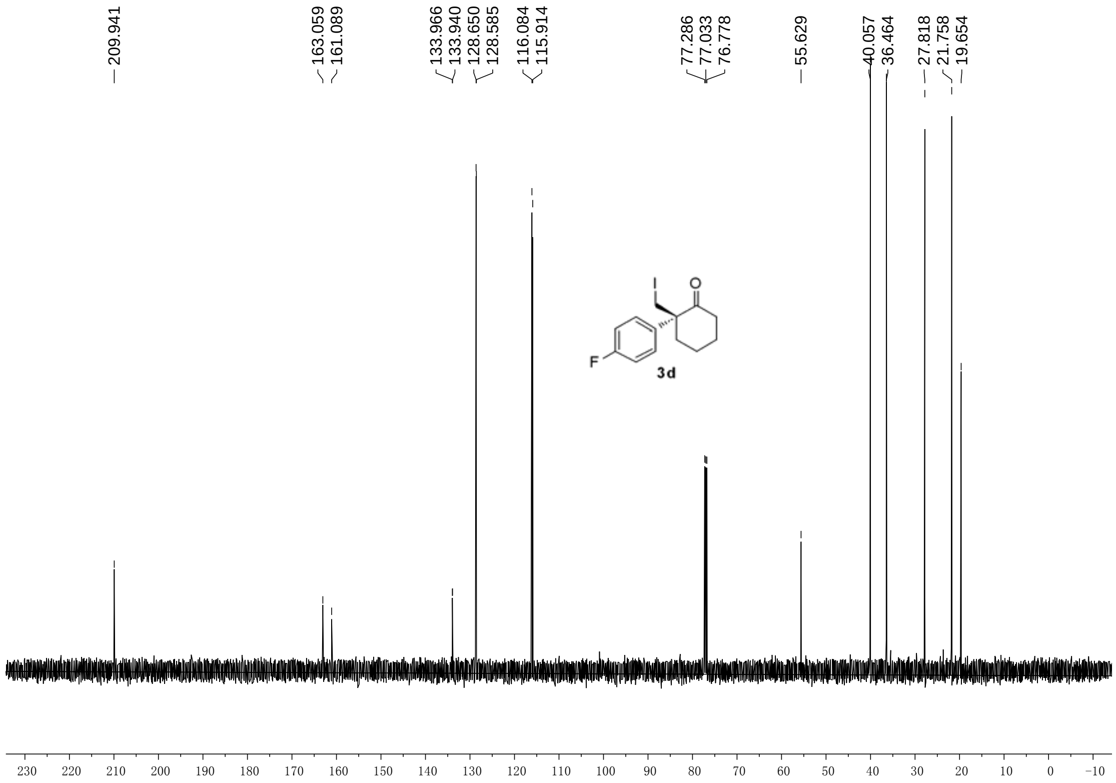
209.941
163.059
161.089
133.966
133.940
128.650
128.585
116.084
115.914
77.286
77.033
76.778
55.629
40.057
36.464
27.818
21.758
19.654
I
O
F
3d
230 220 210 200 190 180 170 160 150 140 130 120 110 100 90 80 70 60 50 40 30 20 10 0 −10

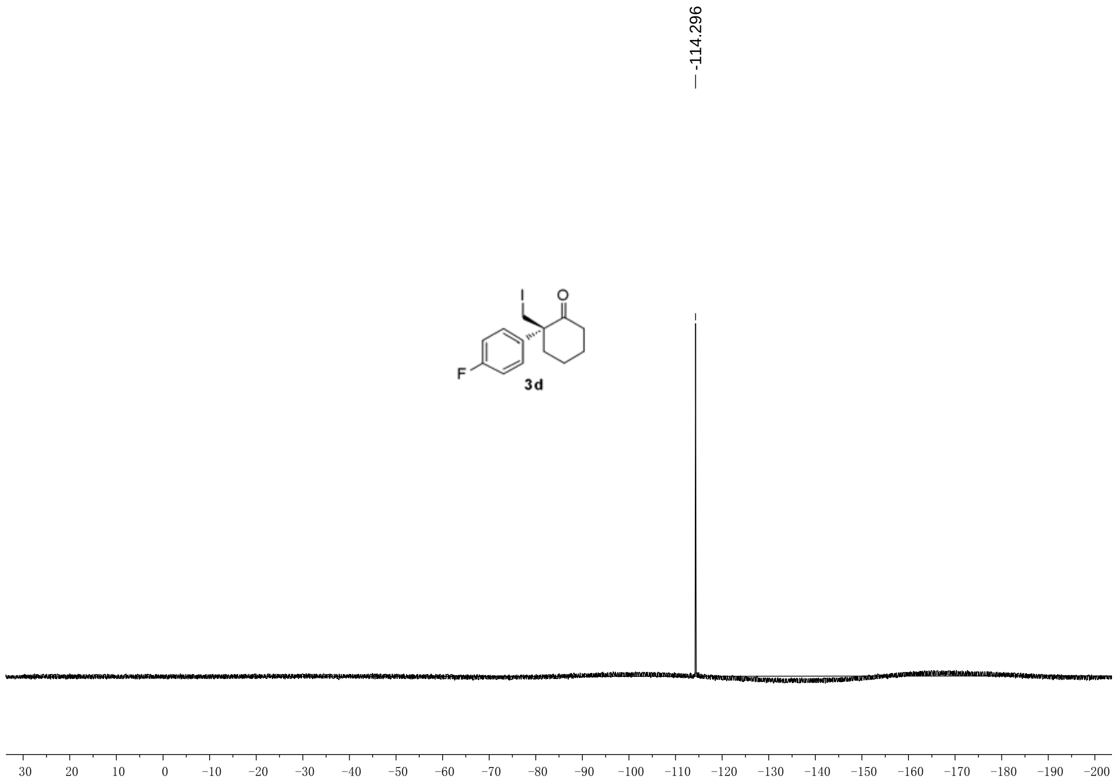
-114.296
I
O
F
3d
30 20 10 0 −10 −20 −30 −40 −50 −60 −70 −80 −90 −100 −110 −120 −130 −140 −150 −160 −170 −180 −190 −200

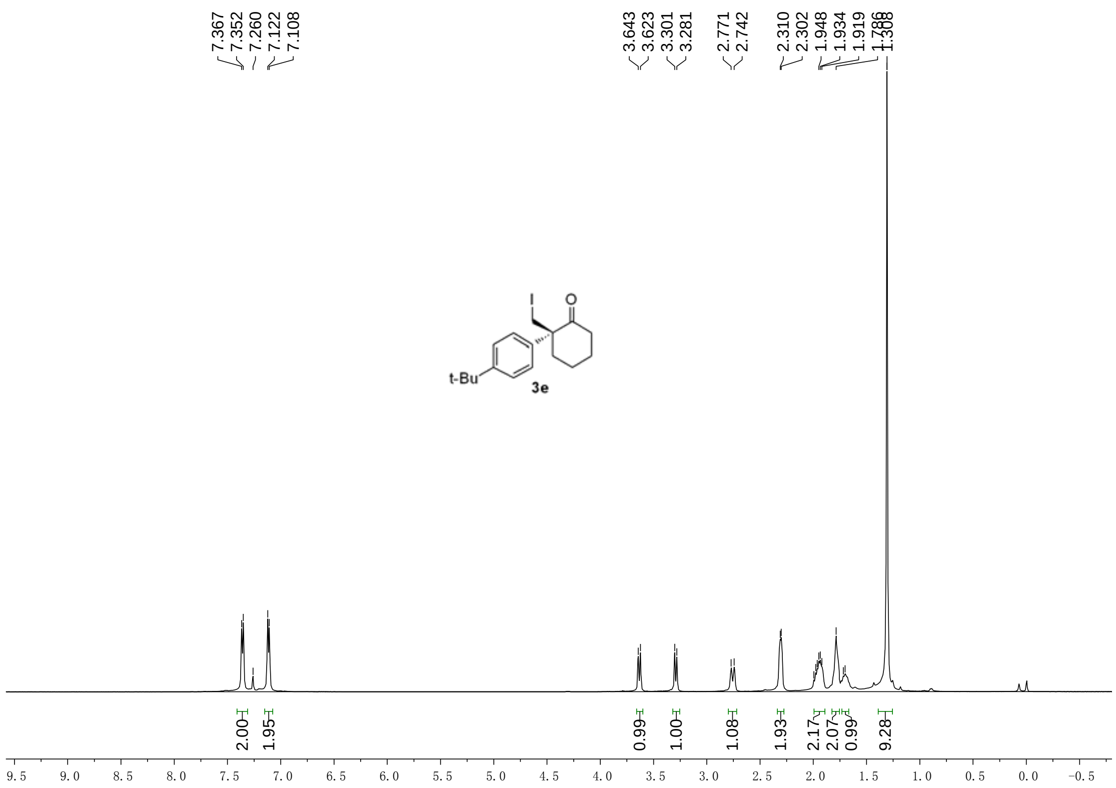

7.367
7.352
7.260
7.122
7.108
3.643
3.623
3.301
3.281
2.771
2.742
2.310
2.302
1.948
1.934
1.919
1.308
2.00
1.95
0.99
1.00
1.08
1.93
2.17
2.07
0.99
9.28
t-Bu
I
O
3e

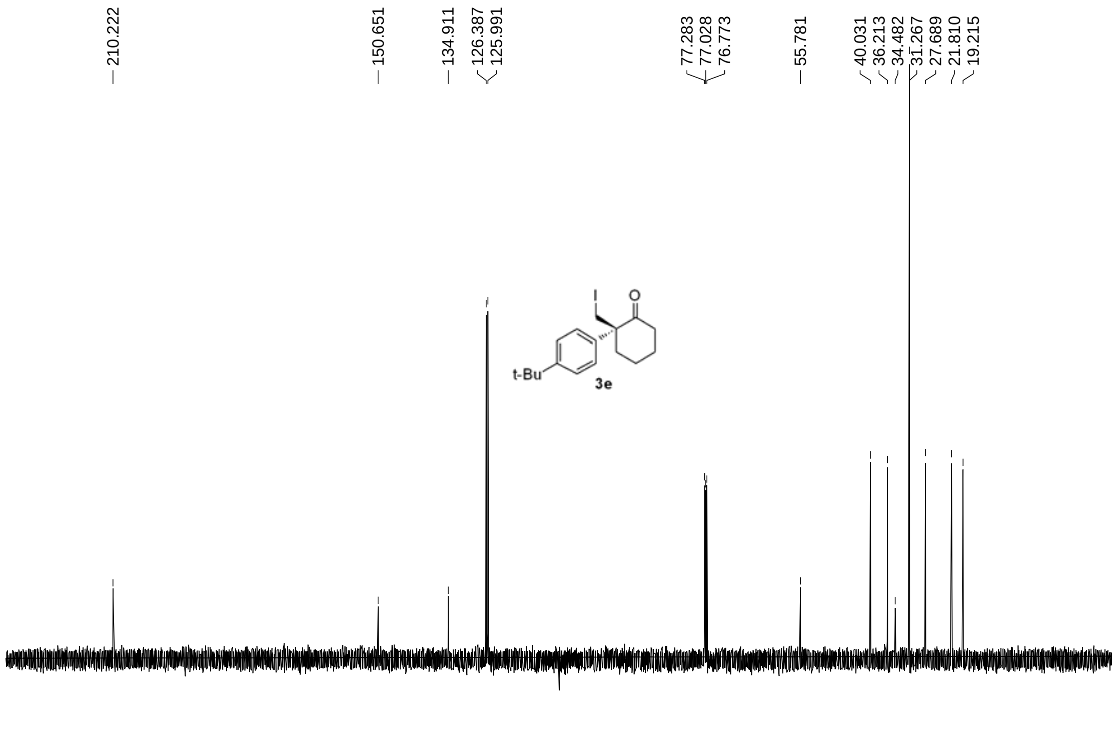

210.222
150.651
134.911
126.387
125.991
77.283
77.028
76.773
55.781
40.031
36.213
34.482
31.267
27.689
21.810
19.215
t-Bu
I
O
3e

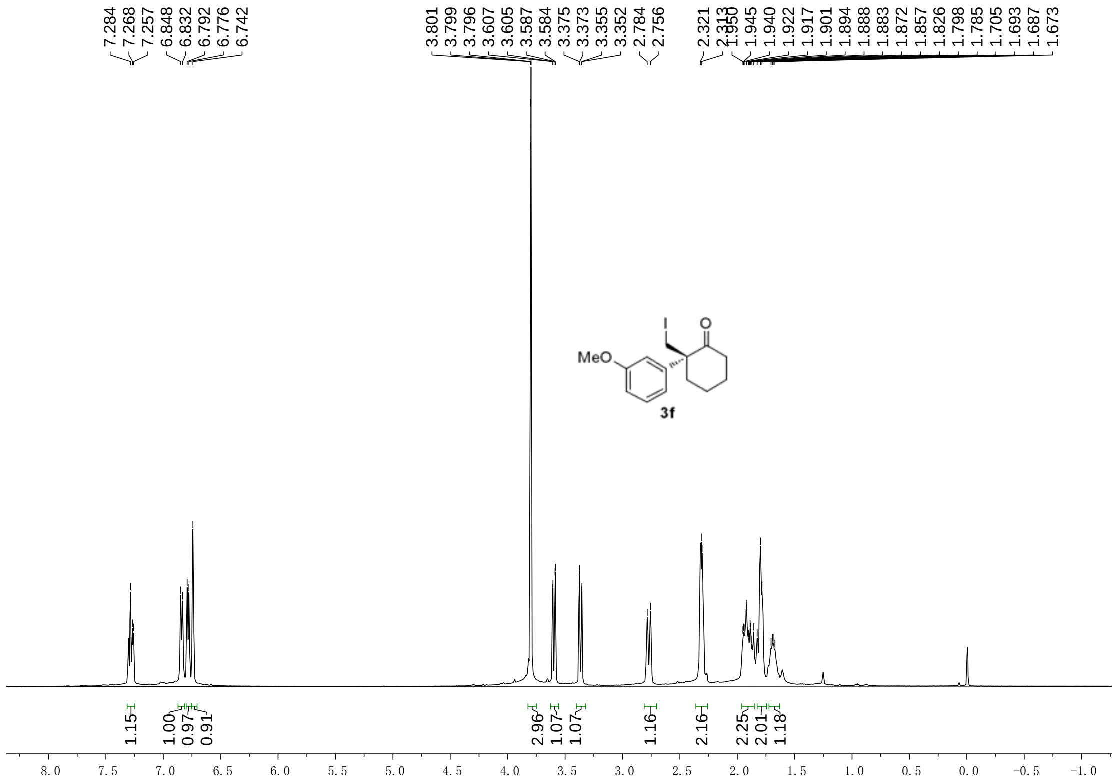
7.284
7.268
7.257
6.848
6.832
6.792
6.776
6.742
3.801
3.799
3.796
3.607
3.605
3.587
3.584
3.375
3.373
3.355
3.352
2.784
2.756
2.321
2.313
1.950
1.945
1.940
1.922
1.917
1.901
1.894
1.888
1.883
1.872
1.857
1.826
1.798
1.785
1.705
1.693
1.687
1.673
MeO
I
O
3f
1.15
1.00
0.97
0.91
2.96
1.07
1.07
1.16
2.16
2.25
2.01
1.18
8.0 7.5 7.0 6.5 6.0 5.5 5.0 4.5 4.0 3.5 3.0 2.5 2.0 1.5 1.0 0.5 0.0 −0.5 −1.0

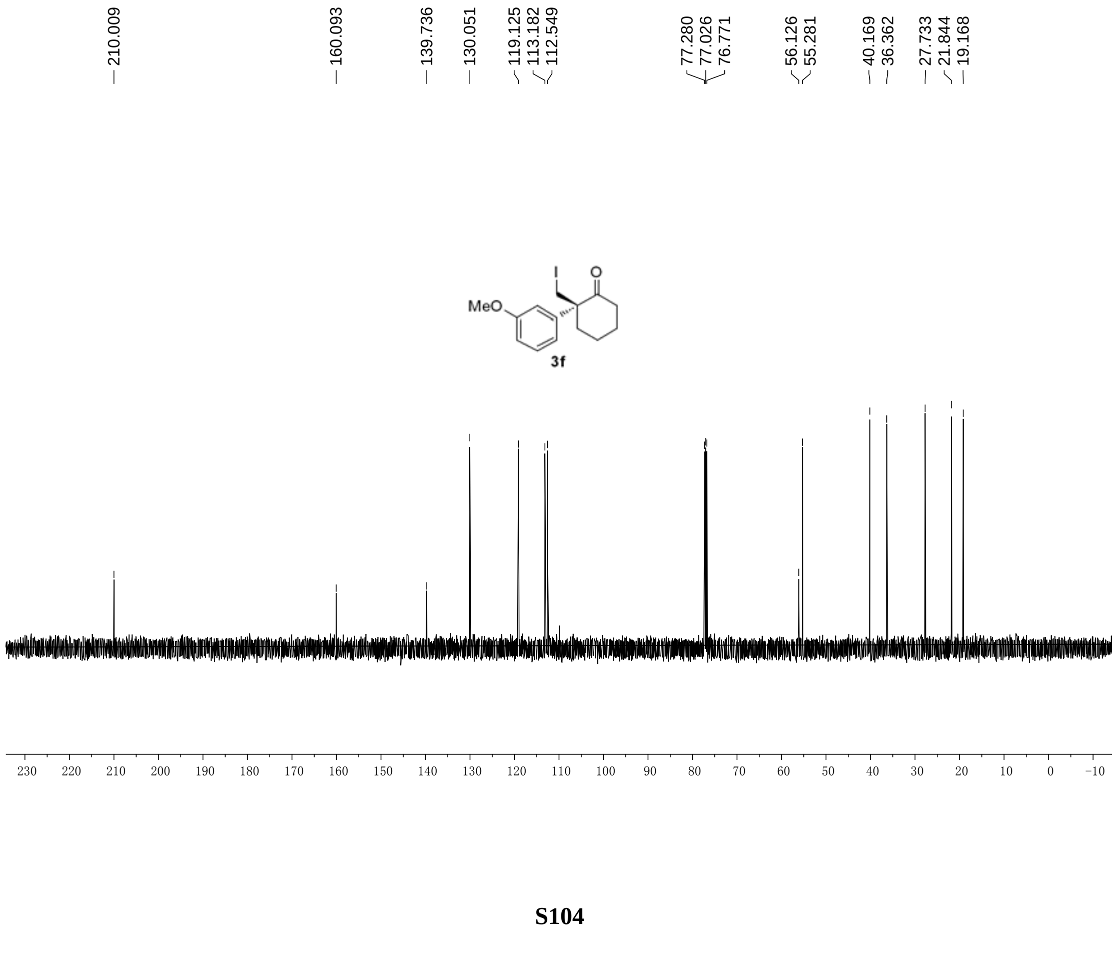
210.009
160.093
139.736
130.051
119.125
113.182
112.549
77.280
77.026
76.771
56.126
55.281
40.169
36.362
27.733
21.844
19.168
MeO
I
O
3f
230 220 210 200 190 180 170 160 150 140 130 120 110 100 90 80 70 60 50 40 30 20 10 0 −10

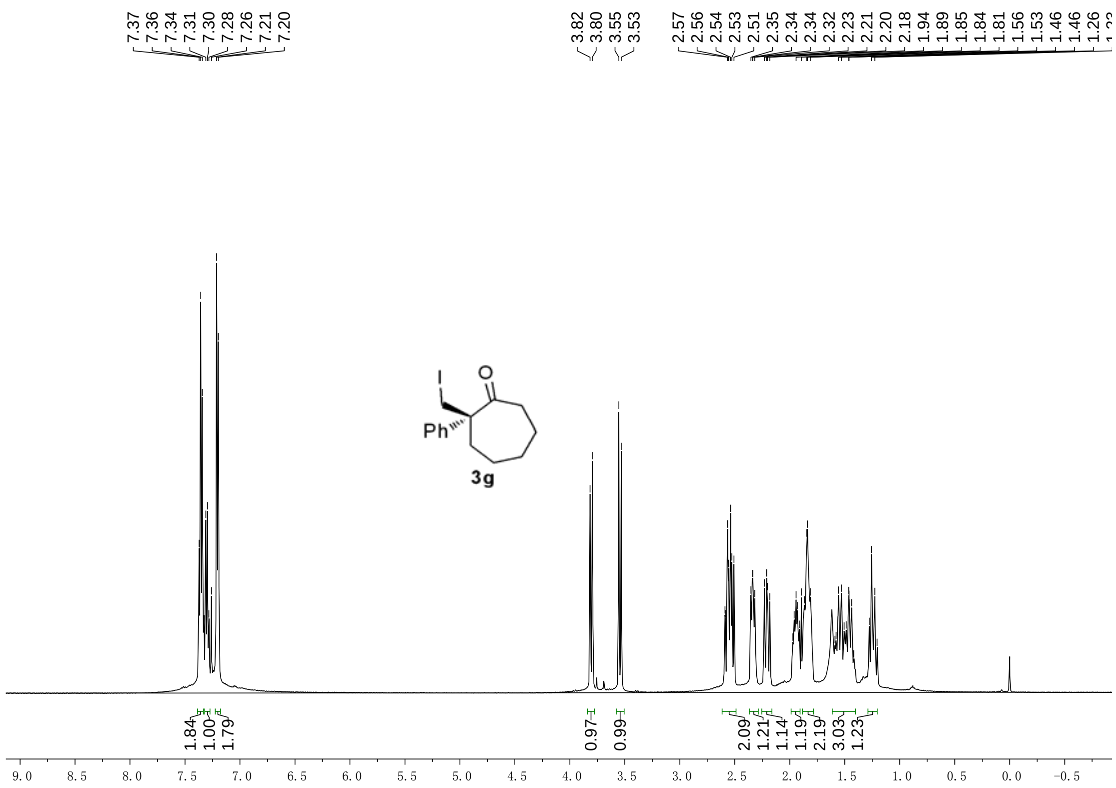

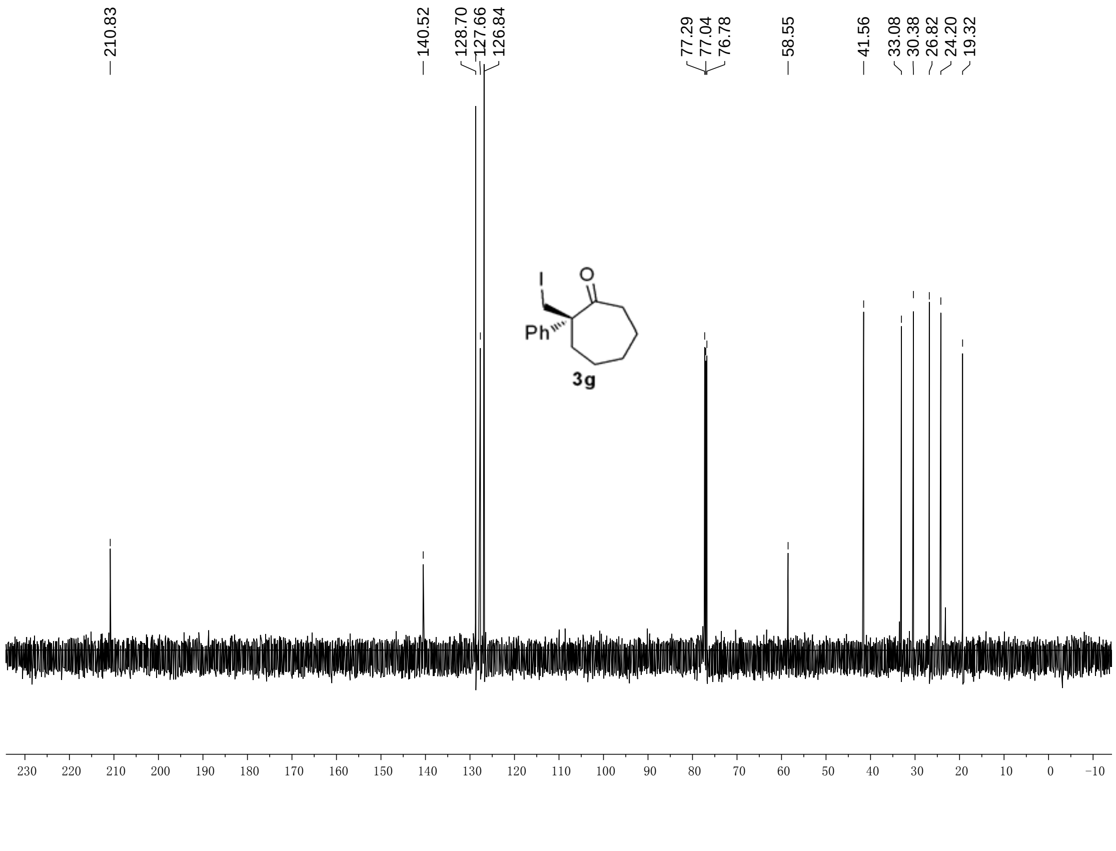

Operator:Administrator Timebase:HPLC Sequence:201310-DAD

Page 1-1
2014-9-28 9:54 上午

2919 SZG-5-35-1+- ID3 98515 214 0.5 15

Sample Name:	SZG-5-35-1+- ID3 98515 214 0.5 15	*Injection Volume:*	1.0
Vial Number:	BD3	*Channel:*	UV_VIS_2
Sample Type:	unknown	*Wavelength:*	214.0
Control Program:	test-dad	*Bandwidth:*	4
Quantif. Method:	WXL	*Dilution Factor:*	1.0000
Recording Time:	2014-9-15 17:41	*Sample Weight:*	1.0000
Run Time (min):	13.00	*Sample Amount:*	1.0000

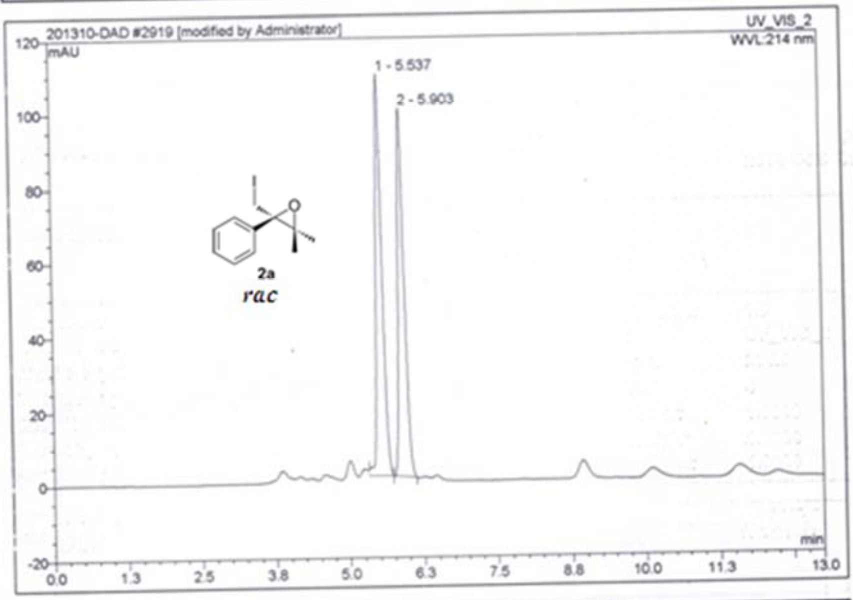

No.	Ret.Time min	Peak Name	Height mAU	Area mAU*min	Rel.Area %	Amount	Type
1	5.54	n.a.	108.318	12.952	51.20	n.a.	Mb*
2	5.90	n.a.	99.150	12.343	48.80	n.a.	bMB*
Total:			207.467	25.296	100.00	0.000	

1998 SZG-9-40 ID3 98515 214 0.5 15

Sample Name:	SZG-9-40 ID3 98515 214 0.5 15	*Injection Volume:*	1.0
Vial Number:	RD5	*Channel:*	UV_VIS_2
Sample Type:	unknown	*Wavelength:*	214.0
Control Program:	test-dad2	*Bandwidth:*	4
Quantif. Method:	WXL	*Dilution Factor:*	1.0000
Recording Time:	2014-5-19 15:36	*Sample Weight:*	1.0000
Run Time (min):	11.51	*Sample Amount:*	1.0000

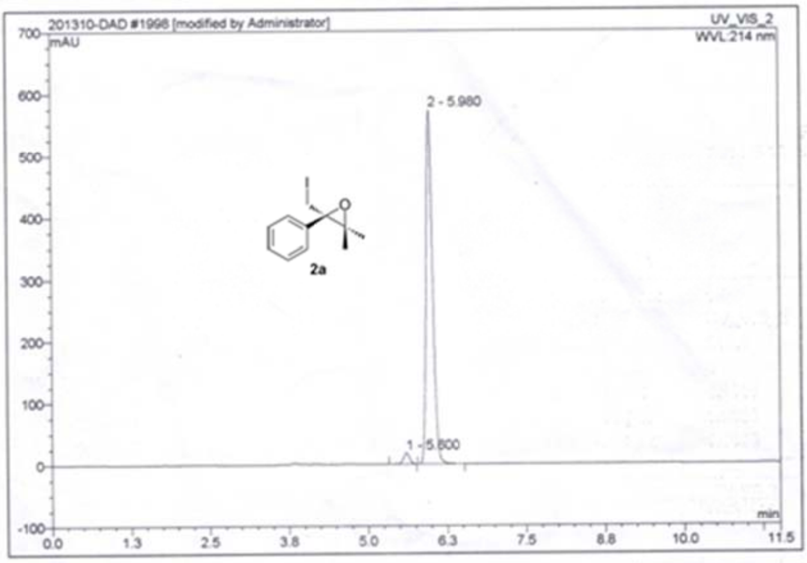

No.	Ret.Time min	Peak Name	Height mAU	Area mAU*min	Rel.Area %	Amount	Type
1	5.60	n.a.	18.626	2.057	2.92	n.a.	BMB*
2	5.98	n.a.	572.307	68.484	97.08	n.a.	BMB*
**Total:**			590.933	70.541	100.00	0.000	

2964 SZG-5-35-R ID3 98515 214 0.5 15

Sample Name:	SZG-5-35-R ID3 98515 214 0.5 15	*Injection Volume:*	1.0
Vial Number:	GE4	*Channel:*	UV_VIS_2
Sample Type:	unknown	*Wavelength:*	214.0
Control Program:	test-dad	*Bandwidth:*	4
Quantif. Method:	WXL	*Dilution Factor:*	1.0000
Recording Time:	2014-9-19 11:04	*Sample Weight:*	1.0000
Run Time (min):	25.98	*Sample Amount:*	1.0000

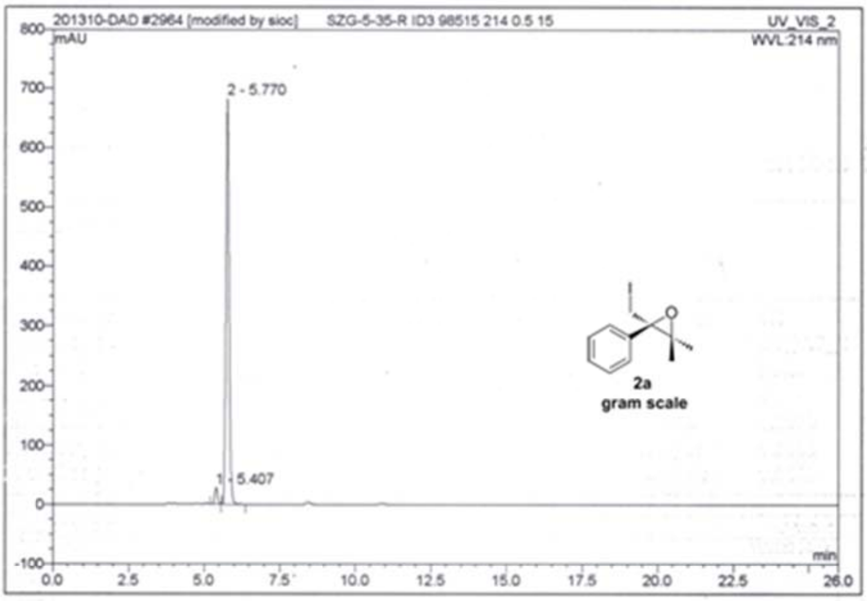

No.	Ret.Time min	Peak Name	Height mAU	Area mAU*min	Rel.Area %	Amount	Type
1	5.41	n.a.	28.271	3.271	3.66	n.a.	BMB*
2	5.77	n.a.	682.521	86.168	96.34	n.a.	BMB*
Total:			710.793	89.439	100.00	0.000	

Operator:Administrator Timebase:HPLC Sequence:201310-DAD

Page 1-
2014-3-10 10:06 上[illegible]

1387 SZG-3-23-1+- ID3 98515 214 0.7

Sample Name:	SZG-3-23-1+- ID3 98515 214 0.7	*Injection Volume:*	3.0
Vial Number:	RA2	*Channel:*	UV_VIS_2
Sample Type:	unknown	*Wavelength:*	214.0
Control Program:	test-dad3	*Bandwidth:*	4
Quantif. Method:	WXL	*Dilution Factor:*	1.0000
Recording Time:	2014-3-3 11:09	*Sample Weight:*	1.0000
Run Time (min):	7.09	*Sample Amount:*	1.0000

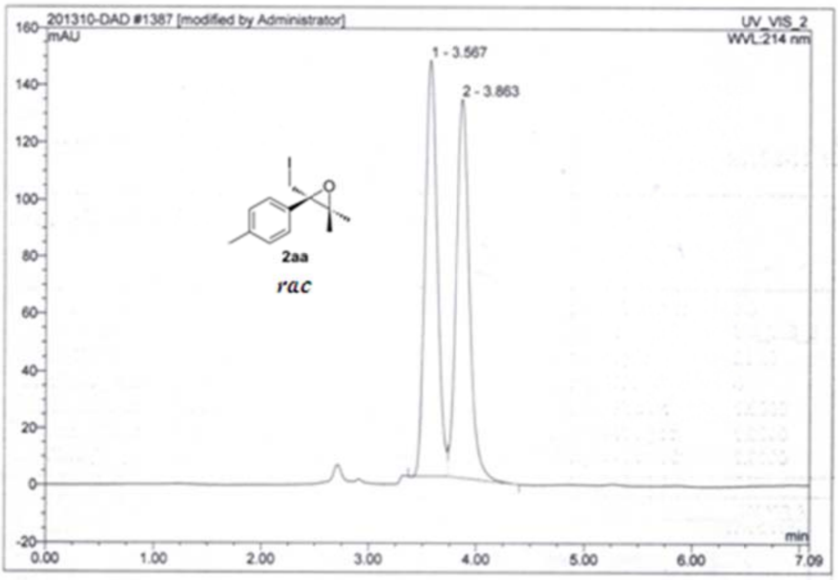

No.	Ret.Time min	Peak Name	Height mAU	Area mAU*min	Rel.Area %	Amount	Type
1	3.57	n.a.	145.763	19.145	50.01	n.a.	BM *
2	3.86	n.a.	132.683	19.137	49.99	n.a.	MB*
Total:			278.446	38.281	100.00	0.000	

653 SZG-4-10-1 ID3 98515 214 0.7

Sample Name:	SZG-4-10-1 ID3 98515 214 0.7	*Injection Volume:*	0.8
Vial Number:	GD2	*Channel:*	UV_VIS_1
Sample Type:	unknown	*Wavelength:*	214
Control Program:	WXL-2014-1	*Bandwidth:*	n.a.
Quantif. Method:	WXL	*Dilution Factor:*	1.0000
Recording Time:	2014/5/26 11:01	*Sample Weight:*	1.0000
Run Time (min):	5.27	*Sample Amount:*	1.0000

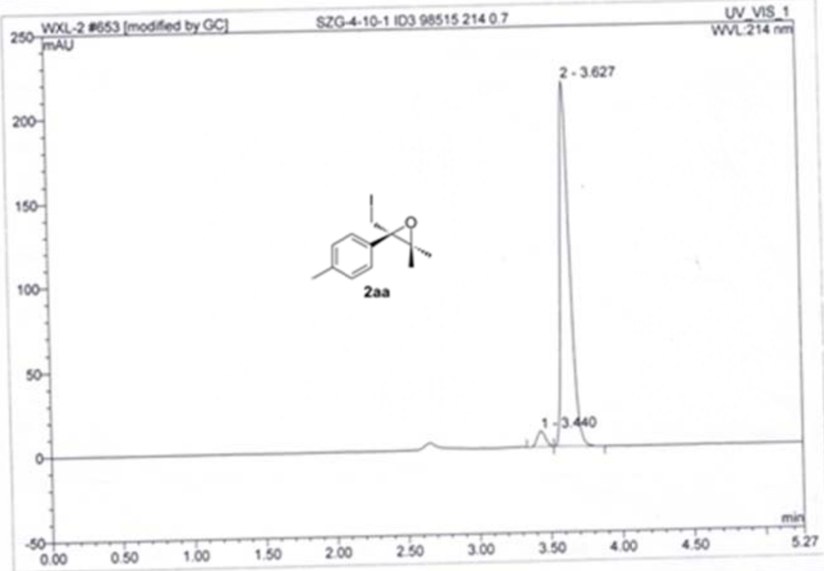

No.	Ret.Time min	Peak Name	Height mAU	Area mAU*min	Rel.Area %	Amount	Type
1	3.44	n.a.	9.311	0.613	3.66	n.a.	BMb*
2	3.63	n.a.	216.894	16.154	96.34	n.a.	bMB*
Total:			226.205	16.767	100.00	0.000	

Operator:Administrator Timebase:HPLC Sequence:201310-DAD

Page 1-
2014-3-10 1:30 下午

1464 SZG-3-28-1+- ADH 98515 214 0.4

Sample Name:	SZG-3-28-1+- ADH 98515 214 0.4	*Injection Volume:*	0.6
Vial Number:	GB1	*Channel:*	UV_VIS_2
Sample Type:	unknown	*Wavelength:*	214.0
Control Program:	test-dad2	*Bandwidth:*	4
Quantif. Method:	WXL	*Dilution Factor:*	1.0000
Recording Time:	2014-3-10 12:41	*Sample Weight:*	1.0000
Run Time (min):	13.82	*Sample Amount:*	1.0000

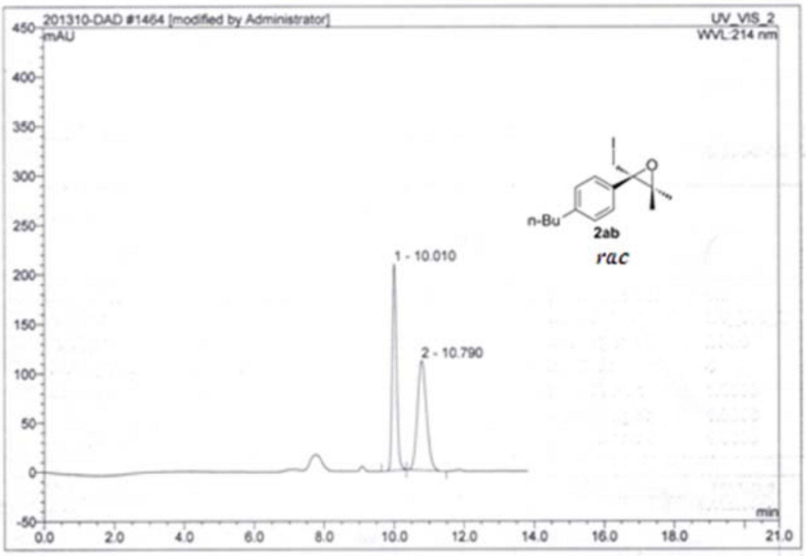

No.	Ret.Time min	Peak Name	Height mAU	Area mAU*min	Rel.Area %	Amount	Type
1	10.01	n.a.	209.568	29.418	46.88	n.a.	BMb*
2	10.79	n.a.	111.552	33.339	53.12	n.a.	bMB*
Total:			321.120	62.757	100.00	0.000	

2066 XZL-4-10-2 AD-H 98515 214 0.4

Sample Name:	XZL-4-10-2 AD-H 98515 214 0.4	*Injection Volume:*	1.0
Vial Number:	GE2	*Channel:*	UV_VIS_2
Sample Type:	unknown	*Wavelength:*	214.0
Control Program:	test-dad2	*Bandwidth:*	4
Quantif. Method:	WXL	*Dilution Factor:*	1.0000
Recording Time:	2014-5-26 14:05	*Sample Weight:*	1.0000
Run Time (min):	18.64	*Sample Amount:*	1.0000

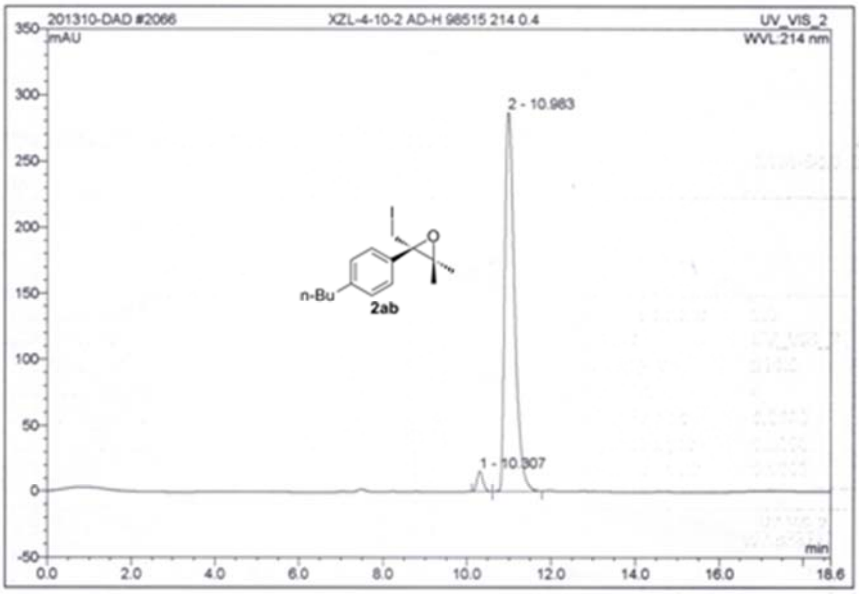

No.	Ret.Time min	Peak Name	Height mAU	Area mAU*min	Rel.Area %	Amount	Type
1	10.31	n.a.	15.920	2.682	3.36	n.a.	BMB
2	10.98	n.a.	287.141	77.081	96.64	n.a.	BMB
Total:			303.062	79.763	100.00	0.000	

rator:Administrator Timebase:HPLC Sequence:201310-DAD

Page 1-1
2014-5-28 5:53 下午

2090 SZG-3-79-1+- PC-3 982 214 0.5

Sample Name:	SZG-3-79-1+- PC-3 982 214 0.5	*Injection Volume:*	1.0
Vial Number:	GB5	*Channel:*	UV_VIS_2
Sample Type:	unknown	*Wavelength:*	214.0
Control Program:	test-dad2	*Bandwidth:*	4
Quantif. Method:	WXL	*Dilution Factor:*	1.0000
Recording Time:	2014-5-28 9:55	*Sample Weight:*	1.0000
Run Time (min):	13.18	*Sample Amount:*	1.0000

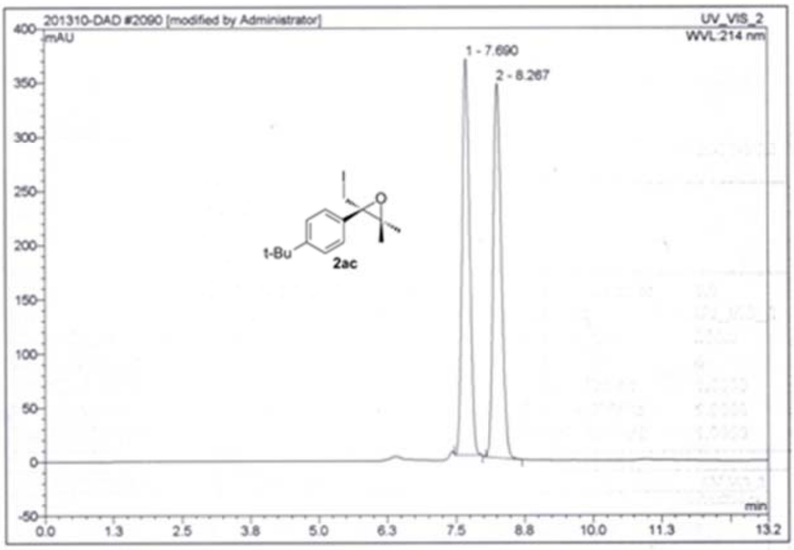

No.	Ret.Time min	Peak Name	Height mAU	Area mAU*min	Rel.Area %	Amount	Type
1	7.69	n.a.	365.988	58.532	49.77	n.a.	MB*
2	8.27	n.a.	345.874	59.082	50.23	n.a.	BMB
Total:			711.863	117.614	100.00	0.000	

Operator:Administrator Timebase:HPLC Sequence:201310-DAD

Page
2014-5-28 5:53 下

2091 SZG-4-10-3 PC-3 982 214 0.5

Sample Name:	SZG-4-10-3 PC-3 982 214 0.5	Injection Volume:	1.0
Vial Number:	GB6	Channel:	UV_VIS_2
Sample Type:	unknown	Wavelength:	214.0
Control Program:	test-dad2	Bandwidth:	4
Quantif. Method:	WXL	Dilution Factor:	1.0000
Recording Time:	2014-5-28 10:10	Sample Weight:	1.0000
Run Time (min):	11.29	Sample Amount:	1.0000

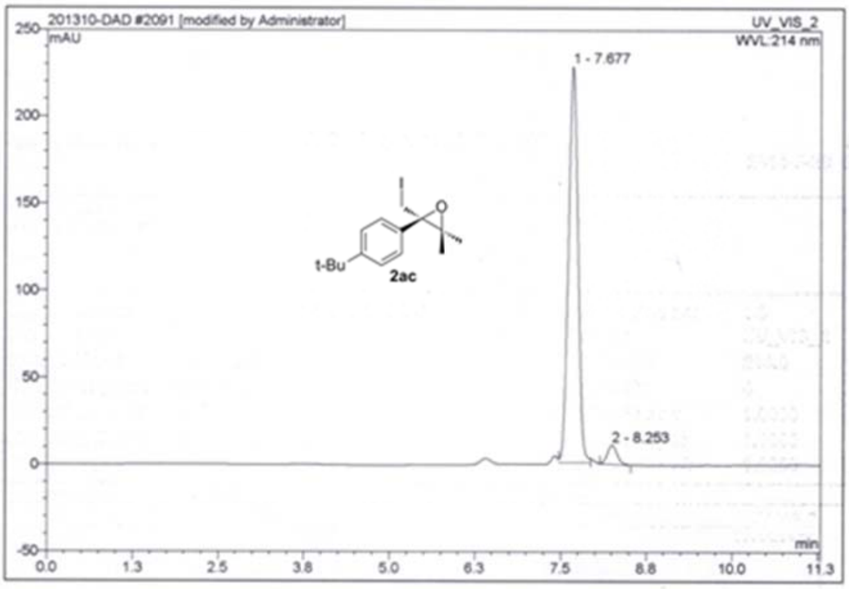

No.	Ret.Time min	Peak Name	Height mAU	Area mAU*min	Rel.Area %	Amount	Type
1	7.68	n.a.	227.619	36.677	95.38	n.a.	M *
2	8.25	n.a.	10.888	1.778	4.62	n.a.	BMB
Total:			238.508	38.455	100.00	0.000	

Operator:GC Timebase:U3000 Sequence:WXL-2

Page 1-
2014/6/9 1:24 下午

775 SXG-4-4-2-1+- ID3 982 214 0.7

Sample Name:	SXG-4-4-2-1+- ID3 982 214 0.7	Injection Volume:	1.0
Vial Number:	RB3	Channel:	UV_VIS_1
Sample Type:	unknown	Wavelength:	214
Control Program:	WXL-2014-1	Bandwidth:	n.a.
Quantif. Method:	WXL	Dilution Factor:	1.0000
Recording Time:	2014/6/9 12:03	Sample Weight:	1.0000
Run Time (min):	11.69	Sample Amount:	1.0000

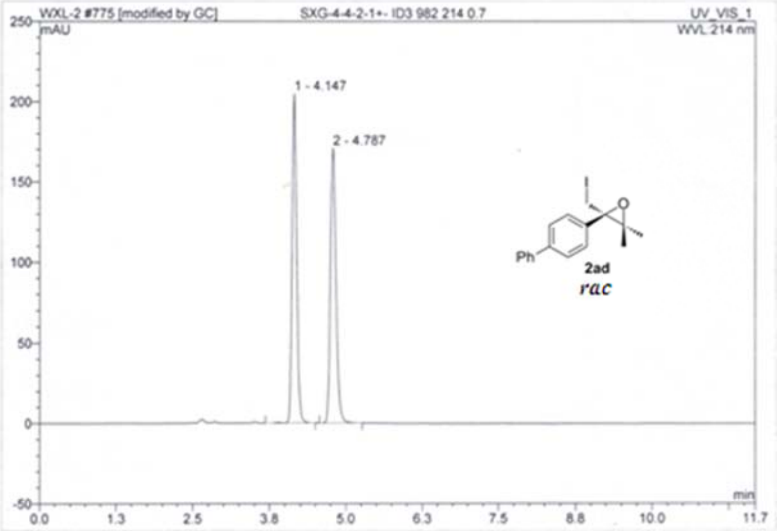

No.	Ret.Time min	Peak Name	Height mAU	Area mAU*min	Rel.Area %	Amount	Type
1	4.15	n.a.	204.836	17.718	49.76	n.a.	BMB*
2	4.79	n.a.	170.788	17.892	50.24	n.a.	BMB*
Total:			375.624	35.610	100.00	0.000	

776 SXG-4-20-2 ID3 982 214 0.7

Sample Name:	SXG-4-20-2 ID3 982 214 0.7	*Injection Volume:*	1.0
Vial Number:	RB4	*Channel:*	UV_VIS_1
Sample Type:	unknown	*Wavelength:*	214
Control Program:	WXL-2014-1	*Bandwidth:*	n.a.
Quantif. Method:	WXL	*Dilution Factor:*	1.0000
Recording Time:	2014/6/9 12:20	*Sample Weight:*	1.0000
Run Time (min):	6.39	*Sample Amount:*	1.0000

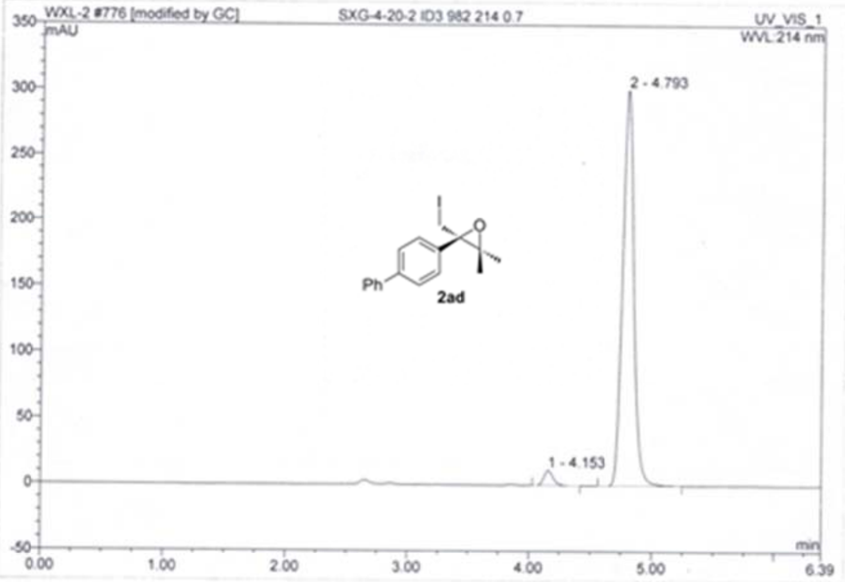

No.	Ret.Time min	Peak Name	Height mAU	Area mAU*min	Rel.Area %	Amount	Type
1	4.15	n.a.	11.532	0.995	3.04	n.a.	BMB*
2	4.79	n.a.	301.235	31.703	96.96	n.a.	BMB*
Total:			312.767	32.698	100.00	0.000	

Operator:Administrator Timebase:HPLC Sequence:201310-DAD

1870 SZG-3-75-2 PC-4 982 214 0.7

Sample Name:	SZG-3-75-2 PC-4 982 214 0.7	*Injection Volume:*	1.0
Vial Number:	RE4	*Channel:*	UV_VIS_2
Sample Type:	unknown	*Wavelength:*	214.0
Control Program:	test-dad2	*Bandwidth:*	4
Quantif. Method:	WXL	*Dilution Factor:*	1.0000
Recording Time:	2014-5-4 17:19	*Sample Weight:*	1.0000
Run Time (min):	25.00	*Sample Amount:*	1.0000

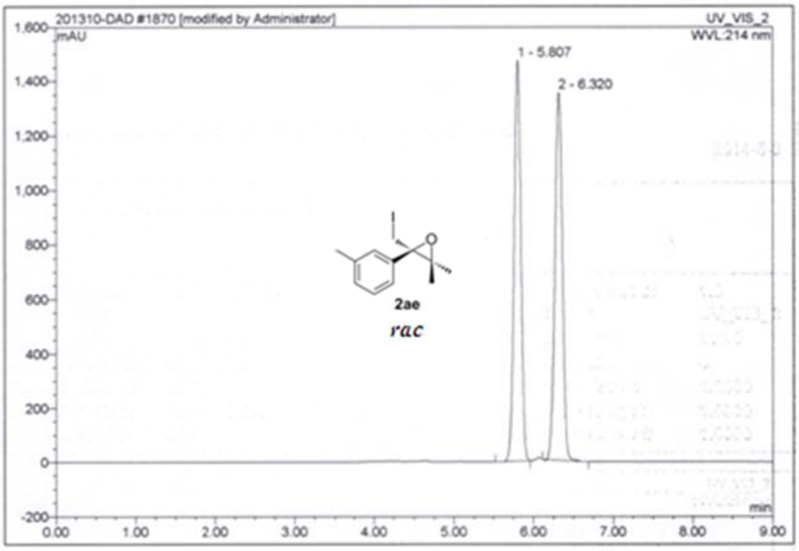

No.	Ret.Time min	Peak Name	Height mAU	Area mAU*min	Rel.Area %	Amount	Type
1	5.81	n.a.	1473.593	144.733	49.91	n.a.	BMB*
2	6.32	n.a.	1352.302	145.268	50.09	n.a.	BMB*
Total:			2825.896	290.001	100.00	0.000	

Operator:GC Timebase:U3000 Sequence:WXL-2

Page 1-
2014/5/27 8:58 上[illegible]

669 SZG-4-10-4 PC-4 982 214 0.7

Sample Name:	SZG-4-10-4 PC-4 982 214 0.7	*Injection Volume:*	1.0
Vial Number:	GE1	*Channel:*	UV_VIS_1
Sample Type:	unknown	*Wavelength:*	214
Control Program:	WXL-2014-1	*Bandwidth:*	n.a.
Quantif. Method:	WXL	*Dilution Factor:*	1.0000
Recording Time:	2014/5/26 19:10	*Sample Weight:*	1.0000
Run Time (min):	25.00	*Sample Amount:*	1.0000

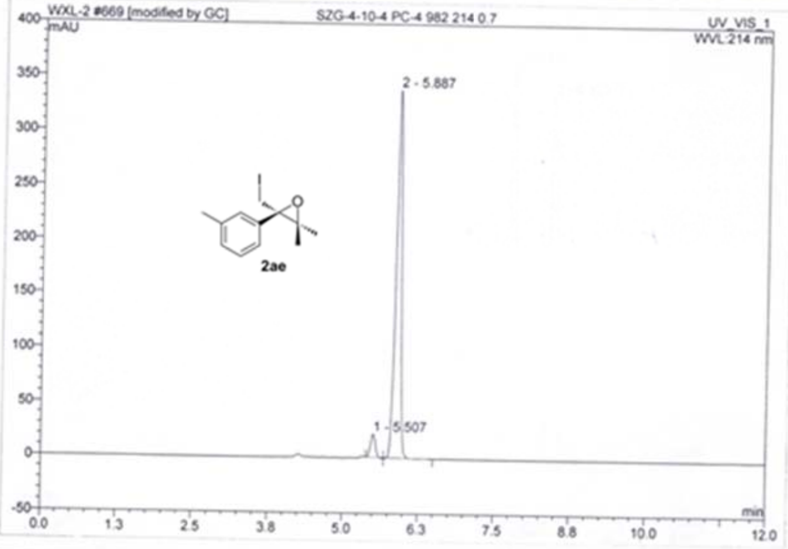

No.	Ret.Time min	Peak Name	Height mAU	Area mAU*min	Rel.Area %	Amount	Type
1	5.51	n.a.	21.627	1.932	5.25	n.a.	BM *
2	5.89	n.a.	339.386	34.870	94.75	n.a.	MB*
Total:			361.013	36.802	100.00	0.000	

2162 SZG-3-75-3+- OD-H 982 214 0.7

Sample Name:	SZG-3-75-3+- OD-H 982 214 0.7	*Injection Volume:*	8.0
Vial Number:	GE5	*Channel:*	UV_VIS_2
Sample Type:	unknown	*Wavelength:*	214.0
Control Program:	test-dad	*Bandwidth:*	4
Quantif. Method:	WXL	*Dilution Factor:*	1.0000
Recording Time:	2014-6-4 12:38	*Sample Weight:*	1.0000
Run Time (min):	16.26	*Sample Amount:*	1.0000

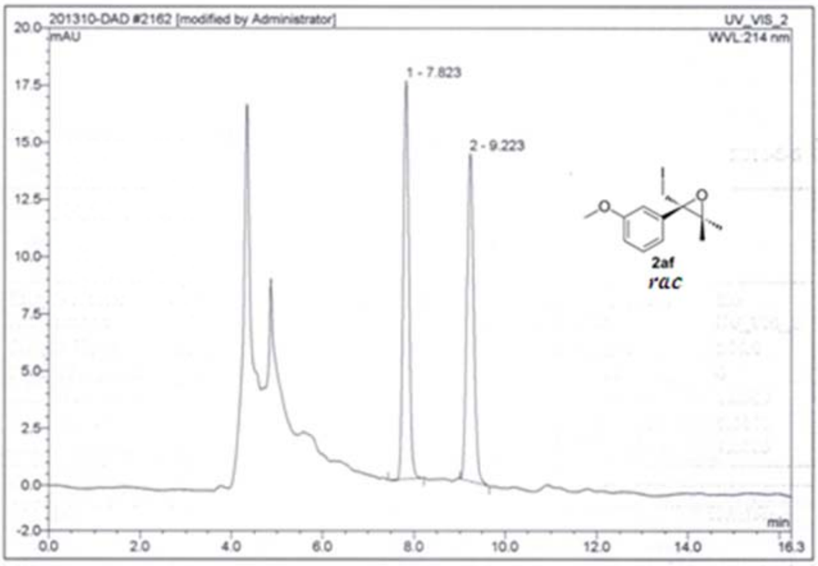

No.	Ret.Time min	Peak Name	Height mAU	Area mAU*min	Rel.Area %	Amount	Type
1	7.82	n.a.	17.433	2.495	49.99	n.a.	BMB*
2	9.22	n.a.	14.342	2.496	50.01	n.a.	BMB
Total:			31.775	4.991	100.00	0.000	

Operator:Administrator Timebase:HPLC Sequence:201310-DAD

Page
2014-6-5 9:48

2163 SZG-4-15-5 OD-H 982 214 0.7

Sample Name:	SZG-4-15-5 OD-H 982 214 0.7	*Injection Volume:*	1.0
Vial Number:	GE2	*Channel:*	UV_VIS_2
Sample Type:	unknown	*Wavelength:*	214.0
Control Program:	test-dad	*Bandwidth:*	4
Quantif. Method:	WXL	*Dilution Factor:*	1.0000
Recording Time:	2014-6-4 13:34	*Sample Weight:*	1.0000
Run Time (min):	13.47	*Sample Amount:*	1.0000

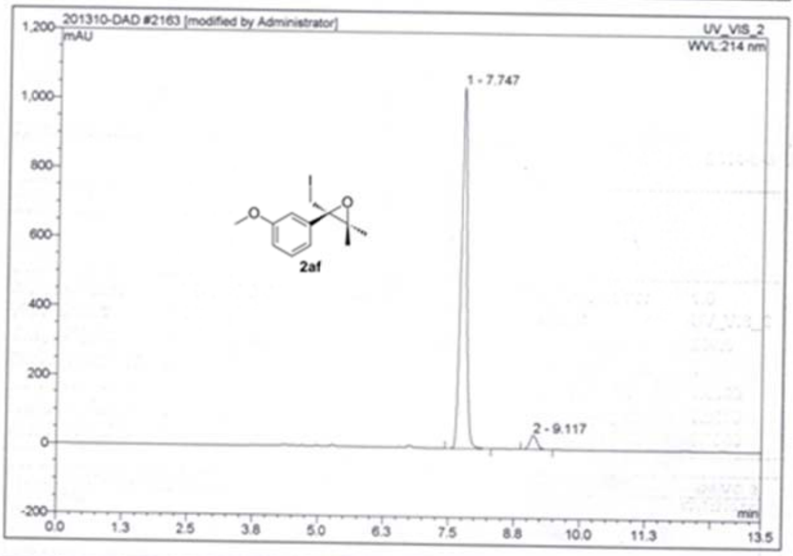

No.	Ret.Time min	Peak Name	Height mAU	Area mAU*min	Rel.Area %	Amount	Type
1	7.75	n.a.	1043.776	144.473	95.58	n.a.	BMB*
2	9.12	n.a.	40.516	6.680	4.42	n.a.	BMB
Total:			1084.292	151.153	100.00	0.000	

91%

Operator:GC Timebase:U3000 Sequence:WXL-2

Page 1-1
2014/6/5 9:58 上午

732 3-75-1+- AD-H 98515 214 0.4 18

Sample Name:	3-75-1+- AD-H 98515 214 0.4 18	*Injection Volume:*	1.0
Vial Number:	GA1	*Channel:*	UV_VIS_1
Sample Type:	unknown	*Wavelength:*	214
Control Program:	WXL-2014-1	*Bandwidth:*	n.a.
Quantif. Method:	WXL	*Dilution Factor:*	1.0000
Recording Time:	2014/6/4 11:12	*Sample Weight:*	1.0000
Run Time (min):	25.01	*Sample Amount:*	1.0000

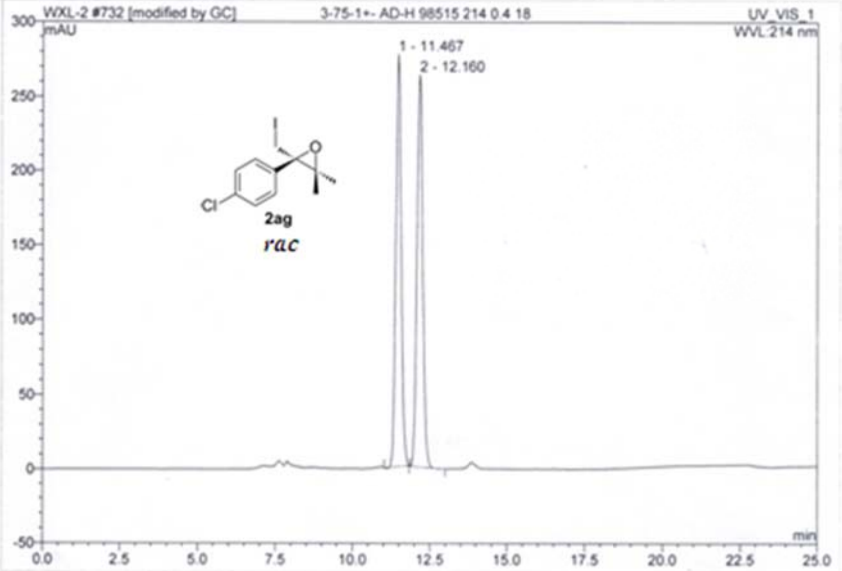

No.	Ret.Time min	Peak Name	Height mAU	Area mAU*min	Rel.Area %	Amount	Type
1	11.47	n.a.	276.589	56.874	49.90	n.a.	BMB*
2	12.16	n.a.	263.099	57.091	50.10	n.a.	BMB*
**Total:**			539.688	113.965	100.00	0.000	

Operator:GC Timebase:U3000 Sequence:WXL-2

Page 1-1
2014/6/5 9:58 上午

733 SZG-4-15-2 AD-H 98515 214 0.4 18

Sample Name:	SZG-4-15-2 AD-H 98515 214 0.4 18	Injection Volume:	1.0
Vial Number:	GA5	Channel:	UV_VIS_1
Sample Type:	unknown	Wavelength:	214
Control Program:	WXL-2014-1	Bandwidth:	n.a.
Quantif. Method:	WXL	Dilution Factor:	1.0000
Recording Time:	2014/6/4 12:04	Sample Weight:	1.0000
Run Time (min):	25.00	Sample Amount:	1.0000

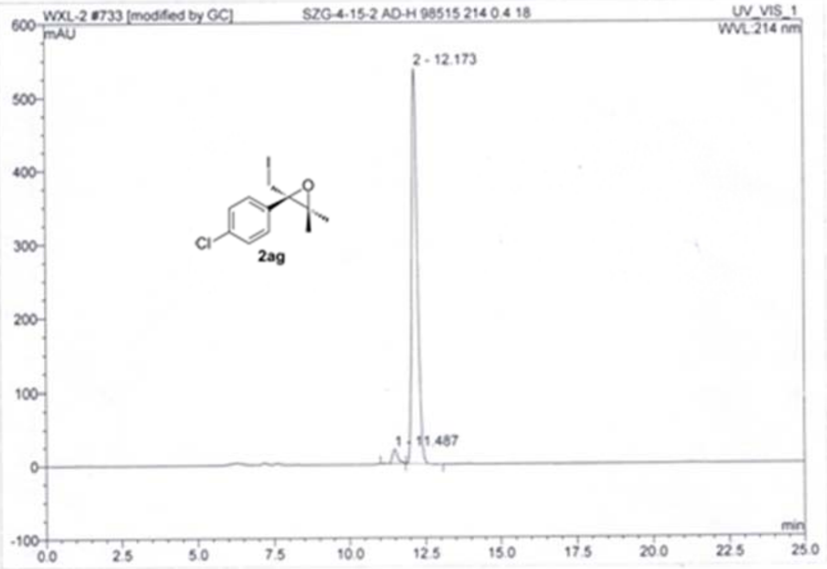

No.	Ret.Time min	Peak Name	Height mAU	Area mAU*min	Rel.Area %	Amount	Type
1	11.49	n.a.	19.735	4.111	3.48	n.a.	BMB*
2	12.17	n.a.	537.701	113.865	96.52	n.a.	BMB*
Total:			557.436	117.976	100.00	0.000	

93%

Operator:GC Timebase:U3000 Sequence:WXL-2

Page 1-1
2014/6/5 9:59 上午

736 3-88-5+- ID3 982 214 0.4 18

Sample Name:	3-88-5+- ID3 982 214 0.4 18	Injection Volume:	2.0
Vial Number:	GA3	Channel:	UV_VIS_1
Sample Type:	unknown	Wavelength:	214
Control Program:	WXL-2014-1	Bandwidth:	n.a.
Quantif. Method:	WXL	Dilution Factor:	1.0000
Recording Time:	2014/6/3 19:41	Sample Weight:	1.0000
Run Time (min):	25.00	Sample Amount:	1.0000

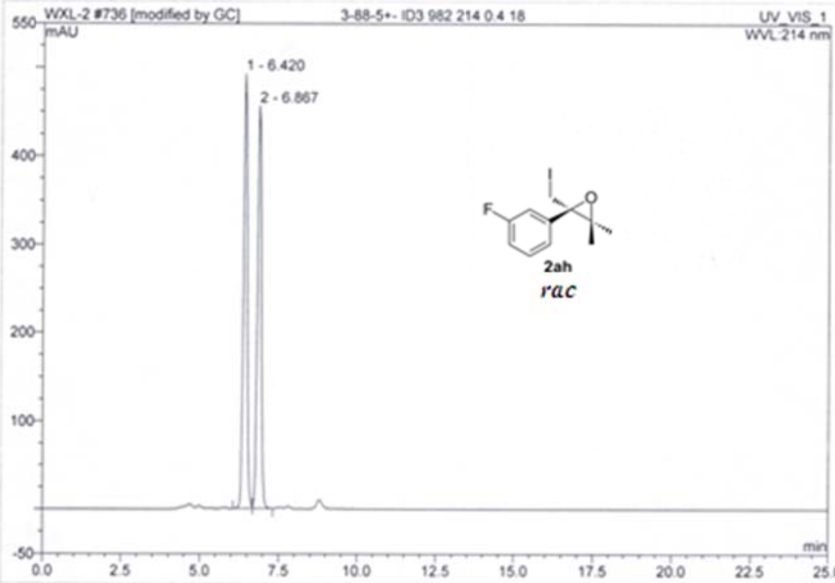

No.	Ret.Time min	Peak Name	Height mAU	Area mAU*min	Rel.Area %	Amount	Type
1	6.42	n.a.	492.835	68.150	50.73	n.a.	BM *
2	6.87	n.a.	456.294	66.197	49.27	n.a.	MB*
Total:			949.129	134.347	100.00	0.000	

Operator:GC Timebase:U3000 Sequence:WXL-2

Page 1-1
2014/6/5 10:00 上午

738 SZG-4-15-3 ID3 982 214 0.4 18

Sample Name:	**SZG-4-15-3 ID3 982 214 0.4 18**	*Injection Volume:*	**1.0**
Vial Number:	**GA5**	*Channel:*	**UV_VIS_1**
Sample Type:	**unknown**	*Wavelength:*	**214**
Control Program:	**WXL-2014-1**	*Bandwidth:*	**n.a.**
Quantif. Method:	**WXL**	*Dilution Factor:*	**1.0000**
Recording Time:	**2014/6/4 10:13**	*Sample Weight:*	**1.0000**
Run Time (min):	**12.19**	*Sample Amount:*	**1.0000**

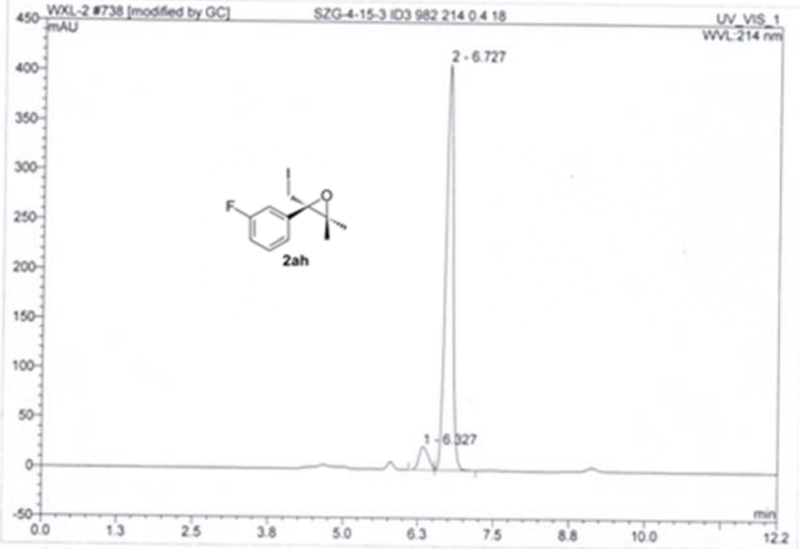

No.	Ret.Time min	Peak Name	Height mAU	Area mAU*min	Rel.Area %	Amount	Type
1	6.33	n.a.	23.487	4.228	6.84	n.a.	BM *
2	6.73	n.a.	410.099	57.544	93.16	n.a.	MB*
Total:			433.586	61.772	100.00	0.000	

Operator:Administrator Timebase:HPLC Sequence:201310-DAD

Page 1-1
2014-8-11 1:30 下午

2642 SZG-4-2-1+- IC 982 214 0.7

Sample Name:	SZG-4-2-1+- IC 982 214 0.7	*Injection Volume:*	1.0
Vial Number:	BD4	*Channel:*	UV_VIS_2
Sample Type:	unknown	*Wavelength:*	214.0
Control Program:	test-dad2	*Bandwidth:*	4
Quantif. Method:	WXL	*Dilution Factor:*	1.0000
Recording Time:	2014-8-11 9:13	*Sample Weight:*	1.0000
Run Time (min):	10.18	*Sample Amount:*	1.0000

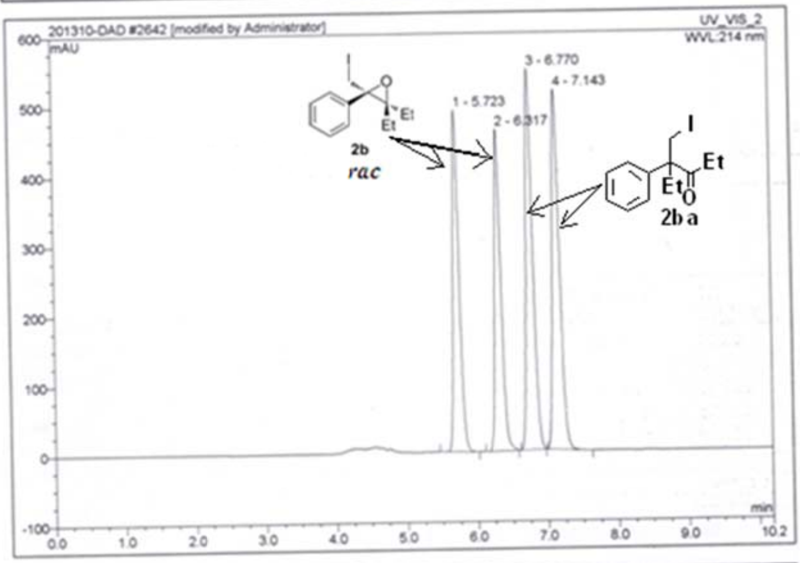

No.	Ret.Time min	Peak Name	Height mAU	Area mAU*min	Rel.Area %	Amount	Type
1	5.72	n.a.	490.068	49.723	22.29	n.a.	BMB*
2	6.32	n.a.	461.636	49.323	22.11	n.a.	BMB*
3	6.77	n.a.	546.193	61.573	27.60	n.a.	BMB*
4	7.14	n.a.	516.511	62.466	28.00	n.a.	BMB*
Total:			2014.407	223.086	100.00	0.000	

Operator:Administrator Timebase:HPLC Sequence:201310-DAD

2643 SZG-4-19-1 IC 982 214 0.7

Sample Name:	SZG-4-19-1 IC 982 214 0.7	*Injection Volume:*	1.0
Vial Number:	BD5	*Channel:*	UV_VIS_2
Sample Type:	unknown	*Wavelength:*	214.0
Control Program:	test-dad2	*Bandwidth:*	4
Quantif. Method:	WXL	*Dilution Factor:*	1.0000
Recording Time:	2014-8-11 8:52	*Sample Weight:*	1.0000
Run Time (min):	19.95	*Sample Amount:*	1.0000

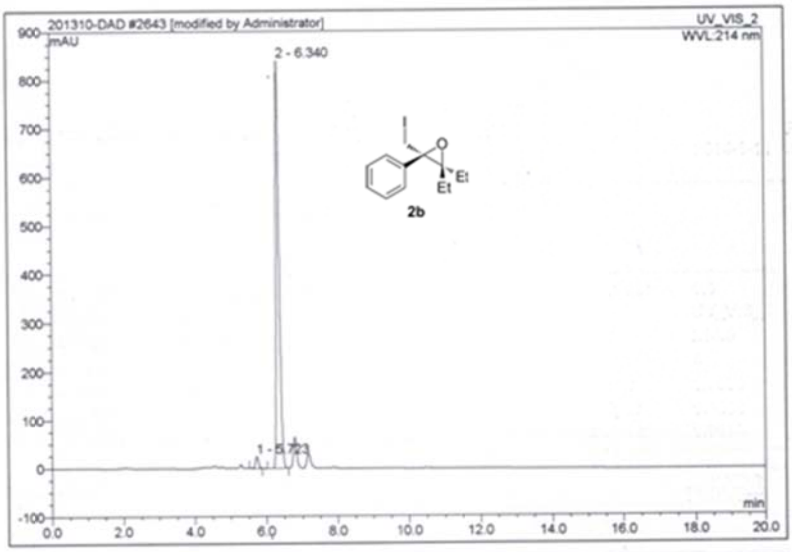

No.	Ret.Time min	Peak Name	Height mAU	Area mAU*min	Rel.Area %	Amount	Type
1	5.72	n.a.	24.099	2.350	2.54	n.a.	BMB*
2	6.34	n.a.	841.147	90.222	97.46	n.a.	BMB*
Total:			865.246	92.572	100.00	0.000	

Operator:GC Timebase:U3000 Sequence:WXL-2

Page 1-1
2014/7/4 7:24 下午

1031 SZG-4-5-1-1+- OD-H 982 214 0.7

Sample Name:	SZG-4-5-1-1+- OD-H 982 214 0.7	*Injection Volume:*	1.0
Vial Number:	GB2	*Channel:*	UV_VIS_1
Sample Type:	unknown	*Wavelength:*	214
Control Program:	WXL-2014-1	*Bandwidth:*	n.a.
Quantif. Method:	WXL	*Dilution Factor:*	1.0000
Recording Time:	2014/7/4 17:46	*Sample Weight:*	1.0000
Run Time (min):	23.11	*Sample Amount:*	1.0000

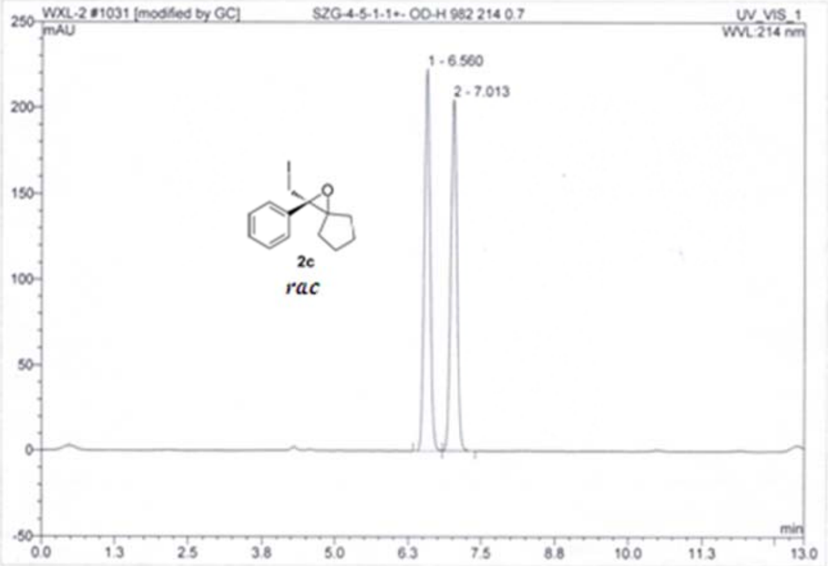

No.	Ret.Time min	Peak Name	Height mAU	Area mAU*min	Rel.Area %	Amount	Type
1	6.56	n.a.	223.051	25.621	50.10	n.a.	BM *
2	7.01	n.a.	205.056	25.516	49.90	n.a.	MB*
Total:			428.107	51.137	100.00	0.000	

Operator:Administrator Timebase:HPLC Sequence:201310-DAD

Page 1
2014-6-5 5:01 下

2189 SZG-4-19-3 OD-H 982 214 0.7

Sample Name:	SZG-4-19-3 OD-H 982 214 0.7	*Injection Volume:*	1.0
Vial Number:	GD3	*Channel:*	UV_VIS_2
Sample Type:	unknown	*Wavelength:*	214.0
Control Program:	test-dad	*Bandwidth:*	4
Quantif. Method:	WXL	*Dilution Factor:*	1.0000
Recording Time:	2014-6-5 14:30	*Sample Weight:*	1.0000
Run Time (min):	14.05	*Sample Amount:*	1.0000

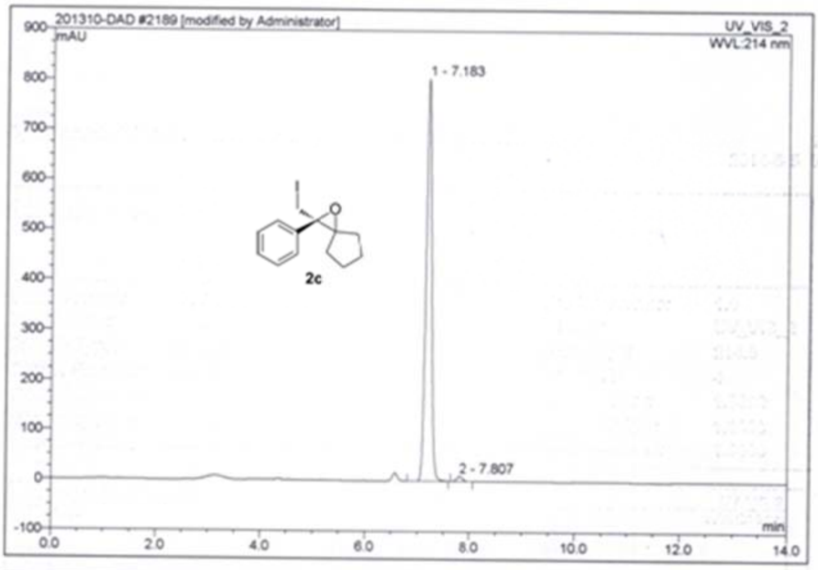

No.	Ret.Time min	Peak Name	Height mAU	Area mAU*min	Rel.Area %	Amount	Type
1	7.18	n.a.	805.280	99.745	98.69	n.a.	BMB*
2	7.81	n.a.	9.842	1.321	1.31	n.a.	BMB*
Total:			815.122	101.067	100.00	0.000	

1411 SZG-5-24 OD-H 982 214 0.7

Sample Name:	SZG-5-24 OD-H 982 214 0.7	Injection Volume:	1.0
Vial Number:	GD1	Channel:	UV_VIS_1
Sample Type:	unknown	Wavelength:	214
Control Program:	WXL-2014-2	Bandwidth:	n.a.
Quantif. Method:	WXL	Dilution Factor:	1.0000
Recording Time:	2014/9/3 9:06	Sample Weight:	1.0000
Run Time (min):	21.55	Sample Amount:	1.0000

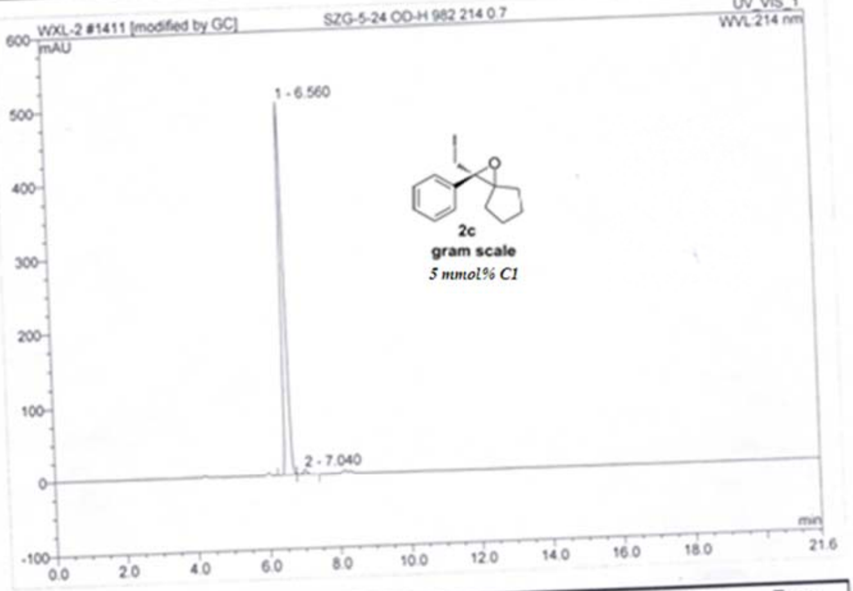

No.	Ret.Time min	Peak Name	Height mAU	Area mAU*min	Rel.Area %	Amount	Type
1	6.56	n.a.	504.943	58.458	98.80	n.a.	BMb*
2	7.04	n.a.	6.532	0.712	1.20	n.a.	bMB*
Total:			511.475	59.170	100.00	0.000	

Operator:Administrator Timebase:HPLC Sequence:201310-DAD

Page 1-1
2015-5-4 2:14 下午

5134 SZG-8-29-1 OD-H 982 214 0.7

Sample Name:	SZG-8-29-1 OD-H 982 214 0.7	*Injection Volume:*	3.0
Vial Number:	RD4	*Channel:*	UV_VIS_2
Sample Type:	unknown	*Wavelength:*	214.0
Control Program:	test-dad	*Bandwidth:*	4
Quantif. Method:	WXL	*Dilution Factor:*	1.0000
Recording Time:	2015-5-4 10:46	*Sample Weight:*	1.0000
Run Time (min):	14.01	*Sample Amount:*	1.0000

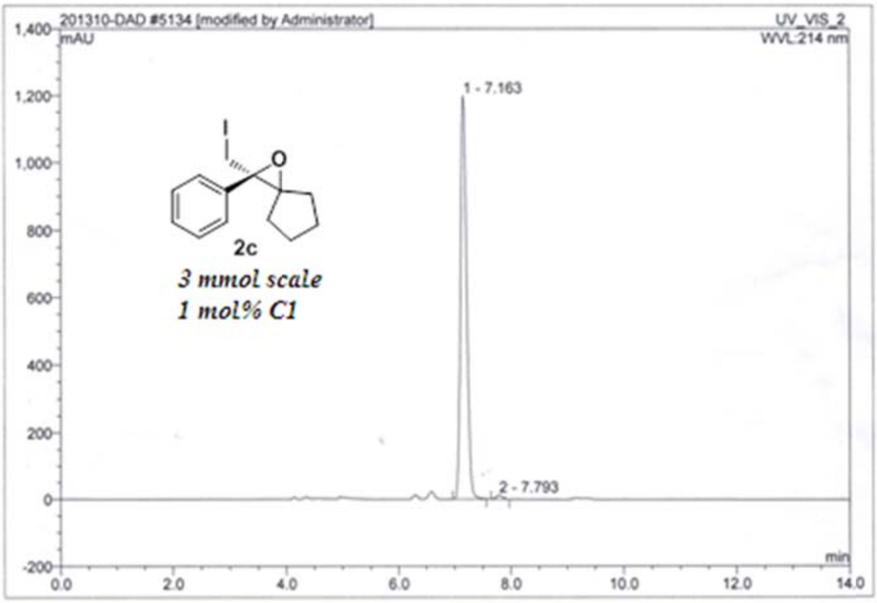

No.	Ret.Time min	Peak Name	Height mAU	Area mAU*min	Rel.Area %	Amount	Type
1	7.16	n.a.	1199.808	151.440	98.83	n.a.	BM *
2	7.79	n.a.	12.715	1.788	1.17	n.a.	M *
Total:			1212.523	153.228	100.00	0.000	

Operator:Administrator Timebase:HPLC Sequence:201310-DAD

Page 1-1
2014-6-27 4:04 下午

2356 SZG-4-39-3+- PC-4 955 214 0.7

Sample Name:	SZG-4-39-3+- PC-4 955 214 0.7	*Injection Volume:*	2.0
Vial Number:	GD3	*Channel:*	UV_VIS_2
Sample Type:	unknown	*Wavelength:*	214.0
Control Program:	test-dad2	*Bandwidth:*	4
Quantif. Method:	WXL	*Dilution Factor:*	1.0000
Recording Time:	2014-6-27 15:33	*Sample Weight:*	1.0000
Run Time (min):	11.82	*Sample Amount:*	1.0000

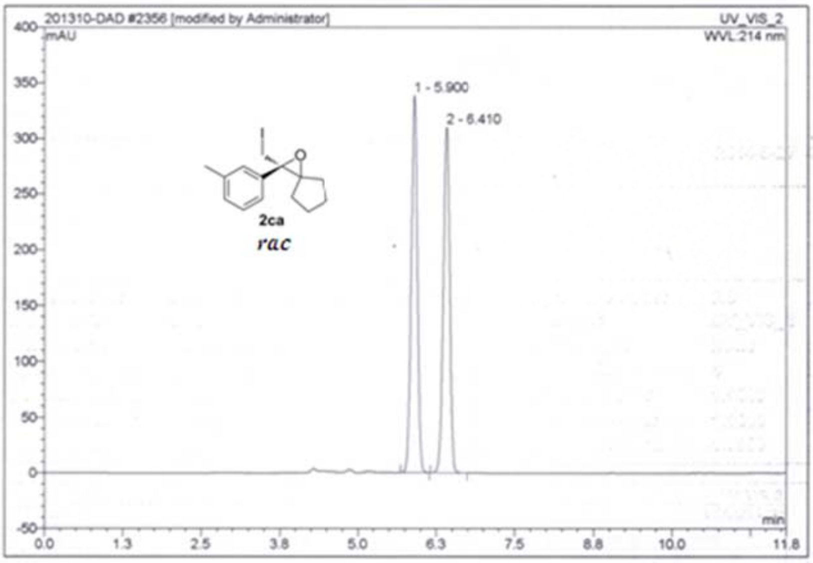

No.	Ret.Time min	Peak Name	Height mAU	Area mAU*min	Rel.Area %	Amount	Type
1	5.90	n.a.	338.631	38.413	51.36	n.a.	BMB*
2	6.41	n.a.	310.429	36.375	48.64	n.a.	BMB*
Total:			649.060	74.787	100.00	0.000	

2357 SZG-4-40-3 PC-4 955 214 0.7

Sample Name:	SZG-4-40-3 PC-4 955 214 0.7	*Injection Volume:*	2.0
Vial Number:	GD4	*Channel:*	UV_VIS_2
Sample Type:	unknown	*Wavelength:*	214.0
Control Program:	test-dad2	*Bandwidth:*	4
Quantif. Method:	WXL	*Dilution Factor:*	1.0000
Recording Time:	2014-6-27 15:46	*Sample Weight:*	1.0000
Run Time (min):	11.33	*Sample Amount:*	1.0000

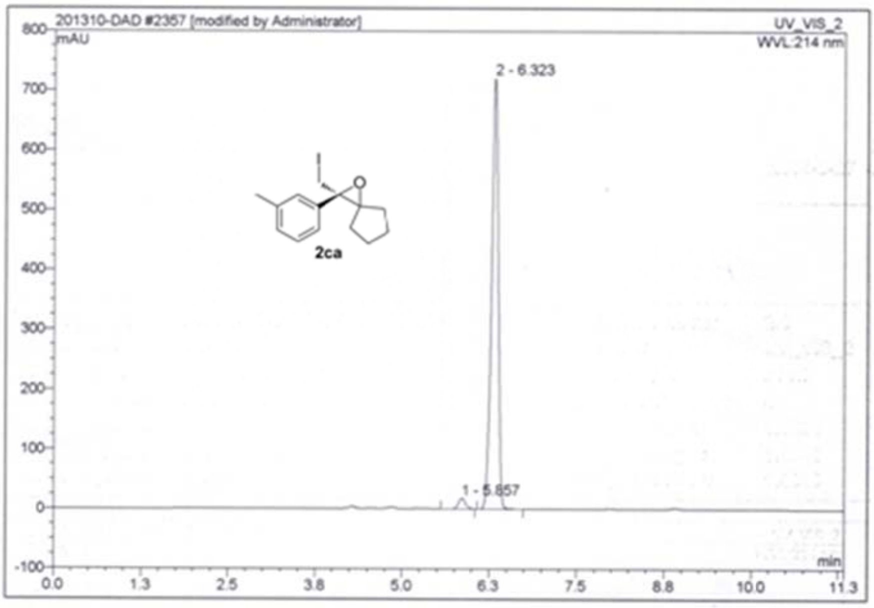

No.	Ret.Time min	Peak Name	Height mAU	Area mAU*min	Rel.Area %	Amount	Type
1	5.86	n.a.	17.726	2.107	2.56	n.a.	BMB*
2	6.32	n.a.	720.618	80.155	97.44	n.a.	BMB*
Total:			738.344	82.262	100.00	0.000	

Operator:Administrator Timebase:HPLC Sequence:201310-DAD

Page 1-1
2014-6-26 2:08 下午

2340 SZG-4-39-2+- PC-4 982 214 0.7

Sample Name:	SZG-4-39-2+- PC-4 982 214 0.7	*Injection Volume:*	1.0
Vial Number:	GD1	*Channel:*	UV_VIS_2
Sample Type:	unknown	*Wavelength:*	214.0
Control Program:	test-dad2	*Bandwidth:*	4
Quantif. Method:	WXL	*Dilution Factor:*	1.0000
Recording Time:	2014-6-26 11:10	*Sample Weight:*	1.0000
Run Time (min):	15.00	*Sample Amount:*	1.0000

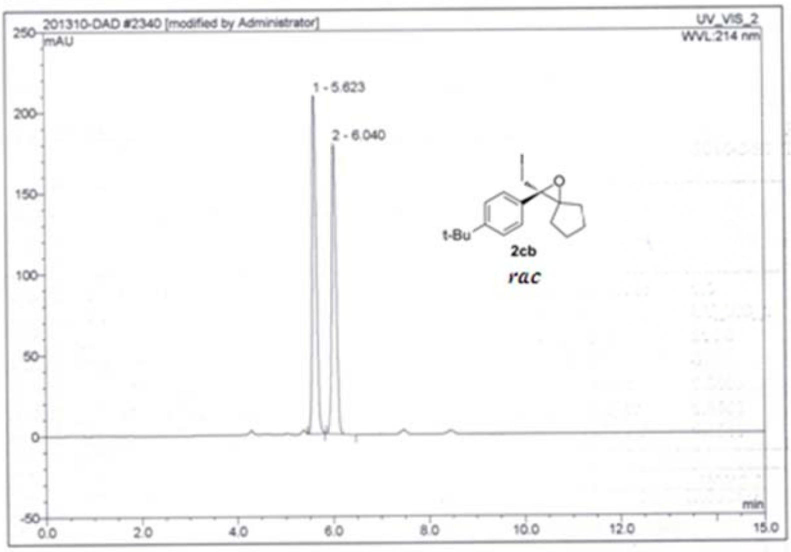

No.	Ret.Time min	Peak Name	Height mAU	Area mAU*min	Rel.Area %	Amount	Type
1	5.62	n.a.	209.253	20.455	51.65	n.a.	BMB*
2	6.04	n.a.	179.776	19.146	48.35	n.a.	BMB*
Total:			389.029	39.601	100.00	0.000	

Operator:Administrator Timebase:HPLC Sequence:201310-DAD

Page 1-1
2014-6-26 2:08 下午

2341 SZG-4-40-2 PC-4 982 214 0.7

Sample Name:	SZG-4-40-2 PC-4 982 214 0.7	*Injection Volume:*	1.0
Vial Number:	GD2	*Channel:*	UV_VIS_2
Sample Type:	unknown	*Wavelength:*	214.0
Control Program:	test-dad2	*Bandwidth:*	4
Quantif. Method:	WXL	*Dilution Factor:*	1.0000
Recording Time:	2014-6-26 11:26	*Sample Weight:*	1.0000
Run Time (min):	11.58	*Sample Amount:*	1.0000

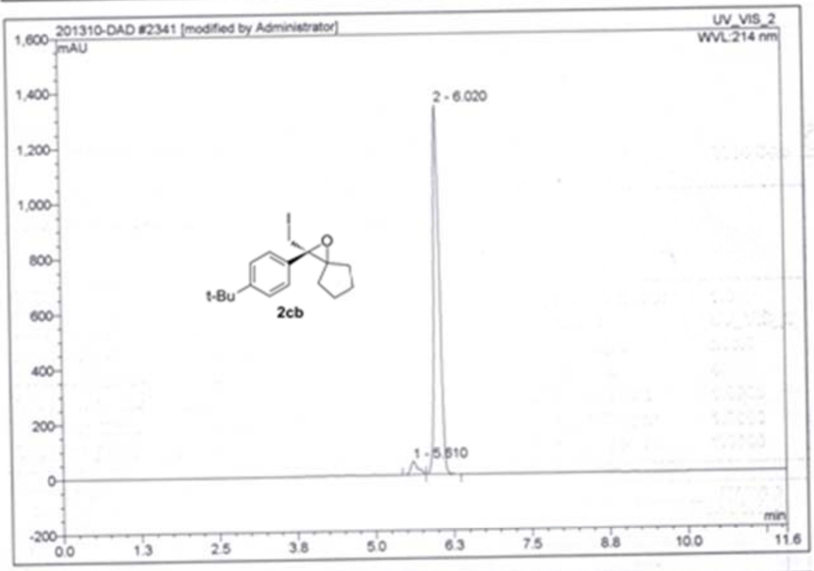

No.	Ret.Time min	Peak Name	Height mAU	Area mAU*min	Rel.Area %	Amount	Type
1	5.61	n.a.	47.390	6.116	4.07	n.a.	BMb*
2	6.02	n.a.	1337.188	144.196	95.93	n.a.	bMB*
Total:			1384.578	150.312	100.00	0.000	

Operator:Administrator Timebase:HPLC Sequence:201310-DAD

2014-6-30 2:18 下午

2366 SZG-4-50-5+- PC-4 982 214 0.7

Sample Name:	SZG-4-50-5+- PC-4 982 214 0.7	*Injection Volume:*	2.0
Vial Number:	GD5	*Channel:*	UV_VIS_2
Sample Type:	unknown	*Wavelength:*	214.0
Control Program:	test-dad2	*Bandwidth:*	4
Quantif. Method:	WXL	*Dilution Factor:*	1.0000
Recording Time:	2014-6-30 10:56	*Sample Weight:*	1.0000
Run Time (min):	12.00	*Sample Amount:*	1.0000

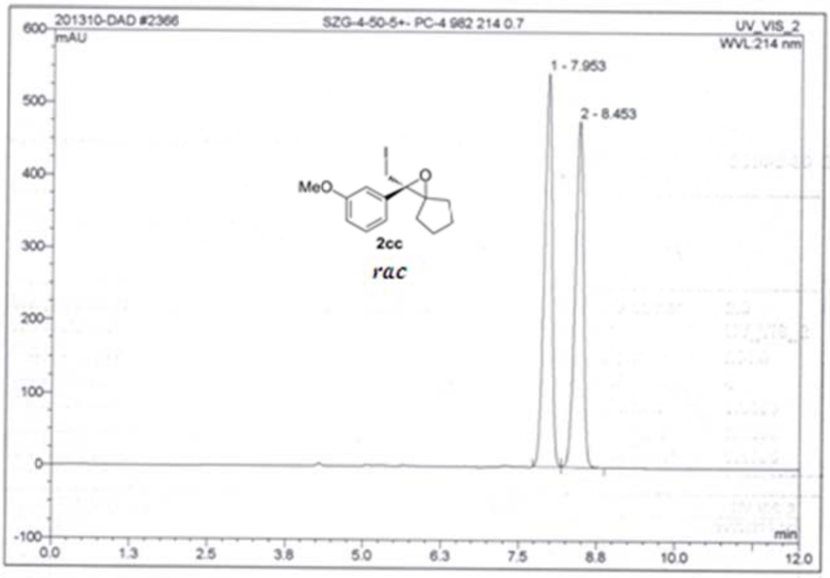

No.	Ret.Time min	Peak Name	Height mAU	Area mAU*min	Rel.Area %	Amount	Type
1	7.95	n.a.	542.318	77.625	51.33	n.a.	BM
2	8.45	n.a.	476.972	73.603	48.67	n.a.	MB
Total:			1019.289	151.228	100.00	0.000	

2367 SZG-4-85-5 PC-4 982 214 0.7

48

Sample Name:	SZG-4-85-5 PC-4 982 214 0.7	*Injection Volume:*	1.0
Vial Number:	GD6 48	*Channel:*	UV_VIS_2
Sample Type:	unknown	*Wavelength:*	214.0
Control Program:	test-dad2	*Bandwidth:*	4
Quantif. Method:	WXL	*Dilution Factor:*	1.0000
Recording Time:	2014-6-30 11:09	*Sample Weight:*	1.0000
Run Time (min):	12.00	*Sample Amount:*	1.0000

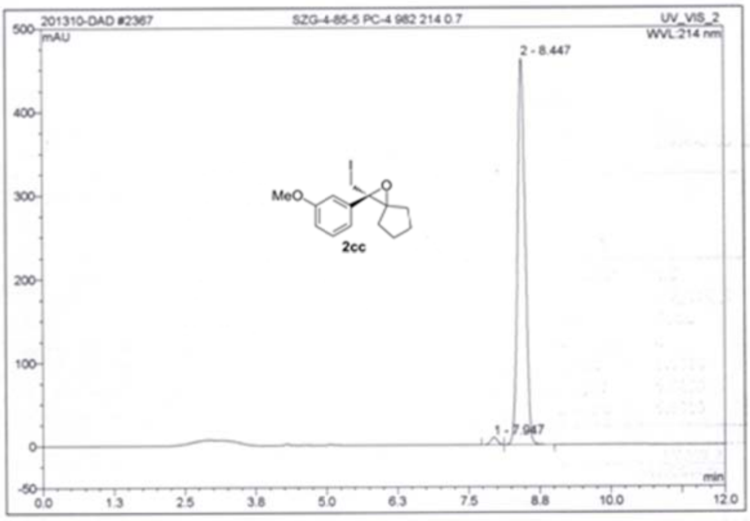

No.	Ret.Time min	Peak Name	Height mAU	Area mAU*min	Rel.Area %	Amount	Type
1	7.95	n.a.	9.002	1.282	1.76	n.a.	BM
2	8.45	n.a.	461.872	71.398	98.24	n.a.	MB
Total:			470.874	72.680	100.00	0.000	

Operator:Administrator Timebase:HPLC Sequence:201310-DAD

Page 1-1
2014-6-26 2:09 下午

2343 SZG-4-39-4+- PC-4 982 214 0.7

Sample Name:	SZG-4-39-4+- PC-4 982 214 0.7	*Injection Volume:*	1.0
Vial Number:	GD3	*Channel:*	UV_VIS_2
Sample Type:	unknown	*Wavelength:*	214.0
Control Program:	test-dad2	*Bandwidth:*	4
Quantif. Method:	WXL	*Dilution Factor:*	1.0000
Recording Time:	2014-6-26 11:40	*Sample Weight:*	1.0000
Run Time (min):	15.00	*Sample Amount:*	1.0000

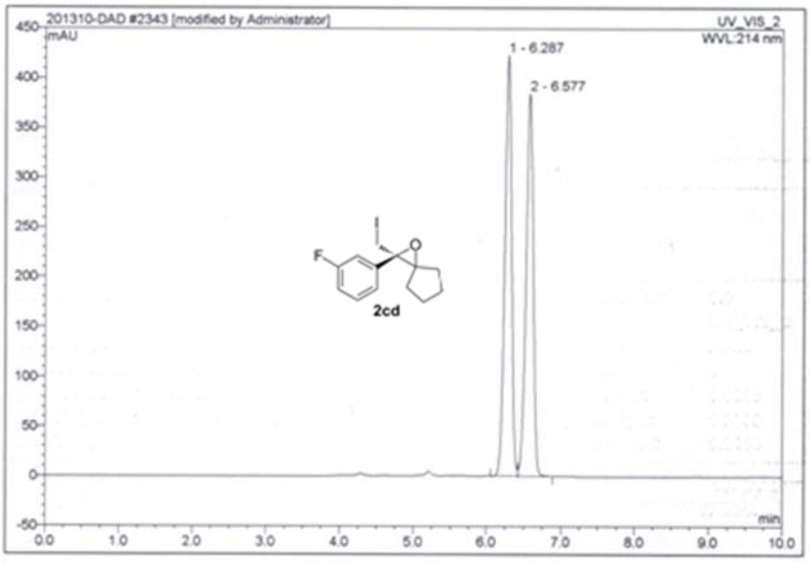

No.	Ret.Time min	Peak Name	Height mAU	Area mAU*min	Rel.Area %	Amount	Type
1	6.29	n.a.	422.662	44.806	50.90	n.a.	BM *
2	6.58	n.a.	384.469	43.228	49.10	n.a.	MB*
Total:			807.131	88.034	100.00	0.000	

Operator:Administrator Timebase:HPLC Sequence:201310-DAD

Page 1-1
2014-6-26 2:10 下午

2344 SZG-4-40-4 PC-4 982 214 0.7

Sample Name:	SZG-4-40-4 PC-4 982 214 0.7	*Injection Volume:*	1.0
Vial Number:	GD4	*Channel:*	UV_VIS_2
Sample Type:	unknown	*Wavelength:*	214.0
Control Program:	test-dad2	*Bandwidth:*	4
Quantif. Method:	WXL	*Dilution Factor:*	1.0000
Recording Time:	2014-6-26 12:12	*Sample Weight:*	1.0000
Run Time (min):	15.00	*Sample Amount:*	1.0000

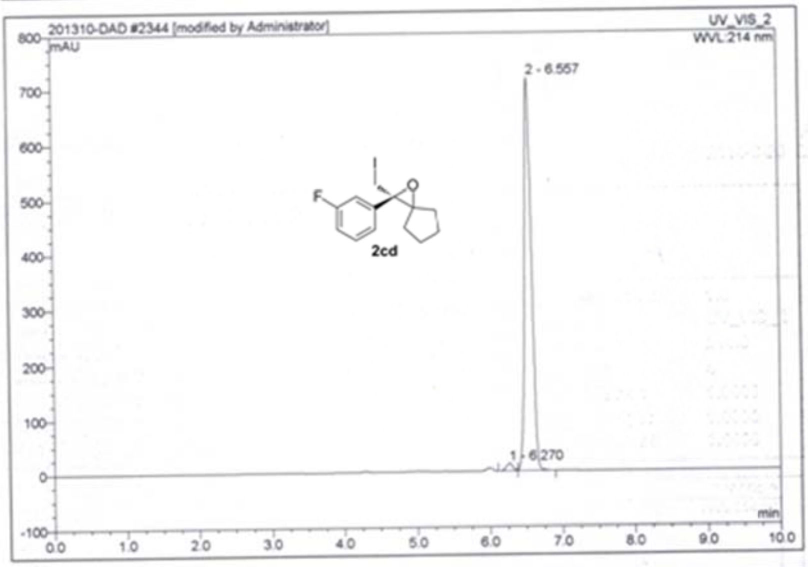

No.	Ret.Time min	Peak Name	Height mAU	Area mAU*min	Rel.Area %	Amount	Type
1	6.27	n.a.	13.988	1.448	1.77	n.a.	BM *
2	6.56	n.a.	716.116	80.334	98.23	n.a.	MB*
Total:			730.104	81.782	100.00	0.000	

Operator:Administrator Timebase:HPLC Sequence:201310-DAD

Page 1-
2014-6-17 10:19 上午

2274 SZG-4-26-2+- PA-2 982 214 0.7

Sample Name:	SZG-4-26-2+- PA-2 982 214 0.7	*Injection Volume:*	3.0
Vial Number:	GD1	*Channel:*	UV_VIS_2
Sample Type:	unknown	*Wavelength:*	214.0
Control Program:	test-dad	*Bandwidth:*	4
Quantif. Method:	WXL	*Dilution Factor:*	1.0000
Recording Time:	2014-6-16 14:25	*Sample Weight:*	1.0000
Run Time (min):	13.44	*Sample Amount:*	1.0000

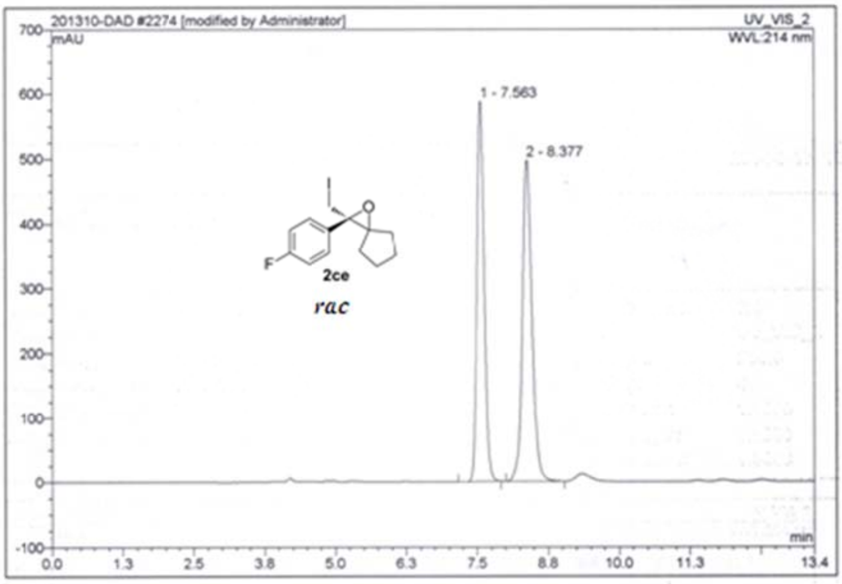

No.	Ret.Time min	Peak Name	Height mAU	Area mAU*min	Rel.Area %	Amount	Type
1	7.56	n.a.	586.983	87.337	47.45	n.a.	BMB*
2	8.38	n.a.	496.722	96.711	52.55	n.a.	BMB*
Total:			1083.705	184.048	100.00	0.000	

2275 SZG-4-27-2 PA-2 982 214 0.7

Sample Name:	SZG-4-27-2 PA-2 982 214 0.7	*Injection Volume:*	1.0
Vial Number:	GD2	*Channel:*	UV_VIS_2
Sample Type:	unknown	*Wavelength:*	214.0
Control Program:	test-dad	*Bandwidth:*	4
Quantif. Method:	WXL	*Dilution Factor:*	1.0000
Recording Time:	2014-6-16 14:55	*Sample Weight:*	1.0000
Run Time (min):	12.44	*Sample Amount:*	1.0000

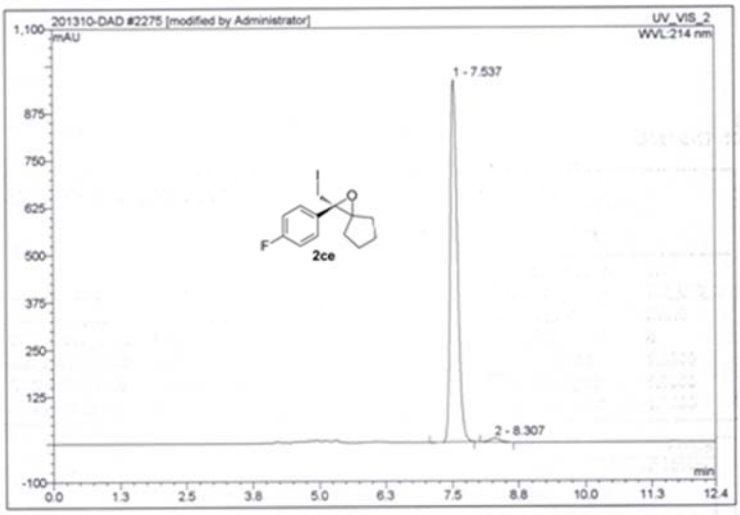

No.	Ret.Time min	Peak Name	Height mAU	Area mAU*min	Rel.Area %	Amount	Type
1	7.54	n.a.	958.690	148.033	98.27	n.a.	BMB*
2	8.31	n.a.	11.152	2.603	1.73	n.a.	BMB
Total:			969.841	150.635	100.00	0.000	

2276 SZG-4-26-1+- PA-2 982 214 0.7

Sample Name:	SZG-4-26-1+- PA-2 982 214 0.7	*Injection Volume:*	1.0
Vial Number:	GD3	*Channel:*	UV_VIS_2
Sample Type:	unknown	*Wavelength:*	214.0
Control Program:	test-dad	*Bandwidth:*	4
Quantif. Method:	WXL	*Dilution Factor:*	1.0000
Recording Time:	2014-6-16 15:09	*Sample Weight:*	1.0000
Run Time (min):	15.00	*Sample Amount:*	1.0000

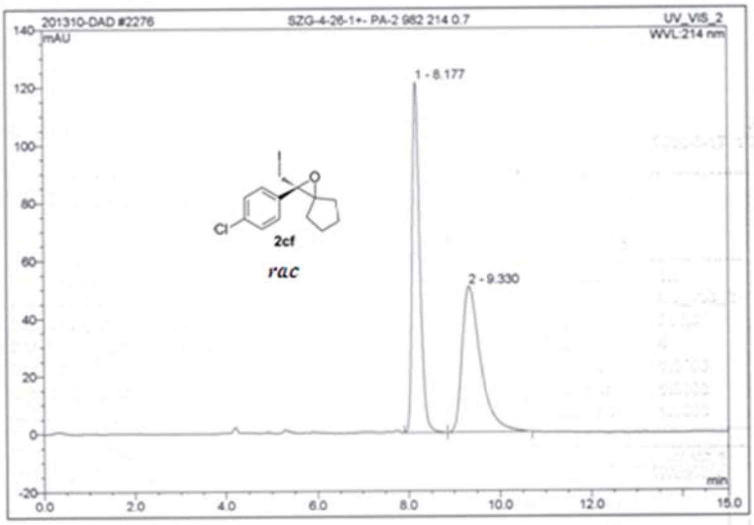

No.	Ret.Time min	Peak Name	Height mAU	Area mAU*min	Rel.Area %	Amount	Type
1	8.18	n.a.	121.196	23.238	49.37	n.a.	BM
2	9.33	n.a.	50.555	23.833	50.63	n.a.	MB
**Total:**			171.750	47.071	100.00	0.000	

Operator:Administrator Timebase:HPLC Sequence:201310-DAD 2014-6-17 10:[illegible]

2277 SZG-4-27-1 PA-2 982 214 0.7

Sample Name:	SZG-4-27-1 PA-2 982 214 0.7	*Injection Volume:*	1.0
Vial Number:	GD4	*Channel:*	UV_VIS_2
Sample Type:	unknown	*Wavelength:*	214.0
Control Program:	test-dad	*Bandwidth:*	4
Quantif. Method:	WXL	*Dilution Factor:*	1.0000
Recording Time:	2014-6-16 15:25	*Sample Weight:*	1.0000
Run Time (min):	14.48	*Sample Amount:*	1.0000

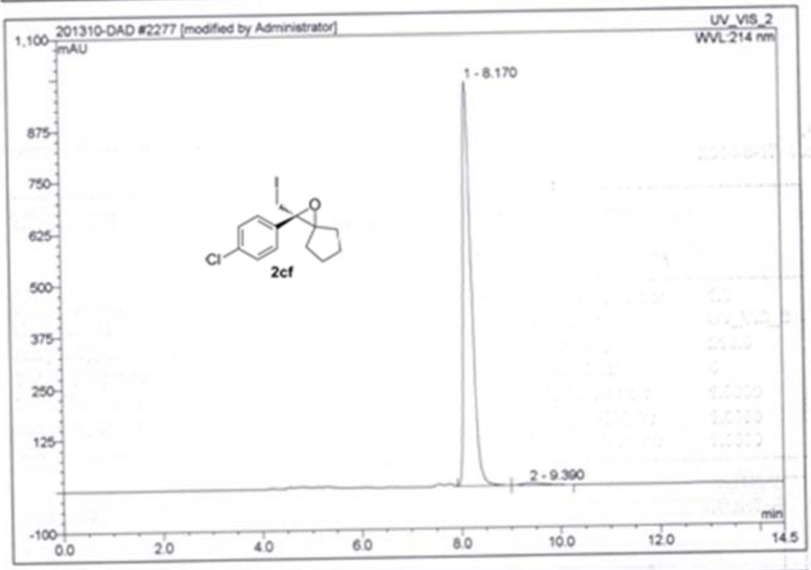

No.	Ret.Time min	Peak Name	Height mAU	Area mAU*min	Rel.Area %	Amount	Type
1	8.17	n.a.	981.711	189.818	98.82	n.a.	BMB
2	9.39	n.a.	4.713	2.261	1.18	n.a.	BMB
Total:			986.425	192.078	100.00	0.000	

Operator:Administrator Timebase:HPLC Sequence:201310-DAD

Page 1-1
2014-6-30 2:16 下午

2362 SZG-4-50-2-1+- PC-4 982 214 0.7

Sample Name:	SZG-4-50-2-1+- PC-4 982 214 0.7	*Injection Volume:*	2.0
Vial Number:	GD1	*Channel:*	UV_VIS_2
Sample Type:	unknown	*Wavelength:*	214.0
Control Program:	test-dad2	*Bandwidth:*	4
Quantif. Method:	WXL	*Dilution Factor:*	1.0000
Recording Time:	2014-6-30 10:05	*Sample Weight:*	1.0000
Run Time (min):	12.00	*Sample Amount:*	1.0000

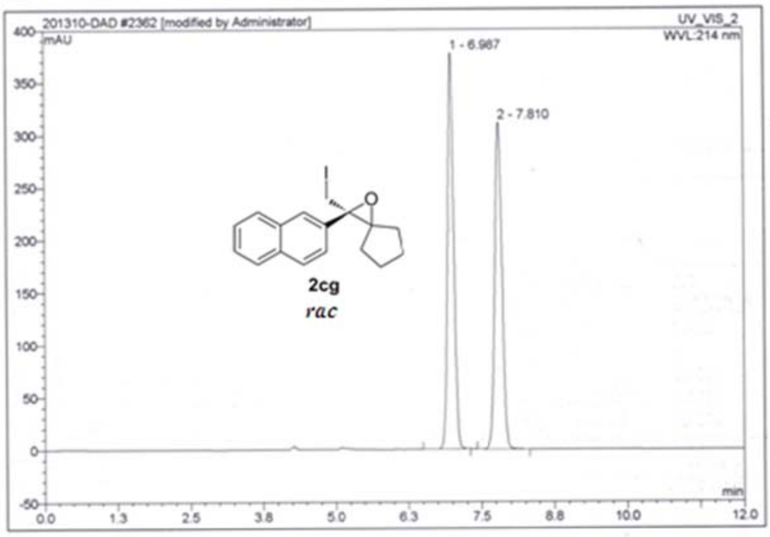

No.	Ret.Time min	Peak Name	Height mAU	Area mAU*min	Rel.Area %	Amount	Type
1	6.99	n.a.	377.565	47.152	51.43	n.a.	BMB*
2	7.81	n.a.	311.745	44.523	48.57	n.a.	BMB*
Total:			689.311	91.675	100.00	0.000	

Operator:Administrator Timebase:HPLC Sequence:201310-DAD

Page 1
2014-6-30 2:16 下

2363 SZG-4-48-2-1 PC-4 982 214 0.7

Sample Name:	SZG-4-48-2-1 PC-4 982 214 0.7	Injection Volume:	2.0
Vial Number:	GD2	Channel:	UV_VIS_2
Sample Type:	unknown	Wavelength:	214.0
Control Program:	test-dad2	Bandwidth:	4
Quantif. Method:	WXL	Dilution Factor:	1.0000
Recording Time:	2014-6-30 10:18	Sample Weight:	1.0000
Run Time (min):	12.00	Sample Amount:	1.0000

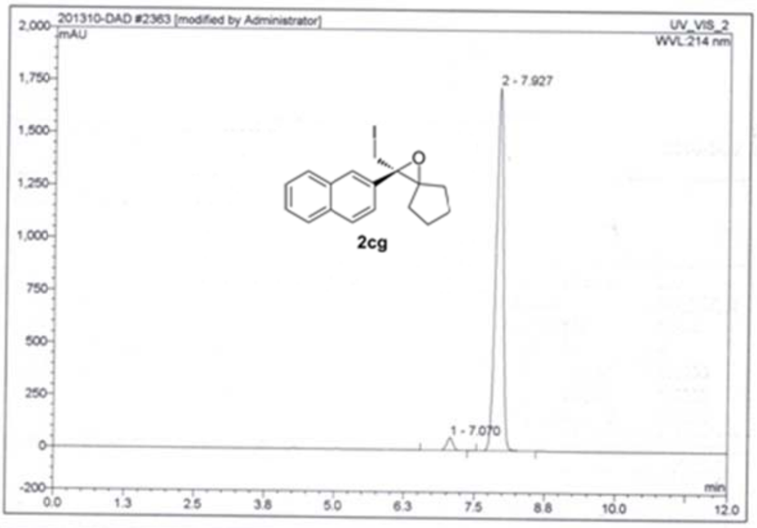

No.	Ret.Time min	Peak Name	Height mAU	Area mAU*min	Rel.Area %	Amount	Type
1	7.07	n.a.	60.426	7.759	2.91	n.a.	BMB*
2	7.93	n.a.	1728.793	258.881	97.09	n.a.	BMB*
Total:			1789.219	266.640	100.00	0.000	

2364 SZG-4-50-3+- PC-4 982 214 0.7

Sample Name:	SZG-4-50-3+- PC-4 982 214 0.7	*Injection Volume:*	2.0
Vial Number:	GD3	*Channel:*	UV_VIS_2
Sample Type:	unknown	*Wavelength:*	214.0
Control Program:	test-dad2	*Bandwidth:*	4
Quantif. Method:	WXL	*Dilution Factor:*	1.0000
Recording Time:	2014-6-30 10:32	*Sample Weight:*	1.0000
Run Time (min):	10.41	*Sample Amount:*	1.0000

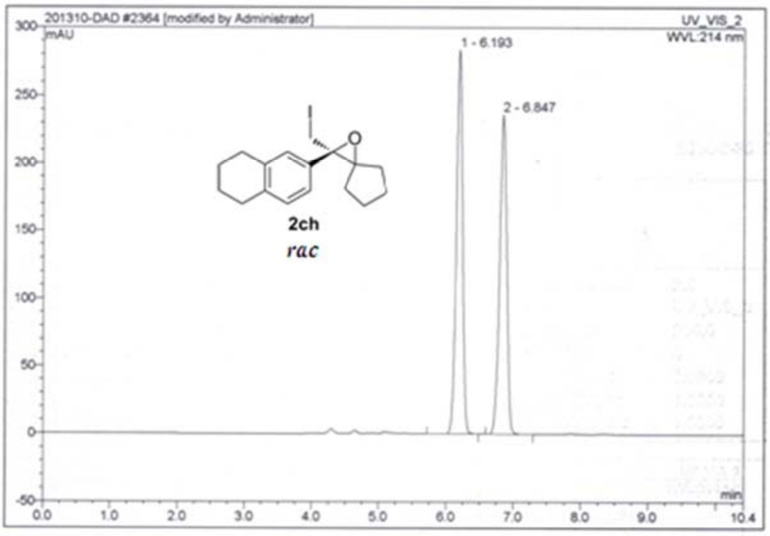

No.	Ret.Time min	Peak Name	Height mAU	Area mAU*min	Rel.Area %	Amount	Type
1	6.19	n.a.	283.671	30.700	51.40	n.a.	BMB*
2	6.85	n.a.	236.691	29.023	48.60	n.a.	BMB*
Total:			520.361	59.722	100.00	0.000	

Operator:Administrator Timebase:HPLC Sequence:201310-DAD

2014-6-30 2:17 下午

2365 SZG-4-48-3 PC-4 982 214 0.7

Sample Name:	SZG-4-48-3 PC-4 982 214 0.7	*Injection Volume:*	2.0
Vial Number:	GD4	*Channel:*	UV_VIS_2
Sample Type:	unknown	*Wavelength:*	214.0
Control Program:	test-dad2	*Bandwidth:*	4
Quantif. Method:	WXL	*Dilution Factor:*	1.0000
Recording Time:	2014-6-30 10:45	*Sample Weight:*	1.0000
Run Time (min):	9.56	*Sample Amount:*	1.0000

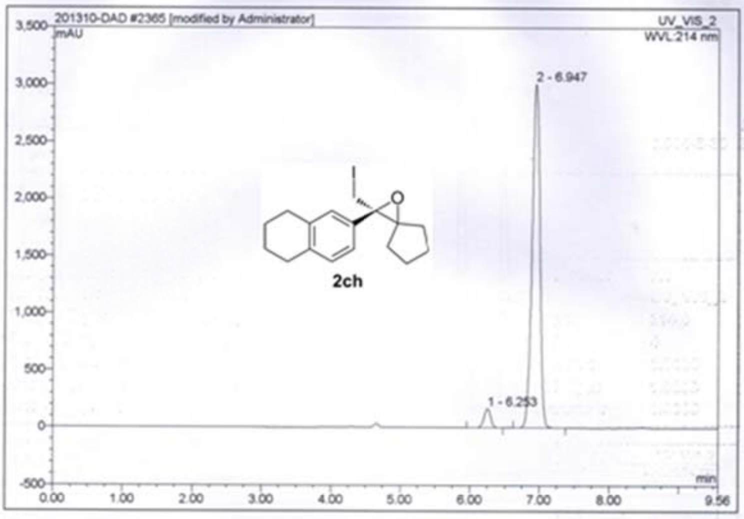

No.	Ret.Time min	Peak Name	Height mAU	Area mAU*min	Rel.Area %	Amount	Type
1	6.25	n.a.	169.273	18.676	4.47	n.a.	BMB*
2	6.95	n.a.	3008.430	399.031	95.53	n.a.	BMB*
Total:			3177.703	417.707	100.00	0.000	

Operator:Administrator Timebase:HPLC Sequence:201310-DAD

Page 1-
2014-6-5 4:59 下午

2191 SZG-3-79-2+- OD-H 982 214 0.7

Sample Name:	SZG-3-79-2+- OD-H 982 214 0.7	*Injection Volume:*	1.0
Vial Number:	GD2	*Channel:*	UV_VIS_2
Sample Type:	unknown	*Wavelength:*	214.0
Control Program:	test-dad	*Bandwidth:*	4
Quantif. Method:	WXL	*Dilution Factor:*	1.0000
Recording Time:	2014-6-5 14:46	*Sample Weight:*	1.0000
Run Time (min):	12.54	*Sample Amount:*	1.0000

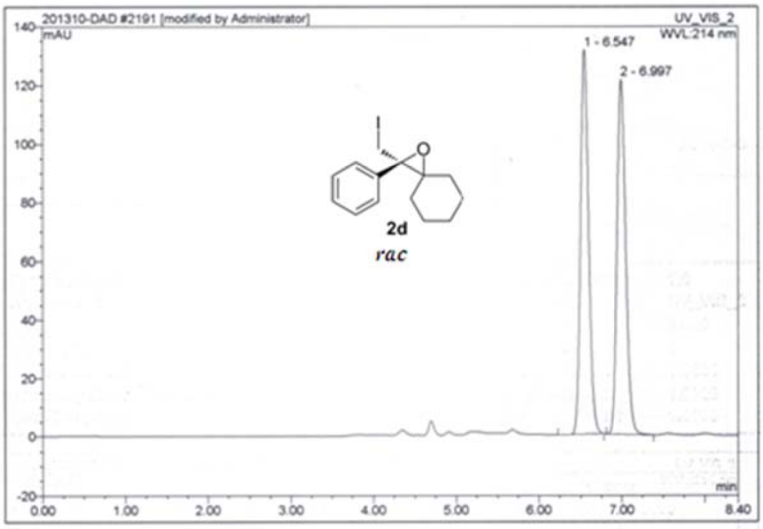

No.	Ret.Time min	Peak Name	Height mAU	Area mAU*min	Rel.Area %	Amount	Type
1	6.55	n.a.	131.402	14.799	49.94	n.a.	BMB*
2	7.00	n.a.	121.251	14.833	50.06	n.a.	BMB*
Total:			252.653	29.632	100.00	0.000	

2192 SZG-4-19-4 OD-H 982 214 0.7

Sample Name:	SZG-4-19-4 OD-H 982 214 0.7	*Injection Volume:*	1.0
Vial Number:	GD1	*Channel:*	UV_VIS_2
Sample Type:	unknown	*Wavelength:*	214.0
Control Program:	test-dad	*Bandwidth:*	4
Quantif. Method:	WXL	*Dilution Factor:*	1.0000
Recording Time:	2014-6-5 15:00	*Sample Weight:*	1.0000
Run Time (min):	16.62	*Sample Amount:*	1.0000

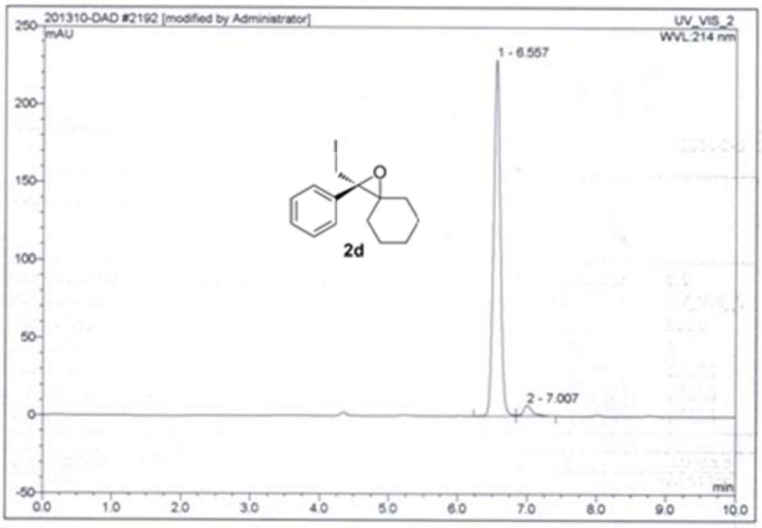

No.	Ret.Time min	Peak Name	Height mAU	Area mAU*min	Rel.Area %	Amount	Type
1	6.56	n.a.	229.120	25.943	95.91	n.a.	BM *
2	7.01	n.a.	7.268	1.107	4.09	n.a.	MB*
Total:			236.388	27.049	100.00	0.000	

Operator:GC Timebase:U3000 Sequence:WXL-2

Page 1
2014/8/11 1:42 下

1241 SZG-4-86 OD-H 982 214 0.7

Sample Name:	**SZG-4-86 OD-H 982 214 0.7**	*Injection Volume:*	**1.0**
Vial Number:	**GC7**	*Channel:*	**UV_VIS_1**
Sample Type:	**unknown**	*Wavelength:*	**214**
Control Program:	**WXL-2014-2**	*Bandwidth:*	**n.a.**
Quantif. Method:	**WXL**	*Dilution Factor:*	**1.0000**
Recording Time:	**2014/8/11 11:29**	*Sample Weight:*	**1.0000**
Run Time (min):	**11.97**	*Sample Amount:*	**1.0000**

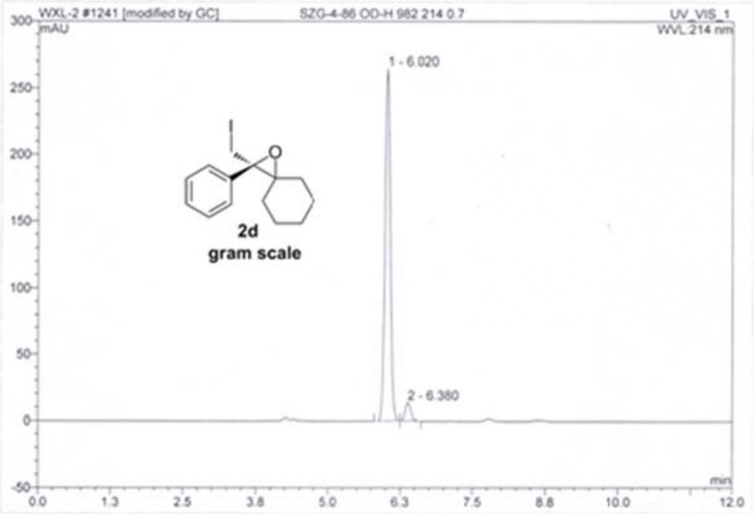

No.	Ret.Time min	Peak Name	Height mAU	Area mAU*min	Rel.Area %	Amount	Type
1	6.02	n.a.	264.667	27.960	94.93	n.a.	BMb*
2	6.38	n.a.	13.428	1.495	5.07	n.a.	bMB*
Total:			278.095	29.454	100.00	0.000	

Operator:Administrator Timebase:HPLC Sequence:201310-DAD

Page 1-1
2014-5-13 3:37 下午

1932 SZG-4-2-2+- IC 955 214 0.7

Sample Name:	SZG-4-2-2+- IC 955 214 0.7	Injection Volume:	1.0
Vial Number:	RC6	Channel:	UV_VIS_2
Sample Type:	unknown	Wavelength:	214.0
Control Program:	test-dad2	Bandwidth:	4
Quantif. Method:	WXL	Dilution Factor:	1.0000
Recording Time:	2014-5-13 11:16	Sample Weight:	1.0000
Run Time (min):	12.00	Sample Amount:	1.0000

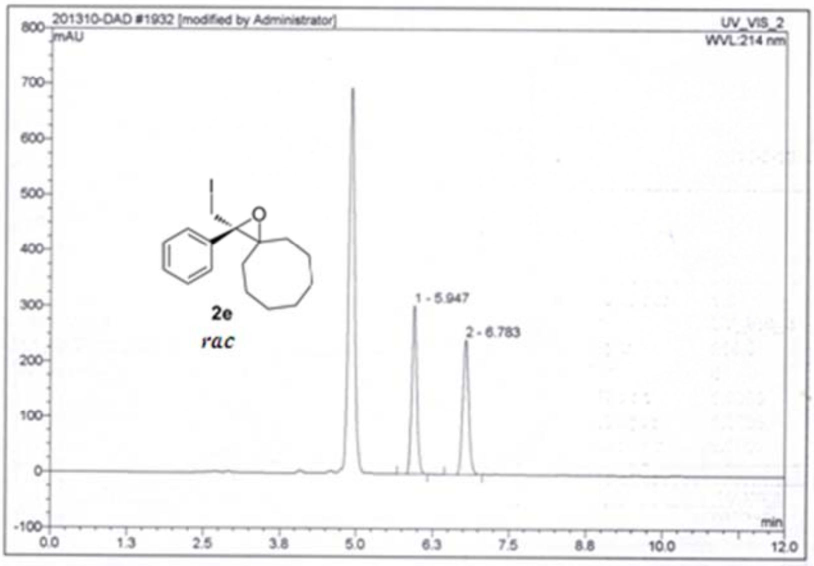

No.	Ret.Time min	Peak Name	Height mAU	Area mAU*min	Rel.Area %	Amount	Type
1	5.95	n.a.	304.751	28.867	51.64	n.a.	BMB*
2	6.78	n.a.	244.028	27.035	48.36	n.a.	BMB*
Total:			548.779	55.902	100.00	0.000	

2194 SZG-4-19-5 IC 955 214 0.7

Sample Name:	SZG-4-19-5 IC 955 214 0.7	Injection Volume:	0.8
Vial Number:	GD1	Channel:	UV_VIS_2
Sample Type:	unknown	Wavelength:	214.0
Control Program:	test-dad	Bandwidth:	4
Quantif. Method:	WXL	Dilution Factor:	1.0000
Recording Time:	2014-6-5 15:49	Sample Weight:	1.0000
Run Time (min):	10.40	Sample Amount:	1.0000

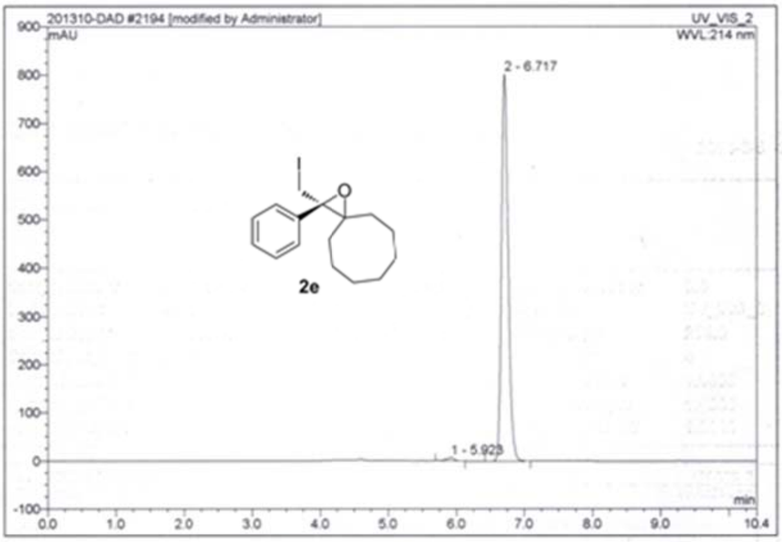

No.	Ret.Time min	Peak Name	Height mAU	Area mAU*min	Rel.Area %	Amount	Type
1	5.92	n.a.	7.144	0.860	0.94	n.a.	BMB*
2	6.72	n.a.	802.576	90.544	99.06	n.a.	BMB*
Total:			809.720	91.404	100.00	0.000	

Operator:Administrator Timebase:HPLC Sequence:201810-DAD

Page 1-1
2014-8-11 1:31 下午

2648 SZG-4-87 IC 955 214 0.7

Sample Name:	SZG-4-87 IC 955 214 0.7	*Injection Volume:*	1.0
Vial Number:	BE3	*Channel:*	UV_VIS_2
Sample Type:	unknown	*Wavelength:*	214.0
Control Program:	test-dad2	*Bandwidth:*	4
Quantif. Method:	WXL	*Dilution Factor:*	1.0000
Recording Time:	2014-8-11 11:15	*Sample Weight:*	1.0000
Run Time (min):	12.00	*Sample Amount:*	1.0000

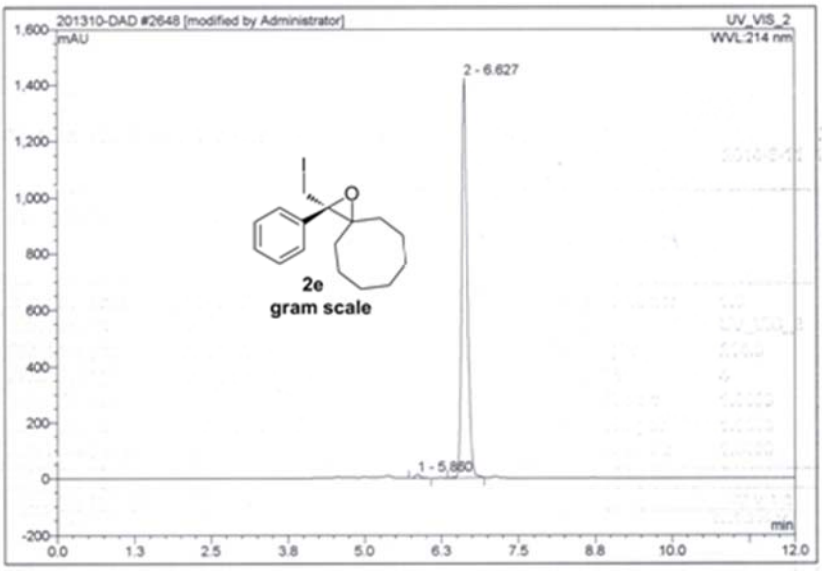

No.	Ret.Time min	Peak Name	Height mAU	Area mAU*min	Rel.Area %	Amount	Type
1	5.86	n.a.	12.885	1.238	0.77	n.a.	BMB*
2	6.63	n.a.	1423.477	159.318	99.23	n.a.	BMB*
Total:			1436.362	160.556	100.00	0.000	

Operator:Administrator Timebase:HPLC Sequence:201310-DAD

Page 1-1
2014-5-27 9:00 上午

2066 SZG-3-100-2+- AD-H 98515 214 0.7

Sample Name:	SZG-3-100-2+- AD-H 98515 214 0.7	*Injection Volume:*	5.0
Vial Number:	GE4	*Channel:*	UV_VIS_2
Sample Type:	unknown	*Wavelength:*	214.0
Control Program:	test-dad2	*Bandwidth:*	4
Quantif. Method:	WXL	*Dilution Factor:*	1.0000
Recording Time:	2014-5-26 15:55	*Sample Weight:*	1.0000
Run Time (min):	30.88	*Sample Amount:*	1.0000

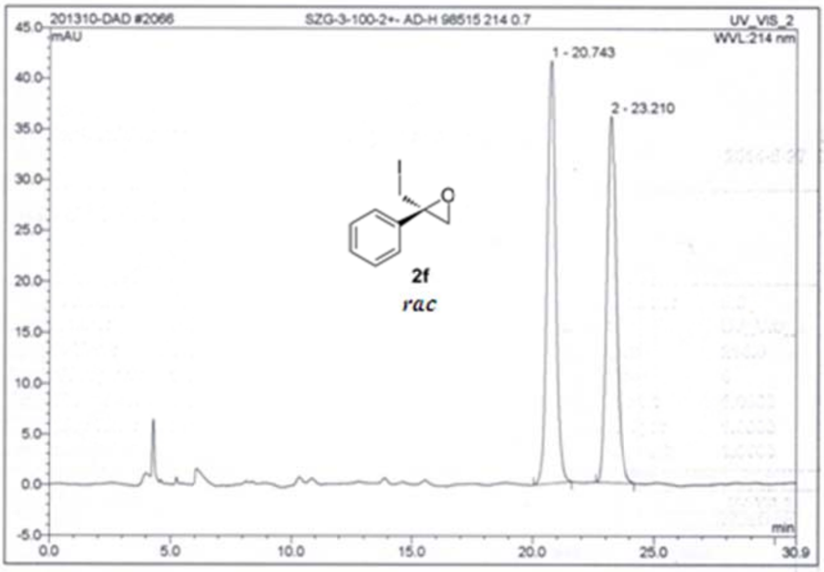

No.	Ret.Time min	Peak Name	Height mAU	Area mAU*min	Rel.Area %	Amount	Type
1	20.74	n.a.	41.701	17.237	50.52	n.a.	BMB
2	23.21	n.a.	36.116	16.880	49.48	n.a.	BMB
Total:			77.817	34.117	100.00	0.000	

Operator:Administrator Timebase:HPLC Sequence:201310-DAD

Page 1-1
2014-5-27 9:00 上午

2067 SZG-4-14-3 AD-H 98515 214 0.7

Sample Name:	SZG-4-14-3 AD-H 98515 214 0.7	*Injection Volume:*	5.0
Vial Number:	GE5	*Channel:*	UV_VIS_2
Sample Type:	unknown	*Wavelength:*	214.0
Control Program:	test-dad2	*Bandwidth:*	4
Quantif. Method:	WXL	*Dilution Factor:*	1.0000
Recording Time:	2014-5-26 16:27	*Sample Weight:*	1.0000
Run Time (min):	28.67	*Sample Amount:*	1.0000

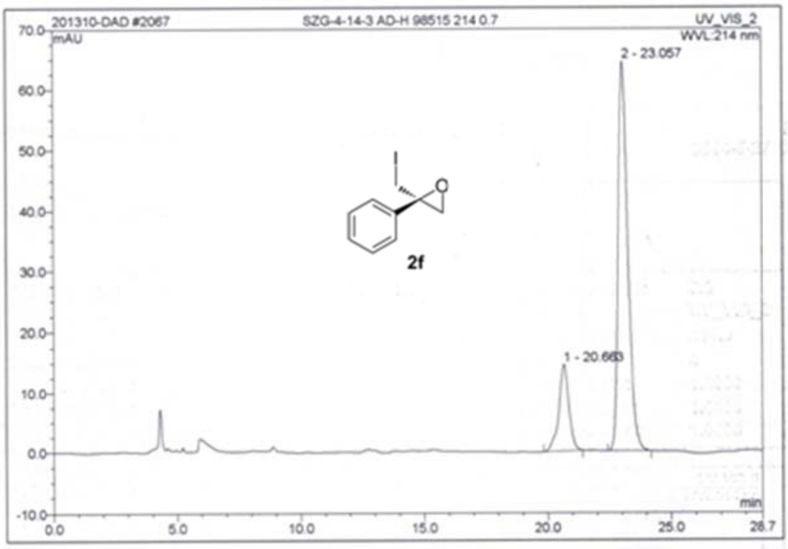

No.	Ret.Time min	Peak Name	Height mAU	Area mAU*min	Rel.Area %	Amount	Type
1	20.66	n.a.	14.487	6.759	18.33	n.a.	BMB
2	23.06	n.a.	64.431	30.110	81.67	n.a.	BMB
Total:			78.918	36.869	100.00	0.000	

1893 SZG-3-95-3+- AD-H 982 214 0.7

Sample Name:	SZG-3-95-3+- AD-H 982 214 0.7	*Injection Volume:*	2.0
Vial Number:	RA1	*Channel:*	UV_VIS_2
Sample Type:	unknown	*Wavelength:*	214.0
Control Program:	test-dad2	*Bandwidth:*	4
Quantif. Method:	WXL	*Dilution Factor:*	1.0000
Recording Time:	2014-5-6 15:32	*Sample Weight:*	1.0000
Run Time (min):	11.56	*Sample Amount:*	1.0000

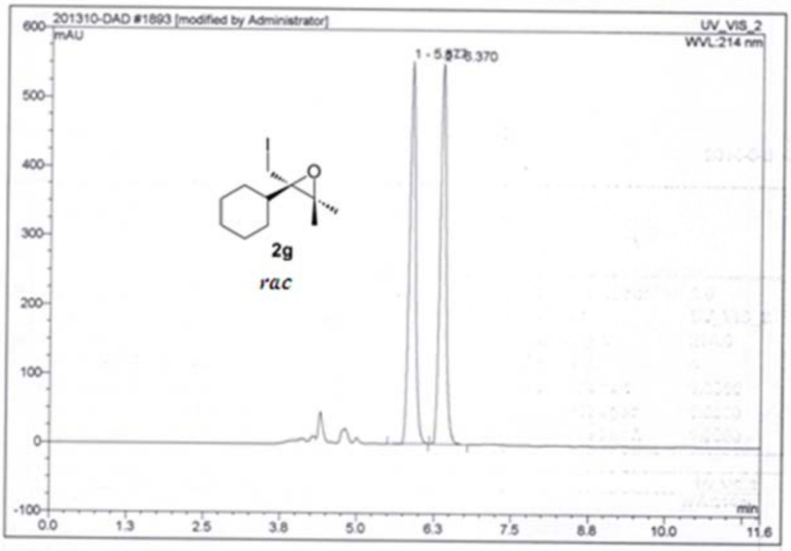

No.	Ret.Time min	Peak Name	Height mAU	Area mAU*min	Rel.Area %	Amount	Type
1	5.88	n.a.	552.706	63.362	50.09	n.a.	BMB*
2	6.37	n.a.	549.805	63.141	49.91	n.a.	BMB*
Total:			1102.511	126.503	100.00	0.000	

Operator:Administrator Timebase:HPLC Sequence:201310-DAD

Page 1
2014-5-6 4:06 下

1894 SZG-3-95-1 AD-H 982 214 0.7

Sample Name:	SZG-3-95-1 AD-H 982 214 0.7	*Injection Volume:*	1.0
Vial Number:	RA2	*Channel:*	UV_VIS_2
Sample Type:	unknown	*Wavelength:*	214.0
Control Program:	test-dad2	*Bandwidth:*	4
Quantif. Method:	WXL	*Dilution Factor:*	1.0000
Recording Time:	2014-5-6 15:51	*Sample Weight:*	1.0000
Run Time (min):	14.77	*Sample Amount:*	1.0000

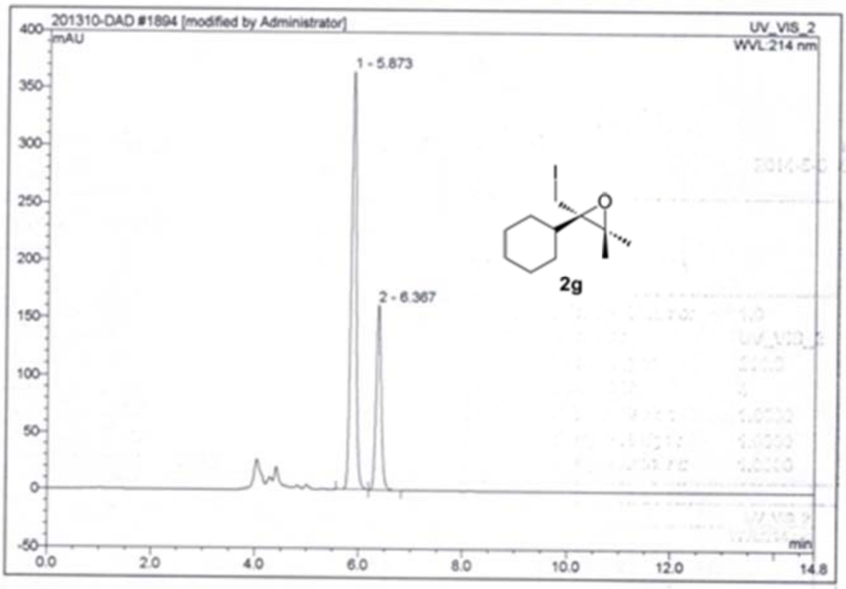

No.	Ret.Time min	Peak Name	Height mAU	Area mAU*min	Rel.Area %	Amount	Type
1	5.87	n.a.	364.621	40.126	68.56	n.a.	BMb*
2	6.37	n.a.	161.802	18.400	31.44	n.a.	bMB*
Total:			526.423	58.525	100.00	0.000	

Operator:Administrator Timebase:HPLC Sequence:201310-DAD

Page 1-1
2014-7-3 11:49 上午

2397 SZG-4-51+- PC-4 982 214 0.7

Sample Name:	SZG-4-51+- PC-4 982 214 0.7	*Injection Volume:*	0.8
Vial Number:	GD3	*Channel:*	UV_VIS_2
Sample Type:	unknown	*Wavelength:*	214.0
Control Program:	test-dad2	*Bandwidth:*	4
Quantif. Method:	WXL	*Dilution Factor:*	1.0000
Recording Time:	2014-7-3 11:34	*Sample Weight:*	1.0000
Run Time (min):	14.49	*Sample Amount:*	1.0000

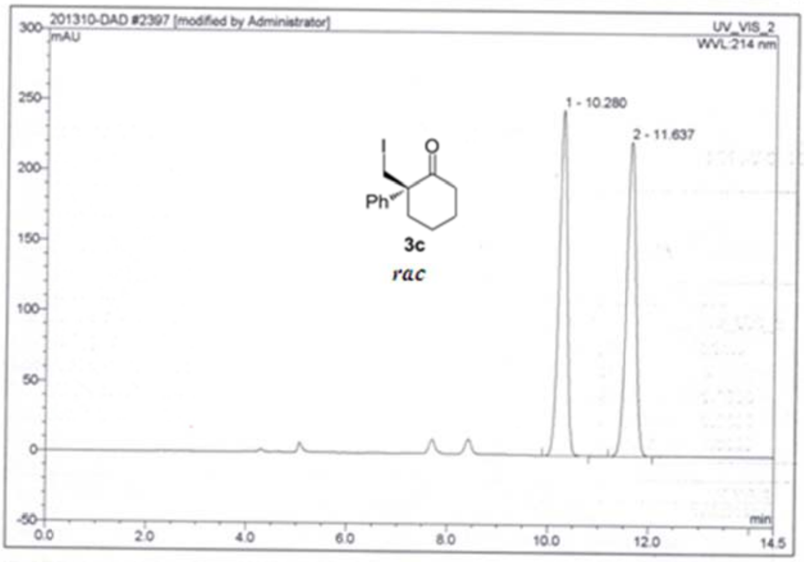

No.	Ret.Time min	Peak Name	Height mAU	Area mAU*min	Rel.Area %	Amount	Type
1	10.28	n.a.	245.628	46.823	48.67	n.a.	BMB
2	11.64	n.a.	223.466	49.373	51.33	n.a.	BMB
Total:			469.094	96.196	100.00	0.000	

Operator:Administrator Timebase:HPLC Sequence:201310-DAD

Page 1-1
2014-9-5 5:03 下午

2842 SZG-5-30 PC-4 982 214 0.7

Sample Name:	SZG-5-30 PC-4 982 214 0.7	*Injection Volume:*	1.0
Vial Number:	GA2	*Channel:*	UV_VIS_2
Sample Type:	unknown	*Wavelength:*	214.0
Control Program:	test-dad2	*Bandwidth:*	4
Quantif. Method:	WXL	*Dilution Factor:*	1.0000
Recording Time:	2014-9-5 15:37	*Sample Weight:*	1.0000
Run Time (min):	20.77	*Sample Amount:*	1.0000

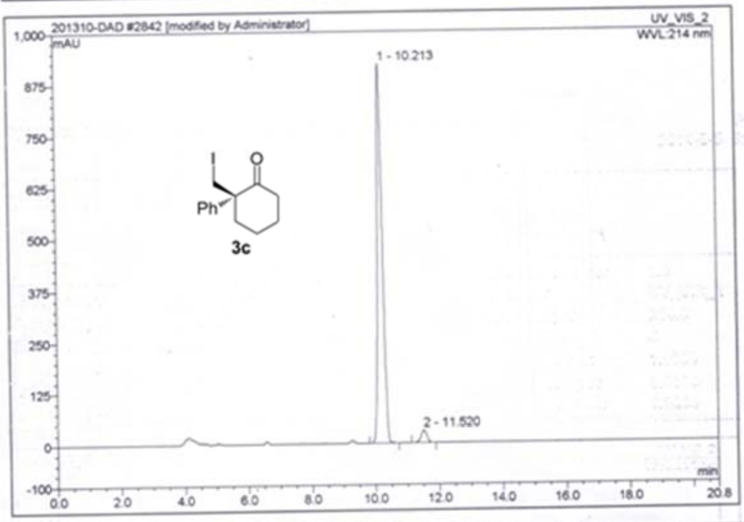

No.	Ret.Time min	Peak Name	Height mAU	Area mAU*min	Rel.Area %	Amount	Type
1	10.21	n.a.	919.844	174.636	96.33	n.a.	BMB
2	11.52	n.a.	30.743	6.663	3.67	n.a.	BMB
Total:			950.587	181.299	100.00	0.000	

Operator:GC Timebase:U3000 Sequence:WXL-2

Page 1-1
2014/9/25 3:42 下午

1608 SZG-5-47+- ID3 91 214 0.7

Sample Name:	SZG-5-47+- ID3 91 214 0.7	Injection Volume:	2.0
Vial Number:	RC6	Channel:	UV_VIS_1
Sample Type:	unknown	Wavelength:	214
Control Program:	WXL-2014-1	Bandwidth:	n.a.
Quantif. Method:	WXL	Dilution Factor:	1.0000
Recording Time:	2014/9/25 13:33	Sample Weight:	1.0000
Run Time (min):	33.10	Sample Amount:	1.0000

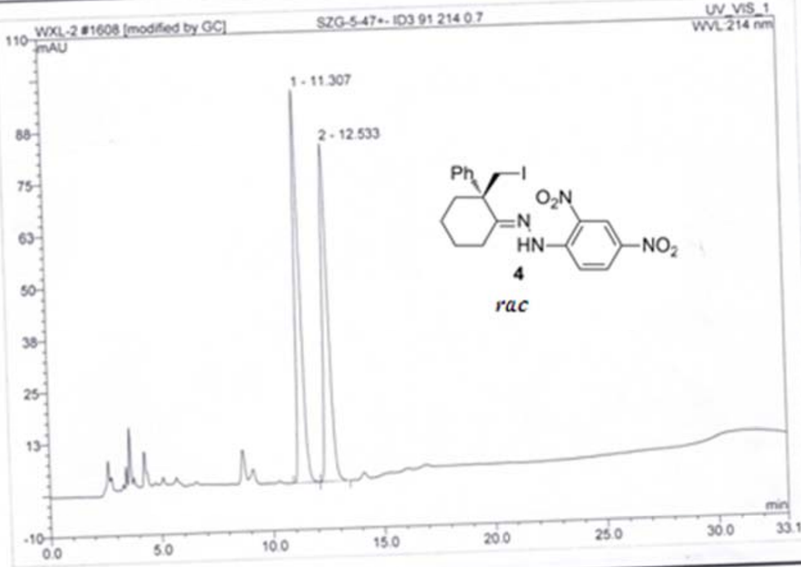

No.	Ret.Time min	Peak Name	Height mAU	Area mAU*min	Rel.Area %	Amount	Type
1	11.31	n.a.	94.862	24.029	50.48	n.a.	BMB
2	12.53	n.a.	81.671	23.573	49.52	n.a.	BMB*
Total:			176.533	47.603	100.00	0.000	

Operator:GC Timebase:U3000 Sequence:WXL-2

Page 1-1
2014/9/25 3:42 下午

1609 SZG-5-40 ID3 91 214 0.7

Sample Name:	SZG-5-40 ID3 91 214 0.7	*Injection Volume:*	3.0
Vial Number:	RC7	*Channel:*	UV_VIS_1
Sample Type:	unknown	*Wavelength:*	214
Control Program:	WXL-2014-1	*Bandwidth:*	n.a.
Quantif. Method:	WXL	*Dilution Factor:*	1.0000
Recording Time:	2014/9/25 14:13	*Sample Weight:*	1.0000
Run Time (min):	25.09	*Sample Amount:*	1.0000

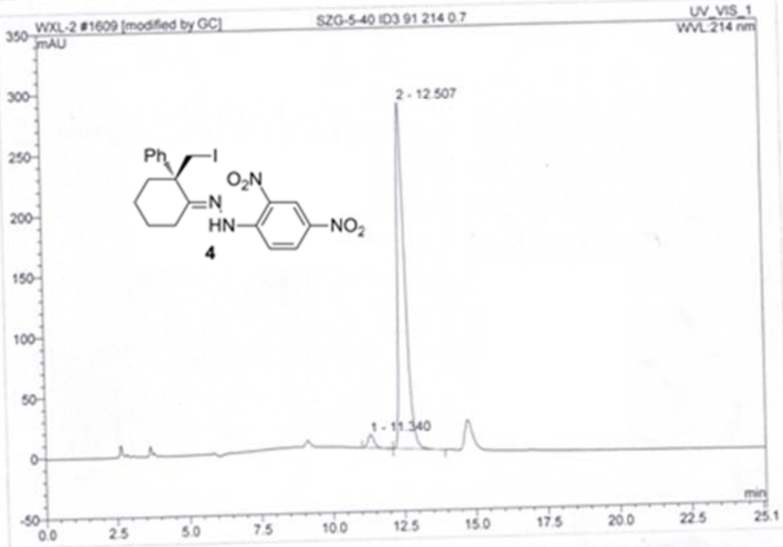

No.	Ret.Time min	Peak Name	Height mAU	Area mAU*min	Rel.Area %	Amount	Type
1	11.34	n.a.	10.808	2.665	3.08	n.a.	BM *
2	12.51	n.a.	286.668	83.844	96.92	n.a.	MB*
Total:			297.476	86.508	100.00	0.000	

1239 SZG-4-93-1+- PC-4 982 214 0.7

Sample Name:	SZG-4-93-1+- PC-4 982 214 0.7	*Injection Volume:*	2.0
Vial Number:	GC7	*Channel:*	UV_VIS_1
Sample Type:	unknown	*Wavelength:*	214
Control Program:	WXL-2014-2	*Bandwidth:*	n.a.
Quantif. Method:	WXL	*Dilution Factor:*	1.0000
Recording Time:	2014/8/11 10:13	*Sample Weight:*	1.0000
Run Time (min):	13.89	*Sample Amount:*	1.0000

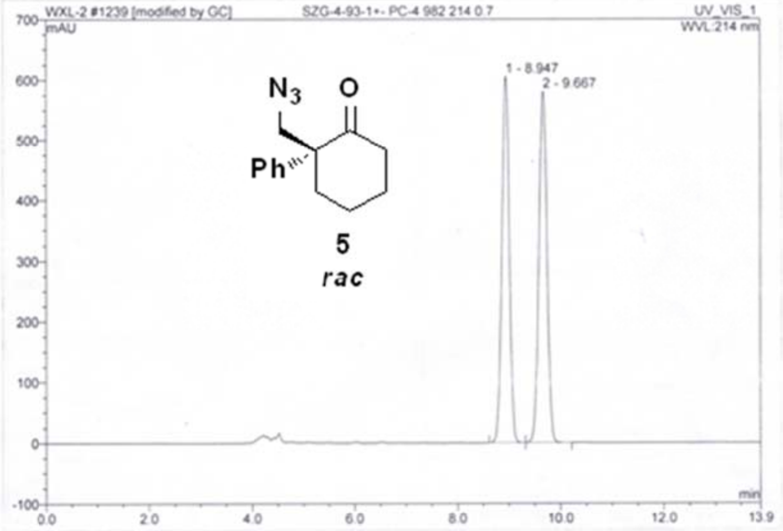

No.	Ret.Time min	Peak Name	Height mAU	Area mAU*min	Rel.Area %	Amount	Type
1	8.95	n.a.	606.479	100.463	48.79	n.a.	BMb*
2	9.67	n.a.	581.812	105.458	51.21	n.a.	bMB*
Total:			1188.291	205.921	100.00	0.000	

2728 SZG-5-7 PC-4 982 214 0.7

Sample Name:	**SZG-5-7 PC-4 982 214 0.7**	*Injection Volume:*	**1.0**
Vial Number:	**BB2**	*Channel:*	**UV_VIS_2**
Sample Type:	**unknown**	*Wavelength:*	**214.0**
Control Program:	**test-dad2**	*Bandwidth:*	**4**
Quantif. Method:	**WXL**	*Dilution Factor:*	**1.0000**
Recording Time:	**2014-8-18 14:32**	*Sample Weight:*	**1.0000**
Run Time (min):	**20.00**	*Sample Amount:*	**1.0000**

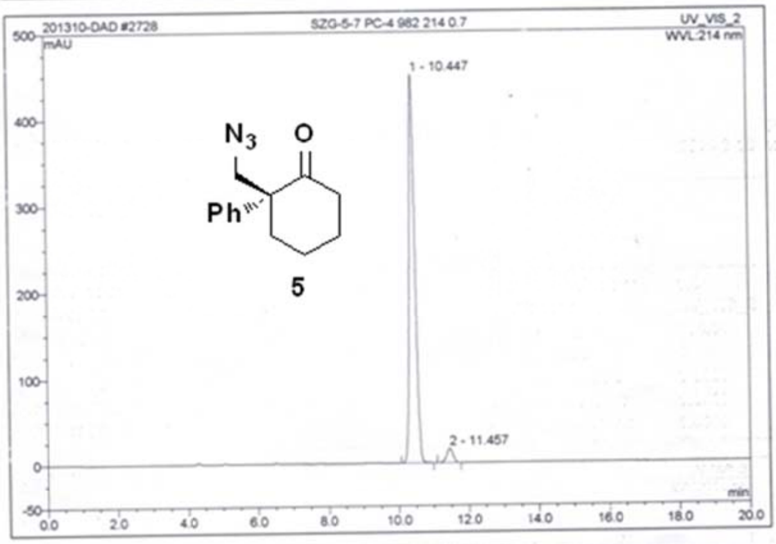

No.	Ret.Time min	Peak Name	Height mAU	Area mAU*min	Rel.Area %	Amount	Type
1	10.45	n.a.	450.431	86.466	96.10	n.a.	BMB
2	11.46	n.a.	16.622	3.505	3.90	n.a.	BMB
Total:			467.053	89.971	100.00	0.000	

2810 SZG-5-28+- PC-4 82 214 0.7

Sample Name:	SZG-5-28+- PC-4 82 214 0.7	*Injection Volume:*	2.0
Vial Number:	BE7	*Channel:*	UV_VIS_2
Sample Type:	unknown	*Wavelength:*	214.0
Control Program:	test-dad2	*Bandwidth:*	4
Quantif. Method:	WXL	*Dilution Factor:*	1.0000
Recording Time:	2014-9-4 14:20	*Sample Weight:*	1.0000
Run Time (min):	13.24	*Sample Amount:*	1.0000

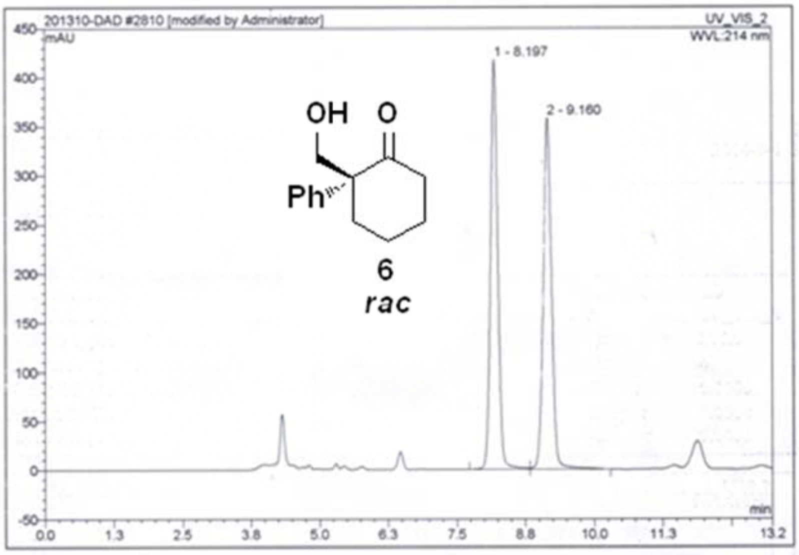

No.	Ret.Time min	Peak Name	Height mAU	Area mAU*min	Rel.Area %	Amount	Type
1	8.20	n.a.	417.751	64.331	50.18	n.a.	BM
2	9.16	n.a.	358.349	63.869	49.82	n.a.	MB
**Total:**			776.100	128.200	100.00	0.000	

2844 SZG-5-31 PC-4 82 214 0.7

Sample Name:	SZG-5-31 PC-4 82 214 0.7	*Injection Volume:*	5.0
Vial Number:	RE7	*Channel:*	UV_VIS_2
Sample Type:	unknown	*Wavelength:*	214.0
Control Program:	test-dad2	*Bandwidth:*	4
Quantif. Method:	WXL	*Dilution Factor:*	1.0000
Recording Time:	2014-9-9 11:33	*Sample Weight:*	1.0000
Run Time (min):	24.67	*Sample Amount:*	1.0000

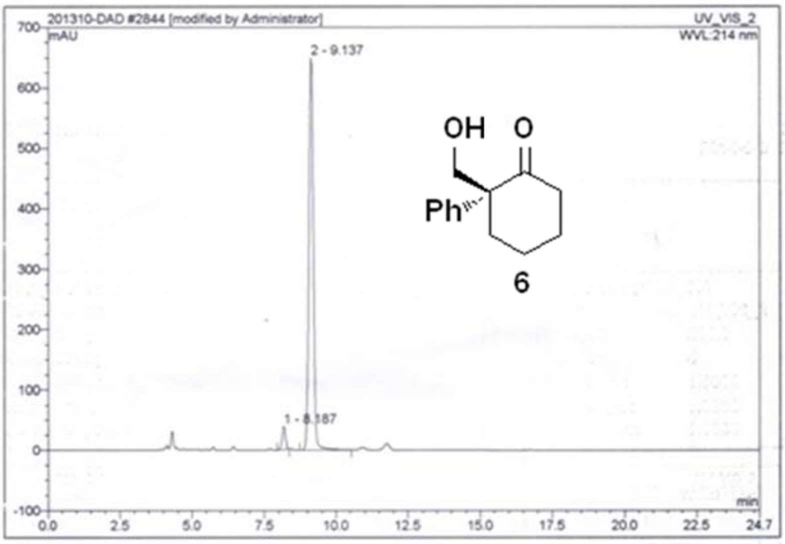

No.	Ret.Time min	Peak Name	Height mAU	Area mAU*min	Rel.Area %	Amount	Type
1	8.19	n.a.	38.636	5.591	4.64	n.a.	BMB
2	9.14	n.a.	649.484	114.866	95.36	n.a.	BMB
**Total:**			688.120	120.457	100.00	0.000	

4173 SZG-7-56-1+- PC-4 982 214 0.7

Sample Name:	SZG-7-56-1+- PC-4 982 214 0.7	*Injection Volume:*	3.0
Vial Number:	GB4	*Channel:*	UV_VIS_2
Sample Type:	unknown	*Wavelength:*	214.0
Control Program:	test-dad	*Bandwidth:*	4
Quantif. Method:	WXL	*Dilution Factor:*	1.0000
Recording Time:	2015-2-3 20:33	*Sample Weight:*	1.0000
Run Time (min):	15.00	*Sample Amount:*	1.0000

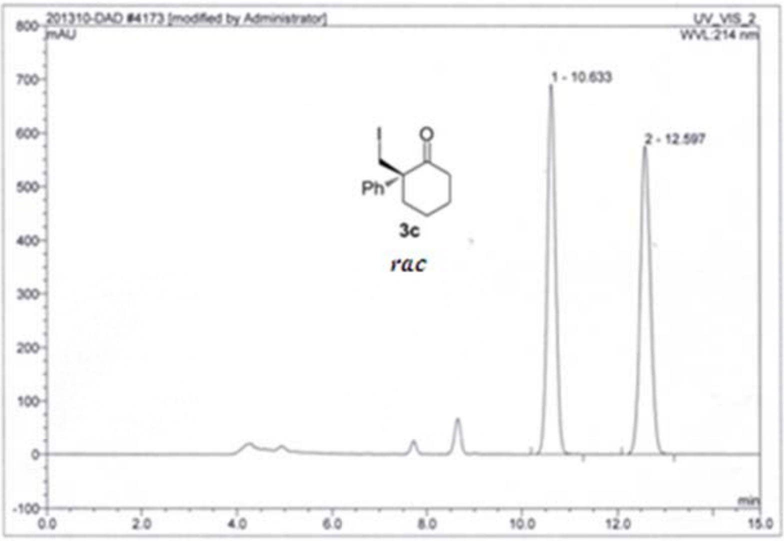

No.	Ret.Time min	Peak Name	Height mAU	Area mAU*min	Rel.Area %	Amount	Type
1	10.63	n.a.	691.829	139.886	49.45	n.a.	BMB
2	12.60	n.a.	576.657	142.996	50.55	n.a.	BMB
Total:			1268.486	282.881	100.00	0.000	

4167 SZG-7-56-1 PC-4 982 214 0.7

Sample Name:	SZG-7-56-1 PC-4 982 214 0.7	Injection Volume:	3.0
Vial Number:	GC1	Channel:	UV_VIS_2
Sample Type:	unknown	Wavelength:	214.0
Control Program:	test-dad	Bandwidth:	4
Quantif. Method:	WXL	Dilution Factor:	1.0000
Recording Time:	2015-2-3 14:54	Sample Weight:	1.0000
Run Time (min):	18.48	Sample Amount:	1.0000

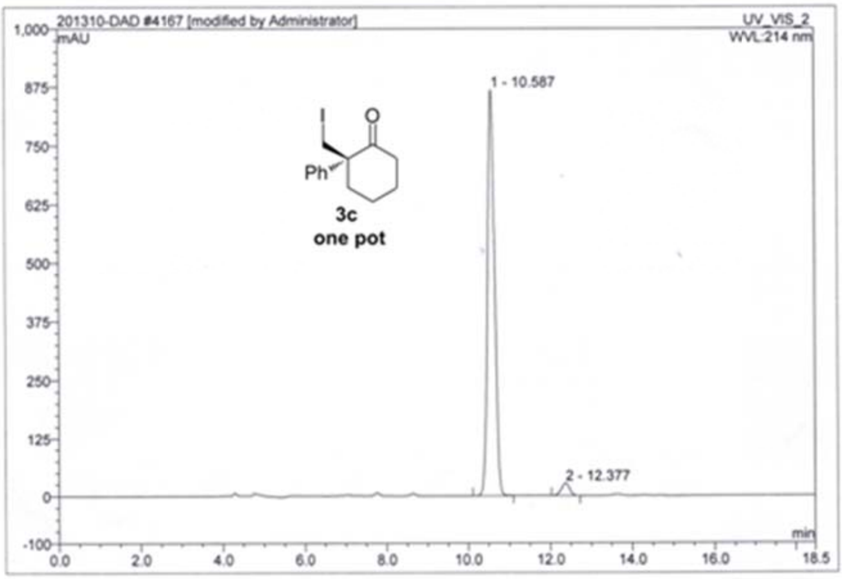

No.	Ret.Time min	Peak Name	Height mAU	Area mAU*min	Rel.Area %	Amount	Type
1	10.59	n.a.	868.349	172.897	96.47	n.a.	BM *
2	12.38	n.a.	26.696	6.330	3.53	n.a.	MB*
Total:			895.045	179.227	100.00	0.000	

Operator:Administrator Timebase:HPLC Sequence:201310-DAD

Page 1-
2015-4-4 8:55 下午

4438 SZG-7-78 PC-4 982 214 0.7

Sample Name:	**SZG-7-78 PC-4 982 214 0.7**	*Injection Volume:*	**1.0**
Vial Number:	**GB2**	*Channel:*	**UV_VIS_2**
Sample Type:	**unknown**	*Wavelength:*	**214.0**
Control Program:	**test-dad**	*Bandwidth:*	**4**
Quantif. Method:	**WXL**	*Dilution Factor:*	**1.0000**
Recording Time:	**2015-3-17 14:03**	*Sample Weight:*	**1.0000**
Run Time (min):	**25.12**	*Sample Amount:*	**1.0000**

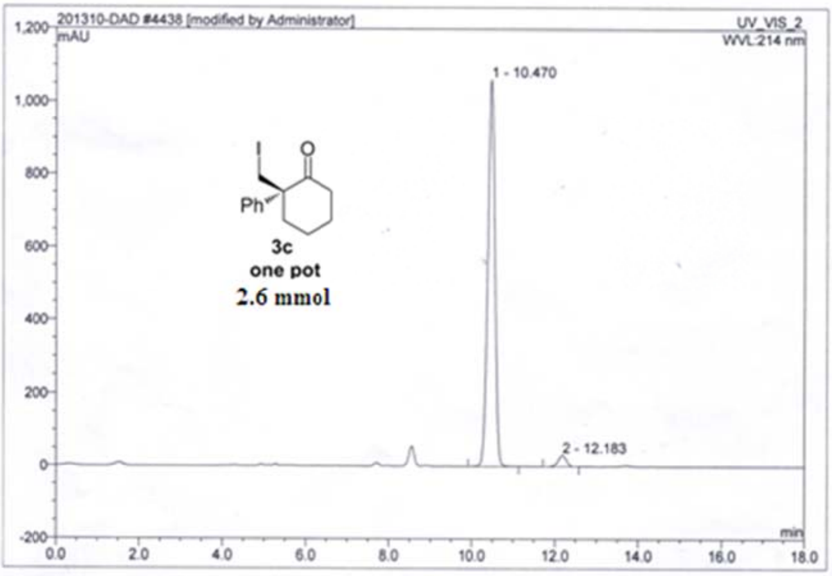

No.	Ret.Time min	Peak Name	Height mAU	Area mAU*min	Rel.Area %	Amount	Type
1	10.47	n.a.	1062.300	209.737	96.65	n.a.	BM *
2	12.18	n.a.	31.676	7.280	3.35	n.a.	BMB*
Total:			1093.975	217.017	100.00	0.000	

2895 SZG-7-58-1 IF3 982 214 0.7

Sample Name:	SZG-7-58-1 IF3 982 214 0.7	*Injection Volume:*	5.0
Vial Number:	BE1	*Channel:*	UV_VIS_1
Sample Type:	unknown	*Wavelength:*	214
Control Program:	WXL-2014-1	*Bandwidth:*	n.a.
Quantif. Method:	WXL	*Dilution Factor:*	1.0000
Recording Time:	2015/2/7 11:13	*Sample Weight:*	1.0000
Run Time (min):	18.01	*Sample Amount:*	1.0000

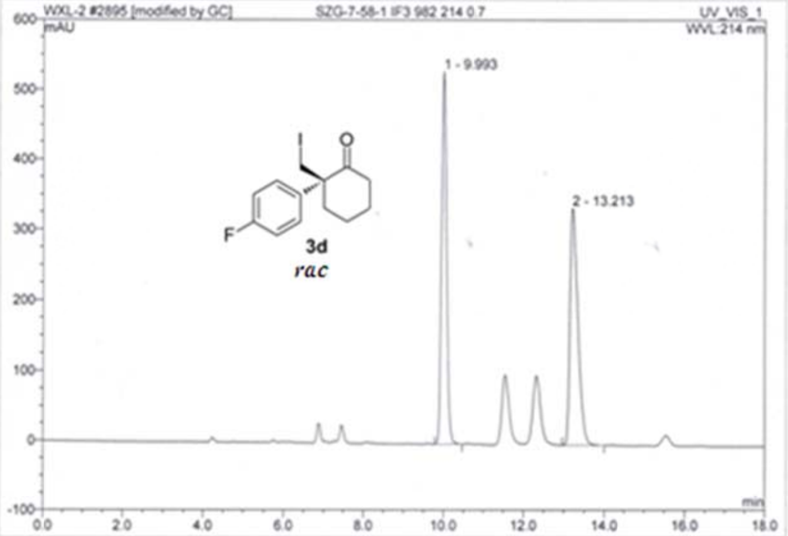

No.	Ret.Time min	Peak Name	Height mAU	Area mAU*min	Rel.Area %	Amount	Type
1	9.99	n.a.	531.138	77.547	50.28	n.a.	BMB*
2	13.21	n.a.	336.888	76.685	49.72	n.a.	MB*
Total:			868.025	154.231	100.00	0.000	

Operator:GC Timebase:U3000 Sequence:WXL-2

Page 1-
2015/2/7 2:34 下午

2896 SZG-7-55-1 IF3 982 214 0.7

Sample Name:	**SZG-7-55-1 IF3 982 214 0.7**	*Injection Volume:*	**5.0**
Vial Number:	**BE2**	*Channel:*	**UV_VIS_1**
Sample Type:	**unknown**	*Wavelength:*	**214**
Control Program:	**WXL-2014-1**	*Bandwidth:*	**n.a.**
Quantif. Method:	**WXL**	*Dilution Factor:*	**1.0000**
Recording Time:	**2015/2/7 11:33**	*Sample Weight:*	**1.0000**
Run Time (min):	**18.01**	*Sample Amount:*	**1.0000**

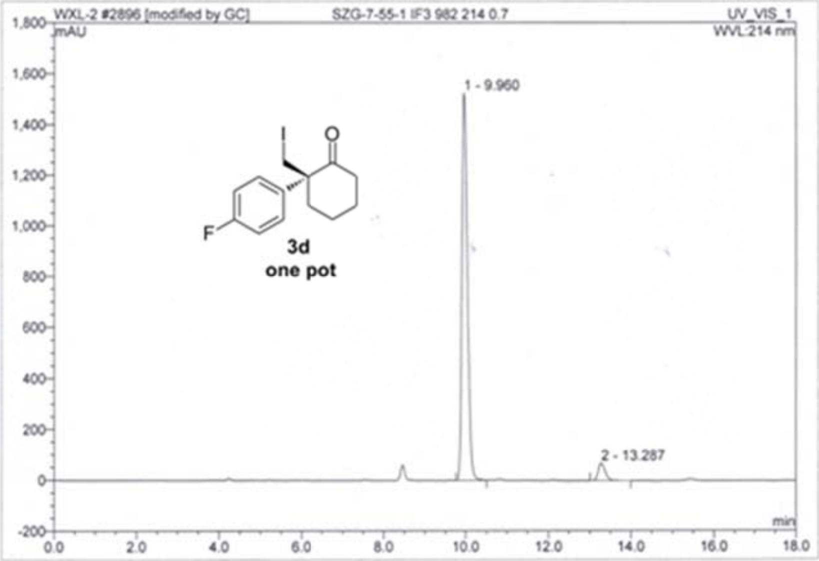

No.	Ret.Time min	Peak Name	Height mAU	Area mAU*min	Rel.Area %	Amount	Type
1	9.96	n.a.	1526.925	239.984	94.61	n.a.	BM *
2	13.29	n.a.	68.078	13.679	5.39	n.a.	BMB
Total:			1595.003	253.663	100.00	0.000	

2891 SZG-7-58-2 PC-4 991 214 0.7

Sample Name:	SZG-7-58-2 PC-4 991 214 0.7	*Injection Volume:*	5.0
Vial Number:	BC1	*Channel:*	UV_VIS_1
Sample Type:	unknown	*Wavelength:*	214
Control Program:	WXL-2014	*Bandwidth:*	n.a.
Quantif. Method:	WXL	*Dilution Factor:*	1.0000
Recording Time:	2015/2/6 18:19	*Sample Weight:*	1.0000
Run Time (min):	30.00	*Sample Amount:*	1.0000

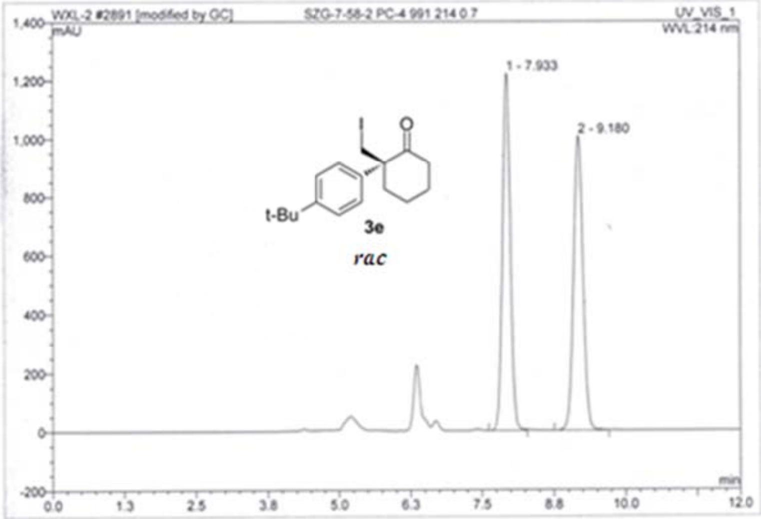

No.	Ret.Time min	Peak Name	Height mAU	Area mAU*min	Rel.Area %	Amount	Type
1	7.93	n.a.	1222.615	195.038	50.68	n.a.	BM *
2	9.18	n.a.	1007.291	189.790	49.32	n.a.	M *
Total:			2229.906	384.827	100.00	0.000	

Operator:GC Timebase:U3000 Sequence:WXL-2

Page 1
2015/2/7 9:48 上

2892 SZG-7-55-2 PC-4 991 214 0.7

Sample Name:	SZG-7-55-2 PC-4 991 214 0.7	*Injection Volume:*	5.0
Vial Number:	BC2	*Channel:*	UV_VIS_1
Sample Type:	unknown	*Wavelength:*	214
Control Program:	WXL-2014	*Bandwidth:*	n.a.
Quantif. Method:	WXL	*Dilution Factor:*	1.0000
Recording Time:	2015/2/6 20:22	*Sample Weight:*	1.0000
Run Time (min):	11.01	*Sample Amount:*	1.0000

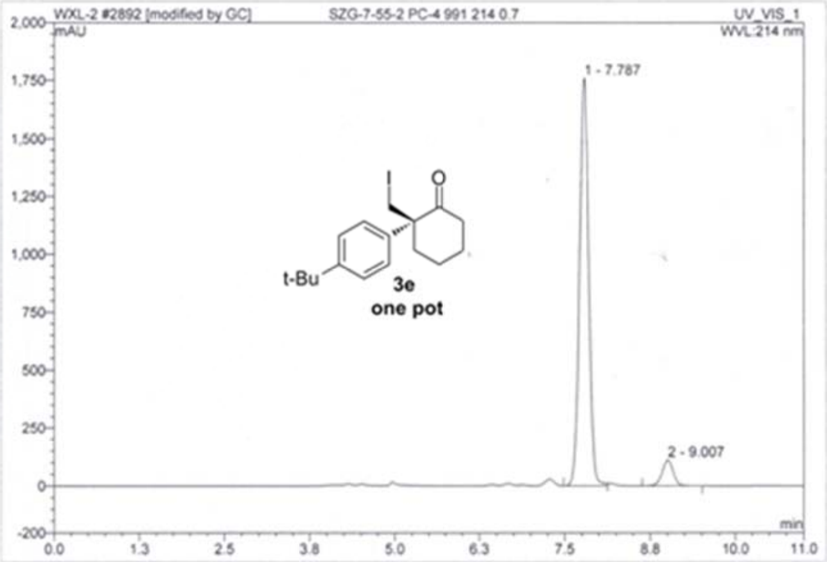

No.	Ret.Time min	Peak Name	Height mAU	Area mAU*min	Rel.Area %	Amount	Type
1	7.79	n.a.	1759.955	278.938	93.12	n.a.	BM *
2	9.01	n.a.	110.995	20.596	6.88	n.a.	MB*
Total:			1870.950	299.533	100.00	0.000	

Operator:GC Timebase:U3000 Sequence:WXL-2

Page 1-1
2015/2/7 9:44 上午

2889 SZG-7-57 PC-4 991 214 0.7

Sample Name:	SZG-7-57 PC-4 991 214 0.7	*Injection Volume:*	5.0
Vial Number:	BD1	*Channel:*	UV_VIS_1
Sample Type:	unknown	*Wavelength:*	214
Control Program:	WXL-2014	*Bandwidth:*	n.a.
Quantif. Method:	WXL	*Dilution Factor:*	1.0000
Recording Time:	2015/2/6 16:23	*Sample Weight:*	1.0000
Run Time (min):	22.95	*Sample Amount:*	1.0000

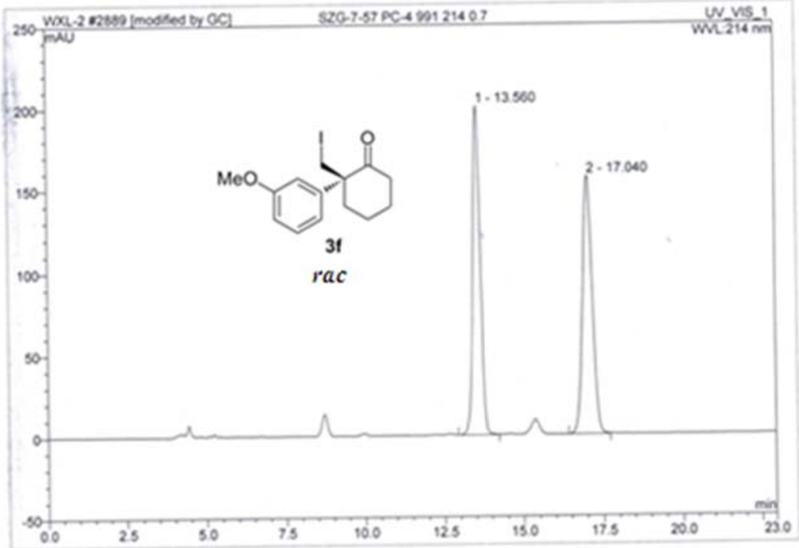

No.	Ret.Time min	Peak Name	Height mAU	Area mAU*min	Rel.Area %	Amount	Type
1	13.56	n.a.	200.590	55.209	49.99	n.a.	BM *
2	17.04	n.a.	156.760	55.228	50.01	n.a.	BM *
Total:			357.350	110.437	100.00	0.000	

Operator:GC Timebase:U3000 Sequence:WXL-2

Page 1-
2015/2/9 1:43 下午

2900 SZG-7-60-3 PC-4 991 214 0.7

Sample Name:	SZG-7-60-3 PC-4 991 214 0.7	Injection Volume:	5.0
Vial Number:	RE2	Channel:	UV_VIS_1
Sample Type:	unknown	Wavelength:	214
Control Program:	WXL-2014-1	Bandwidth:	n.a.
Quantif. Method:	WXL	Dilution Factor:	1.0000
Recording Time:	2015/2/9 10:36	Sample Weight:	1.0000
Run Time (min):	20.00	Sample Amount:	1.0000

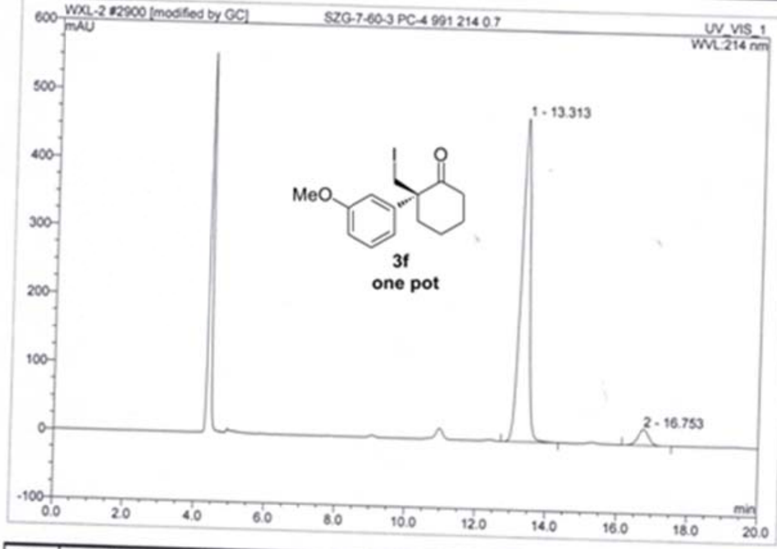

No.	Ret.Time min	Peak Name	Height mAU	Area mAU*min	Rel.Area %	Amount	Type
1	13.31	n.a.	473.013	133.596	94.00	n.a.	BM *
2	16.75	n.a.	23.463	8.527	6.00	n.a.	MB*
Total:			496.476	142.122	100.00	0.000	

Operator:Administrator Timebase:HPLC Sequence:201310-DAD

Page
2015-3-10 7:07 下

4342 SZG-4-89 PC-4 982 214 0.7

Sample Name:	SZG-4-89 PC-4 982 214 0.7	*Injection Volume:*	5.0
Vial Number:	BC6	*Channel:*	UV_VIS_2
Sample Type:	unknown	*Wavelength:*	214.0
Control Program:	test-dad	*Bandwidth:*	4
Quantif. Method:	WXL	*Dilution Factor:*	1.0000
Recording Time:	2015-3-10 18:50	*Sample Weight:*	1.0000
Run Time (min):	15.00	*Sample Amount:*	1.0000

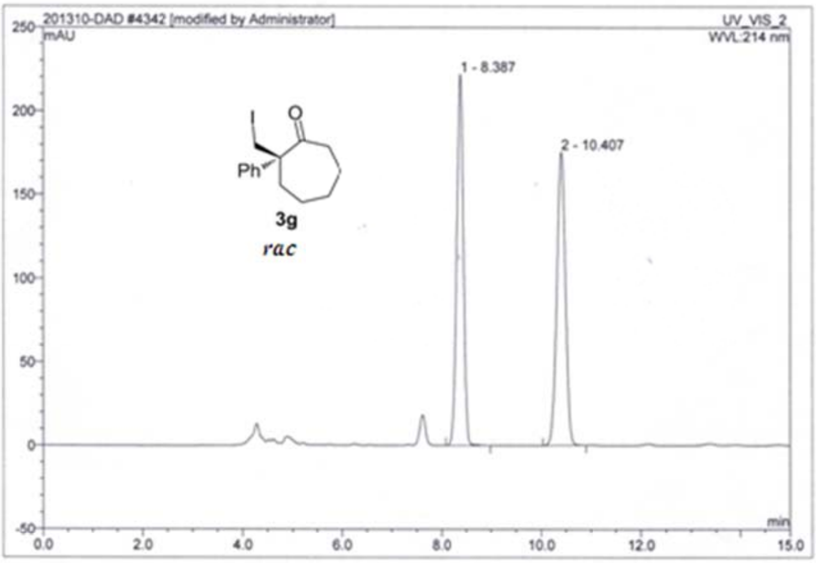

No.	Ret.Time min	Peak Name	Height mAU	Area mAU*min	Rel.Area %	Amount	Type
1	8.39	n.a.	221.806	34.096	49.53	n.a.	BMB
2	10.41	n.a.	175.754	34.748	50.47	n.a.	BMB
Total:			397.560	68.844	100.00	0.000	

4309 SZG-7-60-4B PC-4 982 214 0.7

Sample Name:	**SZG-7-60-4B PC-4 982 214 0.7**	*Injection Volume:*	**3.0**
Vial Number:	**RB1**	*Channel:*	**UV_VIS_2**
Sample Type:	**unknown**	*Wavelength:*	**214.0**
Control Program:	**test-dad**	*Bandwidth:*	**4**
Quantif. Method:	**WXL**	*Dilution Factor:*	**1.0000**
Recording Time:	**2015-3-9 9:24**	*Sample Weight:*	**1.0000**
Run Time (min):	**17.04**	*Sample Amount:*	**1.0000**

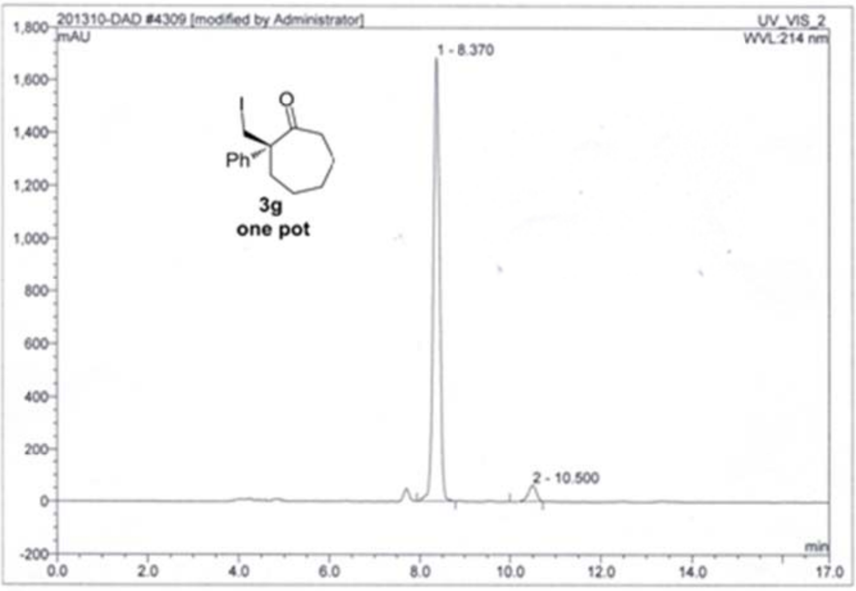

No.	Ret.Time min	Peak Name	Height mAU	Area mAU*min	Rel.Area %	Amount	Type
1	8.37	n.a.	1686.169	271.499	95.56	n.a.	BMB*
2	10.50	n.a.	61.795	12.623	4.44	n.a.	BMB*
Total:			1747.963	284.122	100.00	0.000	

8. VCD and IR experimental of 2c.

BioTools ChiralIR-2X FT-VCD spectrometer, equipped with a single photoelastic modulation (PEM) and a mercury cadmium tellurium (MCT) detector, was used to record the VCD and IR spectra. A solution of **2c** (79 mg) in $CDCl_3$ (150μL) was placed in a BaF_2 cell with a path length of 75μm. Data were acquired at a resolution of 4 cm^{-1} for 3 h. The racemic sample was measured under the same conditions to obtain VCD baseline.

VCD and IR calculations

Conformational analysis of (*R*)-**2c** was performed with Compute VOA (BioTools Inc., Jupiter, FL) using the Monte Carlo protocol at the molecular mechanic force field MMFF94 level. Within a 20 kcal/mol window, four energetically distinct conformers were predicted. Geometry optimization and frequencies calculation were carried out using the B3PW91 hybrid density functional and LANL2DZ basis set with Gaussian 09 (Gaussian Inc., Wallingford, CT). Boltzmann-population-weighted composite VCD and IR spectra were then generated by Compute VOA.

Comparisons of experimental and calculated VCD and IR spectra can be seen in Figure S2. A scaling factor of 0.96, obtained from Compare VOA (BioTools Inc., Jupiter, FL), has been applied to the calculated VCD and IR frequencies. The comparisons establish the absolute configuration of **2c** as (*R*).

The assignment was evaluated by Compare VOA. Table S7 shows the related result, including spectral similarities and enantiomeric similarity index (the difference between the VCD spectral similarity of the correct and the incorrect enantiomers, ESI). The confidence level of the (*R*) assignment is 93%, based on the current Compare VOA database consisting of 105 previous correct assignments for different chiral structures.

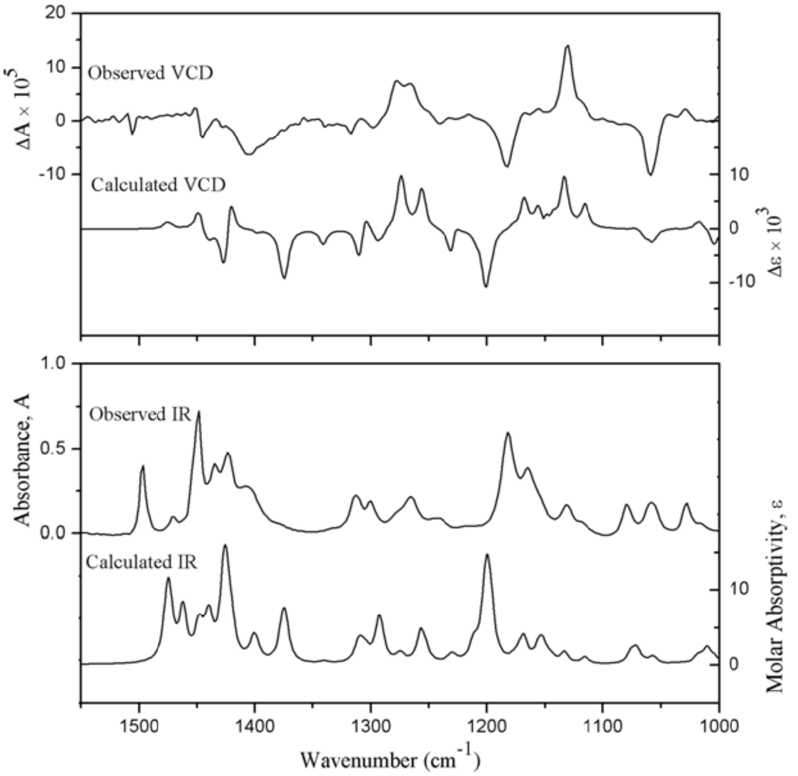

Figure S2. VCD and IR spectra observed for **2c** compared with the corresponding calculated spectra of (*R*)-**2c**.

Table S7. Compare VOA result for VCD and IR spectra of **2c**

Calculation Method	[a]S_{IR}	[b]S_R	[c]S_S	[d]*ESI*
DFT//B3PW91/LANL2DZ	74.0	65.8	9.3	56.5

[a] IR spectral similarity

[b] VCD spectral similarity for the (*R*)-configuration

[c] VCD spectral similarity for the (*S*)-configuration

[d] Enantiomeric similarity index.

9. X-ray crystallography of 2ac and 4

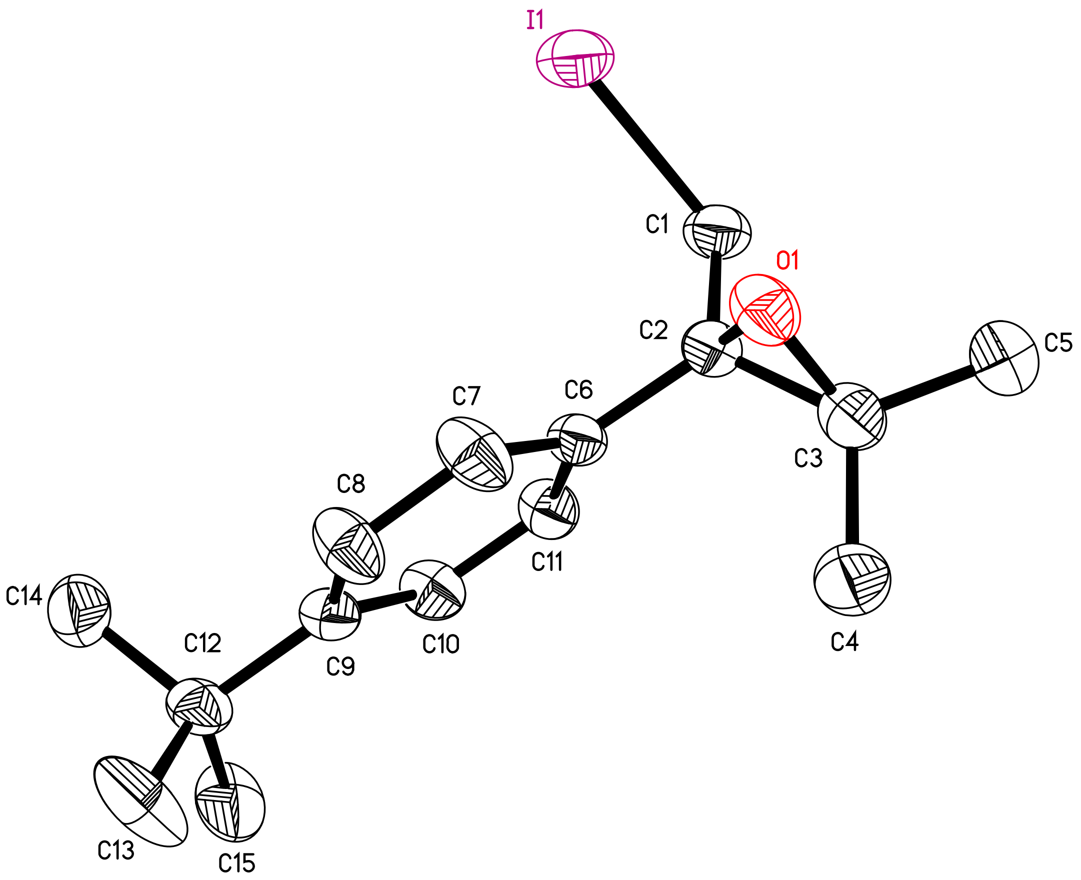

Figure S3. ORTEP drawing of **2ac**

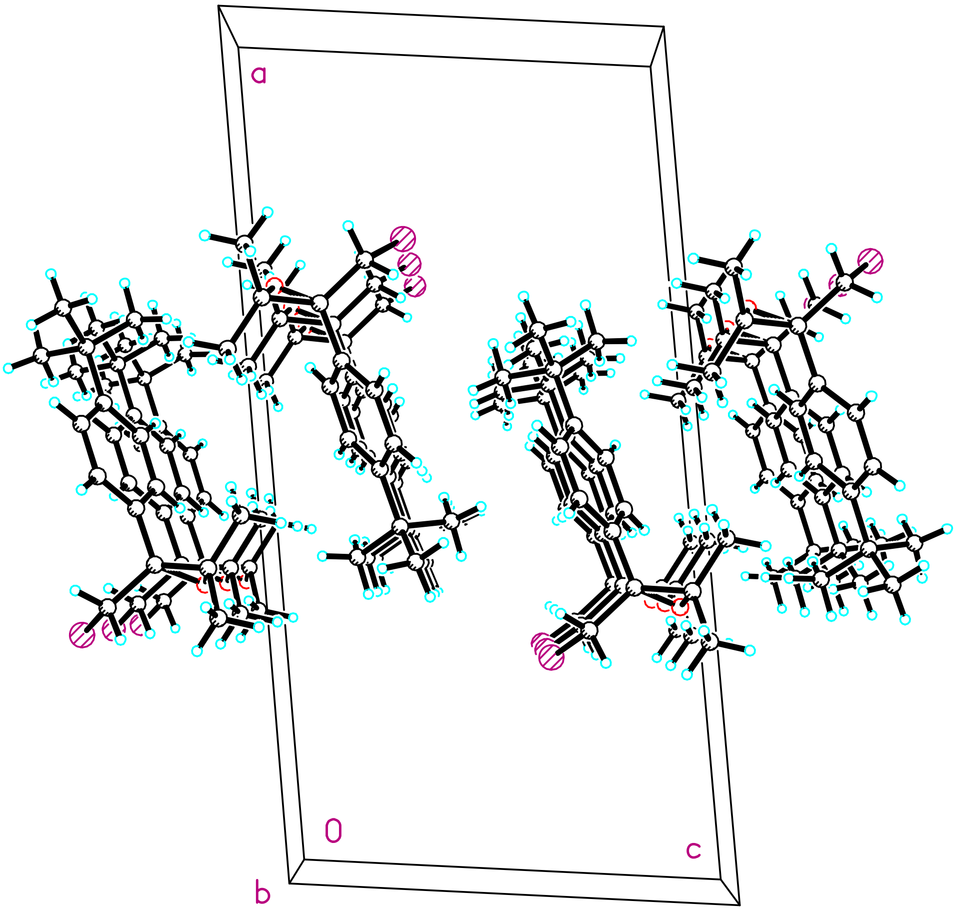

Figure S5. X-ray crystallography of **2ac**

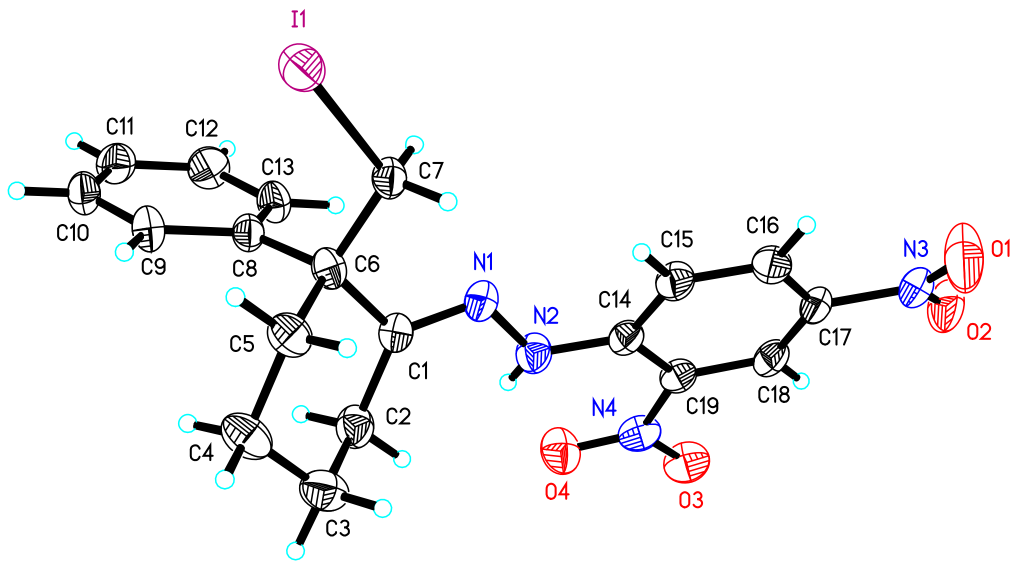

Figure S5. ORTEP drawing of **4**

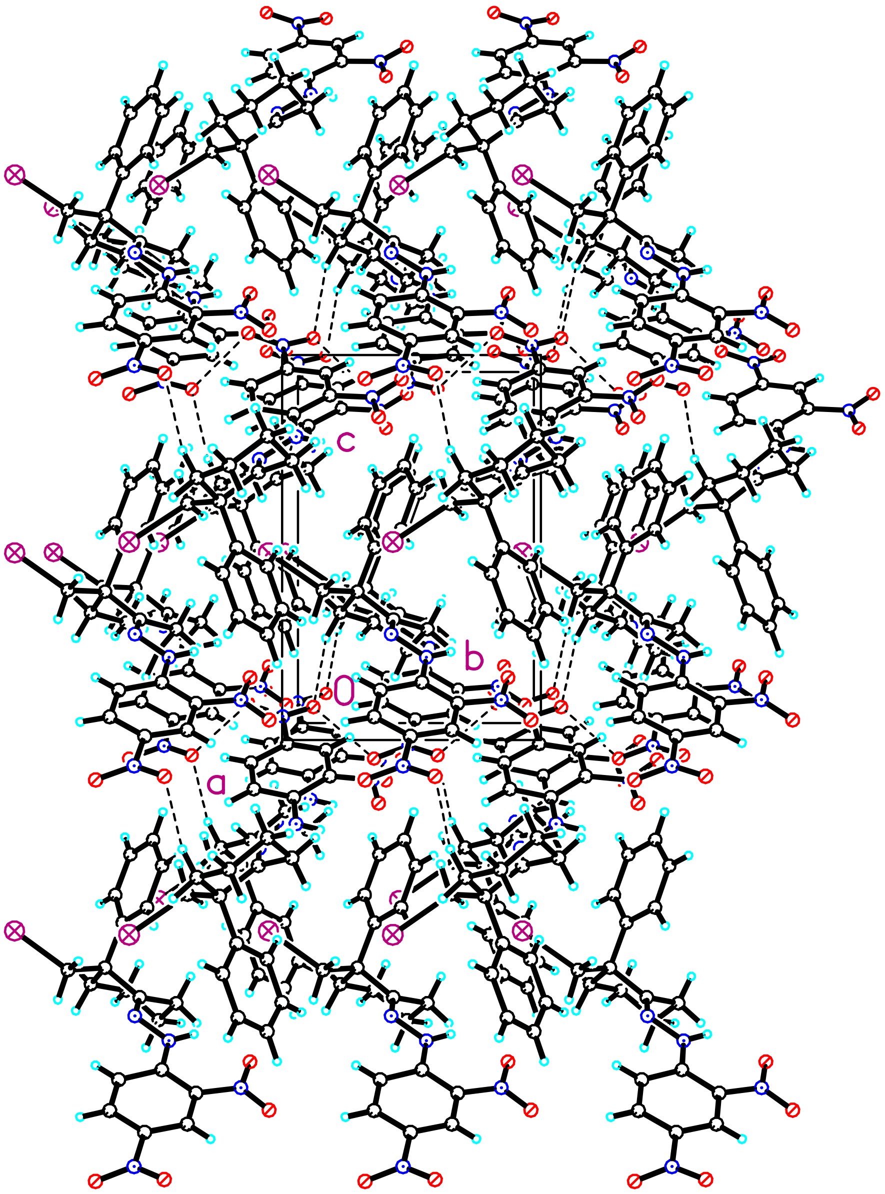

Figure S6. X-ray crystallography of **4**
